# Aminopotassiation by Mixed Potassium/Lithium Amides: A Synthetic Path to Difficult to Access Phenethylamine Derivates

**DOI:** 10.1002/anie.202009318

**Published:** 2020-10-08

**Authors:** Andreas Seymen, Ulrike Opper, Andreas Voß, Lukas Brieger, Felix Otte, Christian Unkelbach, Donal F. O'Shea, Carsten Strohmann

**Affiliations:** ^1^ Inorganic Chemistry TU Dortmund Otto-Hahn-Str. 6 44227 Dortmund Germany; ^2^ Department of Pharmaceutical and Medicinal Chemistry Royal College Surgeons 123 St. Stephen's Green Dublin 2 Ireland

**Keywords:** alkali metals, aminometalation, carbanions, potassium, synergistic effects

## Abstract

Insights gained from a comparison of aminometalation reactions with lithium amides, potassium amides and mixed lithium/potassium amides are presented. A combination of structural characterization, DFT calculations and electrophile reactions of aminometalated intermediates has shown the advantages of using a mixed metal strategy. While potassium amides fail to add, the lithium amides are uncontrollable and eliminated, yet the mixed K/Li amides deliver the best of both systems. Aminopotassiation proceeds to form the alkylpotassium species which has enhanced stability over its lithium counterpart allowing for its isolation and thereby its further characterization.

## Graphical abstract

The use of a mixed‐metal system consisting of LiNR_2_/KOR increases the selectivity of the aminometalation of styrene derivatives in favour of a potassiated intermediate over a polymerization product. Crystal structures along with quantum chemical calculations and experimental investigations demonstrate the additional advantages of potassium over lithium or sodium for the stability of metalated phenylethylamines.

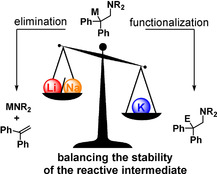

The transition metal catalysed hydroamination is an important reaction in synthetic chemistry.^[1]^ The related catalytic reaction of alkene derivatives with lithium amides has been widely investigated (Scheme 1).^[2]^ Major limitations remain for the use of polar lithium metal amides for alkene addition reactions as uncontrollable polymerization is often an occurring reaction.^[3]^ Presumably, this is due to a combination of reversible β‐elimination from addition product **A** (higher stability of **B** compared to **A**) and carbolithiation by **A** of the starting alkene substrate (higher nucleophilicity of LiCR_3_ than LiNR_2_) (Scheme 1).[^2e^, ^4^] Yet research for new approaches is a necessary topic of modern chemistry as if these undesirable features could be controlled, the synthetic scope of **A** (beyond protonation as in hydroamination) would become available via reactions with electrophiles producing **H**.^[5]^ To date, reactive intermediates such as **A** have not been isolated, making progress in addressing these issue challenging and slow.^[6]^ To access new synthetic strategies and influence the reaction pathway, these two limiting components must be overcome. At first glance, finding a means of preventing both the polymerization and β‐elimination reactions of **A** may appear contradictory. To prohibit these undesirable pathways, the reaction barrier for addition should be lowered and the carbanionic centre formed needs to be stabilized. In this account, we report our efforts to achieve this by exploiting the characteristics of different alkali metals (Li and K) in combination with stabilizing groups and allowing the thermodynamics of the reaction to select the preferred metal from a mixture of both.^[6]^

**Scheme 1:**
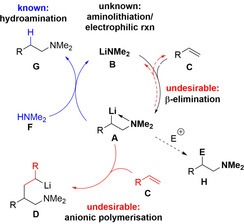
Schematic sequence and side reactions of the catalytic aminolithiation shown for the example of the addition of lithium dimethylamide (**B**) to styrene.

Within this paper, the reaction pathway too difficult to access building blocks is presented on the basis of synthesizing β‐metalated amines. At the outset of this study, the advantage that aminometalated intermediates of type **A** can be accessed by either a deprotonation of **G** or aminometalation reaction with **C** was recognized as a unique approach to investigating this challenging problem.^[7]^ The inaccessibility of **A** via an alkene aminometalation route has restricted studies which may shine light on why this route is so challenging to control. As such, we first chose to access derivatives of **A** via deprotonation using either *t*BuLi or Schlosser's base mixture of *t*BuOK and *n*BuLi which would allow a comparison of metallic reactions containing either lithium alone or both lithium and potassium. Previous work has shown the value of mixed K/Li amides for selective deprotonations which indicated that they had potential for the development of a new aminometalation strategy.^[8]^

The first substrate chosen for investigation was *N*,*N*‐dimethyl‐2,2‐diphenylethan‐1‐amine (**2 a**; Scheme 2) as the inclusion of a geminal diphenyl group should limit undesirable amide eliminations through stabilization of the metalated intermediates.^[9]^

**Scheme 2:**
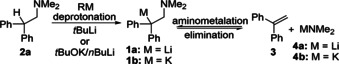
Synthesis of β‐metalated amines by deprotonation and aminometalation.

*t*BuLi and Schlosser's base (*t*BuOK/*n*BuLi) were used to achieve an irreversible deprotonation of **2 a**, and the subsequent metalated mixtures were subjected to crystallization studies. As expected, reaction of **2 a** with *t*BuLi did give deprotonation, although the aminolithiated **1 a** was not observed. In all attempts, the subsequent β‐elimination product lithium dimethylamide **[4 a**⋅**2 THF]_2_** was obtained. The lithium dimethylamide crystallizes in a mixture of THF and *n*‐pentane in the triclinic crystal system, space group *P* $\bar{1}$ (Figure 1). The asymmetric unit contains one half of an inversion symmetric dimer of the lithium amide. The central structural motif is a Li‐N rhombus. Each of the lithium cations is coordinated by two THF molecules as well as two dimethylamide groups. Attempts to in situ react **1 a** with electrophiles failed to provide products (see later for discussion). These results indicate that under the typical reaction conditions used the equilibrium lies towards the aminolithiation starting materials **3** and **4 a**, which is consistent with the known failure of this reaction.

**Figure 1:**
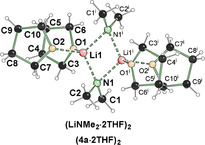
Molecular structure of **[4 a**⋅**2 THF]_2_**.^[10]^ For further information on selected bond lengths and angles see Supporting Information.

Next, deprotonation with Schlosser's mixed metal system of *t*BuOK/*n*BuLi was explored. Again, as expected, an irreversible deprotonation occurs, but in contrast to the above example, this reacting system can self‐select from either lithium or potassium, allowing either amino‐metalated species **1 a** or **1 b** to be formed. From crystallization studies of this reaction, single crystals of the metalated intermediate in which potassium was the metal of choice as in **[1 b**⋅**4 THF]_2_** were obtained. The potassiated species crystallizes at −80 °C in THF in the monoclinic crystal system, space group *P*2_1_/*n*. The asymmetric unit contains one monomer of the metalated species. The potassium is coordinated by four THF molecules, the dimethylamino group and C2, C9, C14 and C19 of the diphenyl group. The C‐K distances range from 3.065(2) Å to 3.389(2) Å. To the best of our knowledge, this is the first monomeric potassiated structure only coordinated by the solvent THF and one nitrogen since other reported monomeric structures utilize chelating nitrogen‐based ligands. By warming up the crystals of the monomeric species on the microscope slide in perfluorinated oil to −20 °C and recrystallisation, a polymeric potassiated species [**1 b**⋅**2 THF**]_∞_ is formed, which crystallized in the monoclinic crystal system, space group *P*2_1_/*c* (Figure 2).^[10]^ This transformation shows that THF can easily be removed from a potassium cation in favour of forming a polymeric potassium network. This can also be done selectively by using the reaction mixture of **[1 b**⋅**4 THF]_2_**, removing the solvent in vaccuo, resolve the residue in *n*‐pentane and crystallize the compound **[1 b**⋅**2 THF]_∞_** at −79 °C. This favouring of potassium over lithium at the carbanion centre is consistent with previous work in which we have observed that the deprotonation of toluene with a mixed K/Li base results in the formation of benzyl potassium.^[11]^

**Figure 2:**
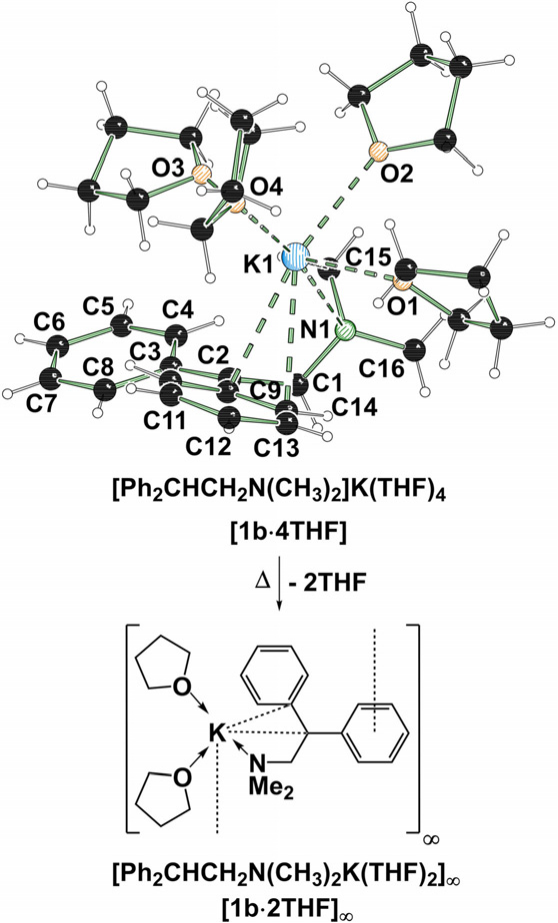
Molecular structure of **[1 b**⋅**4 THF]**.^[10]^ The Formation of a polymeric potassiated species **[1 b**⋅**2 THF]_∞_** occurs on heating the monomeric species. For further information on selected bond lengths and angles see Supporting Information.

To gain a more in‐depth understanding of the reaction mechanisms involved, quantum chemical calculations of the deprotonation and metal amide elimination reactions utilizing *t*BuLi and Schlosser's base have been performed (Scheme 3).^[12]^ The obtained structures **[1 b**⋅**4 THF]** and **[4 a**⋅**2 THF]_2_** served as basis for the calculations and a mixed Na/Li system (*t*BuLi/*t*BuONa) was included for additional comparison (Table 1). Calculations predicted that the most favourable deprotonation conditions are with Schlosser's base (63 kJ mol^−1^) followed by *t*BuLi (73 kJ mol^−1^) being the next best. Other alkyllithiums such as MeLi or *i*PrLi were less efficient and the combination of *t*BuLi with a sodium source further decreased the reactivity (86 kJ mol^−1^). Comparable results were obtained for the deprotonation of *N*,*N*‐dimethyl‐2‐phenylethan‐1‐amine and *N*,*N*‐dimethyl‐2‐phenyl‐2‐(trimethyl‐silyl)ethan‐1‐amine (see SI).

**Scheme 3:**
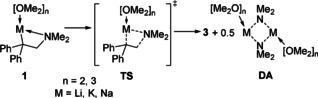
Quantum chemical calculation of the β‐elimination of different alkali metal dimethylamides (DA) using dimethylether (DME) as solvent.

**Table 1:** Results of the quantum chemical calculations of the deprotonation of diphenylethylamine **2 a** in kJ mol^−1^ with different organometallic bases; for further information regarding the modulated reaction schemes see Supporting Information; M062X/6‐31+G(d).[^12^, ^13^]

	*t*BuLi	MeLi	*i*PrLi	*t*BuOK/ *n*BuLi	*t*BuONa/ *n*BuLi
ΔΔ*H*_TS_	73	58	52	63	86
ΔΔ*H*_product_	−99	−79	−94	−99	−78

The subsequent elimination reaction has the highest barrier with potassium (activation barrier: 88 kJ mol^−1^, thermodynamics: 70 kJ mol^−1^, free energy: 36 kJ mol^−1^) and is most likely to happen with lithium compounds (activation barrier: 51 kJ mol^−1^, thermodynamics: 17 kJ mol^−1^, free energy: −15 kJ mol^−1^) (Scheme 3, Table 2).

**Table 2:** Results of the quantum chemical calculations of the β‐elimination of metal dimethylamides from **1** in kJ mol^−1^; for further information regarding the modulated reaction schemes see Supporting Information; M062X/6‐31+G(d).[^12^, ^13^]

	Li + 2 DME	K+3 DME	Na + 2 DME	Na + 3 DME
ΔΔ*H*_TS_	51	88	77	75
ΔΔ*H*_product_	17	70	43	57
ΔΔ*G*_product_	−15	36	−3	25

Comparable results were obtained for lithium, sodium and potassium metalated *N*,*N*‐dimethyl‐2‐phenylethan‐1‐amine and *N*,*N*‐dimethyl‐2‐phenyl‐2‐(trimethylsilyl)ethan‐1‐amine (see SI). Taking together the experimental and computational results strongly indicates that the undesired β‐elimination observed for aminolithiation reactions should be experimentally controllable if potassium amides are used for an addition to styrene derivatives (Scheme 4). Using 4‐methoxystyrene and alkali metal dimethyl‐amides (Li, Na, K) as substrate, quantum chemical calculations of the aminometalation reaction show that a reaction with potassium amides should be kinetically (lower addition barrier/higher elimination barrier) and thermodynamically possible (Scheme 4). In contrast, the lithium was the least favourable with sodium in between. Calculations also revealed that an aminolithiation should be kinetically hindered and, depending on the styrene derivative, could also be endothermic (see SI).

**Scheme 4:**
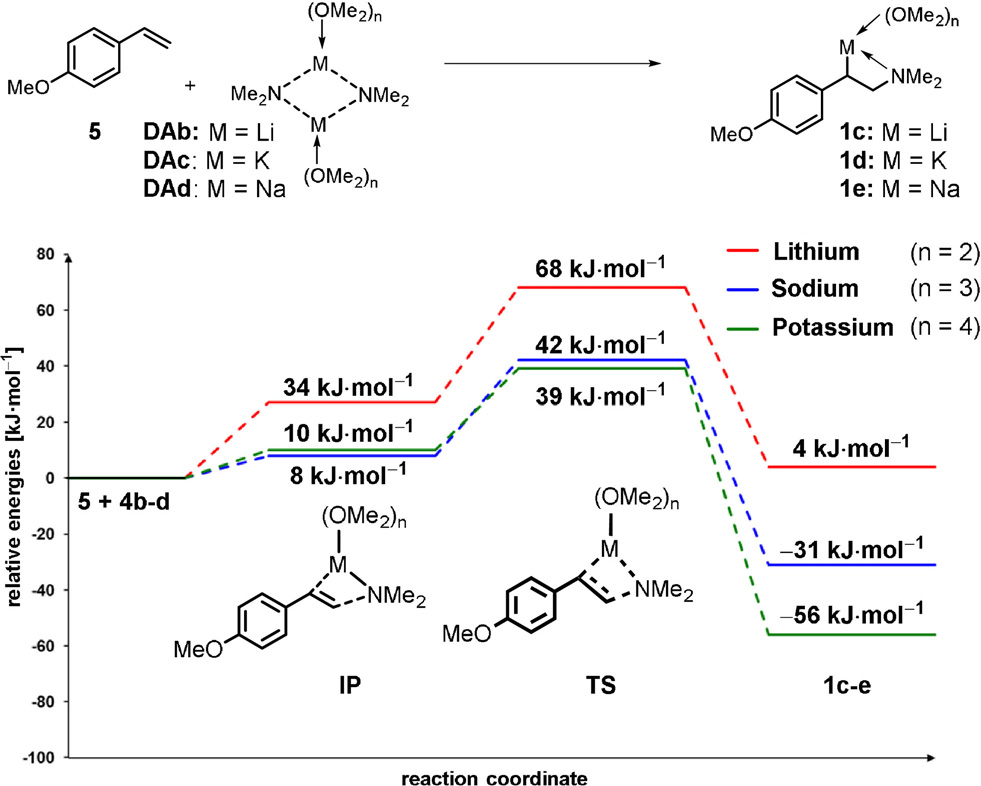
Quantum chemical calculation of the aminometalation of 4‐methoxystyrene with different alkali metal amides using DME as solvent; M062X/6‐31+G(d).[^9^, ^13^]

While computational studies showed the advantage of potassium over lithium and sodium, experimental evidence was obtained to show that potassium alone was insufficient and a mixed K/Li amide is essential for a positive reaction outcome. Using potassium piperidide on its own and in combination with potassium‐*t*‐butoxide and **5** or **3** as substrates, no aminometalation product was obtained, with starting material recovered (Scheme 5).

**Scheme 5:**
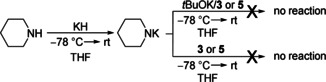
Failed aminopotassiation with potassium piperidide with and without potassium‐*tert*‐butoxide.

This illustrates that a more complex and synergistic mixed metal species is necessary to facilitate conditions for a stoichiometric aminometalation. Schlosser's base provides the two metal components and an alkoxide to in situ produce a potassium amide more suited to our needs. By mixing piperidine with *t*BuOK and *n*BuLi a more effective, synergistic mixed metal system is formed. After adding of **3**, the aminometalation with subsequent aqueous work‐up could be performed in an isolated yield of 85 % (Scheme 6). By changing the solvent to a more nonpolar 1:1 mixture of THF and *n*‐pentane we herein report the first functionalization of an aminometalated species with different electrophiles (MeOD, *n*BuBr, Me_3_SiCl).

**Scheme 6:**
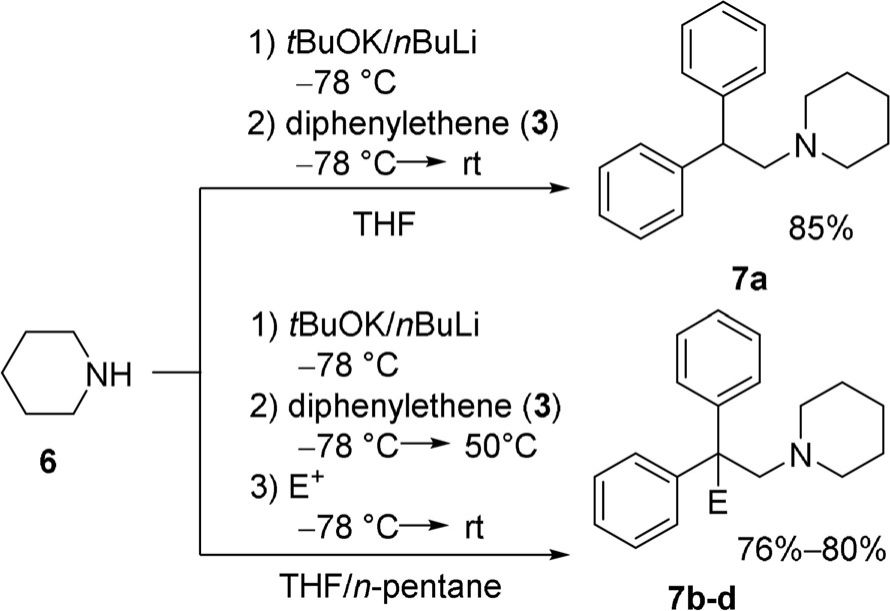
Aminometalation of 1,1‐diphenylethene (**3**) with the Schlosser's base piperidide with subsequent aqueous work‐up or functionalization with electrophiles E^+^ (MeOD, *n*BuBr, Me_3_SiCl).

By adding a second stabilizing phenyl group to the molecule as well as utilizing the effect of the potassium, the aminometalation reaction could be performed. Is the barrier lowering effect of the potassium high enough that no second stabilizing phenyl group is needed? Also, the reaction with 4‐methoxystyrene (**5**) has been performed. An isolated yield of 88 % could be obtained (Scheme 7). Unfortunately, the omission of a second stabilizing phenyl group leads to a more complicated reaction kinetic, being more sensitive towards changes of the reaction parameters and thereby hindering quenching with electrophiles. For example, as recently shown by Hevia et al., moisture plays a significant role in hydroamination reactions.^[14]^ Furthermore, a source from which the metalated 4‐methoxystyrene abstracts a proton could not be identified.^[15]^

**Scheme 7:**
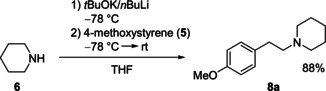
Aminometalation of 4‐methoxystyrene with the Schlosser's base piperidide with subsequent aqueous work‐up.

To prove this reaction, also the intermediate of the aminometalation reaction with a potassium amide should be isolated. Crystals of the aminometalated 1,1‐diphenylethene could be obtained (Figure 3). The species crystallizes in THF in the monoclinic crystal system, space group *P*2_1_/*n*. The structure demonstrates that an aminometalation is possible and the potassium, as already assumed in the calculations, is significantly better stabilized in the reactive intermediate than in the corresponding potassium amide because of interactions with π‐electrons.

**Figure 3:**
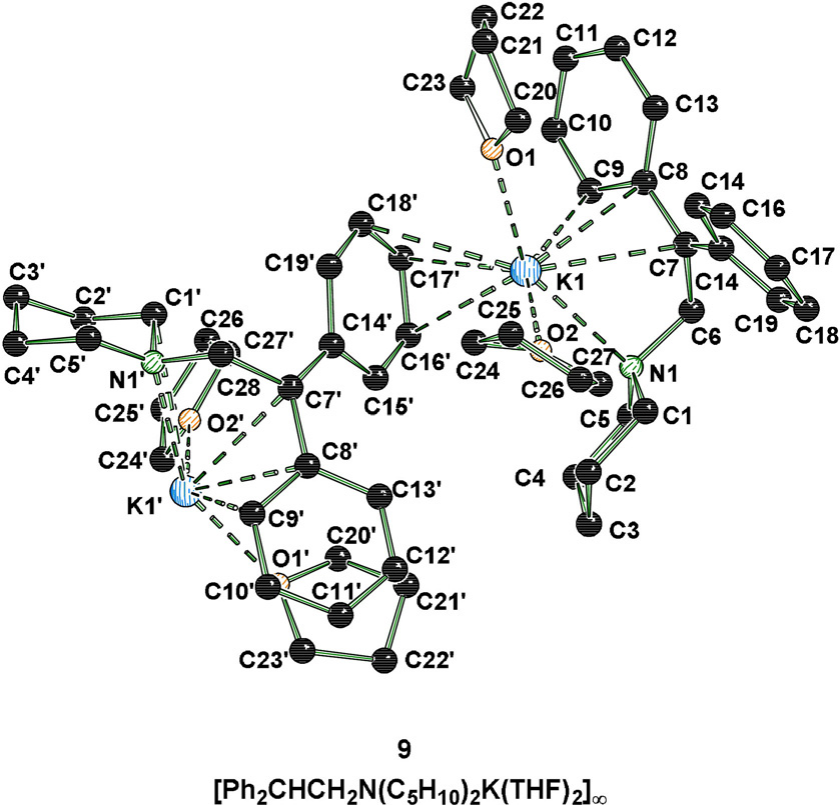
Molecular structure of **9**. The hydrogen atoms and disorder in the THF molecules are omitted for clarity.^[10]^

Moreover, another species could be obtained, which is generated during the aminometalation (Figure 4). This aggregate contains deprotonated piperidine, potassium, lithium, *tert*‐butoxide, enolate and THF as ligand. The species crystallizes in THF in the orthorhombic crystal system, space group *Pnma*. The mixed lithium/potassium structure **10** shows that the extraordinary reactivity might be increased by using this special mixture of an organolithium compound, a potassium compound and an amine. Proof of this synergistic effect was also given by using a reaction mixture without lithium (Scheme 5).^[16]^

**Figure 4:**
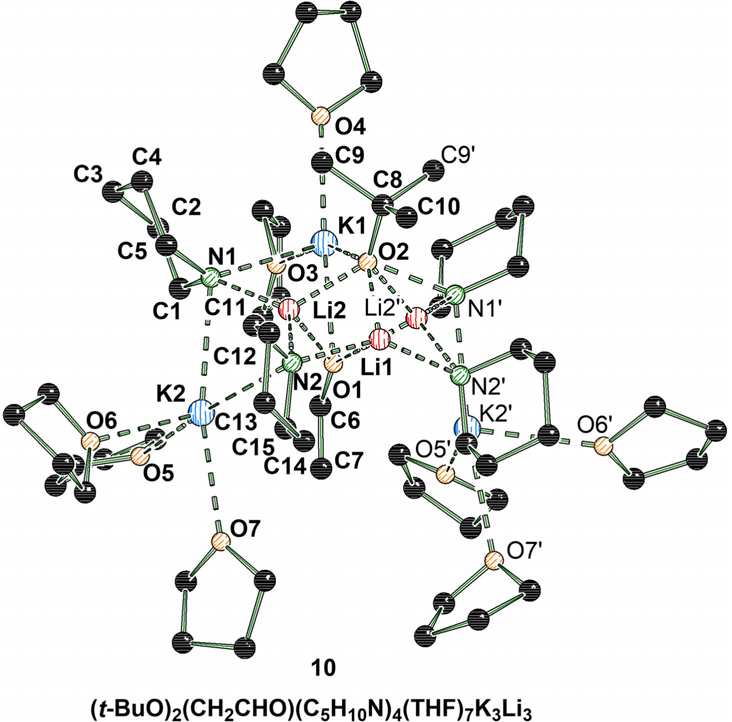
Molecular structure of **10**. The hydrogen atoms and disorder in the THF molecules are omitted for clarity.^[10]^

The molecular structure in the crystal in combination with the failed reactions of the potassiated piperidide on its own and in combination with potassium‐*tert*‐butoxide with both styrene derivatives show that the situation of the reactive potassium amide is much more complex. Considerations are needed whether parts of the structure observed in the crystal are also involved in the reaction mechanism and influence the reaction mechanism. Further anions such as the alkoxide anion seem to be necessary in addition to the amide. Additionally, alkoxides might also increase the solubility and by this increase the reactivity. Also, two different or even more alkali metal ions must be present. However, structure **10** represents only the thermodynamic minimum of a decomposition product of THF and does not show the desired reactivity.

In conclusion, highly reactive intermediates can be accessed either by deprotonation reactions of phenethylamine derivates but also by an alternative pathway: the addition of alkali metal amides to the double bond. A stoichiometric aminometalation reaction of styrene derivatives with potassium amides at low temperatures without competing polymerization reactions is presented and the possibility of quenching with different electrophiles is proven. Quantum chemical calculations based on crystallized reactive intermediates show a first insight into the reaction mechanism and explain the advantage of potassiated amides in comparison to lithiated amides in the aminometalation reactions. The isolation of a complex potassium amide aggregate delivers first explanations why a stoichiometric aminometalation works only with a mixture of lithium, potassium, amide and alkoxide.

## Conflict of interest

The authors declare no conflict of interest.

## Supporting information

As a service to our authors and readers, this journal provides supporting information supplied by the authors. Such materials are peer reviewed and may be re‐organized for online delivery, but are not copy‐edited or typeset. Technical support issues arising from supporting information (other than missing files) should be addressed to the authors.

Supplementary
