# Supplementary material for: Aminopotassiation by Mixed Potassium/Lithium Amides: A Synthetic Path to Difficult to Access Phenethylamine Derivates
Source: Angew Chem Int Ed Engl. 2020 Oct 8;59(50):22500–4. doi: 10.1002/anie.202009318 (PMC7756473; doi:10.1002/anie.202009318)
Supplement: Supplementary file 1 — Supplementary [file ANIE-59-22500-s001.pdf]

Angewandte Chemie
Eine Zeitschrift der Gesellschaft Deutscher Chemiker

Supporting Information

Aminopotassiation by Mixed Potassium/Lithium Amides: A Synthetic Path to Difficult to Access Phenethylamine Derivates

*Andreas Seymen, Ulrike Opper, Andreas Voß, Lukas Brieger, Felix Otte, Christian Unkelbach, Donal F. O'Shea, and Carsten Strohmann**

anie_202009318_sm_miscellaneous_information.pdf

Supporting Information
©Wiley-VCH 2020
69451 Weinheim, Germany

Aminopotassiation by Mixed K/Li Amides: A Synthetic Path to Difficult to Access Phenethylamine Derivatives

Andreas Seymen, Ulrike Opper, Andreas Voß, Lukas Brieger, Felix Otte, Christian Unkelbach, Donal F. O'Shea, and Carsten Strohmann*

Abstract: Insights gained from a comparison of aminometalation reactions with A Lis, A Ks and mixed lithium/A Ks are presented. A combination of structural characterization, DFT calculations and electrophile reactions of aminometalated intermediates has shown the advantages of using a mixed metal strategy. While A Ks fail to add, the A Lis are uncontrollable and eliminated, yet the mixed K/Li amides deliver the best of both. Aminopotassiation proceeds to form the alkylpotassium species which has enhanced stability over its lithium counterpart allowing for its isolation and thereby its further characterization.

DOI: 10.1002/anie.2020XXXXX

Table of Contents

1 General Remarks 02

2 Experimental Procedures 02

3 Single-Crystal X-ray Diffractional Analysis 24

4 Quantum Chemical Calculations 37

5 References 175

1 General Remarks

All reactions with oxygen- and moisture-sensitive compounds were performed under an atmosphere of argon in dried solvents, which were distilled prior to use. All other solvents and commercially available reagents, including the NMR solvents, were used without further purification.

The NMR spectra were measured on a *Bruker Avance DPX-300*, a *Bruker Avance DRX-400* and on a *Bruker Avance DRX-500* NMR spectrometer. All NMR spectra were recorded at room temperature (ca. 22 °C). Chemical shifts (δ in ppm) are referred to tetramethylsilane (TMS), with the deuterium signal of the solvent serving as internal lock and the residual solvent signal as additional reference [^{1}H-NMR: $\delta(C_6D_5H)$ = 7.16, $\delta(CHCl_3)$ = 7.27; ^{13}C-NMR: $\delta(C_6D_6)$ = 128.4, $\delta(CDCl_3)$ = 77.0]. The ^{29}Si-NMR experiments are referred to TMS as external standard and measured via the INEPT puls sequence. For the assignment of the multiplicities the following abbreviations were used: s = singlet, d = doublet, t = triplet, m = multiplet, br = broad signal. Aromatic carbon and hydrogen atoms were assigned as follows: i = ipso, o = ortho, m = meta, p = para.

Melting points were obtained using a *Büchi B-545* melting point apparatus.

Elemental analysis was performed on a *Leco Instrument CHNS-932* apparatus.

GC/EI-MS analyses were obtained using an Agilent 7890B GC system (column: Agilent HP-5MS, 30 m, 0.25 mm, 0.25 µm) with an Agilent 5977A Mass Selective Detector.

For X-Ray crystal structure analysis: Compounds **1b 4THF**, **[1b·2THF]∞, 9** and **10** were recorded on the *Bruker D8 Venture* four-circuit diffractometer from *Bruker AXS GmbH*. Area counter CMOS detector used: *Photon100* from *Bruker AXS GmbH*; X-ray sources: Microfocus source *IµS* Mo from *Incoatec GmbH* with mirror optics HE-LIOS and single-hole collimator from *Bruker AXS GmbH*. Used programs for data collection: *APEX3 Suite* (v2016.9-0) and integrated programs *SAINT* (integration) and *SADABS* (absorption correction) from *Bruker AXS GmbH*.

Compounds **(4a·2THF)$_2$** was recorded on the *CrysAlis CCD* from *Oxford Diffraction Ltd.* using the implemented programs to collect and process the data: CrysAlis (Oxford, 2008; data collection); CrysAlis RED (Oxford, 2008; cell determination and refinement); empirical absorption correction.

The solution of the crystal structures was done with the program *SHELXT*, the structure refinement with SHELXS.[1] The processing and finalization of the crystal structure data was done with the program OLEX^{2}.[2] Tables of atomic coordinates can be found in the appendix: U_{eq} is defined as one third of the trace of the orthogonalized tensor U_{ij}. For the hydrogen atoms the standard values of the SHELXL program were used with Uiso(H) = –1.2 Ueq(C) for CH_2 and CH and with U_{iso}(H) = –1.5 U_{eq}(C) for CH_3. Tables of the anisotropic deflection parameters are given in

the Tables S2 and S3. The exponent of the anisotropic deflection factor has the form: $-2\pi^2[h^2 \cdot a^* \cdot 2U^{11} + ... + 2 \cdot h \cdot k \cdot a^* \cdot b^* \cdot U^{12}]$.

For the selection of air and moisture sensitive crystals the *X-TEMP 2* system was used in combination with a *SMZ1270* stereomicroscope from *Nikon Metrology GmbH*.[3] *MicroMounts* or *MicroLoops* from *MiTeGen* were used for mounting.

2 Experimental Procedures

2.1 Deprotonation of *N*,*N*-Dimethyl-2,2-diphenyl-ethyl-1-amine with the Schlosser's Base

2.1.1 Functionalization with Methanol-d$_4$

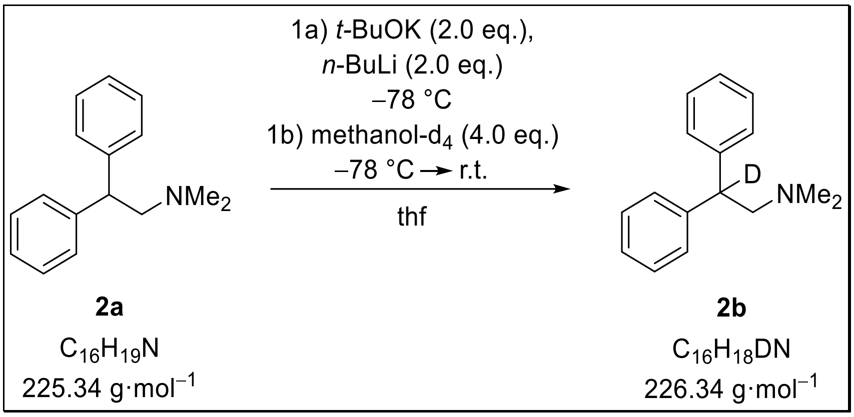

To a stirred solution of *N*,*N*-Dimethyl-2,2-diphenyl-ethyl-1-amine (0.225 g, 1.0 mmol, 1.0 eq.) and potassium-*tert*-butoxide (0.224 g, 2.0 mmol, 2.0 eq.) in THF (10 ml) at –78 °C was added *n*-butyllithium (0.8 ml, 2.0 mmol, 2.0 eq.; 2.5 M in hexane). The reaction mixture was stirred for 1 h, methanol-d$_4$ (0.144 g, 4.0 mmol, 4.0 eq.) was added at –78 °C and the reaction mixture was stirred for 1 h at this temperature, was allowed to warm to room temperature and stirred at room temperature for 1 h. After adding distilled water (30 ml), the organic layer was separated and the aqueous phase was extracted with diethylether (3 x 30 ml). The combined organic layers were dried with Na_2SO_4 and the solvent removed. The resulting yellow residue was purified by kugelrohr distillation (oven temperature: 140 °C, pressure: 1.4 mbar) yielding 0.180 g (0.78 mmol, 78%) of the deuterated product **2b**, which was identified by MS and ^{1}H-NMR, but could not be clearly detected because of the poor resolution of the ^{13}C-NMR spectrum.

^{1}H-NMR (400.3 MHz, C_6D_6): δ = 4.17 [s, 6H; N(C*H*$_3$)$_2$], 4.89 [s, 2H; NC*H*$_2$], 9.12–9.33 [m, 10H; *H*$_{arom.}$].

{^{1}H}^{13}C-NMR (100.6 MHz, C_6D_6): δ = 47.7 [2C; N(*C*H$_3$)$_2$], 67.1 [1C; N*C*H$_2$CD], 81.7 [1C; NCH$_2$*C*D], 129.8 [2C; *C*$_{para}$], 130.5 [8C; *C*$_{ortho,}$ *C*$_{para}$], 146.0 [2C; *C*$_{ipso}$].

**CHN Analysis:**	calculated:	C 84.91 %	H 8.40 %	N 6.19 %
	found:	C 85.1 %	H 8.3 %	N 6.1 %

GC/EI-MS: (70 eV, t_R = 12.10 min): m/z (%): 225 (1) [M–H$^+$], 209 (1) [(M-Me)$^+$], 179 (2) [(M-NMe$_2$)$^+$], 166 (9) [$C_{13}H_{10}D^+$], 91 (1) [$C_7H_6D^+$], 58 (100) [$C_3H_8N^+$].

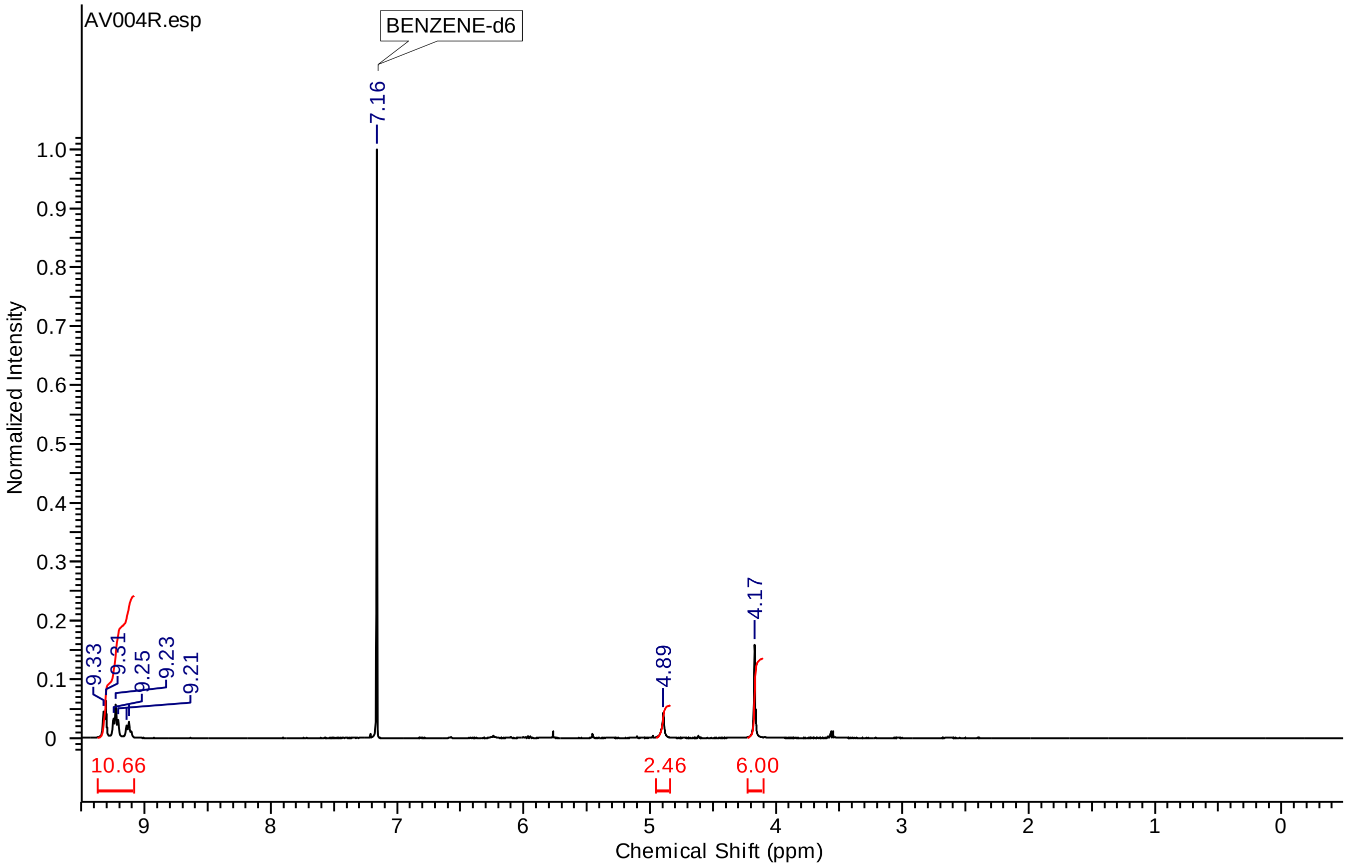

Figure 1: ^{1}H-NMR of compound **2b.**

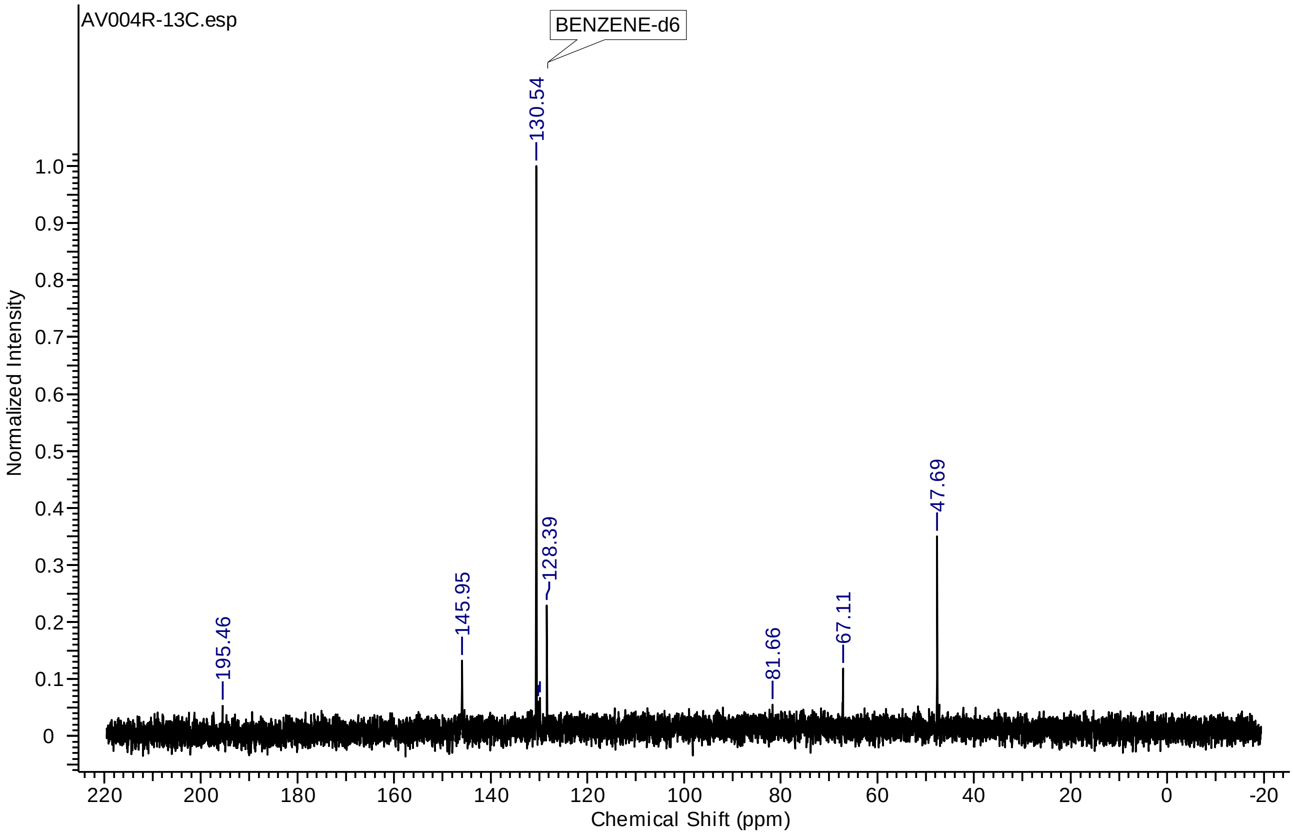

Figure S2: {^{1}H}^{13}C-NMR of compound **2b**.

2.1.2 Functionalization with *n*-Butylbromide

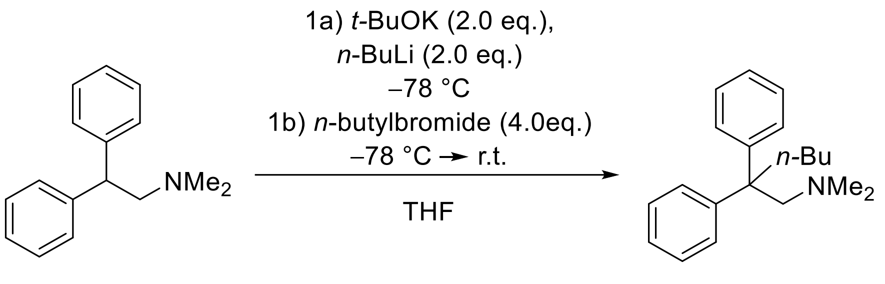

2a $C_{16}H_{19}N$ 225.34 g·mol^{-1} → **2c** $C_{20}H_{27}N$ 281.44 g·mol^{-1}

To a stirred solution of *N*,*N*-Dimethyl-2,2-diphenyl-ethyl-1-amine (0.225 g, 1.0 mmol, 1.0 eq.) and potassium-*tert*-butoxide (0.224 g, 2.0 mmol, 2.0 eq.) in THF (10 ml) at –78 °C was added *n*-butyllithium (0.8 ml, 2.0 mmol. 2.0 eq.; 2.5 M in hexane). The reaction mixture was stirred for 1 h, *n*-butylbromide (0.548 g, 4.0 mmol, 4.0 eq.) was added at –78 °C and the reaction mixture was stirred for 1 h at this temperature, was allowed to warm to room temperature and stirred at room temperature for 1 h. After adding distilled water (30 ml), the organic layer was separated and the aqueous phase was extracted with diethylether (3 x 30 ml). The combined organic layers were dried with Na_2SO_4 and the solvent removed. The resulting yellow residue was purified by kugelrohr distillation (oven temperature: 140 °C, pressure: 1.4 mbar) yielding 0.205 g (0.73 mmol, 73%) of the functionalized product **2c**.

^{1}H-NMR (400.3 MHz, C_6D_6): δ = 0.82 [t, ^{3}J(H,H) = 7.3 Hz, 3H; $CCH_2CH_2CH_2CH_3$], 1.01–1.09 [m, 2H; $CCH_2CH_2CH_2CH_3$], 1.25–1.32 [sxt, ^{3}J(H,H) = 7.3 Hz, 2H; $CCH_2CH_2CH_2CH_3$], 1.89 [m, 6H; $N(CH_3)_2$], 2.34–2.39 [m, 2H; $CCH_2CH_2CH_2CH_3$], 2.94 [s, 2H; NCH_2], 7.02-7.21 [m, 10H; $H_{arom.}$].

{^{1}H}^{13}C-NMR (100.6 MHz, C_6D_6): δ = 14.7 [1C; $CCH_2CH_2CH_2CH_3$], 24.2 [1C; $CCH_2CH_2CH_2CH_3$], 27.5 [1C; $CCH_2CH_2CH_2CH_3$], 37.0 [1C; $CCH_2CH_2CH_2CH_3$], 48.5 [2C; $N(CH_3)_2$], 51.0 [1C; NCH_2C], 66.8 [1C; NCH_2C], 126.2 [2C; C_{para}], 128.7 [4C; C_{ortho}], 129.1 [4C; C_{meta}], 149.3 [2C; C_{ipso}].

**CHN Analysis:**				
	calculated:	C 85.35 %	H 9.67 %	N 4.98 %
	found:	C 85.5 %	H 9.6 %	N 4.6 %

GC/EI-MS: (70 eV, t_R = 14.50 min): m/z (%): 280 (1) [M^+], 252 (1) [$(M\text{-}Me_2)^+$], 224 (1) [$(M\text{-}Bu)^+$], 165 (8) [$(C_{13}H_{11})^+$], 91 (6) [$(C_7H_6)^+$], 77 (1) [$(C_6H_5)^+$], 58 (100) [$(C_3H_8N)^+$].

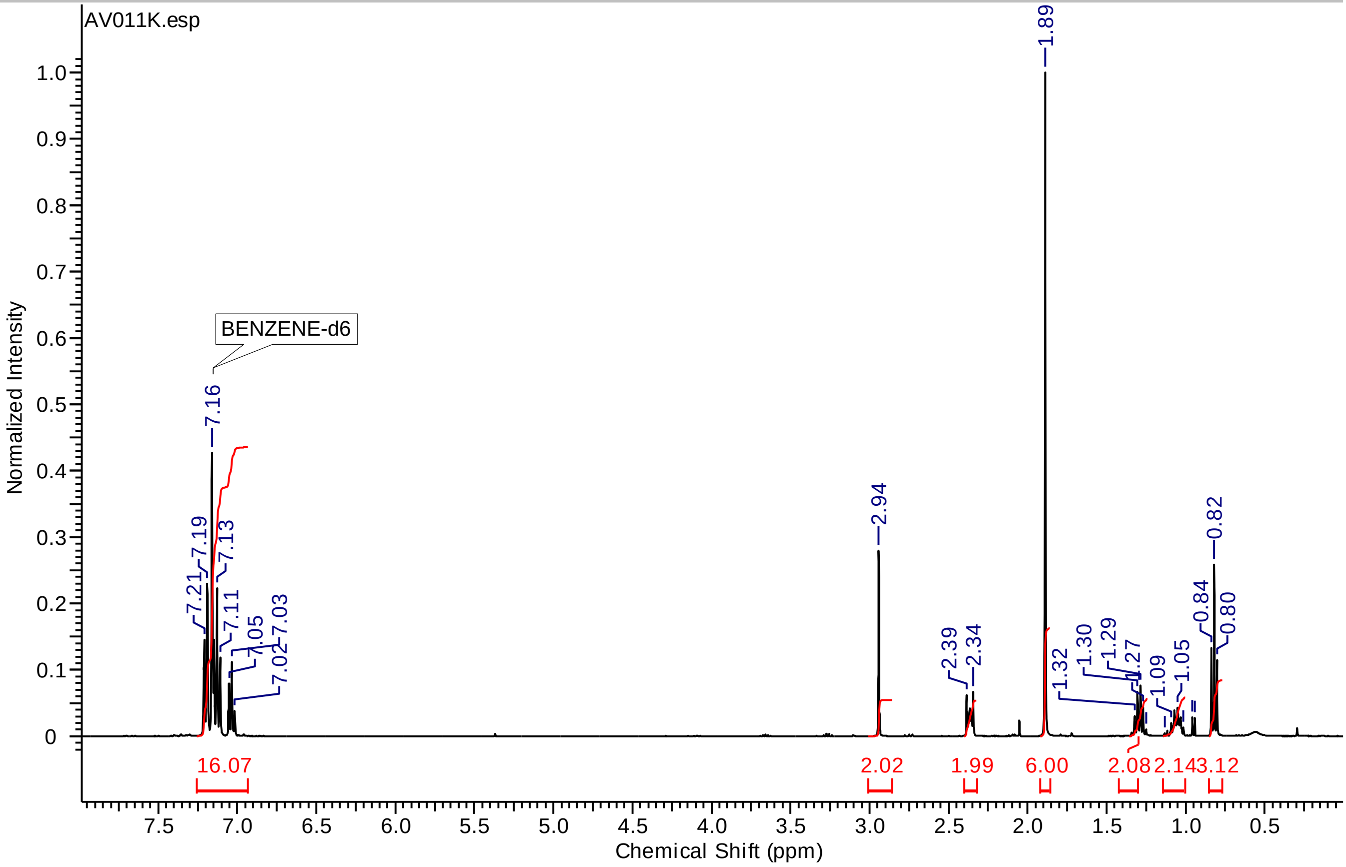

Figure S3: ^{1}H-NMR of compound **2c**.

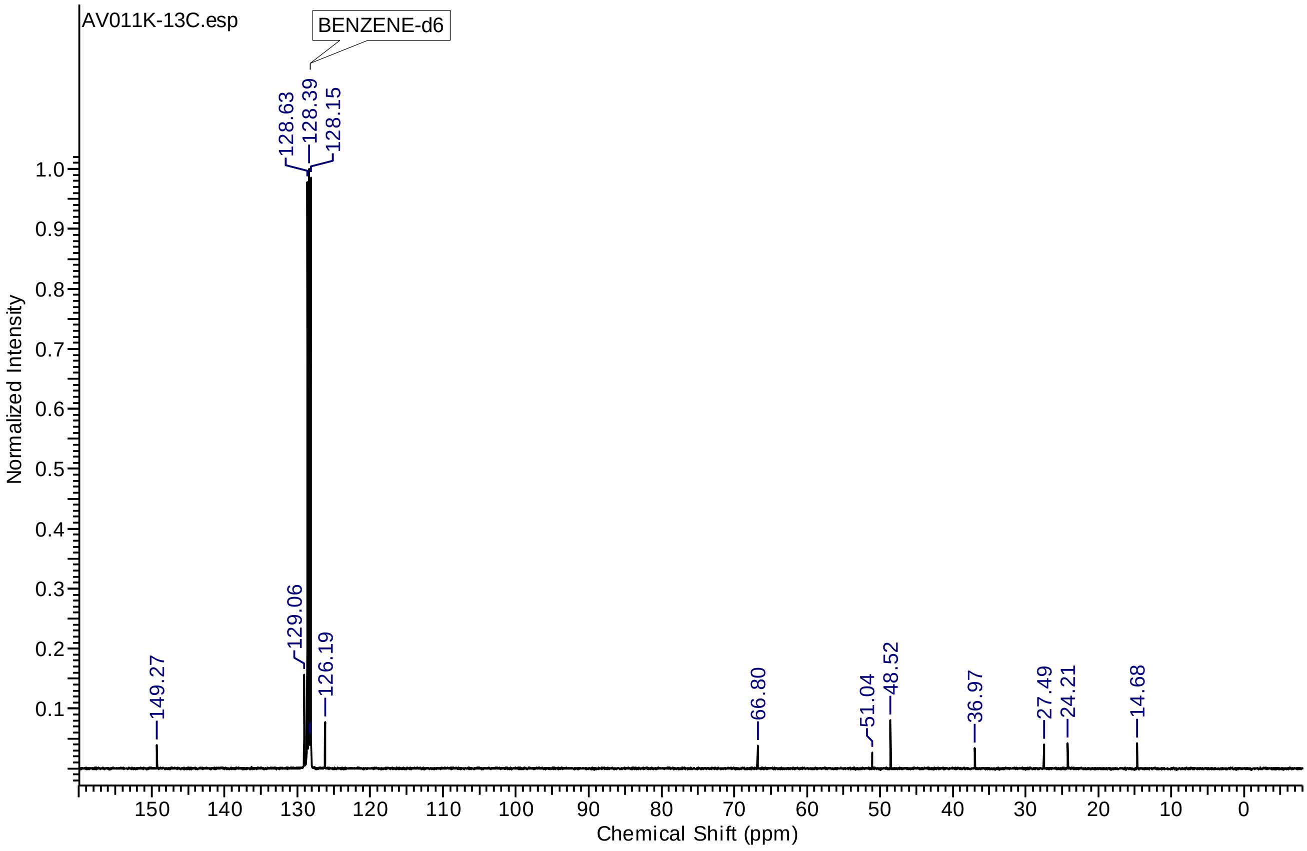

Figure S4: {^{1}H}^{13}C-NMR of compound **2c**.

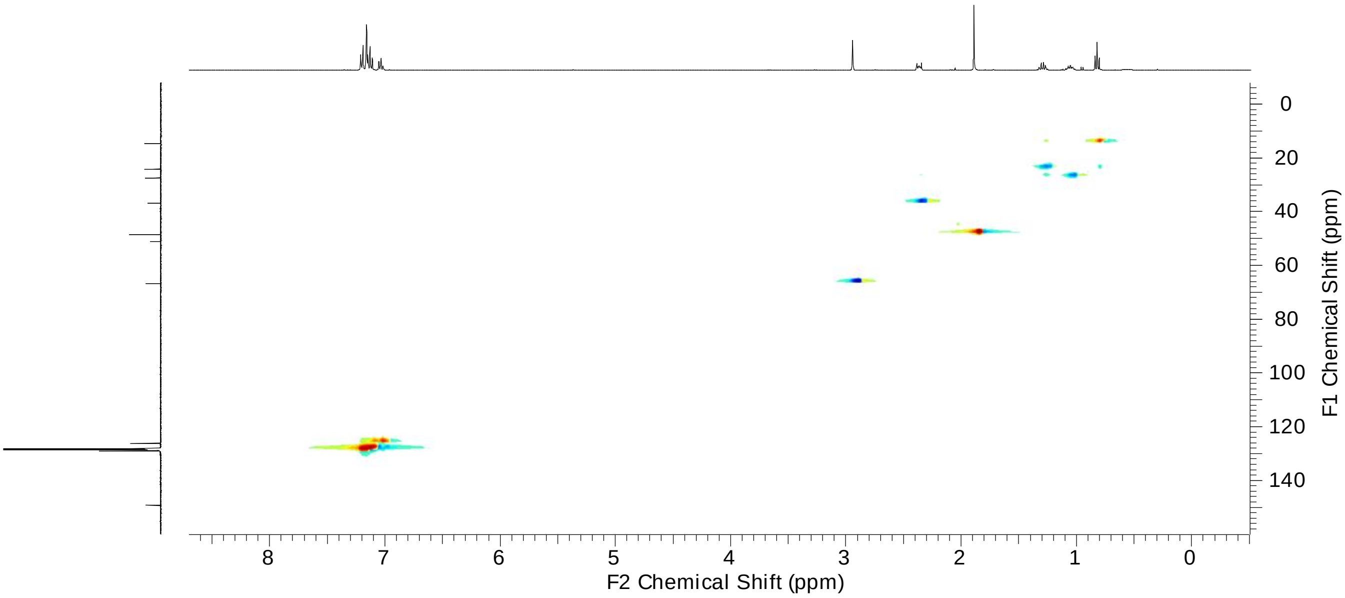

Figure S5: ^{1}H,^{13}C-HSQC-NMR of compound **2c**.

2.2 Aminometalation of 4-Methoxystyrene Using Piperidine and Potassium Hydride

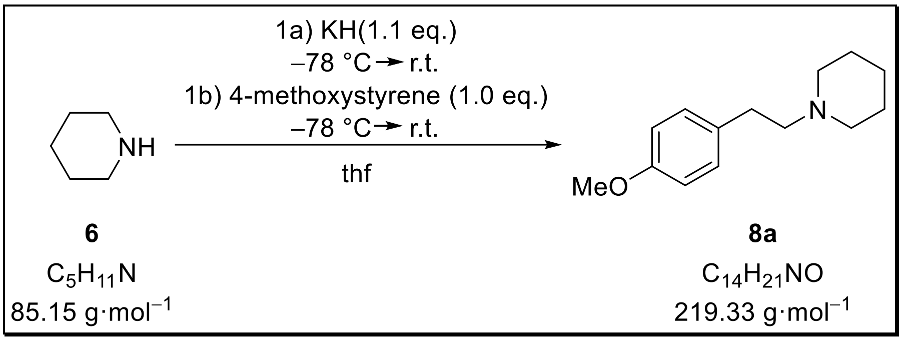

To a stirred solution of piperidine (172 mg, 2.0 mmol, 1.0 eq.) in THF (15 ml) potassium hydride (80 mg, 2.0 mmol, 1.0 eq.) was added at –78 °C and stirred for an hour at room temperature. Again the reaction mixture was cooled down to –78 °C again and 4-methoxystyrene (270 mg, 2.0 mmol, 1.0 eq.) was added. The reaction mixture was allowed to slowly warm to –60 °C and was stirred for 1.5 h at this temperature and 30 min at room temperature. After adding distilled water (15 ml), the organic layer was separated and the aqueous phase was extracted with diethylether (3 x 15 ml). The combined organic layers were dried with Na_2SO_4 and the solvent removed. The resulting yellow residue was analysed by GC-chromatography and showed only unreacted 4-methoxystyrene.

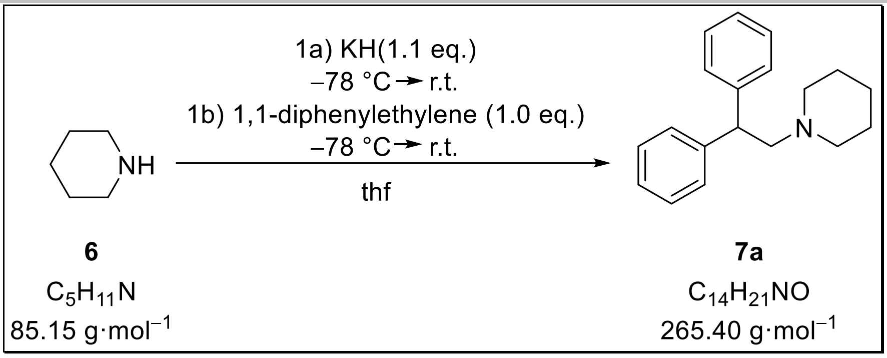

To a stirred solution of piperidine (172 mg, 2 mmol, 1 eq.) in THF (15 ml) potassium hydride (80 mg, 2.0 mmol, 1.0 eq.) was added at –78 °C and stirred for an hour at room temperature. Again the reaction mixture was cooled down to –78 °C again and 1,1-diphenylethene (360 mg, 2.0 mmol, 1.0 eq.) was added. The reaction mixture was allowed to slowly warm to –60 °C and was stirred for 1.5 h at this temperature and 30 min at room temperature. After adding distilled water (15 ml), the organic layer was separated and the aqueous phase was extracted with diethylether (3 x 15 ml). The combined organic layers were dried with Na_2SO_4 and the solvent removed. The resulting yellow residue was analysed by GC-chromatography and showed only unreacted 1,1-diphenylethene.

2.3 Aminometalation of Piperidine Using Potassium Hydride and Potassium-*tert*-butoxide

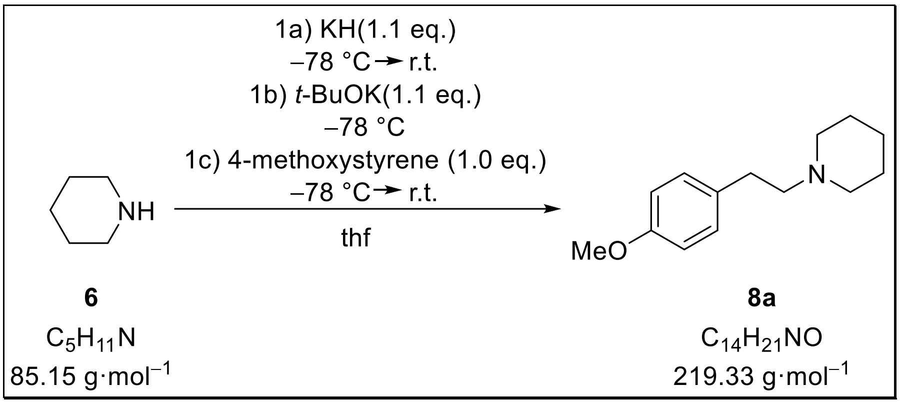

To a stirred solution of piperidine (172 mg, 2.0 mmol, 1.0 eq.) in THF (15 ml) potassium hydride (80 mg, 2.0 mmol, 1.0 eq.) was added at –78 °C and stirred for an hour at room temperature. Then potassium-*tert*-butoxide (224 mg, 2.0 mmol, 1.0 eq.) was added and the reaction mixture was cooled down to –78 °C again. At –78 °C 4-methoxystyrene (270 mg, 2.0 mmol, 1.0 eq.) was added. The reaction mixture was allowed to slowly warm to –60 °C and was stirred for 1.5 h at this temperature and 30 min at room temperature. After adding distilled water (15 ml), the organic layer was separated and the aqueous phase was extracted with diethylether (3 x 15 ml). The combined organic layers were dried with Na_2SO_4 and the solvent removed. The resulting yellow residue was analysed by GC-chromatography and showed only unreacted 4-methoxystyrene.

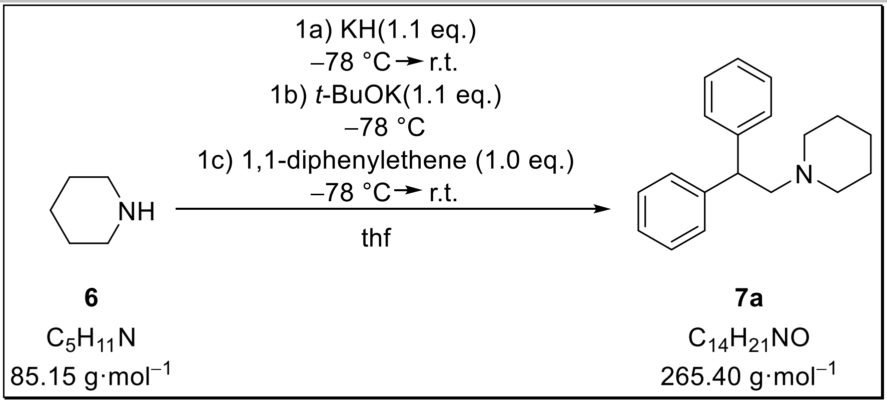

To a stirred solution of piperidine (172 mg, 2 mmol, 1 eq.) in THF (15 ml) potassium hydride (80 mg, 2.0 mmol, 1.0 eq.) was added at –78 °C and stirred for an hour at room temperature. Then potassium-*tert*-butoxide (224 mg, 2.0 mmol, 1.0 eq.) was added and the reaction mixture was cooled down to –78 °C again. At –78 °C 1,1-diphenylethene (360 mg, 2.0 mmol, 1.0 eq.) was added. The reaction mixture was allowed to slowly warm to –60 °C and was stirred for 1.5 h at this temperature and 30 min at room temperature. After adding distilled water (15 ml), the organic layer was separated and the aqueous phase was extracted with diethylether (3 x 15 ml). The combined organic layers were dried with Na_2SO_4 and the solvent removed. The resulting yellow residue was analysed by GC-chromatography and showed only unreacted 1,1-diphenylethene.

2.4 Aminometalation of Piperidine Using Potassium-*tert*-butoxide and *n*-Butyllithium

2.3.1 Aminometalation of Piperidine to 1,1-Diphenylethene

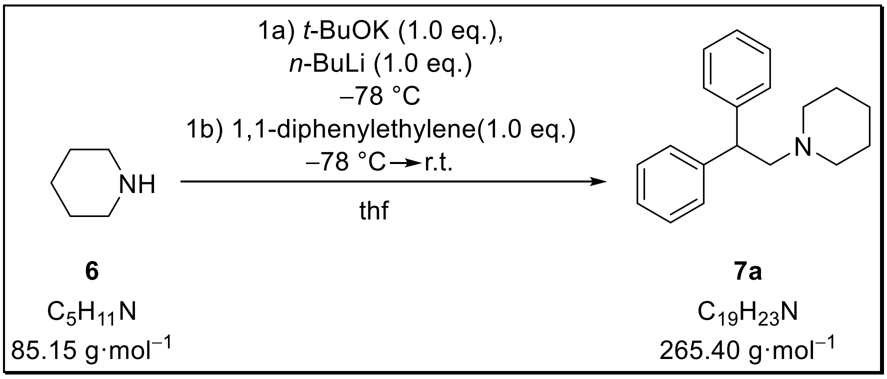

To a stirred solution of piperidine (172 mg, 2.0 mmol, 1.0 eq.) and potassium-*tert*-butoxide (224 mg, 2.0 mmol, 1.0 eq.) in THF (15 ml) at –78 °C was added n-butyllithium (0.8 ml, 2.0 mmol, 1.0 eq.; 2.5 M in hexane). The reaction mixture was stirred for one hour. At –78 °C 1,1-diphenylethene (360 mg, 2.0 mmol, 1.0 eq.) was added. The reaction mixture was allowed to slowly warm to –60 °C and was stirred for 1.5 h at this temperature and 30 min at room temperature. After adding distilled water (15 ml), the organic layer was separated and the aqueous phase was extracted with diethylether (3 x 15 ml). The combined organic layers were dried with Na_2SO_4 and the solvent removed. The resulting yellow residue was purified by kugelrohr distillation (oven temperature: 160 °C, pressure: 0.39 mbar) yielding 0.448 mg (1.69 mmol, 85%) of the aminometalation product **7a**.

^{1}H-NMR (400.2 MHz, benzene-d_6): δ = 1.20–1.27 [m, 2H; $\mathrm{NCH_2CH_2C}H_2$], 1.35–1.41 [m, 4H; $\mathrm{NCH_2C}H_2\mathrm{CH_2}$], 2.28–2.31 [m, 4H; $\mathrm{N}CH_2\mathrm{CH_2CH_2}$], 2.82 [d, ^3J(H,H) = 7.6 Hz, 2H; $\mathrm{N}CH_2\mathrm{CH}$], 4.18 [t, ^3J(H,H) = 7.6 Hz, 1H; $\mathrm{NCH_2C}H$], 7.01–7.22 [m, 10H; arom. *H*].

{^{1}H}^{13}C-NMR (176.1 MHz, benzene-d_6): δ = 25.2 [1C; $\mathrm{NCH_2CH_2}C\mathrm{H_2}$], 26.7 [2C; $\mathrm{NCH_2}C\mathrm{H_2CH_2}$], 49.5 [1C; $\mathrm{NCH_2}C\mathrm{H}$], 55.4 [2C; $\mathrm{N}C\mathrm{H_2CH_2CH_2}$], 65.4 [1C; $\mathrm{N}C\mathrm{H_2CH}$], 126.7 [2C; C_{para}], 128.9 [2C; C_{ortho}], 129.2 [1C; C_{meta}], 145.0 [1C; C_{ipso}].

CHN Analysis:

calculated:	C 85.99 %	H 8.74 %	N 5.28 %
found:	C 86.1 %	H 8.8 %	N 5.3 %

GC/EI-MS: (70 eV, t_R = 15.91 min): m/z (%): 264 (1) [$M-H^+$], 165 (14) [$M-C_6H_{14}N^+$], 98 (100) [$C_6H_{12}N^+$], 77 (3) [$C_6H_5^+$].

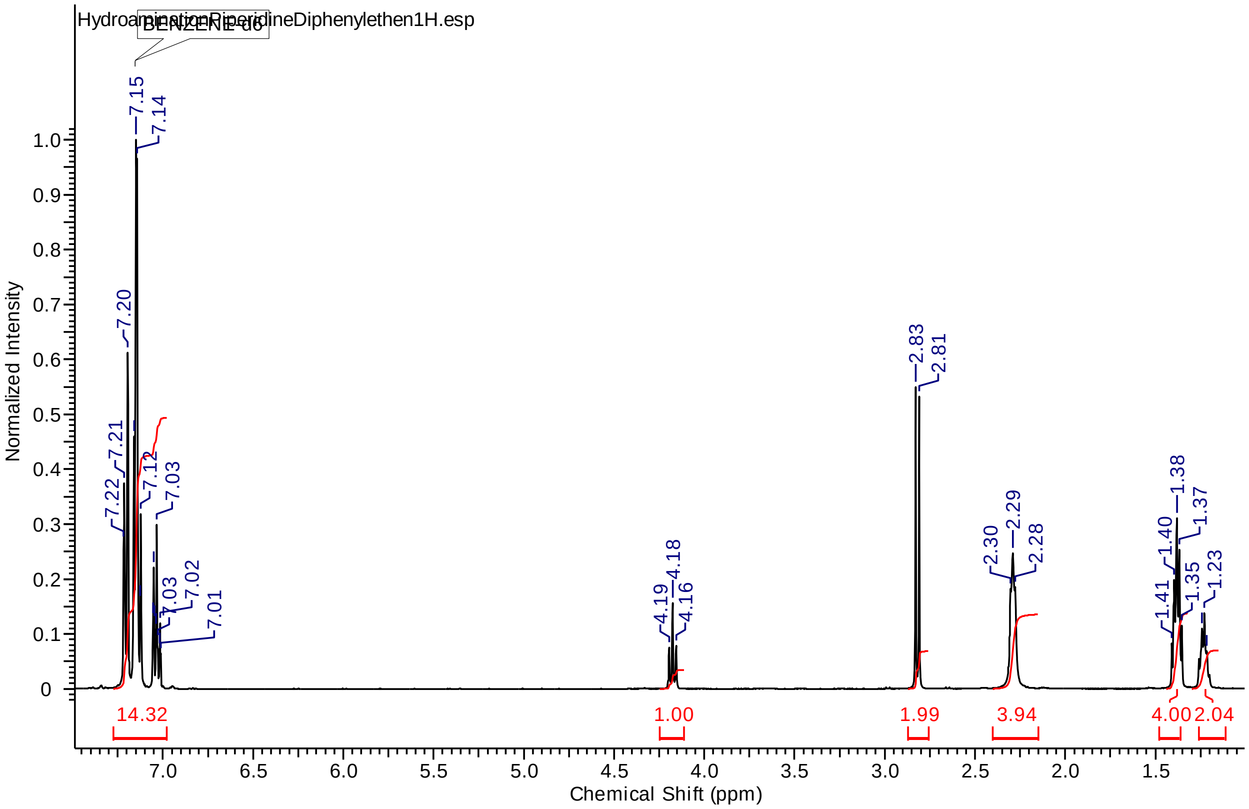

Figure S6 ^{1}H-NMR spectrum of **7a**.

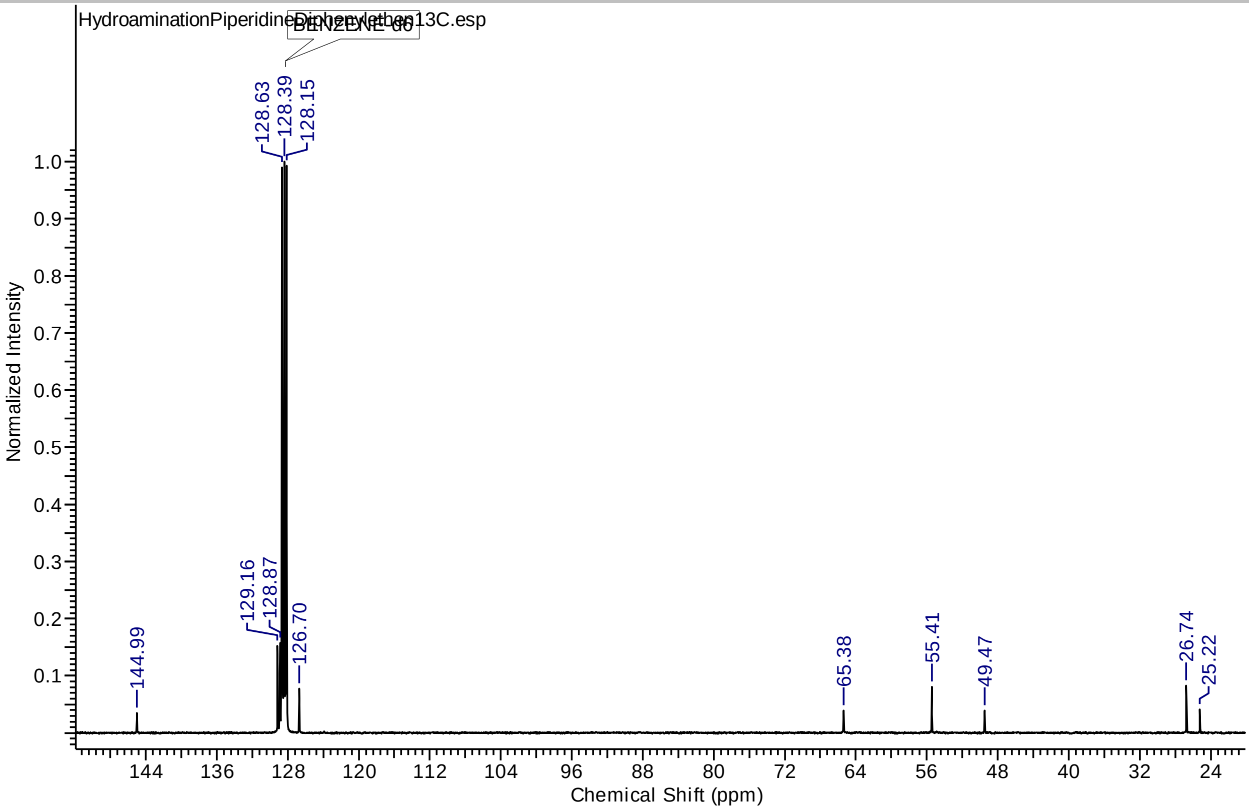

Figure S7 {^{1}H}^{13}C-NMR spectrum of **7a**.

2.3.1.1 Functionalization of 7a with methanol-d_4

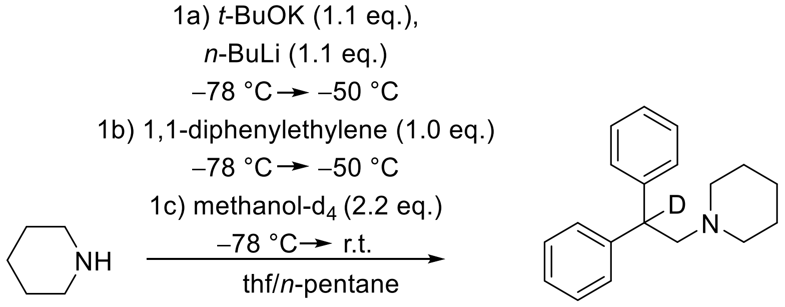

6 $C_5H_{11}N$ 85.15 g·mol^{-1} — **7b** $C_{19}H_{22}DN$ 266.41 g·mol^{-1}

To a stirred solution of piperidine (86 mg, 1.0 mmol, 1.0 eq.) and potassium-tert-butoxide (124 mg, 1.1 mmol, 1.1 eq.) in THF (2 ml) and *n*-pentane (2 ml) at –78 °C was added n-butyllithium (0.44 ml, 1.1 mmol, 1.1 eq.; 2.5 M in *n*-hexane). The reaction mixture was stirred for one hour to –50 °C. At –78 °C 1,1-diphenylethene (180 mg, 1.0 mmol, 1.0 eq.) was added. The reaction mixture was again stirred for one hour to –50 °C. At –78 °C methanol-d_4 (79 mg, 2.2 mmol, 2.2 eq.) was added and the reaction mixture was slowly stirred for 2 h to room temperature. After adding distilled water (15 ml), the organic layer was separated and the aqueous phase was extracted with diethylether (3 x 15 ml). The combined organic layers were dried with Na_2SO_4 and the solvent removed. The resulting yellow residue was purified by kugelrohr distillation (oven temperature: 160 °C, pressure: 0.39 mbar) yielding 0.212 mg (0.80 mmol, 80%) of the deuterated aminometalation product **7b**.

^{1}H-NMR (400.2 MHz, benzene-d_6): δ = 1.20–1.27 [m, 2H; $NCH_2CH_2CH_2$], 1.35–1.41 [m, 4H; $NCH_2CH_2CH_2$], 2.28–2.31 [m, 4H; $NCH_2CH_2CH_2$], 2.82 [d, ^3J(H,H) = 7.6 Hz, 2H; NCH_2CH], 4.18 [t, ^3J(H,H) = 7.6 Hz, 1H; NCH_2CH], 7.02–7.06 [m, 2H, H_{para}], 7.13–7.16 [m, 4, H_{meta}], 7.20–7.22 [m, 4H; H_{ortho}].

{^{1}H}^{13}C-NMR (176.1 MHz, benzene-d_6): δ = 25.2 [1C; $NCH_2CH_2CH_2$], 26.7 [2C; $NCH_2CH_2CH_2$], 49.5 [t, ^3J(C,D) = 19.4 Hz 1C; NCH_2CD], 55.4 [2C; $NCH_2CH_2CH_2$], 65.3 [1C; NCH_2CH], 126.7 [1C; C_{para}], 128.9 [2C; C_{meta}], 129.2 [2C; C_{ortho}], 145.0 [1C; C_{ipso}].

GC/EI-MS: (70 eV, t_R = 15.67 min): m/z (%): 265 (1) [M−H$^+$], 165 (6) [M−$C_6H_{13}DN^+$], 98 (100) [$C_6H_{12}N^+$], 77 (3) [$C_6H_5^+$].

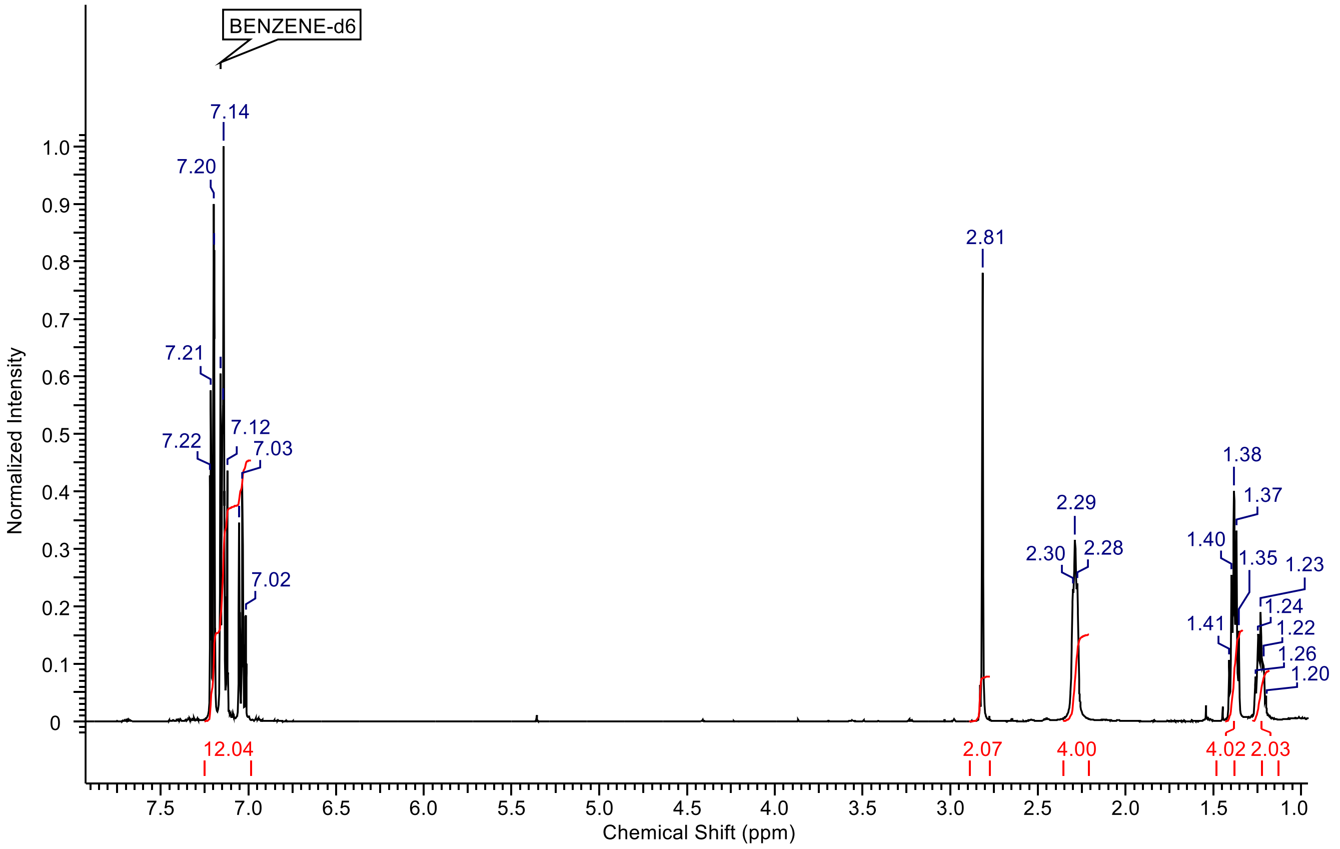

Figure S8 ^{1}H-NMR spectrum of **7b**

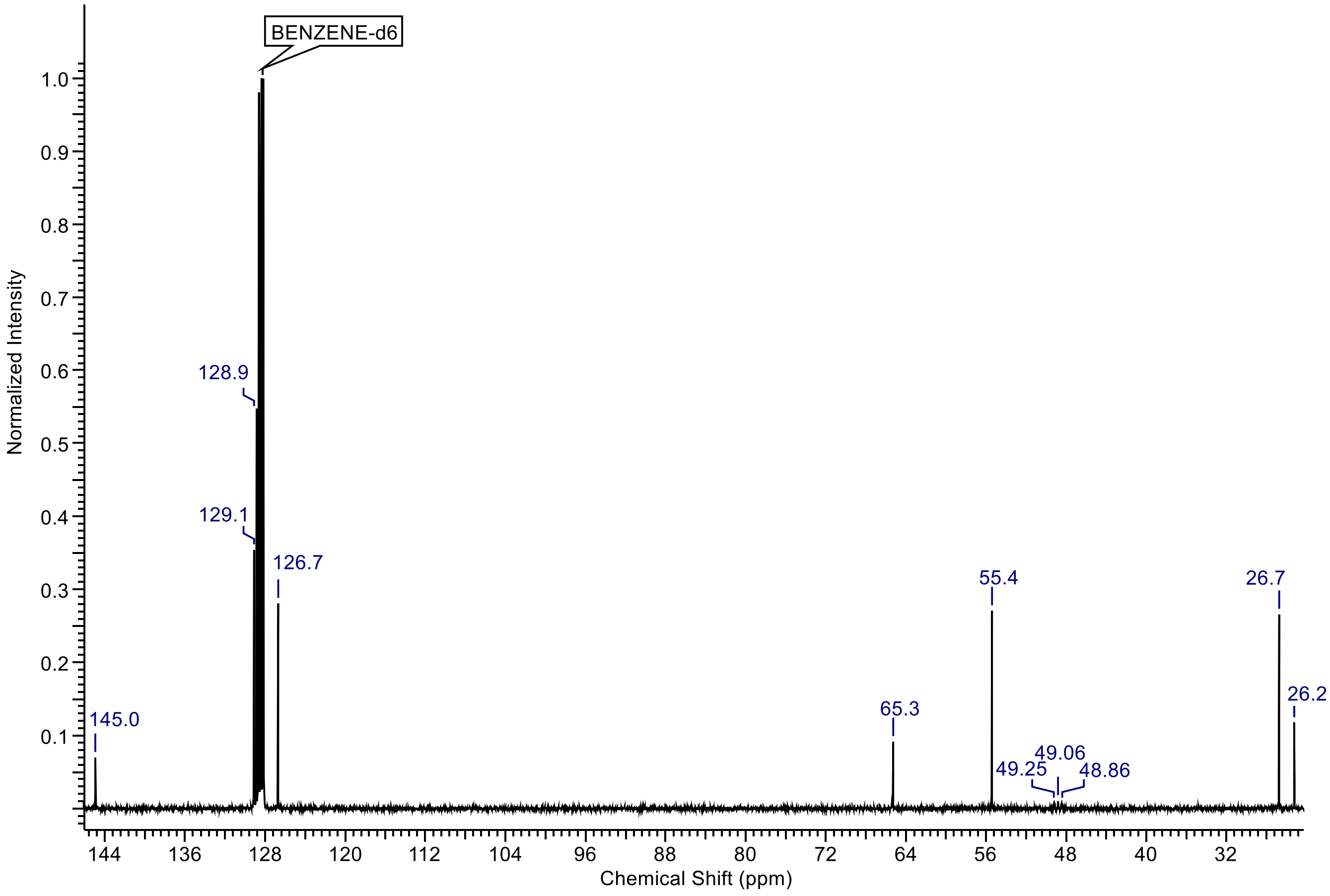

Figure S9 {^{1}H}^{13}C-NMR spectrum of **7b**.

2.3.1.2 Functionalization of 7a with *n*-Butylbromide

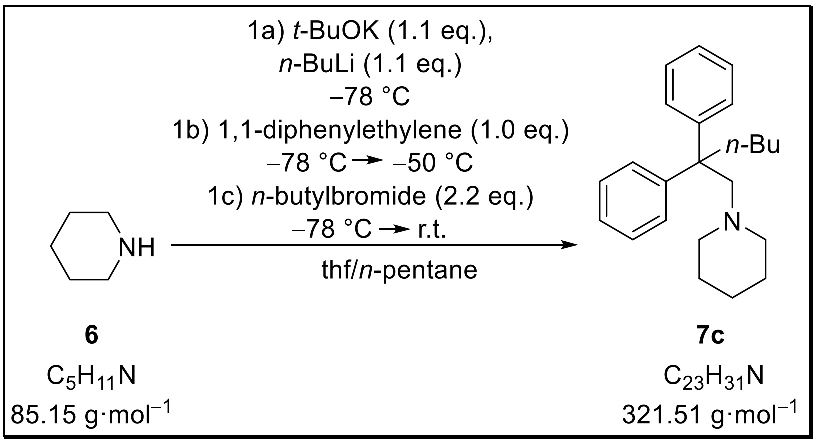

To a stirred solution of piperidine (86 mg, 1.0 mmol, 1.0 eq.) and potassium-tert-butoxide (124 mg, 1.1 mmol, 1.1 eq.) in THF (2 ml) and *n*-pentane (2 ml) at –78 °C was added n-butyllithium (0.44 ml, 1.1 mmol, 1.1 eq.; 2.5 M in *n*-hexane). The reaction mixture was stirred for one hour to –50 °C. At –78 °C 1,1-diphenylethene (180 mg, 1.0 mmol, 1.0 eq.) was added. The reaction mixture was again stirred for one hour to –50 °C. At –78 °C *n*-butylbromide (301 mg, 2.2 mmol, 2.2 eq.) was added and the reaction mixture was slowly stirred for 2 h to room temperature. After adding distilled water (15 ml), the organic layer was separated and the aqueous phase was extracted with diethylether (3 x 15 ml). The combined organic layers were dried with Na_2SO_4 and the solvent removed. The resulting yellow residue was purified by kugelrohr distillation (oven temperature: 160 °C, pressure: 0.39 mbar) yielding 0.244 mg (0.76 mmol, 76%) of the aminometalation product **7c**.

^{1}H-NMR (700.1 MHz, benzene-d$_6$): δ = 0.82 [t, ^3J(H,H) = 7.3 Hz, 3H, $CH_2CH_2CH_2CH_3$], 1.02–1.06 [m, 2H; $CH_2CH_2CH_2CH_3$], 1.20–1.25 [m, 2H; $NCH_2CH_2CH_2$], 1.29 [sxt, ^3J(H,H) = 7.3 Hz, 2H; $CH_2CH_2CH_2CH_3$], 1.35 [qin, ^3J(H,H) = 5.5 Hz, 4H; $NCH_2CH_2CH_2$], 2.06 [t, ^3J(H,H) = 5.5 Hz, 4H; $NCH_2CH_2CH_2$], 2.32-1.24 [m, 2H, $CH_2CH_2CH_2CH_3$], 2.93[s, 2H; CCH_2N], 7.03-7.06 [m, 2H; H_{ortho}], 7.12-7.15 [m, 4H; H_{meta}], 7.20-7.22 [m, 4H; H_{para}].

{^{1}H}^{13}C-NMR (176.1 MHz, benzene-d$_6$): δ = 14.6 [1C; $CCH_2CH_2CH_2CH_3$], 23.4 [1C; $CCH_2CH_2CH_2CH_3$], 24.1 [1C; $NCH_2CH_2CH_2$], 26.5 [2C; $NCH_2CH_2CH_2$], 26.7 [1C; $CCH_2CH_2CH_2CH_3$], 36.0 [1C; $CCH_2CH_2CH_2CH_3$], 50.5 [1C; CCH_2N], 56.6 [2C; $NCH_2CH_2CH_2$], 65.1 [1C, CCH_2N], 125.3 [2C; C_{para}], 127.4 [2C; C_{meta}], 128.33 [1C; C_{ortho}], 149.4 [1C; C_{para}].

**CHN Analysis:**	calculated:	C 85.92%	H 9.72%	N 10.1%
	found:	C 85.9%	H 10.0%	N 4.3%

GC/EI-MS: (70 eV, t_R = 17.57 min): m/z (%): 320 (1) [M–H$^+$], 264 (1) [M−$C_4H_9^+$], 222 (1) [M−$C_6H_{13}N^+$], 165 (17) [M–C_4H_9−$C_6H_{13}N^+$], 98 (100) [$C_6H_{12}N^+$], 91 (16) [$C_7H_7^+$].

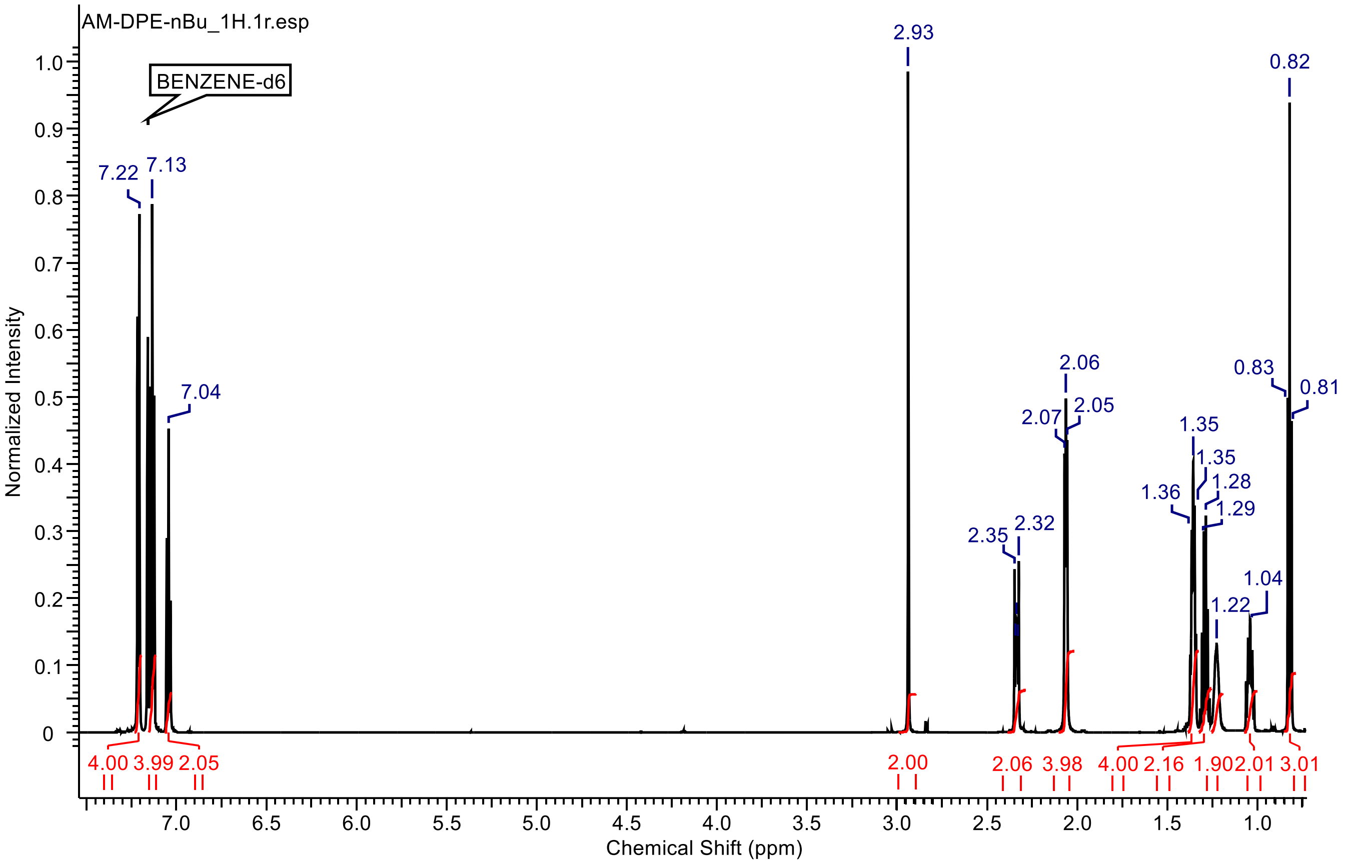

Figure S10 ^{1}H-NMR spectrum of **7c**.

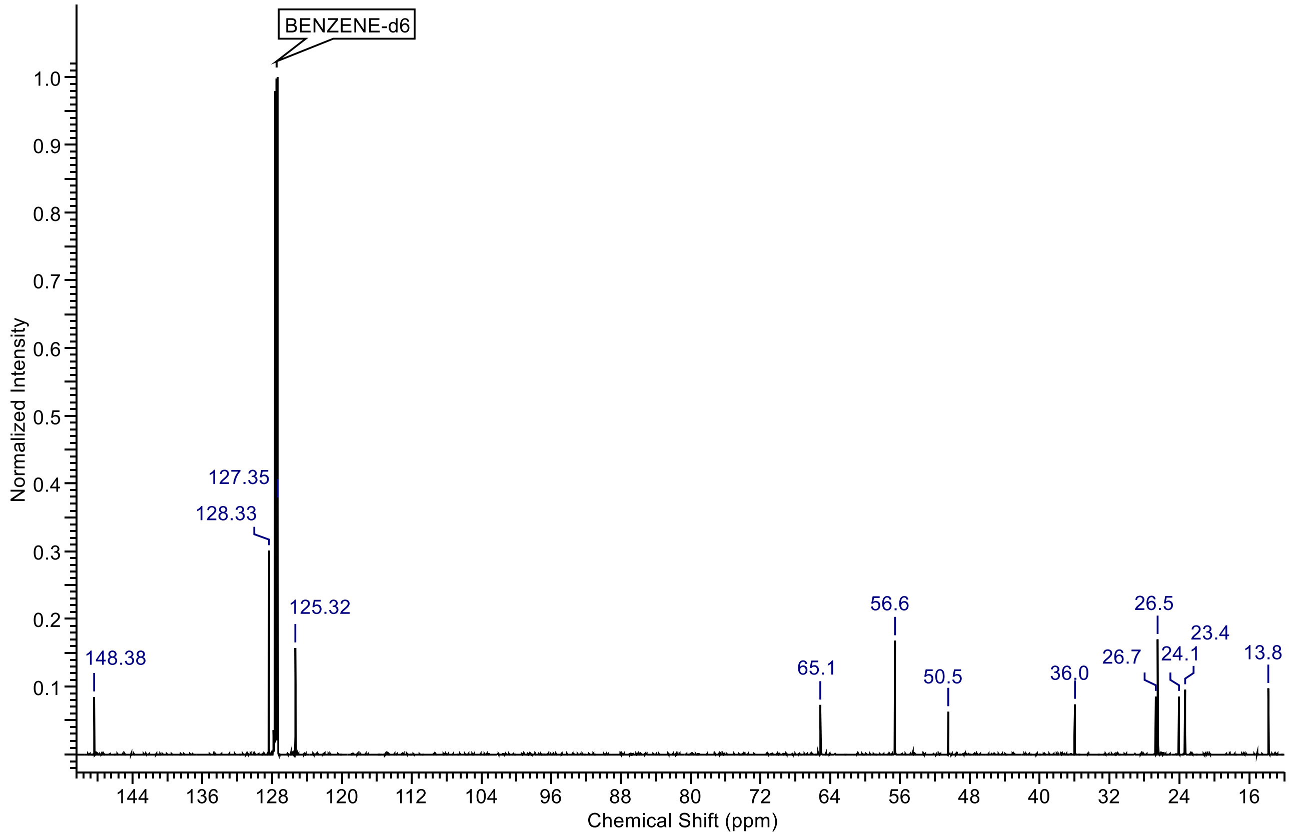

Figure S11 {^{1}H}^{13}C-NMR spectrum of **7c**.

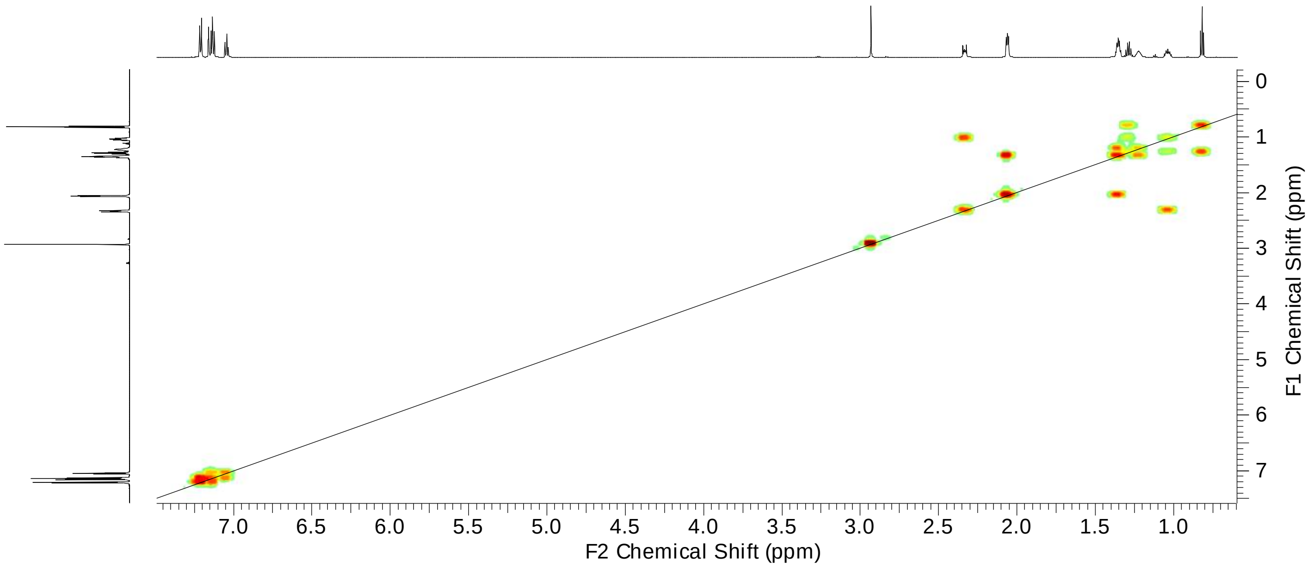

Figure S12 ^{1}H,^{1}H-COSY-NMR spectrum of **7c**.

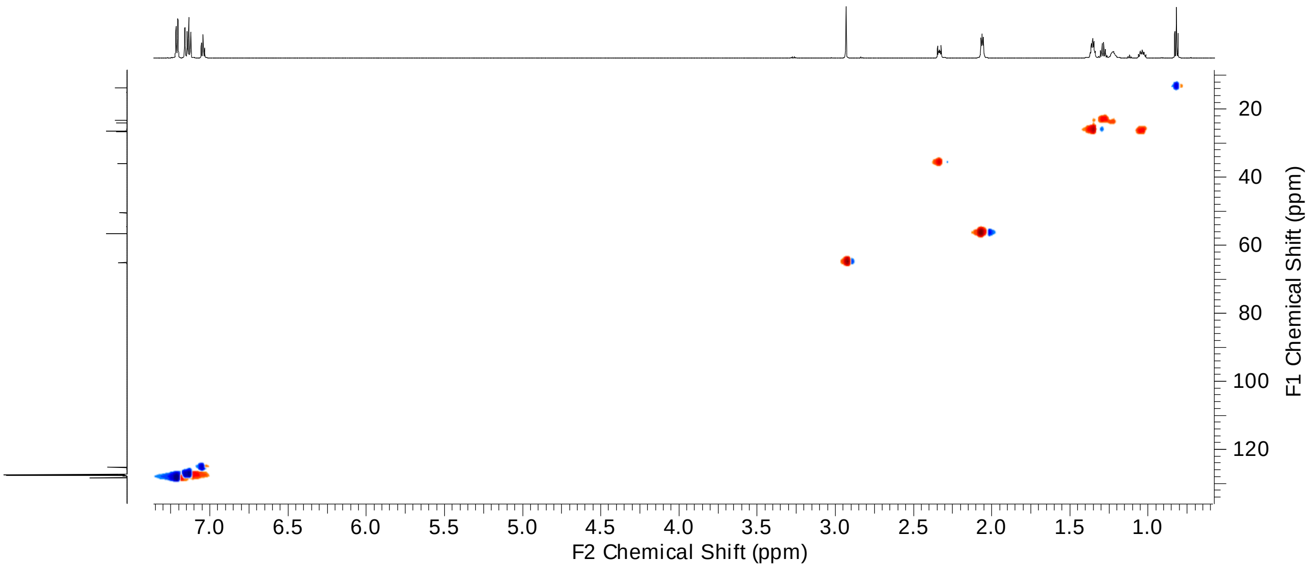

Figure S13 ^{1}H,^{13}C-HSQC-NMR spectrum of **7c**.

2.3.1.3 Functionalization of 7a with Chlor(trimethyl)silan

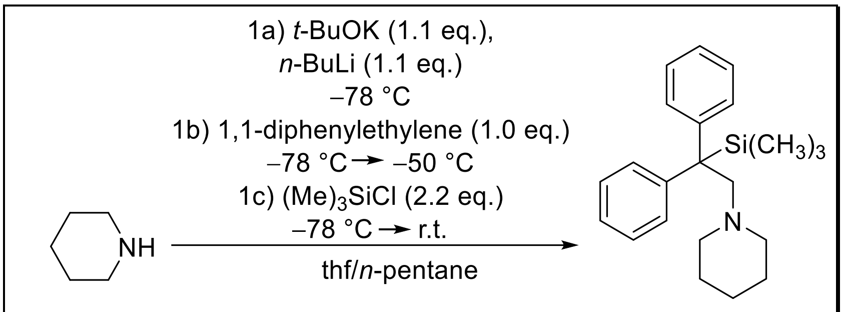

6 $C_5H_{11}N$ 85.15 g·mol^{-1} **7d** $C_{22}H_{31}NSi$ 337.58 g·mol^{-1}

To a stirred solution of piperidine (86 mg, 1.0 mmol, 1.0 eq.) and potassium-tert-butoxide (124 mg, 1.1 mmol, 1.1 eq.) in THF (2 ml) and *n*-pentane (2 ml) at –78 °C was added n-butyllithium (0.44 ml, 1.1 mmol, 1.1 eq.; 2.5 M in *n*-hexane). The reaction mixture was stirred for one hour to –50 °C. At –78 °C 1,1-diphenylethene (180 mg, 1.0 mmol, 1.0 eq.) was added. The reaction mixture was again stirred for one hour to –50 °C. At –78 °C chlor(trimethyl)silan (239 mg, 2.2 mmol, 2.2 eq.) was added and the reaction mixture was slowly stirred for 2 h to room temperature. After adding distilled water (15 ml), the organic layer was separated and the aqueous phase was extracted with diethylether (3 x 15 ml). The combined organic layers were dried with Na_2SO_4 and the solvent removed. The resulting yellow residue was purified by Kugelrohr distillation (oven temperature: 120 °C, pressure: 2.0 x 10^{-5} bar). Despite several attempts, a complete purification couldn't be achieved due to cleavage of the Si–C_{benzyl} bond and the formation of an azeotropic mixture. Nevertheless, compound **7d** could be characterized by NMR and GC/EI-MS.

^{1}H-NMR (400.2 MHz, benzene-d$_6$): δ = 0.19 [s, 9H, Si(C*H*$_3$)$_3$], 1.15–1.21 [m, 2H; NCH$_2$CH$_2$C*H*$_2$], 1.29–1.34 [m, 4H; NCH$_2$C*H*$_2$CH$_2$], 2.03–2.06 [m, 4H; NC*H*$_2$CH$_2$CH$_2$], 2.06 [s, 2H; NC*H*$_2$C], 7.01-7.22 [m, 10H; arom. *H*].

{^{1}H}^{13}C-NMR (100.6 MHz, benzene-d$_6$): δ = 0.4 [3C; Si(*C*H$_3$)$_3$] 24.9 [1C; NCH$_2$CH$_2$*C*H$_2$], 27.0 [2C; NCH$_2$*C*H$_2$CH$_2$], 47.4 [1C; NCH$_2$*C*], 57.6 [2C; N*C*H$_2$CH$_2$CH$_2$], 66.7 [1C; N*C*H$_2$C], 125.8 [2C; C_{para}], 128.25 [2C; C_{ortho}], 130.6 [1C; C_{meta}], 147.8 [1C; C_{ipso}].

{^{1}H}^{29}Si-NMR (79.5 MHz, benzene-d$_6$): δ = 4.1 [1Si; Si(CH$_3$)$_3$]

GC/EI-MS: (70 eV, t_R = 17.09 min): m/z (%): 322 (1) [M–Me$^+$], 264 (1) [M–SiMe$_3^+$], 237 (1) [M–$C_6H_{14}N^+$], 180 (4) [M–SiMe$_3$–$C_5H_{10}N^+$], 165 (6) [M–SiMe$_3$–$C_6H_{13}N^+$], 98 (100) [$C_6H_{12}N^+$], 73 (7) [SiMe$_3^+$].

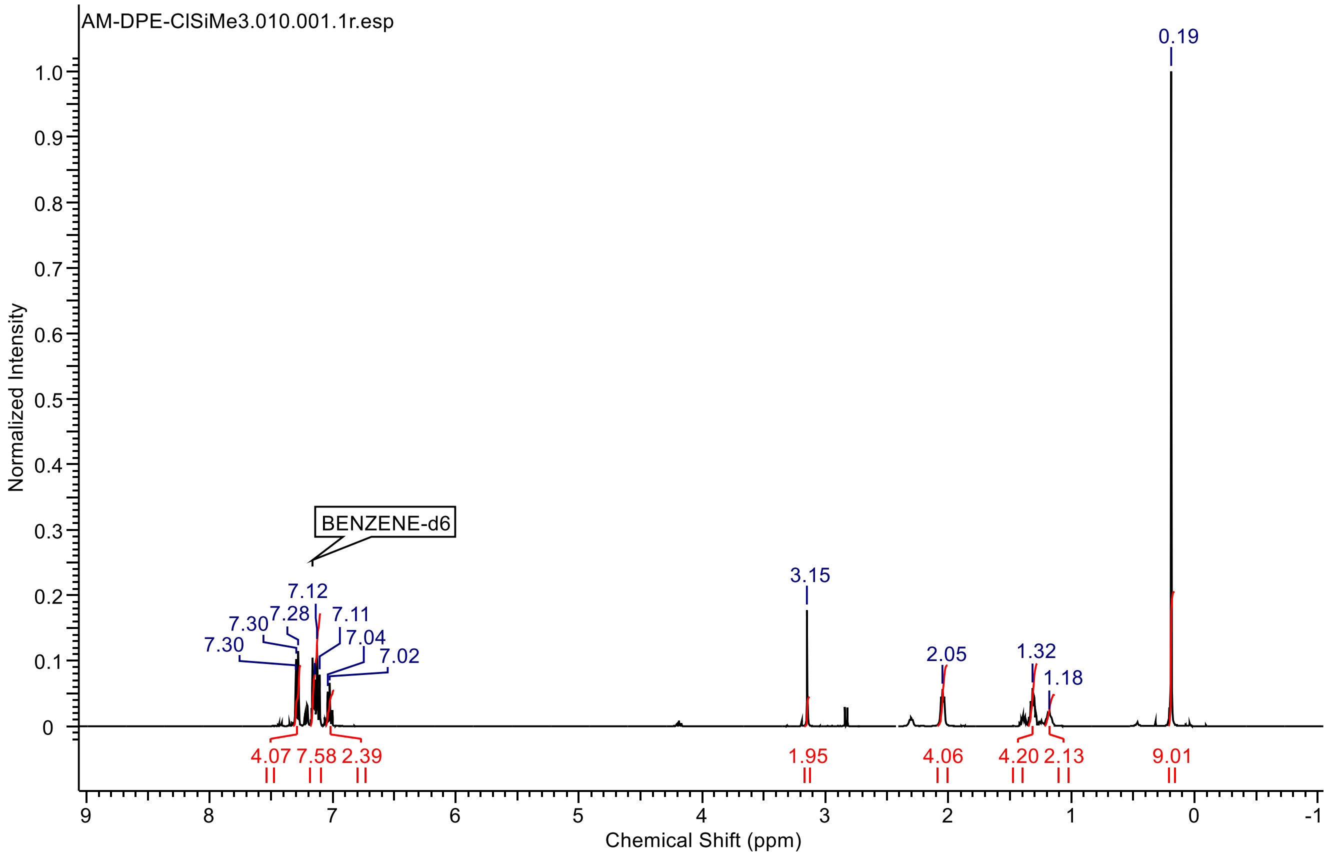

Figure S14 ^{1}H-NMR spectrum of **7d**.

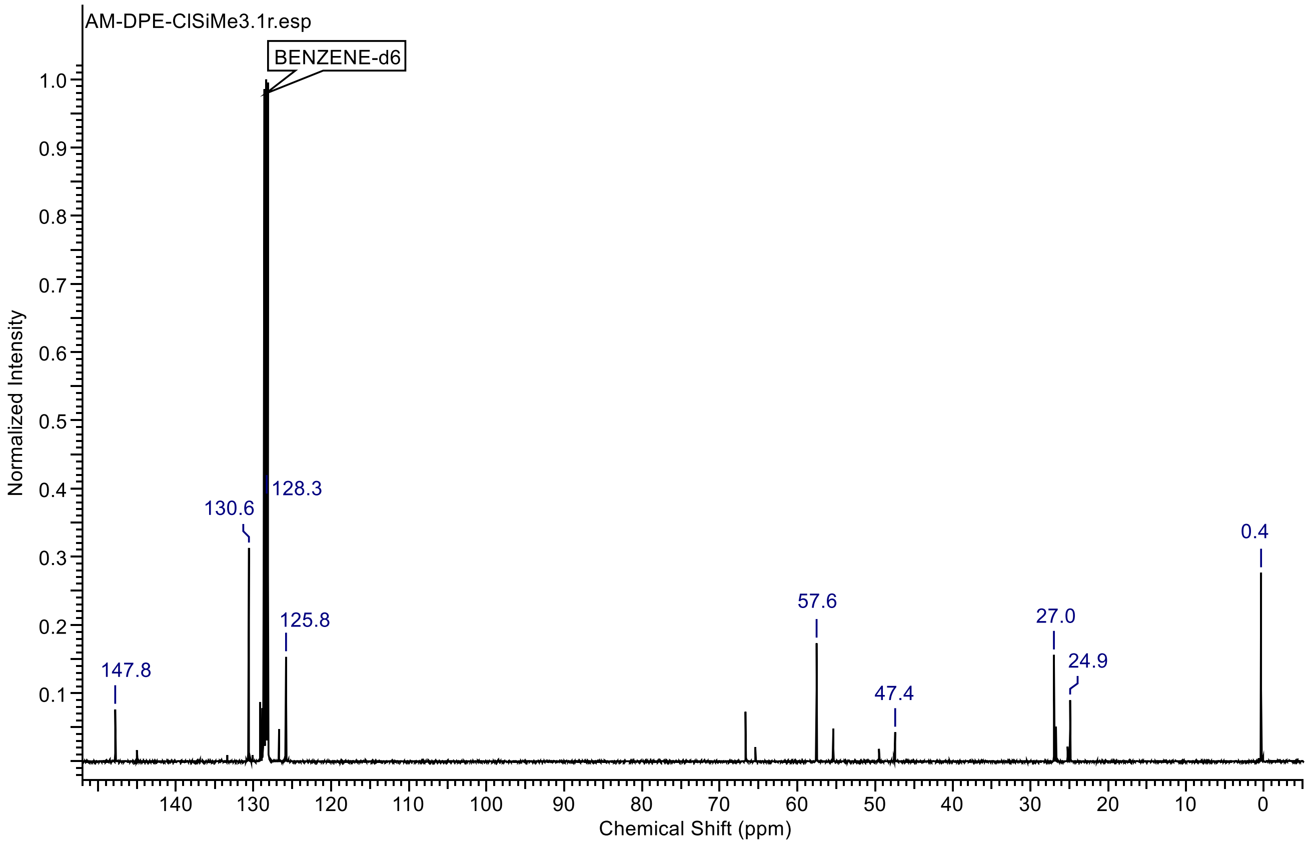

Figure S15 {^{1}H}^{13}C-NMR spectrum of **7d**.

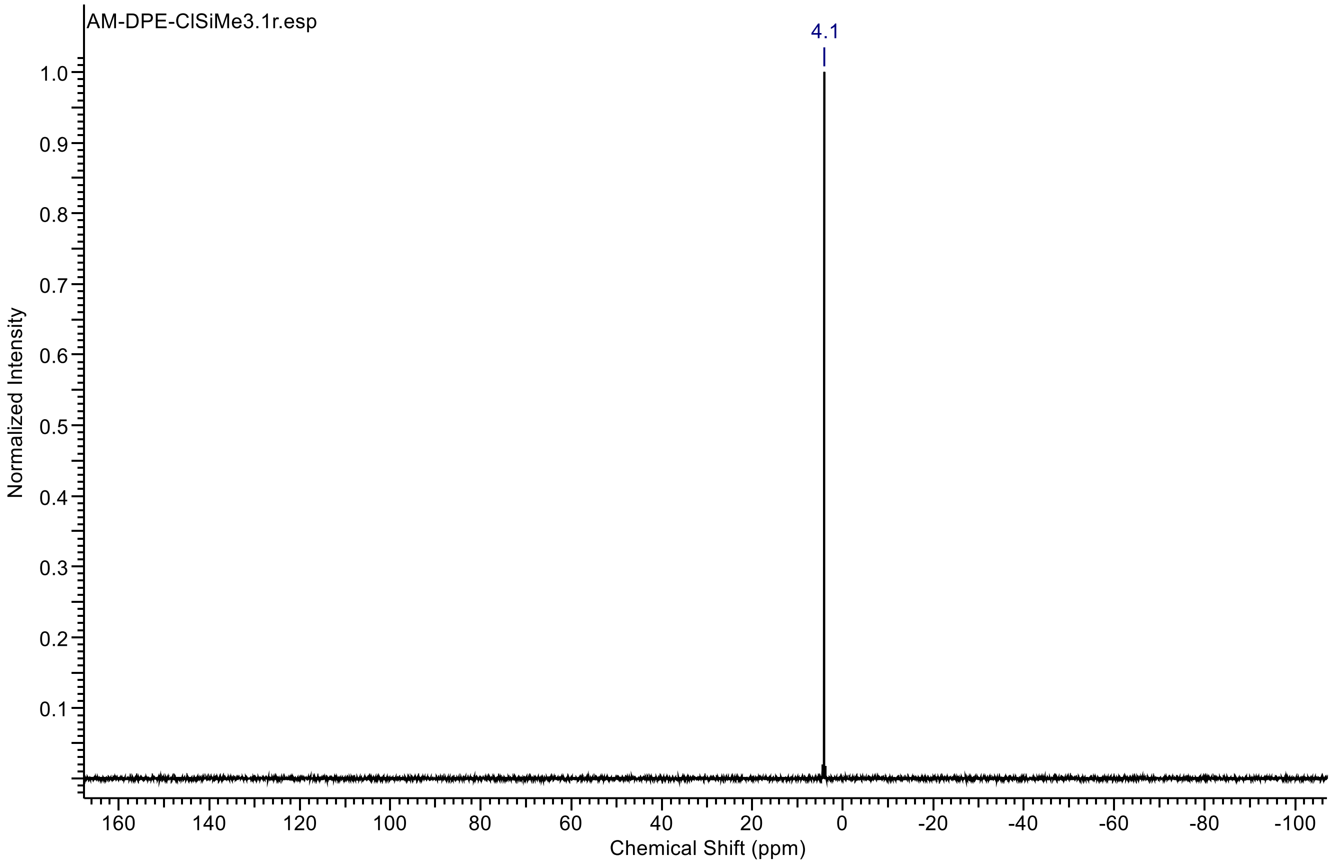

Figure S16 {^{1}H}^{29}Si-NMR spectrum of **7d**.

2.3.2 Aminometalation of Piperidine to 4-Methoxystyrene

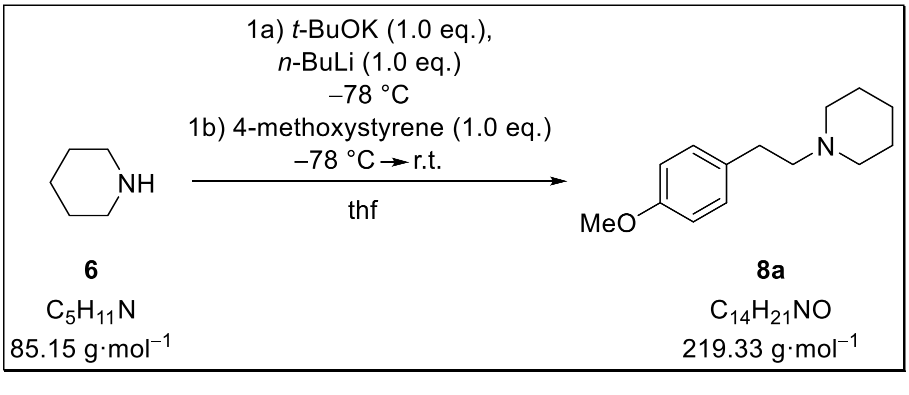

To a stirred solution of piperidine (172 mg, 2.0 mmol, 1.0 eq.) and potassium-tert-butoxide (224 mg, 2.0 mmol, 1.0 eq.) in THF (15 ml) at –78 °C was added n-butyllithium (0.8 ml, 2.0 mmol, 1.0 eq.; 2.5 M in hexane). The reaction mixture was stirred for one hour. At –78 °C 4-methoxystyrene (270 mg, 2.0 mmol, 1.0 eq.) was added. The reaction mixture was allowed to slowly warm to –60 °C and was stirred war 1.5 h at this temperature and 30 min at room temperature. After adding distilled water (15 ml), the organic layer was separated and the aqueous phase was extracted with diethylether (3 x 15 ml). The combined organic layers were dried with Na_2SO_4 and the solvent removed. The resulting yellow residue was purified by Kugelrohr distillation (oven temperature: 130 °C, pressure: 0.39 mbar) yielding 0.387 mg (1.76 mmol, 88%) of the aminometalation product **8a.**

^{1}H-NMR (700.1 MHz, benzene-d$_6$): δ = 1.30–1.37 [m, 2H; NCH$_2$CH$_2$C*H*$_2$], 1.52–1.55 [m, 4H; NCH$_2$C*H*$_2$CH$_2$], 2.27–2.36 [m, 4H; NC*H*$_2$CH$_2$CH$_2$], 2.49 [t, ^3J(H,H) = 7.6 Hz, 2H; NCH$_2$C*H*$_2$], 2.72 [t, ^3J(H,H) = 7.6 Hz, 2H; NC*H*$_2$CH$_2$], 3.34 [s, 3H; OC*H*$_3$], 6.81 [d, ^3J(H,H) = 8.4 Hz, 2H; arom. *H*], 7.05 [d, ^3J(H,H) = 8.4 Hz, 2H; arom. *H*].

{^{1}H}^{13}C-NMR (176.1 MHz, benzene-d$_6$): δ = 25.4 [1C; NCH$_2$CH$_2$*C*H$_2$], 26.9 [2C; NCH$_2$*C*H$_2$CH$_2$], 33.8 [1C; NCH$_2$*C*H$_2$], 55.1 [1C; O*C*H$_3$], 55.3 [2C; N*C*H$_2$CH$_2$CH$_2$], 62.3 [1C; N*C*H$_2$CH$_2$], 114.5 [2C; C_{para}], 130.3 [2C; C_{ortho}], 133.6 [1C; C_{meta}], 158.9 [1C; C_{ipso}].

CHN Analysis:

calculated:	C 76.67 %	H 9.65 %	N 6.39 %
found:	C 76.4 %	H 9.6 %	N 6.5 %

GC/EI-MS: (70 eV, t_R = 13.03 min): m/z (%): 219 (1) [M$^+$], 135 (6) [M-$C_5H_{10}N^+$], 121 (12) [M-$C_6H_{12}N^+$], 107 (1) [$C_7H_7O^+$], 98 (100) [$C_6H_{12}N^+$].

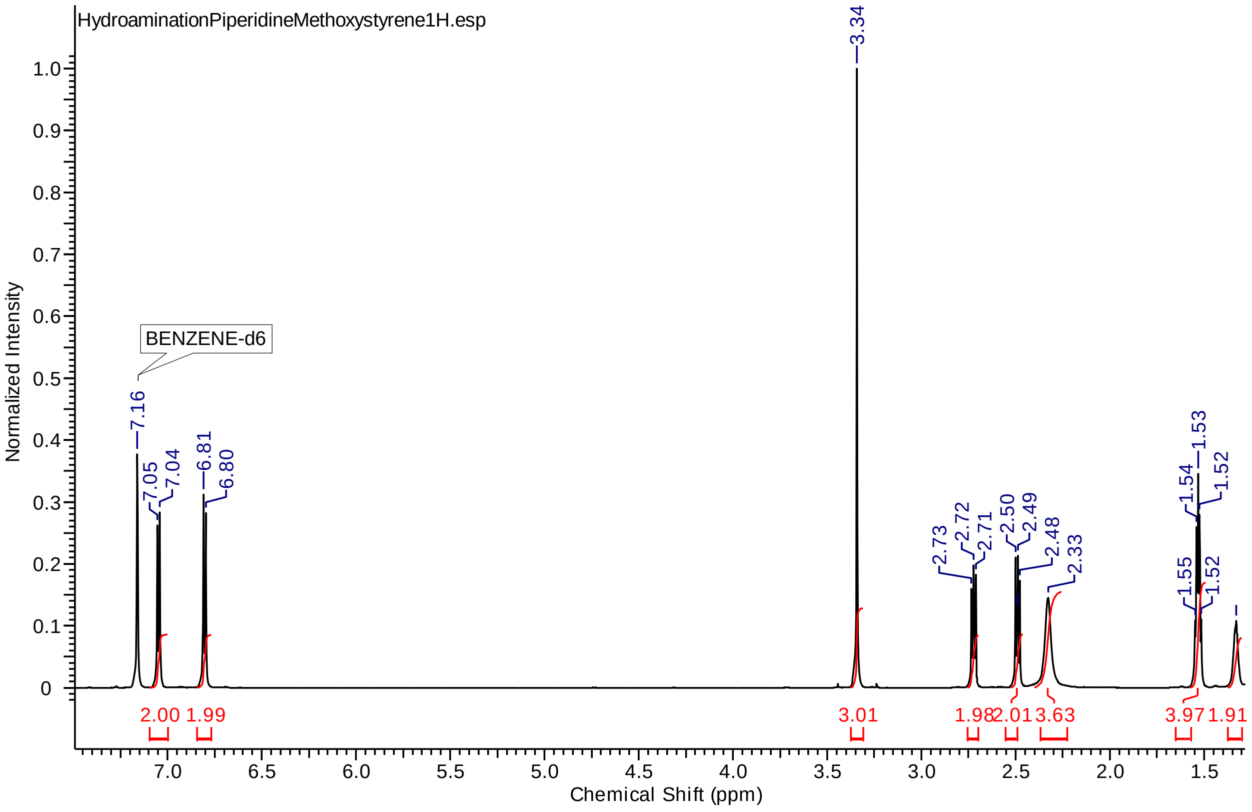

Figure S17 ^{1}H-NMR spectrum of **8a**.

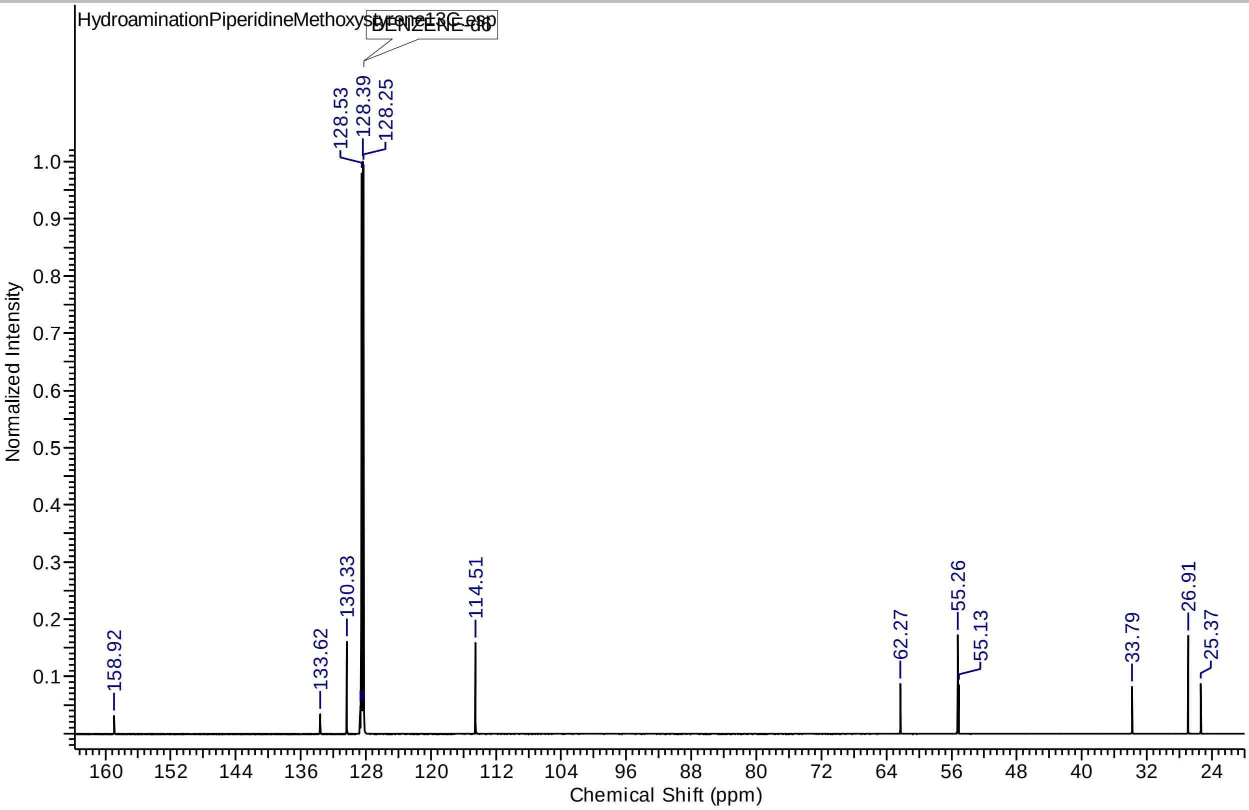

Figure S18 {¹H}^{13}C-NMR spectrum of **8a**.

2.3.2.1 Functionalization with Methanol-d_4

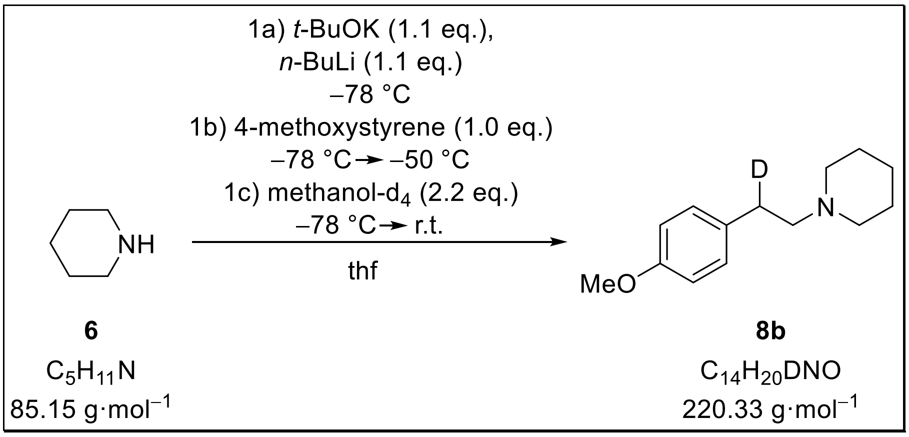

To a stirred solution of piperidine (85 mg, 1.0 mmol, 1.0 eq.) and potassium-tert-butoxide (124 mg, 1.1 mmol, 1.1 eq.) in THF (2 ml) and *n*-pentane (2 ml) at –78 °C was added n-butyllithium (0.44 ml, 1.1 mmol, 1.1 eq.; 2.5 M in *n*-hexane). The reaction mixture was stirred for one hour to –50 °C. At –78 °C 4-methoxystyrene (135 mg, 1.0 mmol, 1.0 eq.) was added. The reaction mixture was again stirred for one hour to –50 °C. At –78 °C methanol-d_4 (79 mg, 2.2 mmol, 2.2 eq.) was added and the reaction mixture was slowly stirred for 2 h to room temperature. After adding distilled water (15 ml), the organic layer was separated and the aqueous phase was extracted with diethylether (3 x 15 ml). The combined organic layers were dried with Na_2SO_4 and the solvent removed. Analysis of the residue with the GC-MS chromatography in combination with NMR spectroscopy showed mainly polymer and only a small amount of the undeuterated aminometalation product **8b** but no formation of the desired product.

2.3.2.2 Functionalization with *n*-Butylbromide

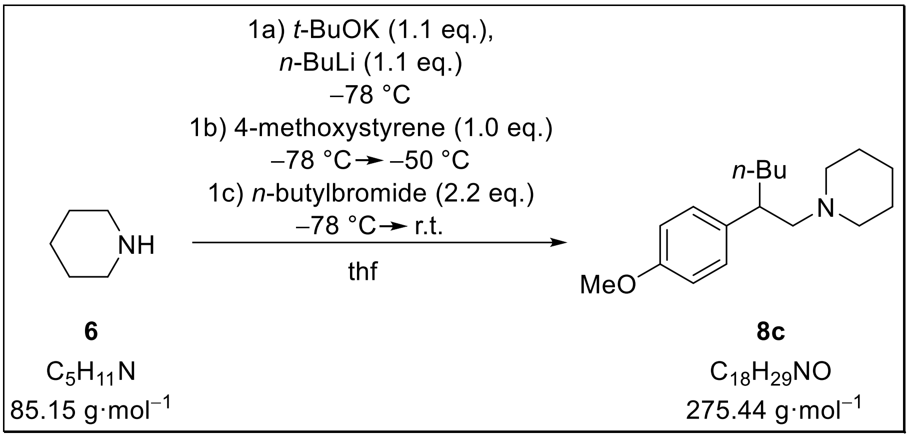

To a stirred solution of piperidine (86 mg, 1.0 mmol, 1.0 eq.) and potassium-tert-butoxide (124 mg, 1.1 mmol, 1.1 eq.) in THF (2 ml) and *n*-pentane (2 ml) at –78 °C was added n-butyllithium (0.44 ml, 1.1 mmol, 1.1 eq.; 2.5 M in *n*-hexane). The reaction mixture was stirred for one hour to –50 °C. At –78 °C 4-methoxystyrene (135 mg, 1.0 mmol, 1.0 eq.) was added. The reaction mixture was again stirred for one hour to –50 °C. At –78 °C *n*-butylbromide (79 mg, 2.2 mmol, 2.2 eq.) was added and the reaction mixture was slowly stirred for 2 h to room temperature. After adding distilled water (15 ml), the organic layer was separated and the aqueous phase was extracted with diethylether (3 x 15 ml). The combined organic layers were dried with Na_2SO_4 and the solvent removed. Analysis of the residue with the GC-MS chromatography in combination with NMR spectroscopy showed mainly polymer and only a small amount of the aminometalation prdoduct **8a** but no formation of the desired product **8c**.

2.3.2.3 Functionalization with Chlor(trimethyl)silan

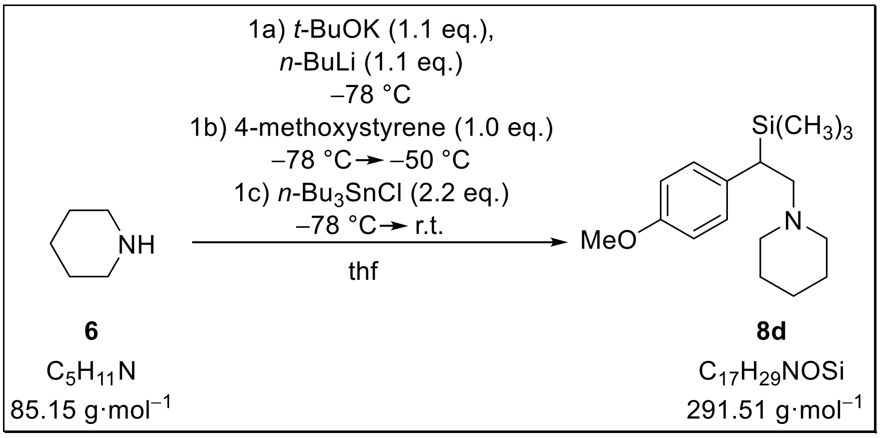

To a stirred solution of piperidine (86 mg, 1.0 mmol, 1.0 eq.) and potassium-tert-butoxide (124 mg, 1.1 mmol, 1.1 eq.) in THF (2 ml) and *n*-pentane (2 ml) at –78 °C was added n-butyllithium (0.44 ml, 1.1 mmol, 1.1 eq.; 2.5 M in *n*-hexane). The reaction mixture was stirred for one hour to –50 °C. At –78 °C 4-methoxystyrene (135 mg, 1.0 mmol, 1.0 eq.) was added. The reaction mixture was again stirred for one hour to –50 °C. At –78 °C tri-*n*-butyltin chloride (239 mg, 2.2 mmol, 2.2 eq.) was added and the reaction mixture was slowly stirred for 2 h to room temperature. After adding distilled water (15 ml), the organic layer was separated and the aqueous phase was extracted with diethylether (3 x 15 ml). The combined organic layers were dried with Na_2SO_4 and the solvent removed. Analysis of the residue with the GC-MS chromatography in combination with NMR spectroscopy showed

mainly polymer and only a small amount of the aminometalation product **8a** but no formation of the desired product **8d**.

2.3.2.4 Comparison of 4-Methoxystyrene with 1,1-Diphenylethene

For both styrene derivatives the subsequent functionalization of the aminometalated species don't work in pure THF. Only by adding *n*-pentane to the reaction mixture and only in the case of the a 1,1-diphenylethene a functionalization of the aminometalation product can be achieved. The potassiated 2,2-diphenylethyl-piperidine seems to form in contradiction to the 4-methoxystyrene an insoluble polymeric structure which precipitates out of the solution at around –50 °C and thereby preventing the metalated system to undergo an elimination process. Afterwards a functionalization is possible. For 4-methoxystyrene we showed that a deprotonation and a subsequent functionalization of *N,N*-dimethyl-4-methoxy-phenylethylamine is only possible in ortho-position to the methoxy group.[4] By slowly elevating the reaction temperature we also showed that an anion migration is happening at around –40 °C followed by an instantaneous elimination of the amide and isolation of 4-methoxystyrene.

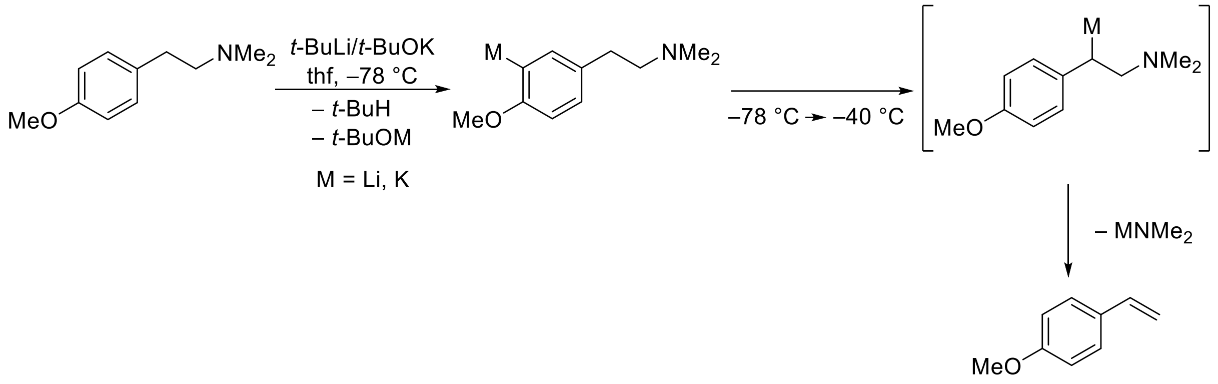

Scheme S1 Process of the anion migration in metalated 4-methoxystyrene.

A similar observation was made for potassiated *N,N*-dimethyl-phenylethylamine where the elimination process is happening at –30 °C. Furthermore, calculations of the anionic 4-methoxystyrene indicating that the benzylic anion is less stable in comparison to styrene. In summary these results are showing that the electronic properties of the 4-methoxystyrene are favoring an elimination and thereby making a functionalization of the methoxy substituted derivative more difficult.

The addition of stoichiometric amounts of deuterated water did not result in a deuterated product. That the aminometalation works when no further electrophile is used indicates that the aminometalated species abstracts a proton somewhere. By carrying out the reaction in THF-d_8 a possible deprotonation of the solvent should be possible to detect by NMR-spectroscopy but for both styrene derivatives no deuterated products could be found.

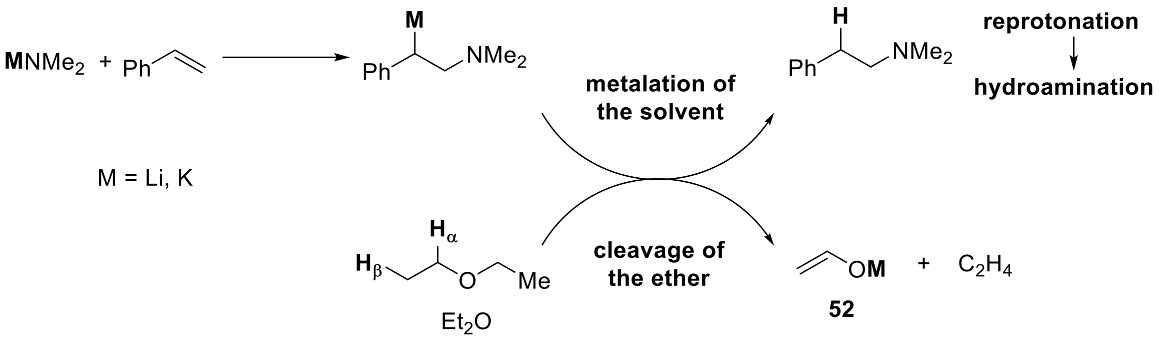

Scheme S2 Possible deprotonation of solvent by the metalated species.

3 Single-Crystal X-ray Diffractional Analysis

Crystallographic data to all determined structures have been deposited at the CCDC (Cambridge Crystallographic Data Centre). These data can be obtained free of charge from the CCDC, Union Road, Cambridge, CB2 IEZ, UK (Fax: +44-1223-336033; e-mail: deposit@ccdc.cam.ac.uk or www: http://www.ccdc.cam.ac.uk). CCDC deposition numbers listed below.

Table S1: CCDC deposition numbers for the crystallized compounds.

Compound	CCDC deposition number
**[4a·2THF]$_2$**	CCDC 2013446
**[1b·4THF]**	CCDC 2013450
**[1b·2THF]$_\infty$**	CCDC 2013448
**9**	CCDC 2013447
**10**	CCDC2013449

3.1 Synthesis of Lithiated Compound [4a·2THF]$_2$

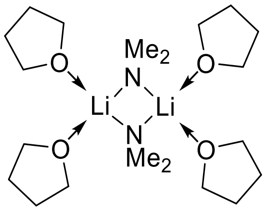

[4a·2THF]$_2$

$C_{20}H_{44}Li_2N_2O_4$

390.46 g·mol^{-1}

To a stirred solution of *N*,*N*-Dimethyl-2-trimethylsilyl-2-phenyl-ethyl-1-amin (0.220 g, 1.0 mmol) in *n*-pentane (0.5 ml) and thf (0.5 ml) at –78 °C *tert*-butyllithium (0.35 ml, 1.0 mmol, 1.0 eq.; 1.9 M in *n*-pentane) was added. The reaction mixture was stored at –30 °C for 1 day and afterwards stored at –78 °C. After 2 d homogeneous crystals (colourless blocks) of **[4a·2THF]$_2$** formed, suitable for X-ray structural analysis. By washing the obtained crystals with toluene and *n*-pentane and dissolving them in THF-d$_8$ the following NMR-spectra were obtained. A yield could not be obtained. Due to weak bonding of the thf molecules the crystals were not weight stable.

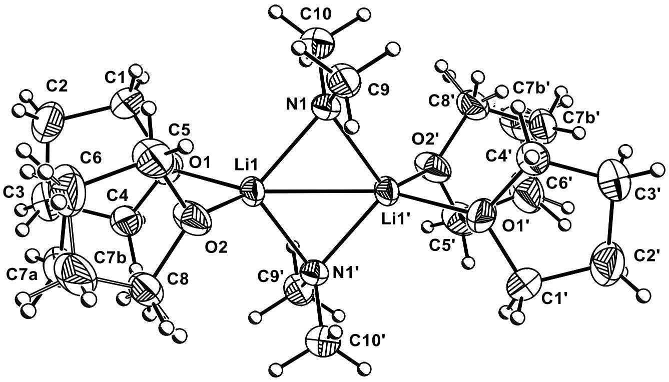

Figure S19 Ortep plot of the molecular structure of **[4a·2THF]$_2$** in the crystal, with the displacement ellipsoids drawn at the 50% probability level.[4] Numbering scheme of hydrogen atoms omitted for clarity. (Symmetry code: i = −1 −x, −y, −z) Selected bond lengths [Å] and angles [°]: Li1−N1 1.996(4), Li1−N1^i 2.007(4), Li1−O1 2.025(4), Li1−O2 2.018(4), O1−Li1−O2 100.78, O1−Li1−N1 115.14, O2−Li1−N1 110.80, N1−Li1−N1^i 105.01.

^{1}H-NMR (400.1 MHz, THF-d_8): δ = 4.54 [s, 6H; N(CH_3)$_2$]

{^{1}H}^{13}C-NMR (100.64 MHz, THF-d_8): δ = 48.4 [2C; N(CH_3)$_2$].

^{7}Li-NMR (155.5 MHz, THF-d_8): δ = 0.61

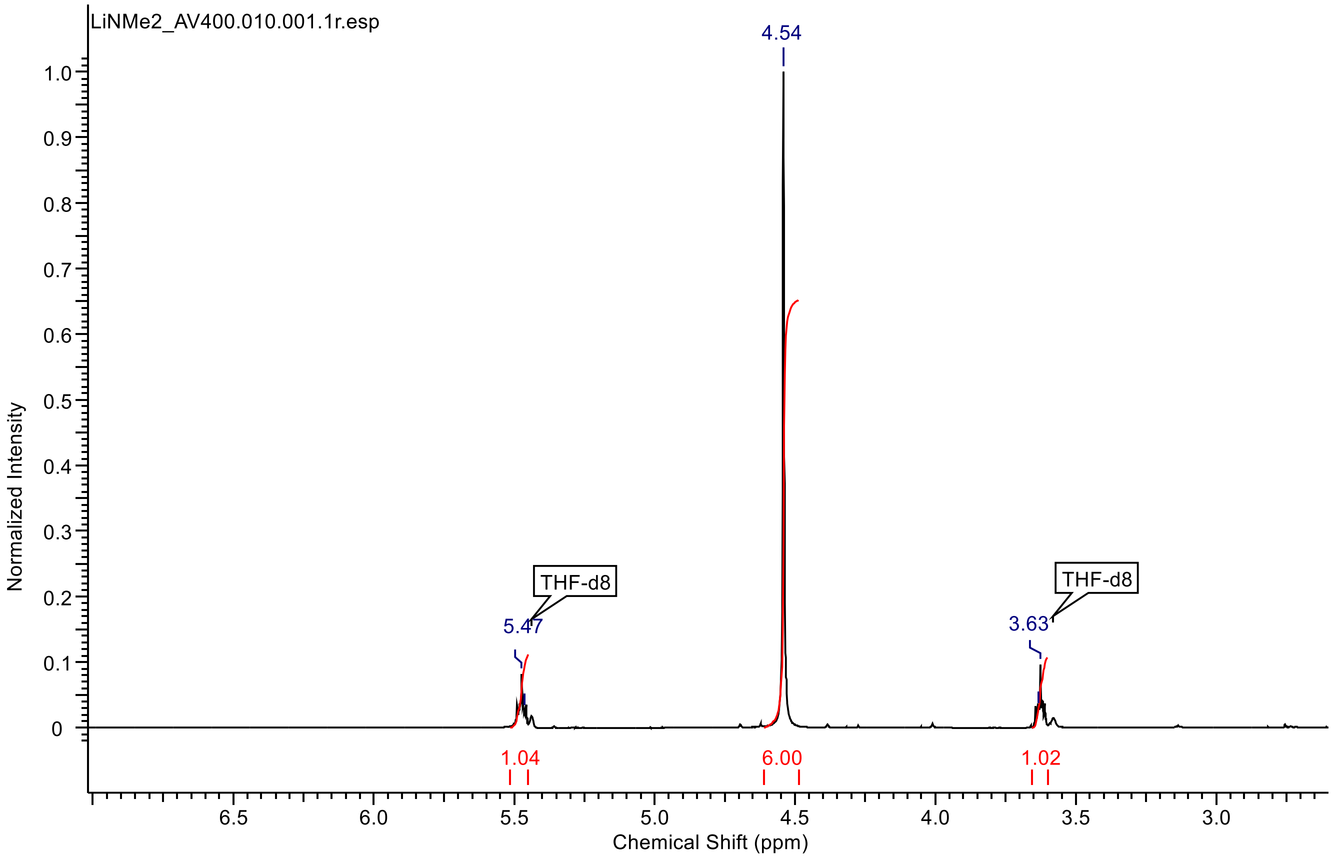

Figure S20 ^{1}H-NMR spectrum of **[4a·2THF]$_2$.**

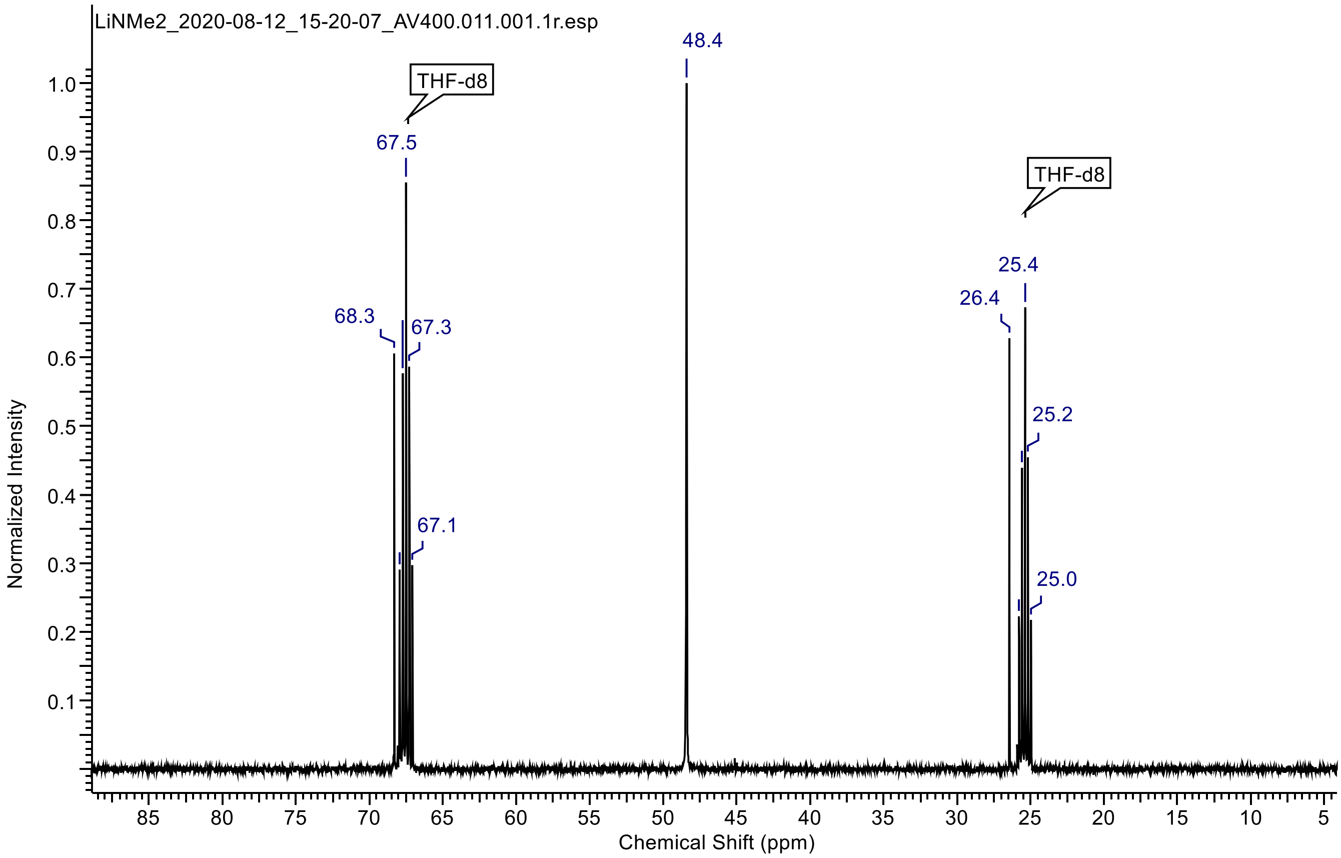

Figure S21 ^{13}C-NMR spectrum of **[4a·2THF]$_2$**.

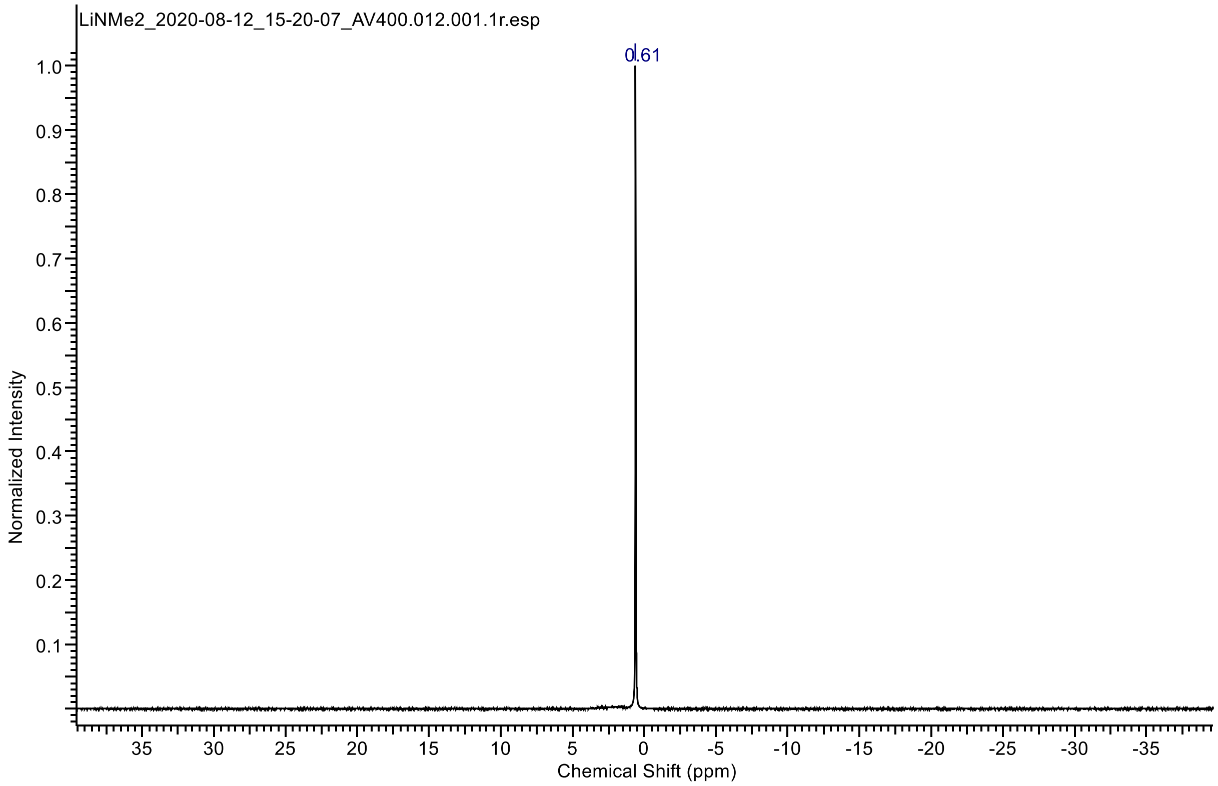

Figure S22 ^{7}Li-NMR spectrum of **[4a·2THF]$_2$**.

3.2 Synthesis of Potassiated Compound [1b·4THF]

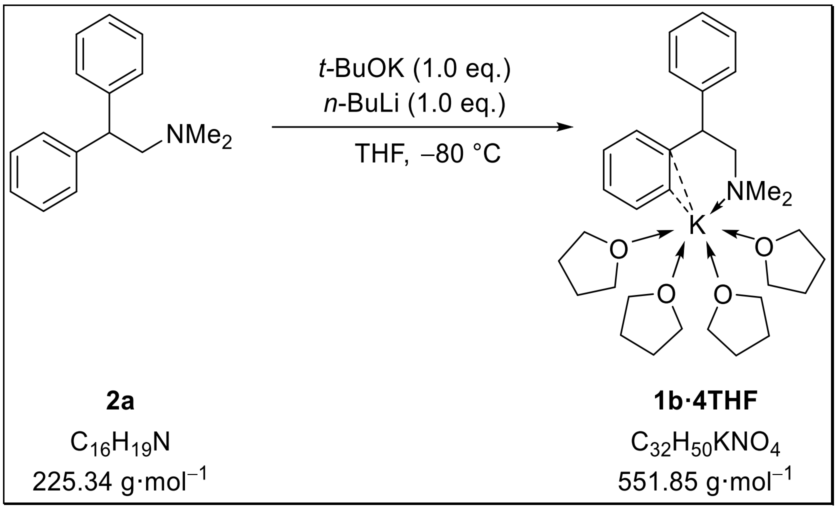

To a stirred solution of *N*,*N*-Dimethyl-2,2-diphenyl-ethyl-1-amin (0.225 g, 1.0 mmol, 1.0 eq.) and potassium-*tert*-butoxide (112 mg, 1.0 mmol, 1.0 eq.) in THF (1 ml) at –78 °C *n*-butyllithium (0.4 ml, 1.0 mmol, 1.0 eq.; 2.5 M in hexane) was added. After a few minutes red crystals of compound [**1b·4THF]** formed, suitable for X-ray structural analysis. No yield or NMR-spectra could be obtained. Due to weak bonding of the thf molecules the crystals were not weight stable, indicating that a polymerization to compound **[1b·2THF]$_\infty$** is occurring while trying to remove the solvent.

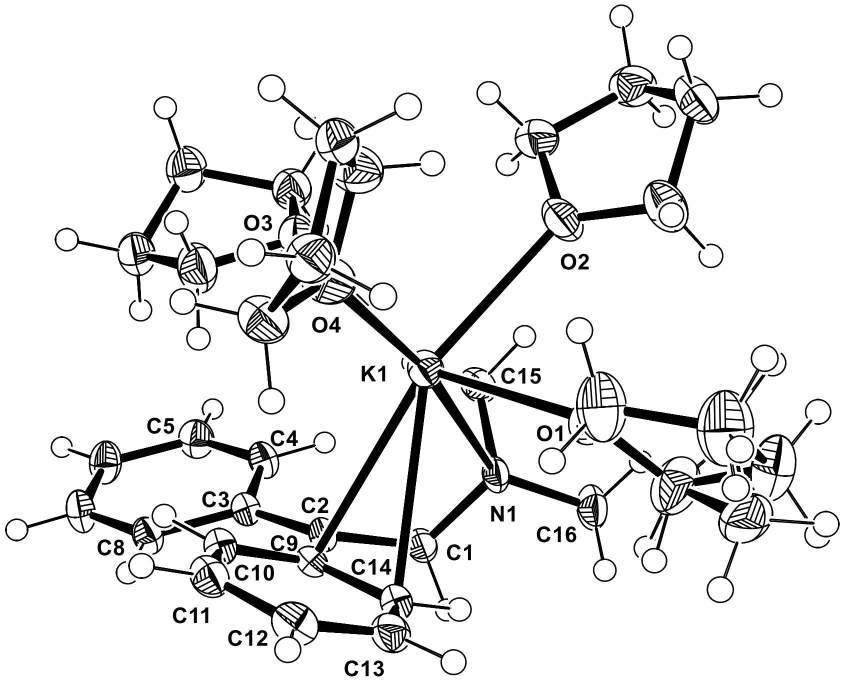

Figure S23 Ortep plot of the molecular structure of **[1b·4THF]** in the crystal, with the displacement ellipsoids drawn at the 50% probability level.[4] Numbering scheme of hydrogen atoms omitted for clarity. Selected bond lengths [Å] and angles [°]: K–O1 2.7175(18), K1–O2 2.7366(17), K1–O3 2.829(2), K1–O4 2.6538(17), K1–N1 2.9065(19), K1–C2 3.389(2), K1–C9 3.065(2), K–C14 3.097(2), K1–C15 3.110(2), N1–C1 1.490(2), N–C1 1.490(2), C1–C2 1.512(2), C2–C3 1.450(3), C2–C9 1.433(3), C3–C4 1.426(3), C4–C5 1.384(3), C3–C8 1.428(2), C9–C14 1.440(2), C13–C14 1.383(3), C1–C2–C3 118.08(16), C3–C2–C9 125.21(16), C1–C2–C9 116.37(15), C8–C3–C2–C9 –22.5(3), C9–C2–C3–C4 160.26(18), C2–C3–C8–C7 179.26(19), C2–C9–C10–C11 179.97(18).

3.3 Synthesis of Potassiated Compound [1b·2THF]$_\infty$

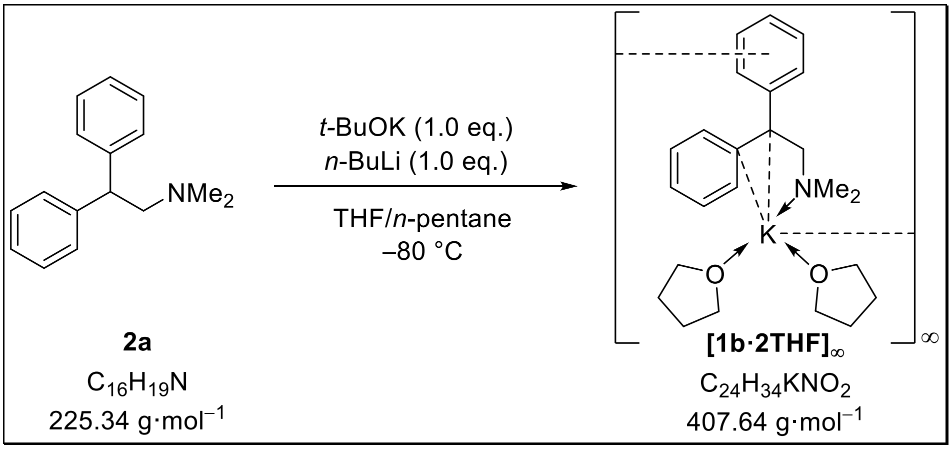

2a	[1b·2THF]$_\infty$
$C_{16}H_{19}N$	$C_{24}H_{34}KNO_2$
225.34 g·mol^{-1}	407.64 g·mol^{-1}

To a stirred solution of *N*,*N*-Dimethyl-2,2-diphenyl-ethyl-1-amin (225 mg, 1.0 mmol, 1.0 eq.) and potassium-*tert*-butoxide (112 mg, 1.0 mmol, 1.0 eq.) in THF (1 ml) at –78 °C *n*-butyllithium (0.4 ml, 1.0 mmol. 1.0 eq.; 2.5 M in hexane) was added. After 48 h all volatile components have been removed by applying a short vacuum and *n*-pentane has been added to the reaction mixture. After 12 h at –78 °C red crystals of compound **[1b·2THF]$_\infty$** formed, suitable for X-ray structural analysis. Another possible method is to use the crystals of the compound **[1b·4THF]**, bring them onto the microscope slide and warm up the X-TEMP to –20 °C. Thereby the crystals dissolve in the perfluorinated oil and recrystallize with two instead of four THFs in the polymeric structure **[1b·2THF]$_\infty$** at –60 °C. A yield could not be obtained. Due to weak bonding of the thf molecules the crystals were not weight stable. Furthermore, by keeping the red crystals for a longer period in vacuum to properly remove the solvent a decomposition of the crystals could be observed. Because of their high sensitivity a complete removal of the washing solvents was not possible leading to large impurities in the NMR spectra. Nevertheless, the most characteristic signals could be characterized, like the highly shifted para protons of the phenyl rings in the ^{1}H-NMR spectrum.

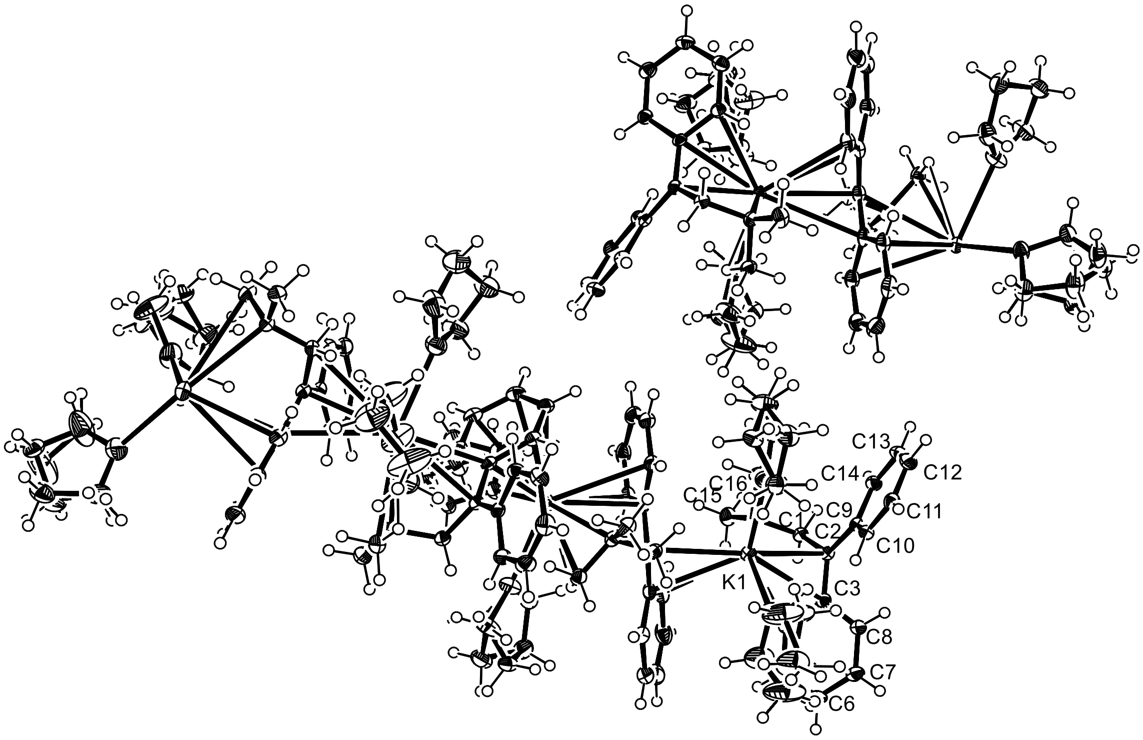

Figure S24 Ortep plot of the molecular structure of **[1b·2THF]$_\infty$** in the crystal, with the displacement ellipsoids drawn at the 50% probability level.[4] Numbering scheme of hydrogen atoms omitted for clarity. Selected bond lengths [Å] and angles [°]: K–O1 2.7063(16), K–O2 2.7046(16), K–N1 2.8954(17), K–C2 3.014(2), K–C3 3.100(2), K–C4 3.221(2), K–C16 3.161(2), K–C18 2.971(2), K–C19 3.109(2), K–C20 3.275(2), N1–C1 1.49(2), C1–C2 1.504(3), C2–C3 1.428(3), C2–C9 1.447(3), C3–C4 1.431(3), C4–C5 1.384(3), C3–C8 1.432(3), C9–C10 1.419(3), C9–C14 1.415(3), C13–C14 1.382(3), C1–C2–C3 116.48(17), C3–C2–C9 123.17(17), C1–C2–C9 120.34(17), C3–C2–C9–C10 −28.7(3), C3–C2–C9–C14 154.84(19), C2–C9–C14–C13 175.89(19), C2–C3–C4–C5 175.67(19).

^{1}H-NMR (400.1 MHz, THF-d_8): δ = 2.14 [s, 6H; N(CH_3)$_2$], 3.26 [s, 2H; CKCH_2], 6.10 [m, 2H; H_{para}], 6.83 [m, 4H; H_{meta}], 7.11 [m, 4H; H_{ortho}]

{^{1}H}^{13}C-NMR (100.64 MHz, THF-d_8): δ = 63.5 [1C, CKCH$_2$],117.2 [4C; C_{para}], 129.0 [4C; C_{ortho}], 129.8 [4C; C_{meta}], 146.9 [2C; C_{ipso}].

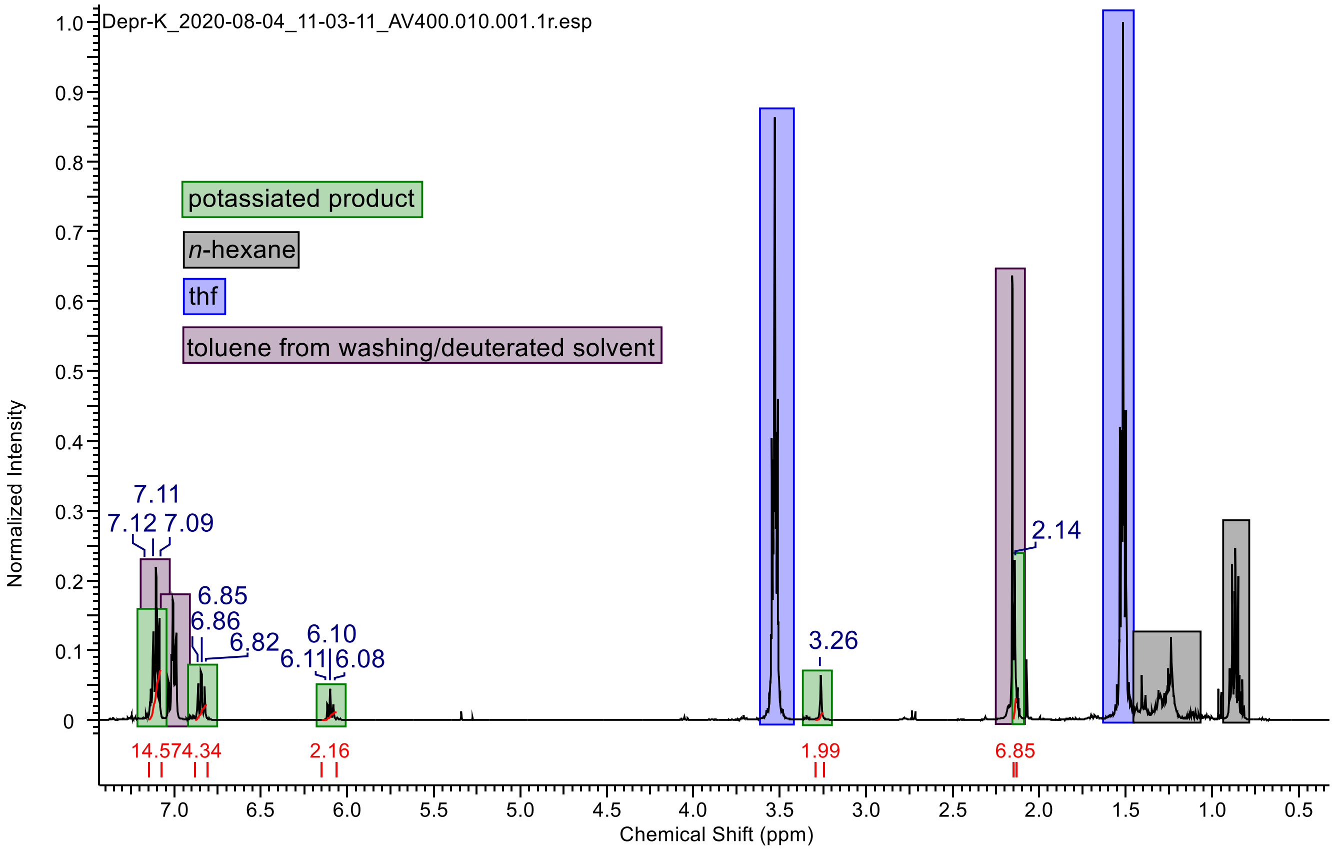

Figure S25 ¹H-NMR spectrum of **[1b·2THF]**$_\infty$.

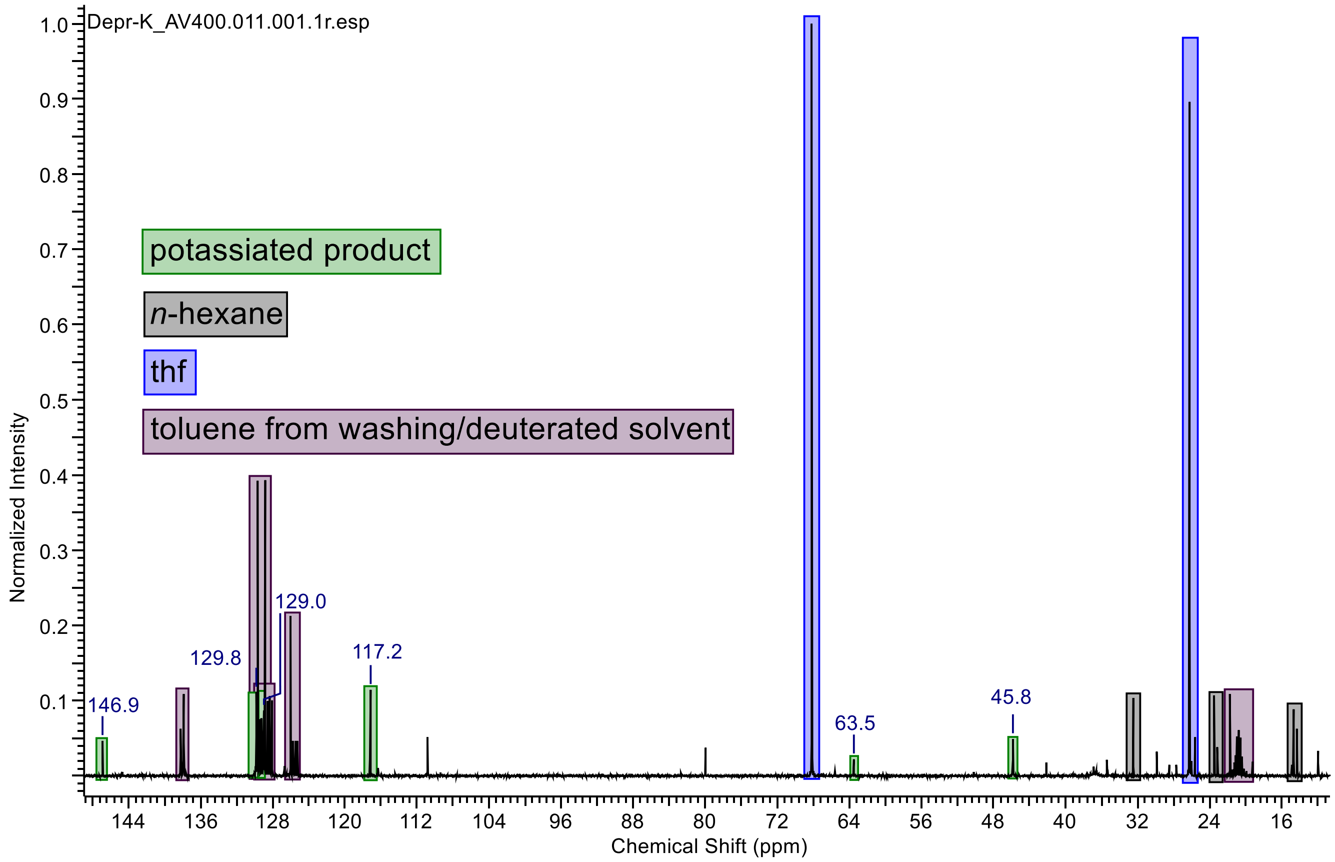

Figure S26 ¹H-NMR spectrum of **[1b·2THF]**$_\infty$.

3.4 Synthesis of Potassiated Compound (9)

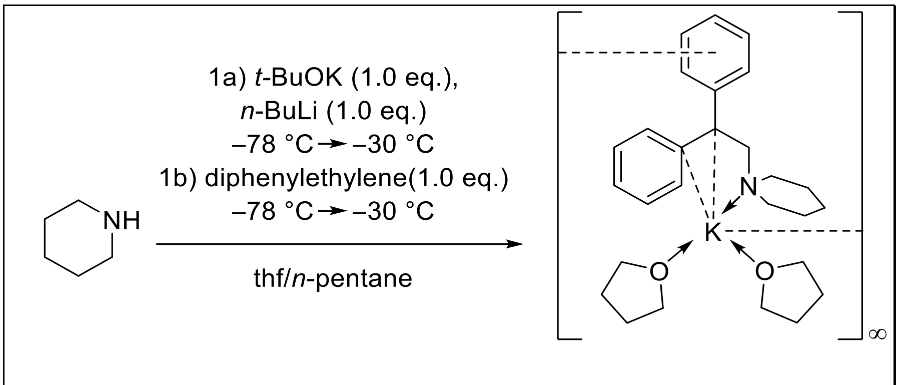

6
$C_5H_{11}N$
85.15 g·mol^{-1}

9
$C_{27}H_{39}KNO_2$
447.71 g·mol^{-1}

To a stirred solution of piperidine (85 mg, 1.0 mmol, 1.0 eq.) in *n*-pentane (1 ml) and thf (1 ml) at –78 °C *n*-butyllithium (0.4 ml, 1.0 mmol, 1.0 eq.; 2.5 M in *n*-hexane) was added. The reaction mixture was warmed up to −30 °C and after one 1 hour cooled down to –80 °C again. Then 1,1-diphenylethene was added, the reaction mixture was allowed to warm up to –30 °C again and afterwards stored at −78 °C. After 3 d homogeneous crystals (orange blocks) of **9** formed, suitable for X-ray structural analysis. A yield could not be obtained. Due to weak bonding of the thf molecules the crystals were not weight stable. Furthermore, by keeping the red crystals for a longer period in vacuum to properly remove the solvent a decomposition and decolorization of the crystals could be observed. Because of their high sensitivity no pure NMR spectrum could be obtained, which is why *n*-hexane and other unidentified decomposition products are visible in the NMR spectra. Nevertheless, the most characteristic signals could be characterized, like the highly shifted para protons of the phenyl rings in the ^{1}H-NMR spectrum.

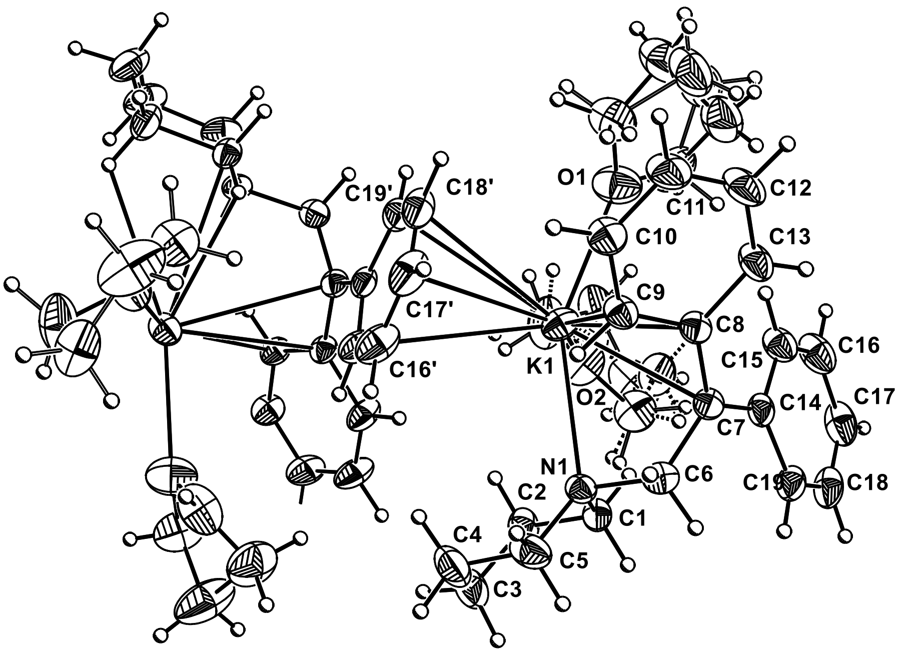

Figure S27 Ortep plot of the molecular structure of **9** in the crystal, with the displacement ellipsoids drawn at the 50% probability level.[4] Numbering scheme of hydrogen atoms omitted for clarity. (Symmetry code: i

= −1 − x, −y, −z) Selected bond lengths [Å] and angles [°]: K1–O1 2.746(18), K1–O2 2.716(12), K1–N1 3.0239(10), K1−C7 3.1639(11), K1−C8 3.1255(11), K1−C19' 3.3996(13), K−C18' 3.2041(13), K−C17' 3.1786(15), K−C16' 3.3000(18), N1−C6 1.4968(15), C6−C7 1.4992(16), C7−C14 1.4360(15), C7−C8 1.4440(14), C14−C15 1.4263(18), C14−C19 1.4289(16), C15−C16 1.3844(18), C16−C17 1.389(2), C17−C18 1.390(2), C18−C19 1.3839(19), C6−C7−C14 120.27(9), C8−C7−C14 123.34(10), C6−C7−C8 116.38(9), C2−C1−C14−C19 −22.16(15), C6−C7−C14−C19 −19.81(12), C8−C7−C14−C15 −24.79(13), C7−C14−C15−C16 −170.52(12), C18−C19−C14−C1 176.98(11).

^{1}H-NMR (400.1 MHz, THF-d$_8$): δ = 2.38 [m, 4H; NC*H*$_2$CH$_2$CH$_2$], 3.25 [s, 2H; CKC*H*$_2$], 6.10 [m, 2H; *H*$_{para}$], 6.83 [m, 4H; *H*$_{meta}$], 7.08 [m, 4H; *H*$_{ortho}$]

{^{1}H}^{13}C-NMR (100.64 MHz, THF-d$_8$): δ = 55.9 [2C, NCH$_2$CH$_2$CH$_2$] 63.6 [1C, CKCH$_2$],117.1 [4C; *C*$_{para}$], 129.0 [4C; *C*$_{ortho}$], 130.0 [4C; *C*$_{meta}$], 146.9 [2C; *C*$_{ipso}$].

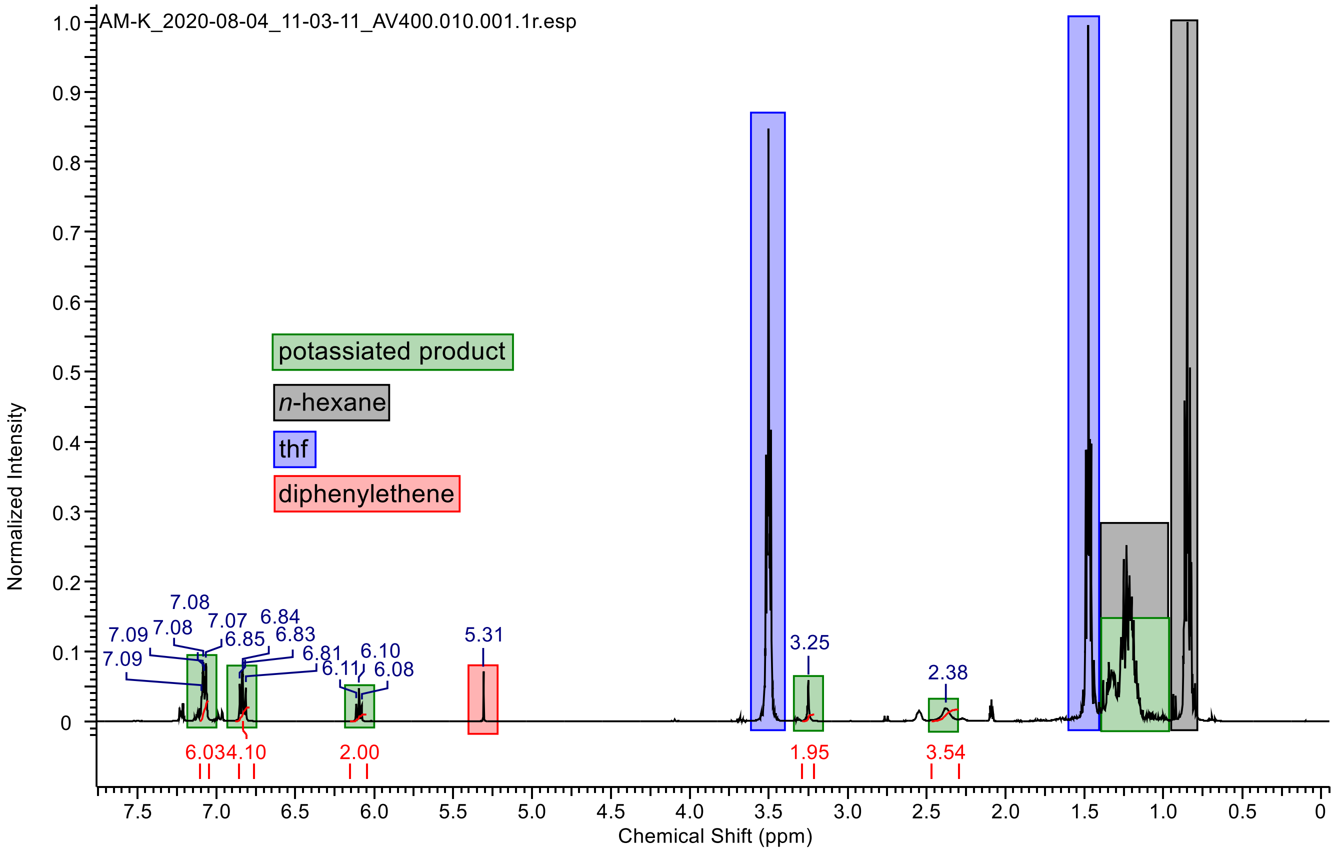

Figure S28 ^{1}H-NMR spectrum of **9**.

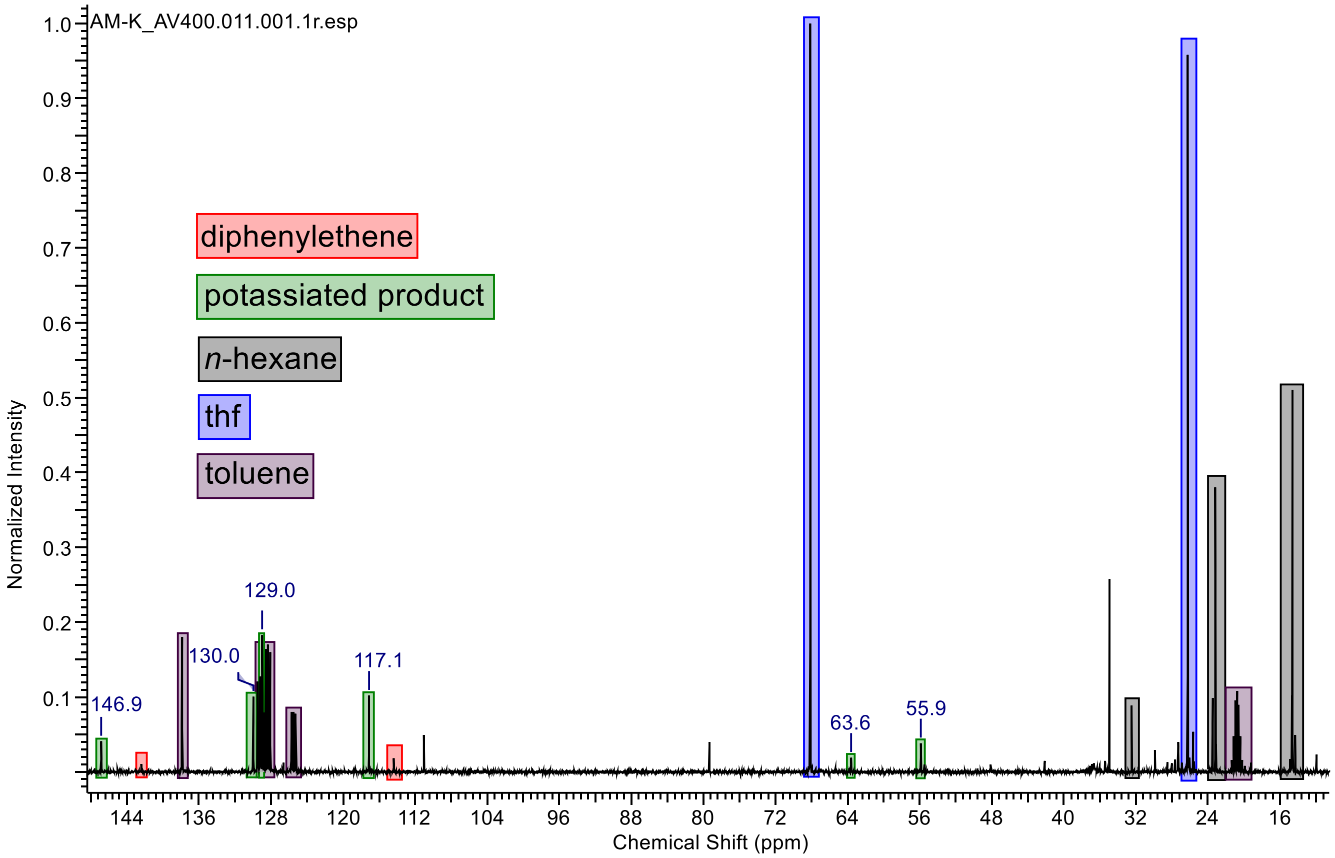

Figure S29 ^{13}C-NMR spectrum of **9**.

3.5 Synthesis of Aggregate (10)

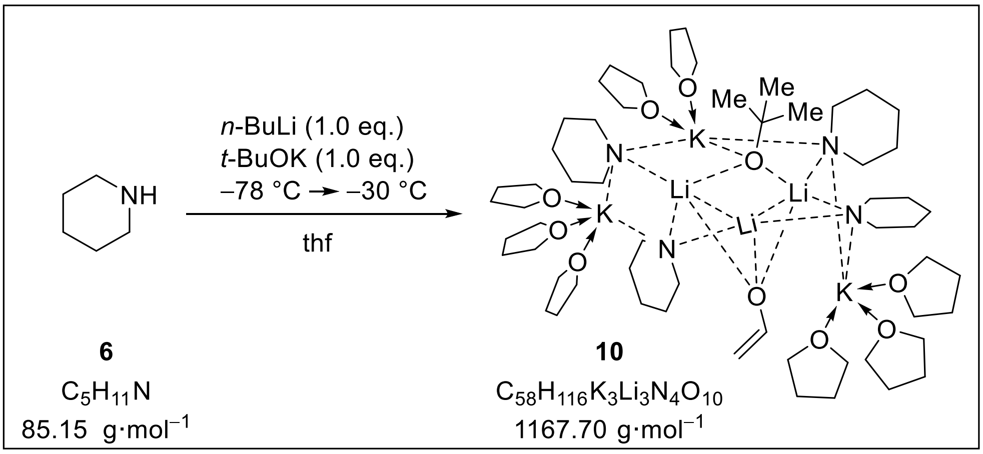

To a stirred solution of Piperidine (85 mg, 1.0 mmol, 1.0 eq.) in *n*-pentane (0.5 ml) and thf (0.5 ml) at –78 °C *n*-butyllithium (0.4 ml, 1.0 mmol, 1.0 eq. of 2.5 M solution of in *n*-hexane) was added. The reaction mixture was stirred for 1 h and was allowed to warm up from –80°C to –30 °C and afterwards stored at –78 °C. After 2 d homogeneous crystals (colourless planks) of **10** were formed, suitable for X-ray structural analysis. No NMR-spectra could be obtained due to the high reactivity of the compound, the deuterated solvent was either deprotonated or cleaved. A yield could not be obtained. Due to weak bonding of the thf molecules the crystals were not weight stable, as well.

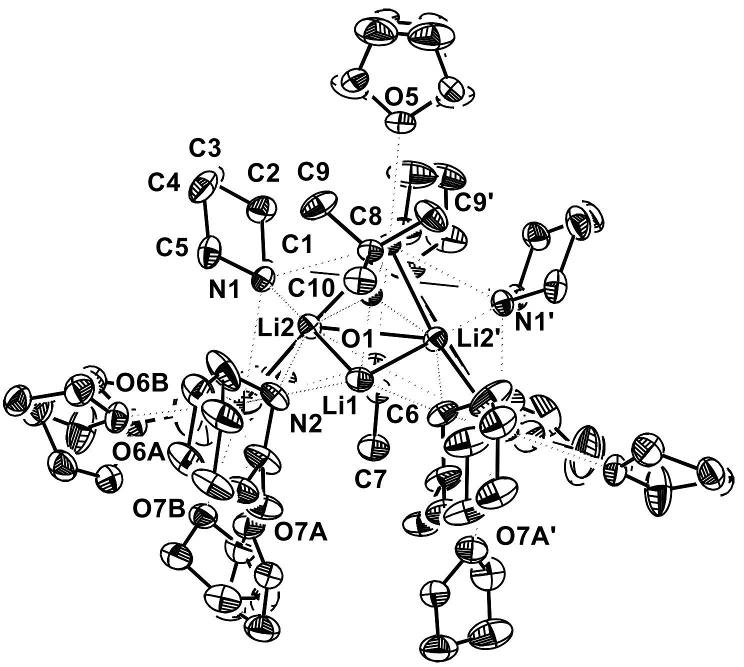

Figure S30 Ortep plot of the molecular structure of **10** in the crystal, with the displacement ellipsoids drawn at the 50% probability level.[4] Numbering scheme of hydrogen atoms omitted for clarity. (Symmetry code: i = +x, 2−y, +z) Selected bond lengths [Å] and angles [°]: K1−O1 27447(17), K1−O2 2.8366(16), K1−O3 2.830(2), K1−O4 2.7564(19), K1−N1 2.9708(15), K1−Li2 2.921(3), K2−O1 3.2081(8), K2−O5 2.7756(14), K2−O6A 2570(3), K2−O6B 2.995(3), K2−O6B 2.995(3), K2−O7A 2.811(3), K2−O7B 2.892(3), K2−N1 2.8816(16), K2−N2 3.0785(16), K2−Li2 2.909(3), K2−C6 3.1328(10), K2−C7 3.2400(13), O1−C6 1.286(3), O1−Li1 2.868(5), O1−Li2 2.040(3), O2−C8 1.396(3), O2−Li1 1.955(4), O2−Li2 1.945(3), N1−Li2 1.986(3), N2−Li1 1.986(3), N2−Li2 2.108(3), C6−C7 1.333(4), Li1−Li2 2.366(4), K1−N1−K2 113.94(5), N1−K1−O1 68.17(3), K1−O1−K2 110.71(3), O1−K2−N1 63.35(4), O2−K1−O4 110.31(6), O1−K1−O3 103.42(), K2−O2−K2^{1} 135.49(6), K2−Li2−K1 114.64(9), Li2−K1−N1 39.39(6), N1−K2−Li2 40.12(7), Li2−N2−Li1 70.39(15), N2−Li1−O2 103.42(13), Li1−O2−Li2 74.72(13), O2−Li2−N2 99.75(14), Li2−O2−Li2 94.32(18), O2−Li2−O1 88.06(12), Li2−O1−Li2 88.72(17), Li2−N2−K2 65.13(9), N2−K2−N1 74.91(4), K2−N1−Li2 70.68(10), N1−Li2−N2 74.91(4), O2−Li1−O1 67.04(13), Li1−O1−K1 102.41(10), O1−K1−O2 59.45(5), O1−K1−O2 59.45(5), K1−O2−Li1 131.11(15), K1−O2−Li1 131.11(15), Li2−O2−K1 72.61(9), Li2−O2−K1 72.61(9), O2−K1−N1 74.00(3), K1−N1−Li2 68.94(9), N1−Li2−O2 125.64(16), Li2−Li1−Li2 74.11(18), Li1−Li2−K1 111.35(12), Li2−K1−Li2 58.45(11), K1−Li2−Li1 111.35(12), O1−Li2−N2 105.40(15), O1−Li2−N1 105.75(14), Li1−N2−K2 119.78(14), N2−K2−O2 63.27(4), K2−N2−Li1 119.78(14), N2−Li1−O1 83.38(13), Li1−O1−K2 93.24(5), O1−C6−C7 128.5(3).

Table S2 Crystal data and structure refinement for **1b·4THF**, **[1b·2THF]$_\infty$** and **[1b·2THF]$_\infty$**.

Compound	**Li-Amid (4a·2THF)$_2$**	**K-Monomer 1b·4THF**	**K-Polymer [1b·2THF]$_\infty$**
Empirical formula	$C_{20}H_{44}Li_2N_2O_4$	$C_{32}H_{50}KNO_4$	$C_{24}H_{34}KNO_2$
Formula weight [g·mol^{-1}]	390.45	551.83	407.62
Temperature [K]	173(2)	100.01	100.01
Wavelength [Å]	0.71073	0.71073	0.71073
Crystal System	triclinic	monoclinic	monoclinic
Space Group (Nr.)	$P\bar{1}$	$P2_1/n$	$P2_1/c$
a [Å]	8.1303(9)	10.603(6)	10.451(2)
b [Å]	8.5936(11)	18.095(9)	57.552(11)
c [Å]	9.2306(12)	16.765(7)	22.780(3)
α [°]	88.833(11)	90.0	90.0
β [°]	68.333(11)	102.873(16)	94.207(6)
γ [°]	89.254(10)	90.0	90.0
Volume [Å^3]	599.23(14)	3136(3)	13664(4)
Z	Z = 1	Z = 4	Z = 24
Density (calculated) ρ [g·cm^{-3}]	1.082	1.169	1.189
Absorption coefficient μ [mm^{-1}]	0.072	0.204	0.251
F(000)	216	1200	5280
Crystal size [mm^3]	0.20 x 0.10 x 0.10	0.40 x 0.20 x 0.20	0.699 x 0.153 x 0.15
Theta range for data collection θ [°]	2.23 – 26.00	4.54 – 54.00	4.168 – 54.00
Index ranges	$-10 \leq h \leq 9$	$-13 \leq h \leq 13$	$-13 \leq h \leq 13$
	$-10 \leq k \leq 10$	$-23 \leq k \leq 23$	$-73 \leq k \leq 73$
	$-11 \leq l \leq 11$	$-18 \leq l \leq 21$	$-29 \leq l \leq 26$
Reflections collected	4307	53288	144670
Independent reflections	4307 (R_{int} = 0.0589)	6855 (R_{int} = 0.0602)	29789 (R_{int} = 0.0430)
Structural refinement	Full-matrix least-squares on F^2	Full-matrix least-squares on F^2	Full-matrix least-squares on F^2
Data / restraints / parameters	4174 / 0 / 142	6855 / 0 / 382	29790 / 0 / 1756
Goodness-of-fit an F^2	0.920	1.031	1.050
Final *R* indices [$I > 2\sigma(I)$]	$R1$ = 0.0589, $wR2$ = 0.1658	$R1$ = 0.0475, $wR2$ = 0.1079	$R1$ = 0.0519, $wR2$ = 0.1113
R indices (all data)	$R1$ = 0.0926, $wR2$ = 0.1789	$R1$ = 0.0670, $wR2$ = 0.1248	$R1$ = 0.0741, $wR2$ = 0.1199
Largest diff. Peak and hole [e·Å^{-3}]	0.32 und –0.31	0.66 und –0.47	0.72 und –0.50

Table S3 Crystal data and structure refinement for **9** and **10**.

Compound	**K-Polymer 9**	**Aggregate 10**
Empirical formula	$C_{27}H_{38}KNO_2$	$C_{58}H_{116}K_3Li_3N_4O_{10}$
Formula weight [g·mol^{-1}]	447.68	1167.66
Temperature [K]	100.0	100.0
Wavelength [Å]	0.71073	0.71073
Crystal System	monoclinic	orthorhombic
Space Group (Nr.)	$P2_1/n$	*Pnma*
a [Å]	12.6917(8)	25.768(2)
b [Å]	14.1688(10)	25.685(3)
c [Å]	13.9663(8)	11.5045(11)
α [°]	90.0	90
β [°]	94.964(3)	90
γ [°]	90.0	90
Volume [Å^3]	2502.1(3)	7614.3(15)
Z	Z = 4	Z = 12
Density (calculated) ρ [g·cm^{-3}]	1.188	1.019
Absorption coefficient μ [mm^{-1}]	0.235	0.226
F(000)	968.0	2552.0
Crystal size [mm^3]	0.398 x 0.291 x 0.197	1.14 x 0.222 x 0.183
Theta range for data collection θ [°]	4.102 – 63.05	4.478 – 55.918
Index ranges	$-18 \leq h \leq 18$	$-33 \leq h \leq 33$
	$-20 \leq k \leq 20$	$-33 \leq k \leq 33$
	$-20 \leq l \leq 17$	$-15 \leq l \leq 14$
Reflections collected	56800	143461
Independent reflections	8345 (R_{int} = 0.0332)	9344 (R_{int} = 0.0518)
Structural refinement	Full-matrix least-squares on F^2	Full-matrix least-squares on F^2
Data / restraints / parameters	8345 /0 / 372	9344 / 12 / 524
Goodness-of-fit an F^2	1.038	1.038
Final *R* indices [$I > 2\sigma(I)$]	$R1$ = 0.0436<br>w$R2$ = 0.1173	$R1$ = 0.0535,<br>w$R2$ = 0.1466
R indices (all data)	$R1$ = 0.0566,<br>w$R2$ = 0.1263	$R1$ = 0.0694,<br>w$R2$ = 0.1572
Largest diff. Peak and hole [e·Å^{-3}]	0.63 und –0.52	0.68 und –0.59

4 Quantum Chemical Calculations

Optimization and additional harmonic vibrational frequency analyses were performed with the software package Gaussian 09 (Revision E.01) on the M062X/6-31+G(d) level of theory.[5] The GJF input files were created with the program GaussView 5.0. The ground state structures were optimized without symmetry restrictions. Vibrational frequency analysis showed no imaginary frequency in the harmonical approximation in case of ground states. The calculations of the structures include the first coordination sphere of the lithium cation. Benchmarks and more details of the used methods can be found in reference of the corresponding paper.[6] The calculated standard orientations of the optimized structures can be found in the following Tables.

4.1 Calculated Deprotonation Reactions of Different Phenylethylamine Derivatives

4.1.1 Deprotonation of *N,N*-dimethyl-2,2-diphenylethan-1-amine (2a)

In the following, the energies (Table S4) and the coordinates of the opitimized structures for the deprotonation reaction of **2a** with various lithium alkyls (*t*-BuLi/MeLi/*i*-PrLi; Scheme S3) and the two Schlosser bases tBuONa/t-BuLi and t-BuOK/t-BuLi (Scheme S4) are listed.

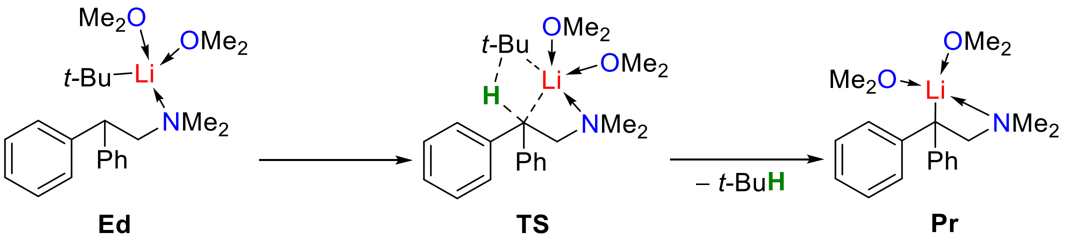

Scheme S3 Calculated reaction scheme for the deprotonation of **2a** with different lithium alkyls; exemplary for *t*-BuLi; with stationary points: Ed = educt; TS = transition state; Pr = product.

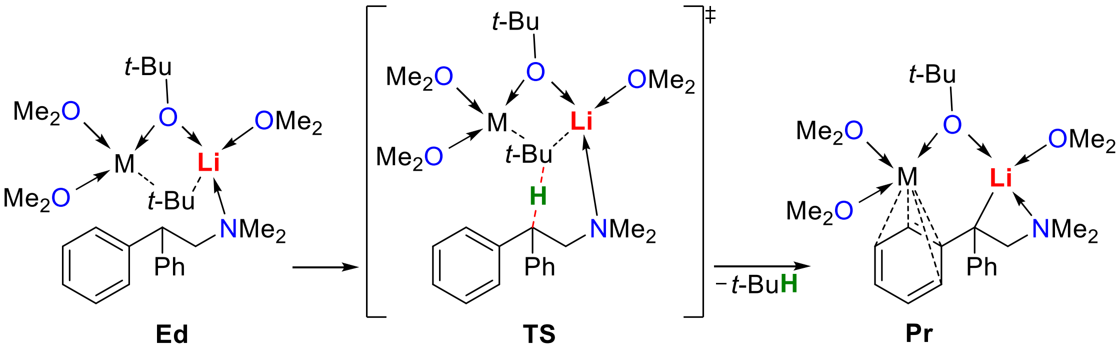

M= Na, K

Scheme S4 Calculated reaction scheme for the deprotonation of **2a** with the Schlosser-base mixture *t*-BuOM/*t*-BuLi; with stationary points: Ed = educt; TS = transition state; Pr = product.

Table S4 Total (SCF) and zero-point-corrected (ZPE) energies of the optimized structures and stationary points of the deprotonation reaction of **2a**.

Stationary point	Metalating reagent	Calculation number	SCF [Hartree]	ZPE [Hartree]
Ed		QM1	–1150.82070444	–1150.215383
TS	*t*-BuLi	QM2	–1150.78920603	–1150.187758
Pr		QM3	–992.494769963	–992.022605
	t-BuH	QM4	–158.363419798	–158.230481
Ed		QM5	–1032.94900865	–1032.430647
TS	MeLi	QM6	–1032.92304560	–1032.408507
Pr		QM3	–992.494769963	–992.022605
	MeH	QM7	–40.4835867202	–40.438039
Ed		QM8	–1111.52638289	–1110.949353
TS	*i*-PrLi	QM9	–1111.50110978	–1110.929585
Pr		QM3	–992.494769963	–992.022605
	i-PrH	QM10	–119.067575926	–118.962723
Ed		QM11	–1701.10235608	–1700.286257
TS	*t*-BuONa/*t*-BuLi	QM12	–1701.06517614	1700.253458
Pr		QM13	–1542.76799801	–1542.085553
Ed		QM14	–2138.71386283	–2137.898466
TS	*t*-BuOK/*t*-BuLi	QM15	–2138.68471831	–2137.874343
Pr		QM16	–1980.38710100	–1979.705505

Table S5 Coordinates of QM1.

Element	X	Y	Z
C	–1.653065	3.988177	–0.890217
C	–1.374758	2.981396	–1.815775
C	–1.220899	1.664147	–1.391175
C	–1.356870	1.318328	–0.040820
C	–1.632518	2.337314	0.876178
C	–1.773559	3.661838	0.459001
C	–1.318937	–0.156280	0.326106
C	–0.758100	–0.535394	1.707161
N	0.695103	–0.335630	1.865960
C	1.507747	–1.984876	–1.377117
C	0.530935	–1.756257	–2.534877
C	0.941425	–3.139384	–0.547816
Li	1.829518	–0.374489	0.028483
C	4.551271	0.738512	0.764282
C	4.285163	–1.580463	0.992704
C	1.244662	–1.398535	2.713366
C	1.001060	0.953726	2.482969
C	1.951366	2.828617	–0.364729
C	2.258117	1.355979	–2.159882

O	2.044734	1.475002	–0.761306
O	3.636278	–0.320011	0.961809
C	2.788098	–2.511894	–2.031169
H	–1.774644	5.016512	–1.218385
H	–1.277069	3.222868	–2.871155
H	–1.004827	0.879144	–2.114350
H	–1.747857	2.101701	1.932349
H	–1.988634	4.436266	1.190343
H	–0.661757	–0.653291	–0.397583
H	–0.955856	–1.605877	1.823700
H	–1.309633	–0.032077	2.521372
H	0.373534	–2.688052	–3.125998
H	–0.470761	–1.432126	–2.217645
H	0.888104	–1.002787	–3.254230
H	1.653181	–3.494734	0.214933
H	0.014940	–2.874632	–0.012454
H	0.695884	–4.030943	–1.171678
H	4.971715	0.700488	–0.249928
H	4.010392	1.676119	0.896324
H	5.365748	0.679190	1.498893
H	4.915650	–1.657245	1.889374
H	3.508734	–2.347929	1.010611
H	4.900446	–1.718798	0.095587
H	1.128332	–2.364972	2.215114
H	2.307692	–1.209190	2.883509
H	0.735934	–1.435880	3.692358
H	0.572100	1.761500	1.890557
H	0.594349	1.018144	3.508688
H	2.088099	1.080244	2.529993
H	1.055749	3.296906	–0.791057
H	1.891229	2.860936	0.723645
H	2.844298	3.381470	–0.692092
H	2.334716	0.290992	–2.379004
H	1.413748	1.791730	–2.711409
H	3.184316	1.876051	–2.442766
H	3.287112	–1.759243	–2.663373
H	3.529331	–2.858070	–1.298339
H	2.585759	–3.384361	–2.694867
C	–2.709629	–0.774044	0.189813
C	–2.871869	–1.987611	–0.485749
C	–3.832167	–0.180508	0.776282
C	–4.125186	–2.592301	–0.574504
H	–2.011240	–2.463312	–0.950146
C	–5.084521	–0.785403	0.694155
H	–3.733887	0.773057	1.289356
C	–5.235969	–1.994145	0.016416

H	–4.230435	–3.532117	–1.109231
H	–5.944470	–0.307256	1.155029
H	–6.213186	–2.463095	–0.054857

Table S6 Coordinates of QM2.

Element	X	Y	Z
C	–1.385992	4.185378	–0.471327
C	–1.536335	3.196996	–1.447433
C	–1.474619	1.852145	–1.098977
C	–1.278099	1.433300	0.232284
C	–1.157355	2.445694	1.198276
C	–1.201601	3.798323	0.854804
C	–1.233641	–0.035492	0.514636
C	–0.571472	–0.457533	1.820636
N	0.926815	–0.420317	1.856973
C	0.315946	–1.305268	–1.632864
C	–0.922287	–1.540982	–2.494827
C	0.707638	–2.642965	–1.001955
Li	1.654765	–0.430610	–0.088783
C	4.633278	–0.870747	–0.968489
C	3.983645	–2.546324	0.510731
C	1.396052	–1.509023	2.718323
C	1.416355	0.838062	2.429307
C	3.598449	1.933710	0.325840
C	1.797617	2.326606	–1.132256
O	2.529740	1.353463	–0.394502
O	3.680131	–1.254422	0.010776
C	1.427888	–0.856812	–2.588745
H	–1.427439	5.237561	–0.738523
H	–1.698035	3.477212	–2.485620
H	–1.593584	1.089196	–1.865040
H	–1.050050	2.180886	2.247042
H	–1.108922	4.551998	1.633275
H	–0.393144	–0.538408	–0.505854
H	–0.842578	–1.497629	2.003257
H	–0.942976	0.098554	2.704697
H	–0.730904	–2.323492	–3.254092
H	–1.791088	–1.857901	–1.909207
H	–1.212064	–0.636740	–3.045734
H	1.595366	–2.587304	–0.352519
H	–0.105981	–3.047117	–0.387496
H	0.945148	–3.406360	–1.769218
H	4.657836	–1.604846	–1.784550
H	4.331695	0.097819	–1.366396
H	5.631255	–0.796496	–0.515398

H	4.968640	−2.543109	0.996108
H	3.219424	−2.815905	1.238580
H	3.980834	−3.282953	−0.303406
H	1.126618	−2.474856	2.278322
H	2.484180	−1.453817	2.825266
H	0.947544	−1.447299	3.724614
H	1.151322	1.678630	1.787142
H	0.989864	1.009763	3.433001
H	2.505731	0.793498	2.521814
H	3.228262	2.690073	1.030397
H	4.101194	1.134199	0.873501
H	4.306662	2.408909	−0.367065
H	1.076441	1.789923	−1.745965
H	1.258837	2.999867	−0.451991
H	2.481447	2.903101	−1.769187
H	1.133186	0.036117	−3.154543
H	2.389163	−0.624970	−2.109724
H	1.647640	−1.647471	−3.335571
C	−2.570916	−0.707413	0.361092
C	−2.717132	−2.099869	0.546248
C	−3.744965	−0.009163	0.017912
C	−3.934853	−2.749167	0.378889
H	−1.850106	−2.704241	0.799856
C	−4.966048	−0.657703	−0.157689
H	−3.709559	1.067711	−0.112350
C	−5.077264	−2.034480	0.017603
H	−3.987491	−3.825750	0.522310
H	−5.841908	−0.071440	−0.424946
H	−6.029150	−2.538816	−0.119566

Table S7 Coordinates of QM3.

Element	X	Y	Z
C	−2.253825	2.348588	−1.238650
C	−1.316064	1.336714	−1.441430
C	−0.079321	1.269078	−0.715778
C	0.032259	2.260635	0.321534
C	−0.920066	3.243614	0.525164
C	−2.075815	3.327560	−0.265999
H	−3.141047	2.368329	−1.870053
H	−1.524942	0.612255	−2.223531
H	0.902165	2.252519	0.969819
H	−0.757126	3.968709	1.319879
H	−2.804891	4.117780	−0.118569
C	0.472191	−0.860056	−1.909527
H	0.059943	−0.424183	−2.831857
H	1.341539	−1.456466	−2.223640

N	–0.589976	–1.787204	–1.385510
C	–1.374128	–2.357328	–2.474906
H	–0.741130	–2.915064	–3.186350
H	–2.125010	–3.046655	–2.074192
H	–1.886770	–1.559693	–3.022431
C	0.012893	–2.864806	–0.603137
H	–0.772948	–3.482546	–0.153107
H	0.655983	–3.511011	–1.226600
H	0.623501	–2.437659	0.197851
Li	–1.080209	–0.509841	0.169614
C	0.866608	0.211949	–0.930137
O	–0.470079	–1.003751	1.968173
O	–2.948203	–0.574537	0.704805
C	–3.921203	–0.683828	–0.318605
H	–4.869492	–1.046009	0.099181
H	–4.075994	0.287844	–0.802364
H	–3.541880	–1.398918	–1.050724
C	–3.296221	0.409635	1.671446
H	–4.234743	0.130605	2.168383
H	–2.483541	0.444195	2.399095
H	–3.398620	1.390662	1.192040
C	–1.039703	–2.101030	2.655334
H	–2.021711	–2.283966	2.215155
H	–0.407653	–2.993497	2.547170
H	–1.148159	–1.867693	3.723011
C	0.830445	–0.682117	2.444296
H	0.790320	–0.478019	3.521817
H	1.526235	–1.509145	2.249460
H	1.171222	0.199286	1.903074
C	2.259564	0.260022	–0.488509
C	3.033379	–0.915759	–0.317808
C	2.949388	1.474131	–0.250203
C	4.360774	–0.882851	0.095390
H	2.581973	–1.886537	–0.504440
C	4.273365	1.505987	0.174378
H	2.445483	2.414606	–0.448893
C	4.997102	0.328564	0.365895
H	4.900935	–1.819711	0.213372
H	4.752370	2.469341	0.334287
H	6.030961	0.354683	0.696576

Table S8 Coordinates of QM4.

Element	X	Y	Z
C	–0.000089	0.000006	0.380973
H	–0.000144	–0.000103	1.480259
C	0.231895	–1.433797	–0.096535

H	–0.557824	–2.105355	0.258034
H	0.239029	–1.474273	–1.193369
H	1.192635	–1.822291	0.259008
C	–1.357754	0.516112	–0.096499
H	–2.174671	–0.121975	0.25833
H	–1.544858	1.535563	0.258684
H	–1.396164	0.53104	–1.193358
C	1.125867	0.917664	–0.096467
H	0.981995	1.944518	0.257499
H	2.102107	0.570148	0.25939
H	1.158376	0.942819	–1.193304

Table S9 Coordinates of QM5.

Element	X	Y	Z
C	–1.445609	4.027313	0.045485
C	–1.324688	3.272805	–1.123175
C	–1.177604	1.891575	–1.047134
C	–1.150755	1.22827	0.185984
C	–1.295286	1.992859	1.346068
C	–1.433338	3.381571	1.27919
C	–1.060669	–0.286064	0.164806
C	–0.445698	–0.993799	1.387503
N	1.014688	–0.855775	1.532748
C	1.240636	–2.220162	–1.688087
Li	1.904113	–0.789519	–0.336138
C	4.745341	–0.026224	–0.144717
C	4.246631	–2.317686	–0.253081
C	1.566906	–2.074137	2.13071
C	1.399965	0.29196	2.347126
C	2.243015	2.347044	–0.489889
C	1.912156	1.098439	–2.449674
O	1.980998	1.069319	–1.031666
O	3.82993	–1.040266	0.20969
H	–1.557447	5.10651	–0.007181
H	–1.345918	3.762785	–2.093366
H	–1.092769	1.300487	–1.957611
H	–1.301943	1.506503	2.319206
H	–1.539046	3.955436	2.195948
H	–0.417241	–0.567014	–0.680434
H	–0.645476	–2.058618	1.235392
H	–0.961510	–0.710437	2.323486
H	4.773363	0.10863	–1.235179
H	4.411568	0.899944	0.326636
H	5.752615	–0.274893	0.216215
H	5.178589	–2.609460	0.250073
H	3.447392	–3.023648	–0.024853
H	4.391878	–2.301967	–1.339741
H	1.370991	–2.922775	1.46846
H	2.64834	–1.959310	2.24776
H	1.121973	–2.277862	3.120323
H	1.007533	1.211695	1.912894
H	1.021611	0.204222	3.382312
H	2.493745	0.353039	2.382198

H	1.402909	3.02702	–0.682831
H	2.374749	2.234885	0.587431
H	3.161885	2.767303	–0.925110
H	1.639281	0.091712	–2.770916
H	1.15881	1.828145	–2.776180
H	2.889211	1.381364	–2.867307
C	–2.426901	–0.913448	–0.103165
C	–2.490043	–2.075750	–0.880942
C	–3.606749	–0.401378	0.441966
C	–3.708435	–2.711387	–1.108537
H	–1.571978	–2.476582	–1.309096
C	–4.826797	–1.039977	0.218421
H	–3.578358	0.510345	1.034141
C	–4.881692	–2.195693	–0.557661
H	–3.740456	–3.608080	–1.721126
H	–5.736570	–0.628239	0.646829
H	–5.833247	–2.688410	–0.736410
H	2.029858	–2.650840	–2.333962
H	0.822137	–3.093120	–1.144306
H	0.438618	–1.929002	–2.395895

Table S10 Coordinates of QM6.

Element	X	Y	Z
C	–0.611978	4.113315	0.129555
C	–0.892740	3.317602	–0.986042
C	–1.014807	1.938958	–0.857147
C	–0.877633	1.288305	0.389781
C	–0.561837	2.107751	1.489213
C	–0.443846	3.493577	1.365272
C	–1.080052	–0.187095	0.445741
C	–0.556832	–0.868965	1.701625
N	0.900338	–1.213533	1.614338
C	0.252826	–1.357482	–1.840814
Li	1.500568	–0.571671	–0.261295
C	4.462406	–0.860012	–0.388398
C	3.332739	–2.733250	–1.249998
C	1.05978	–2.662221	1.463168
C	1.64768	–0.762716	2.784428
C	2.564445	2.150511	0.115695
C	2.219572	1.578776	–2.137976
O	2.262261	1.113767	–0.800264
O	3.256451	–1.597383	–0.406524
H	–0.529495	5.192228	0.033843
H	–1.025872	3.778809	–1.962276
H	–1.251557	1.333086	–1.729976
H	–0.444387	1.674437	2.478434
H	–0.227174	4.090712	2.248095
H	–0.338652	–0.723087	–0.615990
H	–1.120684	–1.791211	1.88634
H	–0.701680	–0.259732	2.605123
H	4.65661	–0.408522	–1.370768
H	4.353755	–0.069039	0.35518
H	5.302278	–1.511098	–0.112119

H	4.112508	–3.418316	–0.891776
H	2.360398	–3.227290	–1.222418
H	3.553273	–2.434426	–2.283214
H	0.52782	–2.992546	0.564547
H	2.120076	–2.910532	1.359743
H	0.648851	–3.204382	2.331677
H	1.531757	0.318839	2.90337
H	1.304991	–1.253482	3.711745
H	2.711466	–0.988749	2.64751
H	1.834156	2.962399	0.028023
H	2.508049	1.727586	1.121723
H	3.57841	2.533784	–0.070098
H	1.954231	0.732499	–2.772595
H	1.460384	2.363141	–2.239053
H	3.203367	1.97306	–2.429002
C	–2.446123	–0.652766	0.045734
C	–3.549260	0.201709	–0.118255
C	–2.659395	–2.021786	–0.207324
C	–4.794723	–0.284792	–0.511706
H	–3.433238	1.265246	0.07319
C	–3.902677	–2.512006	–0.592752
H	–1.822282	–2.711957	–0.114237
C	–4.983704	–1.644564	–0.751752
H	–5.625950	0.407416	–0.621721
H	–4.025563	–3.575446	–0.782801
H	–5.954638	–2.022396	–1.058909
H	1.253813	–1.255006	–2.298623
H	–0.454461	–0.938231	–2.569184
H	0.042614	–2.436204	–1.794255

Table S11 Coordinates of QM7.

Element	X	Y	Z
C	0	0	0
H	0.630646	0.630646	0.630646
H	–0.630646	–0.630646	0.630646
H	–0.630646	0.630646	–0.630646
H	0.630646	–0.630646	–0.630646

Table S12 Coordinates of QM8.

Element	X	Y	Z
C	1.036229	4.042717	0.570204
C	0.859137	3.089643	1.574637
C	0.87112	1.732816	1.261858
C	1.07974	1.294719	–0.052348
C	1.242014	2.260414	–1.051034
C	1.216526	3.622936	–0.745743
C	1.242231	–0.197880	–0.289583
C	0.713117	–0.749384	–1.624338
N	–0.758280	–0.710061	–1.760166
C	–1.283932	–1.897535	1.664514
C	–0.403796	–1.468672	2.846677
C	–0.730686	–3.233838	1.149727

Li	–1.771380	–0.563503	0.091996
C	–4.665159	0.030098	–0.636303
C	–4.149365	–2.192927	–0.092442
C	–1.248574	–1.988011	–2.289058
C	–1.198899	0.368686	–2.641335
C	–2.332546	2.547867	0.06675
C	–2.629193	1.288711	2.025293
O	–2.322840	1.258801	0.641902
O	–3.670894	–0.971116	–0.630712
H	1.031651	5.102009	0.811234
H	0.713115	3.404511	2.604891
H	0.734017	0.989405	2.044823
H	1.409201	1.954295	–2.081948
H	1.353315	4.35491	–1.537152
H	0.662275	–0.714318	0.487174
H	1.027585	–1.797706	–1.663224
H	1.193837	–0.254498	–2.485010
H	–0.304076	–2.244385	3.633562
H	0.630326	–1.245332	2.534387
H	–0.769494	–0.560429	3.35025
H	–1.424406	–3.752051	0.469524
H	0.205619	–3.100393	0.578121
H	–0.486800	–3.962534	1.951475
H	–4.956456	0.296451	0.389455
H	–4.247054	0.909969	–1.128535
H	–5.550956	–0.313802	–1.187683
H	–4.976120	–2.576630	–0.706021
H	–3.317083	–2.897550	–0.094412
H	–4.488847	–2.051516	0.941623
H	–1.003016	–2.789626	–1.587076
H	–2.334605	–1.937124	–2.401434
H	–0.797828	–2.216426	–3.270195
H	–0.816803	1.325071	–2.280793
H	–0.846585	0.21841	–3.677653
H	–2.294192	0.403528	–2.649936
H	–1.588871	3.192881	0.549191
H	–2.082513	2.444206	–0.989959
H	–3.330532	3.000792	0.165048
H	–2.629993	0.254997	2.375228
H	–1.866792	1.862479	2.569802
H	–3.614547	1.750761	2.183698
C	2.702021	–0.615545	–0.133640
C	3.024867	–1.728808	0.648794
C	3.734044	0.055654	–0.797738
C	4.345361	–2.160256	0.76678
H	2.23359	–2.259107	1.173472
C	5.054249	–0.375507	–0.685810
H	3.508963	0.934553	–1.396772
C	5.364957	–1.485457	0.098582
H	4.576166	–3.023284	1.384995
H	5.842527	0.160662	–1.207054
H	6.394622	–1.818860	0.191286
H	–2.278043	–2.127513	2.104611

Table S13 Coordinates of QM9.

Element	X	Y	Z
C	–0.846964	4.168337	–0.023473
C	–0.721621	3.313203	–1.116900
C	–0.743484	1.929793	–0.932727
C	–0.924937	1.356239	0.336764
C	–1.062914	2.239169	1.42084
C	–1.013511	3.621371	1.25086
C	–1.061499	–0.135569	0.466981
C	–0.400054	–0.758629	1.691577
N	1.08688	–0.613490	1.743961
C	0.379975	–1.506951	–1.626058
C	–0.433859	–1.087874	–2.849091
C	–0.079116	–2.891002	–1.158079
Li	1.651434	–0.427983	–0.218450
C	4.537039	–0.852319	0.300766
C	3.554317	–2.822112	–0.517594
C	1.675631	–1.836215	2.297424
C	1.500433	0.506461	2.591703
C	2.617743	2.458514	–0.130672
C	2.429002	1.395194	–2.226465
O	2.480584	1.225209	–0.818878
O	3.380939	–1.441200	–0.258100
H	–0.829593	5.245872	–0.161237
H	–0.606411	3.724134	–2.117689
H	–0.656714	1.26571	–1.789778
H	–1.242134	1.833198	2.415045
H	–1.134215	4.276013	2.11059
H	–0.263172	–0.692603	–0.562522
H	–0.576632	–1.834810	1.633456
H	–0.822278	–0.432239	2.664537
H	–0.484049	–1.872951	–3.622135
H	–1.470968	–0.862224	–2.560766
H	–0.027569	–0.188226	–3.332233
H	0.469271	–3.246958	–0.272981
H	–1.139020	–2.854926	–0.873652
H	0.015932	–3.668647	–1.935163
H	5.381577	–0.928772	–0.396968
H	4.312296	0.199606	0.482337
H	4.806921	–1.347236	1.244574
H	3.747916	–3.366038	0.417535
H	2.634862	–3.189337	–0.975201
H	4.395087	–2.976588	–1.206422
H	1.461774	–2.684633	1.64093
H	2.76167	–1.722413	2.376224
H	1.275038	–2.056605	3.301606
H	1.105633	1.446441	2.206828
H	1.144641	0.375343	3.628788
H	2.595536	0.562101	2.611076
H	1.694659	3.045354	–0.196965
H	2.831642	2.228854	0.914047
H	3.455688	3.028979	–0.553505
H	2.295143	0.406884	–2.670571
H	1.587019	2.040514	–2.504297

H	3.367635	1.838959	–2.583784
C	–2.473934	–0.591272	0.270925
C	–3.375262	0.1262	–0.550027
C	–2.998648	–1.761879	0.864262
C	–4.672038	–0.310063	–0.790327
H	–3.052359	1.050997	–1.017709
C	–4.300171	–2.198914	0.622849
H	–2.392773	–2.366631	1.530664
C	–5.154259	–1.484679	–0.211463
H	–5.315537	0.28244	–1.436407
H	–4.645632	–3.111266	1.103694
H	–6.168004	–1.825848	–0.398600
H	1.441249	–1.578156	–1.942356

Table S14 Coordinates of QM10.

Element	X	Y	Z
C	0	0.592545	0
H	–0.000001	1.249747	0.878229
C	1.267146	–0.261139	0.000001
H	1.30052	–0.907586	0.884525
H	1.300236	–0.908035	–0.884207
H	2.17189	0.355102	–0.000303
C	–1.267146	–0.261139	0.000001
H	–1.300424	–0.907732	0.884422
H	–2.171890	0.355102	–0.000103
H	–1.300332	–0.907890	–0.884309
H	0.000001	1.249692	–0.878267

Table S15 Coordinates of QM11.

Element	X	Y	Z
C	–0.472433	–3.517712	0.727683
C	–0.815973	–2.271314	0.206775
C	–1.918917	–1.567514	0.690301
C	–2.695091	–2.153572	1.695526
C	–2.360916	–3.401211	2.21648
C	–1.242706	–4.085900	1.739273
H	0.407586	–4.033196	0.351706
H	–0.220641	–1.828970	–0.588579
H	–3.567531	–1.628997	2.078465
H	–2.971405	–3.838616	3.001627
H	–0.976022	–5.053826	2.153926
C	–2.202555	–0.159606	0.186978
H	–1.434354	0.071922	–0.565003
C	–1.968533	0.81813	1.354744
H	–2.859819	0.876968	2.008235
H	–1.151020	0.413121	1.95197
N	–1.544571	2.162956	0.950788
C	–2.603781	2.874799	0.240021
H	–2.265902	3.887411	–0.000284
H	–2.851281	2.368014	–0.693666
H	–3.520320	2.947717	0.853281
C	–1.173996	2.907161	2.155723

H	–0.884787	3.925894	1.883844
H	–2.013486	2.957146	2.872646
H	–0.316457	2.421342	2.631538
O	4.086513	–0.491476	–1.029295
C	–0.470062	2.089877	–2.375756
H	–0.491106	3.025638	–1.796702
H	–0.458459	2.386386	–3.450587
H	–1.435656	1.592295	–2.206028
C	0.608071	–0.032276	–2.919849
H	0.529291	0.241169	–3.997908
H	1.487647	–0.699657	–2.860267
H	–0.279487	–0.646508	–2.693558
C	4.864412	0.534535	–0.442879
H	4.871039	1.428517	–1.081025
H	4.403708	0.779029	0.517042
H	5.895278	0.190695	–0.282212
C	4.566313	–0.869041	–2.304149
H	5.575217	–1.296940	–2.222887
H	3.876649	–1.617310	–2.700216
H	4.589303	–0.005461	–2.982308
O	1.327976	0.539336	1.371135
C	1.788823	–0.080051	2.523267
C	0.634836	–0.537859	3.435237
H	1.007484	–1.049483	4.33173
H	0.04047	0.323447	3.764068
H	–0.023262	–1.230284	2.894754
C	2.606247	–1.336847	2.154577
H	3.455342	–1.063322	1.511862
H	3.0068	–1.855593	3.034613
H	1.963888	–2.045535	1.611215
C	2.69183	0.872621	3.328382
H	3.02242	0.431417	4.277136
H	3.586817	1.135118	2.749584
H	2.146529	1.799392	3.545775
C	0.719699	1.201683	–2.019963
Li	0.457837	1.673235	0.20527
O	1.252632	3.510993	0.404199
C	2.622116	3.461229	0.751596
H	2.807109	2.442126	1.092987
H	3.249627	3.696925	–0.120181
H	2.837355	4.176025	1.558694
C	0.870067	4.764133	–0.112961
H	1.404453	4.981434	–1.048626
H	–0.201902	4.725545	–0.315674
H	1.076514	5.560342	0.616811
C	3.181948	–3.510132	–0.700733
H	3.7134	–2.869392	0.003792
H	2.818623	–4.410434	–0.185405
H	3.86552	–3.812886	–1.507235
O	2.096749	–2.766966	–1.222775
C	1.409974	–3.480648	–2.234474
H	1.058234	–4.449817	–1.855697
H	0.554332	–2.880347	–2.545805
H	2.069814	–3.650265	–3.097248

C	1.972722	1.95142	–2.476003
H	2.082029	2.918876	–1.966851
H	2.898742	1.38595	–2.288085
H	1.956465	2.171276	–3.569900
Na	1.848284	–0.478052	–0.510652
C	–3.559993	–0.092441	–0.498908
C	–3.630716	–0.327343	–1.876776
C	–4.752483	0.148213	0.190857
C	–4.850581	–0.316366	–2.548530
H	–2.711055	–0.512724	–2.428787
C	–5.976577	0.160015	–0.476598
H	–4.738006	0.340924	1.260313
C	–6.031397	–0.070931	–1.849312
H	–4.877308	–0.494583	–3.619889
H	–6.889617	0.354196	0.079601
H	–6.984499	–0.057322	–2.370030

Table S16 Coordinates of QM12.

Element	X	Y	Z
C	0.028014	2.796161	2.323911
C	0.355479	1.529334	1.838368
C	1.449229	1.304534	0.968318
C	2.163218	2.47228	0.595526
C	1.835028	3.732322	1.078128
C	0.765364	3.916308	1.957062
H	–0.810409	2.895464	3.011775
H	–0.257637	0.696237	2.160712
H	3.02159	2.385453	–0.062248
H	2.437368	4.585026	0.771297
H	0.526662	4.900404	2.351461
C	1.82805	–0.052297	0.457331
H	0.934868	–0.363920	–0.647088
C	1.466281	–1.177799	1.433565
H	2.22831	–1.326370	2.223745
H	0.539507	–0.936084	1.954025
N	1.202435	–2.496132	0.798733
C	2.403406	–3.026203	0.154171
H	2.195146	–4.019030	–0.258253
H	2.728554	–2.374944	–0.655831
H	3.231719	–3.121507	0.87803
C	0.773764	–3.436047	1.833738
H	0.583313	–4.418047	1.389192
H	1.539499	–3.551048	2.621465
H	–0.152584	–3.082015	2.292519
O	–3.288903	1.316504	–1.446777
C	1.179495	–1.512493	–2.702167
H	1.067619	–2.546766	–2.344857
H	0.987241	–1.525762	–3.792792
H	2.229083	–1.240224	–2.563918
C	0.331484	0.840822	–2.548321
H	0.472534	0.83414	–3.645747
H	–0.574994	1.442721	–2.376332
H	1.168155	1.401968	–2.110043

C	–4.262176	0.288842	–1.445177
H	–4.221534	–0.285264	–2.381939
H	–4.025568	–0.372031	–0.609352
H	–5.268072	0.713483	–1.323887
C	–3.348001	2.088063	–2.629360
H	–4.340024	2.547349	–2.741861
H	–2.586847	2.864851	–2.549699
H	–3.137743	1.460212	–3.506863
O	–1.850434	–0.906429	0.911368
C	–2.697259	–0.813579	2.015167
C	–1.975601	–1.136754	3.338951
H	–2.643394	–1.000799	4.198794
H	–1.639162	–2.178657	3.343067
H	–1.101916	–0.494719	3.493168
C	–3.268859	0.615197	2.122552
H	–3.782735	0.891862	1.193012
H	–3.983181	0.718777	2.948374
H	–2.457237	1.33472	2.293779
C	–3.870902	–1.804968	1.903659
H	–4.523406	–1.763815	2.784586
H	–4.488338	–1.594328	1.0222
H	–3.478600	–2.824980	1.814854
C	0.201578	–0.579710	–1.984400
Li	–0.653191	–1.930060	–0.000615
O	–1.446866	–3.684456	–0.521481
C	–2.821239	–3.664430	–0.865046
H	–3.225440	–2.725533	–0.486645
H	–2.944830	–3.710387	–1.955992
H	–3.341230	–4.514119	–0.403378
C	–0.751668	–4.736431	–1.157798
H	–0.835504	–4.648827	–2.250335
H	0.2977	–4.655693	–0.872922
H	–1.149990	–5.709280	–0.839841
C	–2.282263	3.904063	0.076984
H	–2.846471	3.144352	0.622527
H	–1.870920	4.634672	0.78589
H	–2.957433	4.415181	–0.626129
O	–1.239783	3.253051	–0.619100
C	–0.437996	4.164666	–1.341917
H	–0.036852	4.935503	–0.672350
H	0.388998	3.602804	–1.778005
H	–1.027262	4.645149	–2.138525
C	–1.195375	–1.091847	–2.382033
H	–1.414418	–0.842913	–3.440636
H	–1.253018	–2.183781	–2.315712
H	–2.022616	–0.693229	–1.784843
Na	–1.252366	0.94398	–0.209333
C	3.297269	–0.064954	0.054943
C	3.720146	0.306099	–1.230194
C	4.312501	–0.372889	0.976241
C	5.066833	0.333091	–1.591840
H	2.97793	0.592433	–1.967213
C	5.66093	–0.357317	0.624966
H	4.055051	–0.619180	2.002278

C	6.049911	–0.012086	–0.668015
H	5.343527	0.623997	–2.602102
H	6.411508	–0.604788	1.371754
H	7.100085	–0.000636	–0.945769

Table S17 Coordinates of QM13.

Element	X	Y	Z
C	0.642135	–0.092209	–2.814849
C	–0.482104	0.46028	–2.211966
C	–1.418935	–0.313972	–1.435623
C	–1.026063	–1.700206	–1.295223
C	0.09612	–2.231011	–1.905752
C	0.964996	–1.443993	–2.683241
H	1.285711	0.556093	–3.405675
H	–0.655994	1.522499	–2.365430
H	–1.645662	–2.371986	–0.712231
H	0.296954	–3.292813	–1.776084
H	1.830683	–1.871017	–3.180196
C	–2.728141	1.756297	–0.959115
H	–2.594337	2.062586	–2.007928
H	–3.750587	2.061202	–0.684039
N	–1.754459	2.5864	–0.178188
C	–1.724572	3.950503	–0.698373
H	–2.718632	4.426724	–0.637745
H	–1.014115	4.554405	–0.124543
H	–1.407175	3.942	–1.745605
C	–2.123323	2.619633	1.235733
H	–1.359923	3.163531	1.804351
H	–3.097457	3.117055	1.390938
H	–2.191400	1.597178	1.61712
C	–2.553623	0.269706	–0.818775
C	2.526127	2.474065	–0.377455
O	1.716903	1.451521	0.102784
C	1.854329	3.207849	–1.551093
H	2.459596	4.047174	–1.915905
H	1.684156	2.516471	–2.382781
H	0.882852	3.602258	–1.230524
C	3.868637	1.905919	–0.868405
H	4.530115	2.682262	–1.272722
H	4.394983	1.408042	–0.044110
H	3.683367	1.165046	–1.657537
C	2.785097	3.500244	0.741457
H	3.405416	4.340767	0.406022
H	1.825712	3.898233	1.097164
H	3.287883	3.024776	1.592747
O	3.075499	–2.637882	–0.407143
C	2.961539	–3.893882	–1.039682
H	3.840217	–4.515312	–0.817821
H	2.067583	–4.380104	–0.645616
H	2.865968	–3.781275	–2.128688
C	4.253628	–1.956668	–0.787283
H	4.248687	–1.735321	–1.864047
H	4.285465	–1.018882	–0.226420

H	5.143064	–2.554890	–0.544303
Li	–0.093194	1.370804	–0.024762
O	–0.142005	0.057379	1.619494
C	0.421077	0.733421	2.736988
H	1.146452	1.443696	2.338037
H	–0.370478	1.254208	3.293299
H	0.915217	0.012729	3.401518
C	–1.121306	–0.883708	2.039464
H	–1.917563	–0.385859	2.608891
H	–1.558198	–1.336295	1.152874
H	–0.648371	–1.647569	2.672236
O	2.777088	–1.106502	1.962724
C	3.600506	–0.055000	2.426822
H	4.65855	–0.272056	2.216029
H	3.285196	0.841064	1.889398
H	3.476909	0.085268	3.510617
C	3.093553	–2.344238	2.560038
H	2.98766	–2.280760	3.652452
H	2.402585	–3.089746	2.164531
H	4.122514	–2.644007	2.31442
Na	1.588337	–0.756380	–0.010918
C	–3.658859	–0.499499	–0.243072
C	–4.465261	0.016123	0.800874
C	–4.039037	–1.775399	–0.725925
C	–5.529669	–0.696680	1.343255
H	–4.252025	1.002158	1.203354
C	–5.096250	–2.492911	–0.176586
H	–3.524172	–2.186507	–1.588833
C	–5.850878	–1.969870	0.874005
H	–6.110291	–0.250426	2.147577
H	–5.349115	–3.464688	–0.594955
H	–6.677149	–2.529847	1.301687

Table S18 Coordinates of QM14.

Element	X	Y	Z
C	0.563838	–0.647335	–2.827198
C	–0.464494	0.060605	–2.214947
C	–1.494885	–0.576907	–1.427939
C	–1.247438	–1.986521	–1.212031
C	–0.214050	–2.672486	–1.827740
C	0.713214	–2.027495	–2.664439
H	1.265183	–0.093639	–3.449479
H	–0.508808	1.130762	–2.402119
H	–1.923446	–2.552986	–0.580957
H	–0.126348	–3.743678	–1.650815
H	1.50189	–2.579244	–3.166707
C	–2.668254	1.613781	–1.203347
H	–2.474205	1.782633	–2.273553
H	–3.683684	1.998236	–1.014778
N	–1.688432	2.491807	–0.484258

C	–1.561451	3.765406	–1.188955
H	–2.533978	4.282539	–1.262181
H	–0.857810	4.419658	–0.665907
H	–1.186346	3.5935	–2.202571
C	–2.130775	2.741976	0.886484
H	–1.370457	3.321063	1.424026
H	–3.082532	3.302314	0.910521
H	–2.276193	1.787289	1.398871
C	–2.589411	0.144547	–0.892762
C	2.391575	2.540354	–0.161703
O	1.681621	1.402777	0.196635
C	2.146888	2.875783	–1.643799
H	2.692077	3.769779	–1.972380
H	2.451371	2.030288	–2.273076
H	1.075827	3.043792	–1.812344
C	3.900819	2.332315	0.062047
H	4.492256	3.191127	–0.279155
H	4.10798	2.183563	1.128802
H	4.243469	1.440095	–0.474615
C	1.944523	3.736591	0.699484
H	2.457376	4.668758	0.430169
H	0.864315	3.891259	0.585006
H	2.145323	3.527854	1.75798
O	3.904382	–1.772459	–1.028812
C	4.714146	–2.913117	–1.198410
H	5.772592	–2.631228	–1.286663
H	4.582281	–3.544689	–0.317473
H	4.417045	–3.473468	–2.096073
C	4.013266	–0.873060	–2.114568
H	3.728825	–1.363374	–3.056302
H	3.330523	–0.041692	–1.919548
H	5.039674	–0.489289	–2.197299
Li	–0.114160	1.234325	–0.039332
O	–0.366505	0.15171	1.707335
C	0.140651	0.888141	2.811517
H	0.892919	1.56888	2.410235
H	–0.672138	1.447348	3.295819
H	0.594544	0.202985	3.540412
C	–1.388470	–0.749069	2.111604
H	–2.249559	–0.203345	2.521018
H	–1.714427	–1.302236	1.232587
H	–0.993828	–1.433469	2.875266
K	1.564726	–1.151433	0.143688
O	2.50409	–1.439299	2.657707
C	3.295537	–0.263425	2.720867
H	4.351836	–0.499072	2.529531

H	2.922991	0.426852	1.956075
H	3.205249	0.206919	3.710862
C	2.849611	–2.372488	3.655461
H	2.703669	–1.942923	4.656526
H	2.197705	–3.240251	3.537533
H	3.898185	–2.686281	3.553628
C	–3.777322	–0.486763	–0.306918
C	–4.566521	0.174143	0.664748
C	–4.258092	–1.753785	–0.716581
C	–5.711135	–0.396718	1.212094
H	–4.276148	1.164347	1.003464
C	–5.397554	–2.329436	–0.164132
H	–3.754726	–2.271906	–1.526757
C	–6.134340	–1.665113	0.817049
H	–6.274553	0.156781	1.959919
H	–5.728214	–3.300561	–0.525407
H	–7.023957	–2.115080	1.2474

Table S19 Coordinates of QM15.

Element	X	Y	Z
C	–0.219101	–2.972978	2.161623
C	–0.968035	–1.828559	1.874233
C	–1.478142	–1.603161	0.593453
C	–1.267323	–2.590563	–0.380989
C	–0.526293	–3.734664	–0.100481
C	0.016679	–3.924159	1.17283
H	0.179472	–3.114005	3.162889
H	–1.142784	–1.105057	2.663239
H	–1.687230	–2.451363	–1.373791
H	–0.375828	–4.480244	–0.876586
H	0.597569	–4.814925	1.397788
C	–2.241241	–0.339195	0.202445
H	–1.799225	–0.005021	–0.747844
C	–2.029925	0.82063	1.197614
H	–2.811685	0.809824	1.982504
H	–1.055863	0.710677	1.681183
N	–1.983053	2.13871	0.549672
C	–3.233162	2.455791	–0.132923
H	–3.171258	3.462812	–0.556715
H	–3.414030	1.757853	–0.950314
H	–4.095084	2.415815	0.558331
C	–1.705972	3.153319	1.565565
H	–1.742230	4.146925	1.109933
H	–2.445083	3.120461	2.387198
H	–0.702341	2.996649	1.967717
O	4.062641	–0.287290	–0.854245

C	–0.965318	1.798439	–2.816971
H	–0.935278	2.811267	–2.394697
H	–0.912077	1.917278	–3.925257
H	–1.967477	1.397157	–2.600585
C	–0.077949	–0.447442	–2.988403
H	–0.029683	–0.340180	–4.097210
H	0.668118	–1.227362	–2.748360
H	–1.067425	–0.877698	–2.771614
C	4.216579	1.106036	–0.657274
H	4.332435	1.625059	–1.618623
H	3.304834	1.455675	–0.162156
H	5.090632	1.312147	–0.021647
C	5.1002	–0.846719	–1.625708
H	6.073284	–0.708225	–1.132285
H	4.885396	–1.912287	–1.729353
H	5.135436	–0.383372	–2.621743
O	1.171489	1.265947	1.172348
C	1.813356	1.247604	2.399734
C	0.823287	1.140606	3.579143
H	1.344988	1.122695	4.544513
H	0.128627	1.986977	3.587178
H	0.238834	0.217738	3.498528
C	2.757334	0.026443	2.497551
H	3.487924	0.039691	1.678798
H	3.310729	–0.001426	3.444782
H	2.171172	–0.902862	2.430961
C	2.648716	2.524774	2.615892
H	3.115522	2.550528	3.608799
H	3.44804	2.599134	1.868302
H	2.001674	3.404766	2.513863
C	0.140479	0.891461	–2.281762
Li	0.106847	1.86765	–0.230954
O	0.506616	3.859575	–0.497211
C	1.86679	4.215605	–0.360219
H	2.365971	3.361185	0.09441
H	2.309186	4.440202	–1.341266
H	1.969308	5.092938	0.294441
C	–0.248554	4.884881	–1.101845
H	0.112639	5.084505	–2.120572
H	–1.285370	4.548598	–1.151557
H	–0.187955	5.807267	–0.506308
K	1.479409	–0.858235	–0.314220
C	3.765265	–3.267717	0.492457
H	3.98417	–2.260425	0.856133
H	3.25215	–3.842491	1.2772
H	4.70882	–3.771919	0.236855

O	2.943819	–3.134015	–0.648076
C	2.688259	–4.370013	–1.274356
H	2.205164	–5.071386	–0.578805
H	2.021673	–4.177929	–2.117395
H	3.622287	–4.819055	–1.641606
C	1.469228	1.438979	–2.805610
H	1.702566	2.436059	–2.402184
H	2.320669	0.784222	–2.556114
H	1.475663	1.538712	–3.917701
C	–3.699019	–0.706906	–0.043177
C	–4.214296	–0.748558	–1.341208
C	–4.536575	–1.061987	1.018845
C	–5.538704	–1.118339	–1.573788
H	–3.572370	–0.476617	–2.178378
C	–5.860204	–1.430526	0.792695
H	–4.145757	–1.060598	2.034716
C	–6.367366	–1.457807	–0.506527
H	–5.921963	–1.138161	–2.590237
H	–6.495457	–1.701250	1.6315
H	–7.399929	–1.743777	–0.685093

Table S20 Coordinates of QM16.

Element	X	Y	Z
C	0.401749	–2.728520	2.386119
C	–0.434430	–1.656482	2.061221
C	–1.166001	–1.619552	0.855518
C	–1.051743	–2.763468	0.027199
C	–0.227161	–3.832346	0.351675
C	0.525659	–3.821203	1.531815
H	0.947826	–2.707614	3.326958
H	–0.511644	–0.839715	2.769606
H	–1.625287	–2.794118	–0.895817
H	–0.172891	–4.685261	–0.321302
H	1.1666	–4.659488	1.792015
C	–1.958967	–0.455282	0.366768
H	–1.192367	0.026768	–0.773105
C	–1.970492	0.719787	1.343479
H	–2.729082	0.607994	2.147526
H	–0.993845	0.794041	1.829163
N	–2.163279	2.04518	0.703768
C	–3.475933	2.146674	0.069982
H	–3.620834	3.159826	–0.319437
H	–3.561036	1.442517	–0.755178
H	–4.283472	1.934049	0.793205
C	–2.051144	3.085553	1.724945
H	–2.198539	4.069897	1.269147

H	–2.801742	2.953556	2.524426
H	–1.053063	3.059286	2.168415
O	3.892315	–0.075409	–1.197121
C	–1.579287	1.377759	–2.718642
H	–1.585570	2.360749	–2.227755
H	–1.396927	1.557072	–3.796624
H	–2.594093	0.976813	–2.630769
C	–0.517891	–0.858918	–2.908520
H	–0.384334	–0.678694	–3.993966
H	0.299287	–1.533939	–2.606584
H	–1.446734	–1.428398	–2.793566
C	3.979668	1.337237	–1.177693
H	3.896716	1.744011	–2.196185
H	3.143287	1.697818	–0.570684
H	4.932996	1.659785	–0.733640
C	4.873601	–0.667186	–2.019264
H	5.882215	–0.450877	–1.638132
H	4.693705	–1.743534	–2.008919
H	4.793826	–0.291551	–3.049088
O	1.213309	1.540741	0.960026
C	1.998257	1.718388	2.091951
C	1.161192	1.737677	3.385774
H	1.797436	1.805594	4.277349
H	0.482692	2.596616	3.394894
H	0.562054	0.824577	3.467005
C	3.020509	0.566769	2.230182
H	3.632613	0.481836	1.322911
H	3.698083	0.7144	3.080039
H	2.495559	–0.386578	2.393703
C	2.769211	3.050986	2.028166
H	3.354375	3.233036	2.938426
H	3.460227	3.0644	1.176356
H	2.05489	3.874389	1.906403
C	–0.517710	0.454514	–2.120521
Li	–0.196611	2.028405	–0.063108
O	–0.014229	3.925284	–0.670445
C	1.296977	4.398331	–0.915792
H	1.980011	3.678593	–0.464240
H	1.482138	4.47012	–1.996957
H	1.439472	5.384818	–0.454590
C	–0.996397	4.751987	–1.258603
H	–0.854176	4.807044	–2.346654
H	–1.970032	4.308751	–1.046676
H	–0.948605	5.763381	–0.832461
K	1.489817	–0.841373	–0.151032
C	4.01957	–2.967981	0.440464

H	4.207743	−1.921824	0.698132
H	3.603069	−3.495291	1.310656
H	4.968913	−3.441619	0.150203
O	3.10385	−2.978924	−0.634268
C	2.858757	−4.280191	−1.119812
H	2.483337	−4.933545	−0.320483
H	2.101938	−4.198437	−1.902143
H	3.777811	−4.714646	−1.538820
C	0.835848	1.120112	−2.425815
H	0.883707	1.407172	−3.496807
H	1.036322	2.038647	−1.860279
H	1.698872	0.459581	−2.271559
C	−3.363401	−0.952875	0.027687
C	−3.840772	−1.117519	−1.276696
C	−4.230260	−1.321237	1.07051
C	−5.124534	−1.603138	−1.534161
H	−3.201802	−0.854731	−2.111223
C	−5.513405	−1.802362	0.826776
H	−3.881803	−1.244426	2.098851
C	−5.971750	−1.942519	−0.483579
H	−5.459144	−1.713689	−2.562561
H	−6.153012	−2.078940	1.661185
H	−6.971715	−2.319116	−0.680128

4.1.2 Deprotonation of *N,N*-dimethyl-2-phenyl-2-(trimethyl-silyl)ethan-1-amine (11)

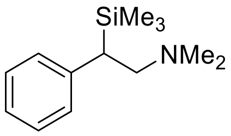

11

Figure S31 Lewis formula of *N,N*-dimethyl-2-phenyl-2-(trimethyl-silyl)ethan-1-amine (**11**).

In the following, the energies (Table S21) and the coordinates of the opitimized structures for the deprotonation reaction of **11** with various lithium alkyls (*t*-BuLi/MeLi/*i*-PrLi; Scheme S5) and the two Schlosser bases *t*-BuONa/*t*-BuLi and *t*-BuOK/*t*-BuLi (Scheme S6) are listed.

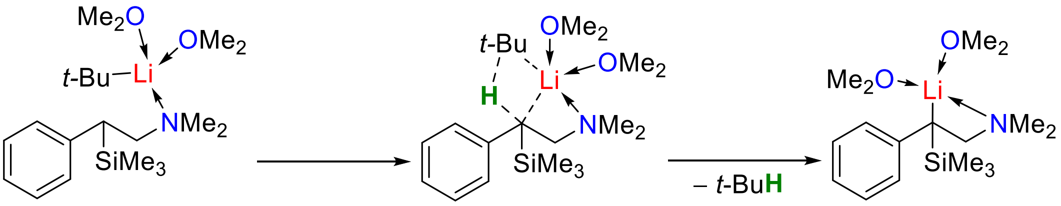

Scheme S5 Calculated reaction scheme for the deprotonation of **11** with different lithium alkyls; exemplary for *t*-BuLi; with stationary points: Ed = educt; TS = transition state; Pr = product.

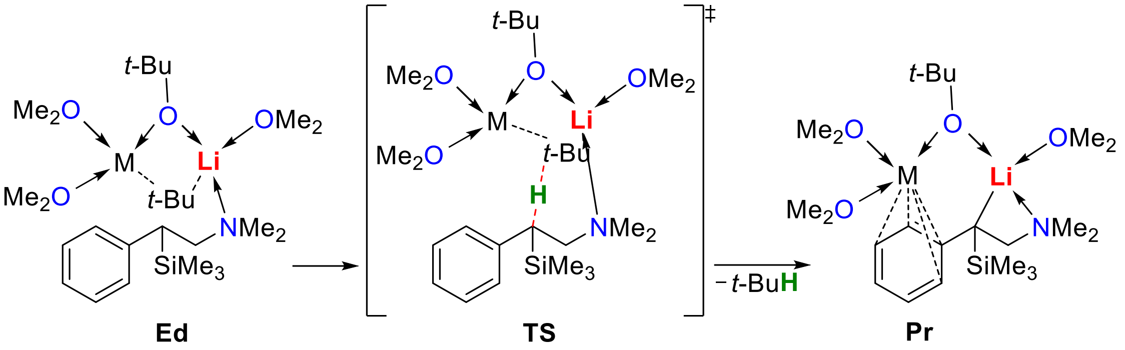

M= Na, K

Scheme S6 Calculated reaction scheme for the deprotonation of **11** with the Schlosser-base mixture *t*-BuOM/*t*-BuLi; with stationary points: Ed = educt; TS = transition state; Pr = product.

Table S21 Total (SCF) and zero-point-corrected (ZPE) energies of the optimized structures and stationary points of the deprotonation reaction of **11**.

stationary point			SCF [Hartree]	ZPE [Hartree]
Ed		QM17	–1328.43521694	–1327.810950
TS	*t*-BuLi	QM18	–1328.40023452	–1327.778783
Pr		QM19	–1170.10072953	–1169.608823
	t-BuH	QM4	–158.363419798	–158.230481
Ed		QM20	–1210.56330768	–1210.024184
TS	MeLi	QM21	-1210.53814781	-1210.003351
Pr		QM19	–1170.10072953	–1169.608823
	MeH	QM7	–40.4835867202	–40.438039
Ed		QM22	–1289.13992553	–1288.542791
TS	*i*-PrLi	QM23	–1289.11585193	–1288.522571
Pr		QM19	–1170.10072953	–1169.608823
	i-PrH	QM10	–119.067575926	–118.962723
Ed		QM24	–1878.7086686	–1877.872654
TS	*t*-BuONa/*t*-BuLi	QM25	–1878.67459604	–1877.841895
Pr		QM26	–1720.37377244	–1719.671084
Ed		QM27	–2316.32264421	–2315.487908
TS	*t*-BuOK/*t*-BuLi	QM28	–2316.29242873	–2315.460939
Pr		QM29	–2157.99443281	–2157.292651

Table S22 Coordinates of QM17.

Element	X	Y	Z
C	–2.586254	3.538692	–0.570301
C	–1.953181	2.743638	–1.526731
C	–1.500277	1.47018	–1.191671
C	–1.683568	0.947457	0.096858
C	–2.323510	1.756453	1.045431
C	–2.761561	3.040382	0.720064
C	–1.302329	–0.487751	0.360275
C	–0.656015	–0.830595	1.703335

N	0.728139	–0.334294	1.857797
C	1.589816	–1.640621	–1.509314
C	0.528808	–1.656093	–2.610413
C	1.37304	–2.908360	–0.681220
Li	1.726451	–0.092356	–0.024132
C	4.386549	1.308601	0.609662
C	4.387942	–1.003751	0.96797
C	1.48091	–1.228507	2.737279
C	0.74817	1.011615	2.427224
C	1.405945	3.144226	–0.216303
C	1.778489	1.803615	–2.099344
O	1.615624	1.827433	–0.687990
O	3.592123	0.153962	0.788332
Si	–2.855896	–1.576768	0.055983
C	–2.363993	–3.388695	–0.104869
C	–3.708491	–1.015170	–1.523255
C	–4.038374	–1.386944	1.51541
C	2.93481	–1.797653	–2.222038
H	–2.939676	4.53287	–0.828046
H	–1.810126	3.118436	–2.537527
H	–1.002940	0.851463	–1.938148
H	–2.482057	1.380133	2.054575
H	–3.253031	3.647601	1.475636
H	–0.577976	–0.790947	–0.408862
H	–0.604099	–1.923533	1.774442
H	–1.268362	–0.494264	2.559975
H	0.607294	–2.568744	–3.245405
H	–0.503668	–1.652656	–2.226279
H	0.610322	–0.800612	–3.301082
H	2.181154	–3.082209	0.047454
H	0.431911	–2.885443	–0.108967
H	1.330273	–3.829147	–1.309835
H	4.952378	1.243165	–0.329772
H	3.717771	2.168167	0.57087
H	5.088306	1.423913	1.447282
H	4.911134	–0.958238	1.9337
H	3.723399	–1.868334	0.942779
H	5.123985	–1.095647	0.159945
H	1.568873	–2.215013	2.272203
H	2.482225	–0.818357	2.895383
H	0.990226	–1.338771	3.720449
H	0.147021	1.683473	1.813729
H	0.343125	1.021468	3.455717
H	1.782836	1.373036	2.454006
H	0.415067	3.507099	–0.518679
H	1.471125	3.131928	0.872137
H	2.178687	3.818318	–0.613870
H	1.828148	0.754806	–2.393264
H	0.926359	2.294585	–2.588552
H	2.704728	2.326022	–2.377668
H	–1.836376	–3.761874	0.780536
H	–3.264098	–4.002157	–0.234605
H	–1.714791	–3.552758	–0.972250
H	–4.099399	0.003819	–1.427960

H	–3.019651	–1.032197	–2.376017
H	–4.546216	–1.682925	–1.756152
H	–4.356892	–0.346693	1.646116
H	–4.939130	–1.989872	1.349291
H	–3.584170	–1.726325	2.453836
H	3.16743	–0.947896	–2.883939
H	3.776791	–1.893652	–1.522068
H	2.964769	–2.705640	–2.868101

Table S23 Coordinates of QM18.

Element	X	Y	Z
C	1.987071	3.786643	0.04762
C	1.78612	2.978782	1.167269
C	1.459726	1.632114	1.020492
C	1.33605	1.022915	–0.246227
C	1.548709	1.86386	–1.360105
C	1.864895	3.212807	–1.219546
C	1.108523	–0.451140	–0.312394
C	0.542891	–1.031351	–1.603597
N	–0.918117	–0.798364	–1.843184
C	–0.720328	–1.553092	1.688685
C	0.017211	–1.255691	2.990814
C	–0.333507	–2.957320	1.231853
Li	–1.607069	–0.217037	0.056054
C	–4.569904	0.693222	0.158293
C	–4.247156	–1.393414	–0.833688
C	–1.425192	–1.876717	–2.692596
C	–1.149819	0.465459	–2.545616
C	–1.801325	2.847742	–0.117086
C	–1.572748	2.013769	2.057265
O	–1.734496	1.680066	0.687453
O	–3.630314	–0.179340	–0.443196
Si	2.824702	–1.218699	–0.054735
C	2.952245	–2.959777	–0.805541
C	3.438747	–1.336695	1.730457
C	4.133237	–0.196022	–0.974683
C	–2.227776	–1.573115	1.989425
H	2.243847	4.836077	0.158791
H	1.883808	3.401434	2.165323
H	1.295892	1.015389	1.903589
H	1.501883	1.443384	–2.362586
H	2.037598	3.816484	–2.107800
H	0.11471	–0.850755	0.697577
H	0.641623	–2.119975	–1.537796
H	1.077787	–0.735484	–2.533865
H	–0.093205	–2.083569	3.715748
H	1.090872	–1.114371	2.824095

H	–0.353247	–0.353601	3.495137
H	–0.813118	–3.223247	0.276492
H	0.751098	–3.036915	1.086245
H	–0.617553	–3.729062	1.969649
H	–4.964414	0.24785	1.081983
H	–4.054843	1.622789	0.394754
H	–5.400935	0.891129	–0.531609
H	–4.893673	–1.229203	–1.706319
H	–3.459502	–2.103827	–1.086210
H	–4.847149	–1.802893	–0.010132
H	–1.393909	–2.829106	–2.154045
H	–2.456312	–1.666456	–2.992668
H	–0.819316	–1.974478	–3.609992
H	–0.737727	1.294299	–1.970882
H	–0.666510	0.465978	–3.539411
H	–2.227400	0.618061	–2.675402
H	–0.799269	3.27793	–0.242199
H	–2.214133	2.565362	–1.087008
H	–2.466789	3.587592	0.349517
H	–1.300673	1.104501	2.589666
H	–0.774599	2.754051	2.177133
H	–2.516103	2.416405	2.453829
H	2.883953	–2.926478	–1.899686
H	3.935971	–3.375204	–0.553314
H	2.194819	–3.661413	–0.440862
H	3.315947	–0.393450	2.274509
H	2.932459	–2.127780	2.293701
H	4.510874	–1.571006	1.713152
H	4.25888	0.796379	–0.525770
H	5.104165	–0.706581	–0.952348
H	3.858021	–0.044884	–2.026385
H	–2.661387	–0.582638	2.201411
H	–2.806201	–2.015096	1.167651
H	–2.441569	–2.199852	2.876127

Table S24 Coordinates of QM19.

Element	X	Y	Z
C	–1.857400	2.431553	–1.349343
C	–0.973231	1.351037	–1.390804
C	0.191461	1.271946	–0.554191
C	0.304471	2.360996	0.376383
C	–0.591975	3.411516	0.420633
C	–1.692010	3.478053	–0.450127
H	–2.693147	2.448831	–2.048420
H	–1.159938	0.575974	–2.132009
H	1.146285	2.374249	1.06044

H	–0.428695	4.207463	1.144365
H	–2.381726	4.315447	–0.423130
C	0.842005	–0.874107	–1.646670
H	0.539509	–0.423393	–2.605116
H	1.735177	–1.483394	–1.875271
N	–0.264642	–1.823179	–1.271412
C	–0.829219	–2.455667	–2.456905
H	–0.060687	–3.000521	–3.032298
H	–1.607228	–3.170423	–2.166657
H	–1.272597	–1.696607	–3.110344
C	0.247539	–2.852089	–0.367521
H	–0.576746	–3.481231	–0.012022
H	0.990805	–3.497222	–0.869018
H	0.732759	–2.373000	0.488757
Li	–1.027494	–0.500804	0.116633
C	1.107213	0.168524	–0.587413
O	–0.911527	–0.896594	2.015261
O	–3.002854	–0.592740	0.213611
C	–3.722390	–0.754631	–0.993658
H	–4.759783	–1.044529	–0.780839
H	–3.715695	0.176763	–1.574451
H	–3.228657	–1.543871	–1.562331
C	–3.547239	0.443897	1.021199
H	–4.585362	0.20504	1.286883
H	–2.934235	0.501187	1.921796
H	–3.503334	1.402238	0.489653
C	–1.509535	–2.048680	2.575632
H	–2.324535	–2.342266	1.911167
H	–0.776444	–2.862957	2.656171
H	–1.908225	–1.823754	3.573896
C	0.178269	–0.409358	2.780015
H	–0.146873	–0.209589	3.809604
H	0.999557	–1.138706	2.787852
H	0.511807	0.5137	2.305535
Si	2.859018	0.181518	–0.007631
C	3.343435	1.324687	1.430501
H	4.381689	1.090552	1.698694
H	3.312957	2.383058	1.150046
H	2.737463	1.192551	2.334259
C	4.034159	0.663102	–1.421789
H	3.816065	1.680323	–1.768105
H	5.085739	0.626446	–1.109928
H	3.916898	–0.007846	–2.281772
C	3.425411	–1.549611	0.542339
H	2.918443	–1.873766	1.459646
H	3.254063	–2.316961	–0.221386

H	4.502684	–1.532711	0.75051

Table S25 Coordinates of QM 20.

Element	X	Y	Z
C	3.285583	3.107259	–0.283153
C	2.464676	2.824929	0.807142
C	1.726992	1.642801	0.839556
C	1.786264	0.714298	–0.207533
C	2.63226	1.004945	–1.287464
C	3.366993	2.187777	–1.329095
C	0.995713	–0.566839	–0.119022
C	0.241678	–1.009162	–1.375517
N	–0.965350	–0.219041	–1.687570
C	–2.010529	–1.928386	1.281445
Li	–2.080475	–0.242016	0.074342
C	–4.615483	0.712637	0.722661
C	–4.633473	–1.173400	–0.647544
C	–1.736466	–0.914968	–2.716065
C	–0.651068	1.128806	–2.156662
C	–1.708579	2.773664	0.749084
C	–1.576313	1.205486	2.497929
O	–1.641619	1.406073	1.094636
O	–4.031513	0.09213	–0.408832
Si	2.130215	–1.963901	0.551318
C	1.255098	–3.625700	0.429986
C	2.498742	–1.547692	2.349903
C	3.73954	–2.046319	–0.431611
H	3.860451	4.028025	–0.315400
H	2.39687	3.526547	1.634859
H	1.084667	1.424532	1.690059
H	2.716979	0.298273	–2.110256
H	4.010581	2.390651	–2.180991
H	0.240291	–0.464703	0.674175
H	–0.114822	–2.030970	–1.209320
H	0.908582	–1.031751	–2.259456
H	–4.380528	0.140777	1.631186
H	–4.199429	1.718095	0.801556
H	–5.704741	0.779711	0.598733
H	–5.701944	–1.040479	–0.865293
H	–4.138719	–1.615707	–1.512405
H	–4.495628	–1.830802	0.218454
H	–1.990002	–1.919754	–2.364425
H	–2.661023	–0.363564	–2.914871
H	–1.170344	–1.004336	–3.660423
H	–0.148146	1.700497	–1.375361
H	0.004538	1.108222	–3.045831
H	–1.584139	1.636311	–2.424573
H	–0.743988	3.26472	0.937434
H	–1.948219	2.836628	–0.313690
H	–2.492947	3.282121	1.328131
H	–1.456813	0.132726	2.657879
H	–0.731518	1.763438	2.924982
H	–2.506021	1.552715	2.971179

H	1.196285	–3.978151	–0.606482
H	1.808529	–4.380244	1.001865
H	0.236984	–3.572766	0.832195
H	2.975939	–0.565351	2.442273
H	1.572545	–1.535736	2.936886
H	3.169267	–2.291096	2.795807
H	4.336283	–1.135085	–0.316749
H	4.344181	–2.893360	–0.086255
H	3.55059	–2.193397	–1.502092
H	–2.851447	–1.970392	2.004241
H	–1.108532	–2.063812	1.912272
H	–2.093463	–2.875015	0.710545

Table S26 Coordinates of QM22.

Element	X	Y	Z
C	2.131669	3.679938	0.281933
C	1.629347	2.903077	1.326644
C	1.277222	1.573314	1.107552
C	1.440916	0.97621	–0.151819
C	1.946427	1.769678	–1.191006
C	2.278761	3.107926	–0.981693
H	2.40571	4.717437	0.450468
H	1.507412	3.336129	2.316661
H	0.883477	0.968215	1.923624
H	2.089089	1.334005	–2.178617
H	2.668183	3.700589	–1.805360
C	1.204405	–0.506328	–0.293612
C	0.497053	–0.999383	–1.557802
H	0.509355	–2.095709	–1.532312
H	1.03367	–0.706526	–2.477732
N	–0.922787	–0.588429	–1.663064
H	0.570335	–0.827411	0.545798
C	–1.355867	–1.617300	1.808214
C	–0.340969	–1.371149	2.930521
H	–0.330503	–2.163767	3.706743
H	0.69611	–1.332908	2.550738
H	–0.509751	–0.418380	3.457184
C	–1.076257	–3.011770	1.232551
H	–1.886370	–3.379983	0.583202
H	–0.165771	–3.017362	0.608899
H	–0.912484	–3.799765	1.998436
Li	–1.764787	–0.244396	0.244217
C	–4.519610	0.566803	–0.844679
H	–5.469618	0.723308	–0.315400
H	–3.964598	1.506047	–0.876314
H	–4.736419	0.223962	–1.868011
C	–4.377655	–1.604634	0.046128

H	–4.619249	–2.080179	–0.915633
H	–3.697993	–2.236767	0.617757
H	–5.305007	–1.449977	0.613638
C	–1.719250	–1.660055	–2.260792
H	–1.710043	–2.534043	–1.602400
H	–2.753350	–1.320967	–2.376913
H	–1.334405	–1.951324	–3.254081
C	–1.064470	0.622837	–2.466251
H	–0.460611	1.42466	–2.038855
H	–0.744246	0.458822	–3.511551
H	–2.114944	0.935534	–2.472675
C	–1.721043	2.998137	0.02915
H	–0.733881	3.379676	0.31704
H	–1.762202	2.883467	–1.054824
H	–2.497481	3.710119	0.344001
C	–2.134732	1.818615	2.017417
H	–2.279311	0.801457	2.387502
H	–1.243340	2.258214	2.485412
H	–3.011592	2.439235	2.248747
O	–1.959691	1.732062	0.610914
O	–3.719261	–0.369183	–0.160661
H	–2.345157	–1.672137	2.311332
Si	2.890155	–1.393086	–0.056074
C	2.62881	–3.221223	0.315613
H	2.054822	–3.735352	–0.463543
H	3.600648	–3.723358	0.396506
H	2.097888	–3.356974	1.264408
C	3.813701	–0.593384	1.375243
H	4.051877	0.45439	1.160361
H	3.220005	–0.619717	2.296517
H	4.752742	–1.126189	1.565626
C	3.918193	–1.215768	–1.631265
H	4.108182	–0.163192	–1.870441
H	4.891384	–1.703526	–1.500145
H	3.430848	–1.680624	–2.496281

Table S27 Coordinates of QM23.

Element	X	Y	Z
C	2.03124	3.703499	0.166935
C	1.784348	2.875326	1.26178
C	1.420058	1.542494	1.074005
C	1.316648	0.967439	–0.212220
C	1.567163	1.83113	–1.299761
C	1.914798	3.167339	–1.117654
H	2.316727	4.741722	0.309634
H	1.872709	3.269546	2.271897
H	1.228403	0.909397	1.936744

H	1.54252	1.434454	–2.312773
H	2.123018	3.789033	–1.985514
C	1.057198	–0.497469	–0.329907
C	0.403259	–0.981794	–1.620646
H	0.432222	–2.076647	–1.617793
H	0.917162	–0.672320	–2.553264
N	–1.046795	–0.610585	–1.758665
H	0.114783	–0.819475	0.702533
C	–0.792961	–1.326926	1.757702
C	–0.147964	–0.951175	3.088703
H	–0.415026	–1.641845	3.906651
H	0.947976	–0.974931	3.010291
H	–0.421035	0.060551	3.416061
C	–0.566649	–2.812066	1.482408
H	–1.021074	–3.144391	0.536528
H	0.511309	–3.010306	1.389379
H	–0.939906	–3.474994	2.280354
Li	–1.557513	–0.074520	0.130705
C	–4.484126	0.234654	–0.546491
H	–5.399616	0.331047	0.052795
H	–4.021786	1.214783	–0.669646
H	–4.744877	–0.186049	–1.528758
C	–4.056444	–1.886328	0.363902
H	–4.342064	–2.375058	–0.577932
H	–3.269882	–2.463194	0.853285
H	–4.934252	–1.830549	1.021084
C	–1.828418	–1.753775	–2.227678
H	–1.770377	–2.568798	–1.500032
H	–2.878064	–1.461969	–2.341357
H	–1.462979	–2.124574	–3.200669
C	–1.240832	0.498034	–2.692194
H	–0.647800	1.357871	–2.380760
H	–0.940538	0.221227	–3.718381
H	–2.299267	0.785463	–2.708384
C	–1.612327	2.989788	–0.082641
H	–0.610868	3.284717	0.248528
H	–1.603831	2.815575	–1.158616
H	–2.334901	3.783554	0.151711
C	–2.145812	1.936869	1.94841
H	–2.470914	0.977236	2.355702
H	–1.182593	2.222033	2.390591
H	–2.897602	2.706292	2.169765
O	–2.013716	1.780647	0.544178
O	–3.546127	–0.589556	0.115129
H	–1.880877	–1.155088	1.861391
Si	2.701969	–1.384060	–0.083485
C	2.635309	–3.152932	–0.768713
H	2.579892	–3.156008	–1.864092
H	3.551869	–3.684967	–0.486066
H	1.785655	–3.729473	–0.385612
C	3.301054	–1.482161	1.706874
H	3.27524	–0.504162	2.201348
H	2.708665	–2.183132	2.304618
H	4.342247	–1.829343	1.715421

C	4.077701	–0.495995	–1.044295
H	4.276304	0.496107	–0.620964
H	5.011883	–1.070444	–1.016580
H	3.803351	–0.352829	–2.097352

Table S28 Coordinates of QM24.

Element	X	Y	Z
C	–0.861884	–3.459104	0.715586
C	–1.084322	–2.219831	0.117751
C	–2.050549	–1.339651	0.610808
C	–2.813534	–1.753459	1.712164
C	–2.603831	–2.994674	2.307596
C	–1.620504	–3.853244	1.815941
H	–0.083059	–4.110365	0.326896
H	–0.496159	–1.921980	–0.747919
H	–3.571951	–1.088061	2.121211
H	–3.202279	–3.288075	3.165712
H	–1.446679	–4.815888	2.287635
C	–2.248908	0.027694	–0.005974
H	–1.400980	0.211099	–0.684448
C	–2.154418	1.081083	1.128442
H	–3.143006	1.271326	1.590646
H	–1.507461	0.663339	1.902971
N	–1.538494	2.356909	0.744209
C	–2.457500	3.158289	–0.053030
H	–1.970706	4.083713	–0.374382
H	–2.753579	2.610208	–0.947653
H	–3.363423	3.421534	0.523291
C	–1.186513	3.090023	1.962473
H	–0.803341	4.079514	1.697616
H	–2.062493	3.216897	2.624088
H	–0.399748	2.547879	2.495624
O	3.926395	–0.791245	–0.793554
C	–0.222313	2.171985	–2.449631
H	–0.149437	3.121308	–1.897700
H	–0.142734	2.431623	–3.531840
H	–1.240921	1.793236	–2.298684
C	0.628471	–0.068261	–2.912252
H	0.640387	0.18832	–3.997487
H	1.418842	–0.831565	–2.791391
H	–0.339503	–0.566629	–2.727878
C	4.71738	0.19862	–0.164234
H	4.870156	1.054858	–0.835495
H	4.171908	0.527672	0.72286
H	5.691563	–0.216284	0.129241
C	4.504851	–1.259403	–1.994235
H	5.453348	–1.775488	–1.788560
H	3.794343	–1.954039	–2.446894
H	4.687223	–0.428182	–2.688755
O	1.199513	0.534829	1.399682
C	1.536606	–0.066485	2.603023
C	0.290793	–0.394059	3.444022

H	0.552604	–0.910936	4.376329
H	–0.247579	0.524626	3.707825
H	–0.381752	–1.040227	2.868102
C	2.271308	–1.399261	2.34025
H	3.164273	–1.225492	1.723593
H	2.592795	–1.895608	3.264611
H	1.600083	–2.089700	1.807956
C	2.458036	0.84595	3.43423
H	2.668684	0.427129	4.426372
H	3.418589	0.998795	2.924998
H	1.983046	1.826295	3.56372
C	0.83958	1.162469	–2.027106
Li	0.494161	1.702783	0.167628
O	1.414846	3.483287	0.382472
C	2.751799	3.316857	0.813194
H	2.822752	2.291446	1.176536
H	3.448458	3.480727	–0.021738
H	2.98175	4.023131	1.623745
C	1.183583	4.756692	–0.173618
H	1.785151	4.901607	–1.082206
H	0.125481	4.819297	–0.434195
H	1.428297	5.543758	0.55421
C	2.782788	–3.732508	–0.482239
H	3.281883	–3.115949	0.266323
H	2.290076	–4.584540	0.006845
H	3.525881	–4.113350	–1.197680
O	1.830189	–2.919822	–1.141279
C	1.193502	–3.607739	–2.201223
H	0.698111	–4.516799	–1.833190
H	0.4512	–2.937367	–2.636930
H	1.926761	–3.886926	–2.971343
C	2.188599	1.758395	–2.431031
H	2.390408	2.70842	–1.916087
H	3.03201	1.088047	–2.208236
H	2.240682	1.975793	–3.524442
Na	1.649513	–0.627016	–0.420436
Si	–3.857523	–0.047813	–1.073562
C	–3.742444	0.840904	–2.737404
H	–2.881897	0.484638	–3.315511
H	–4.646417	0.599193	–3.310800
H	–3.674177	1.931166	–2.680203
C	–5.313224	0.640094	–0.087828
H	–5.203684	1.710675	0.117815
H	–6.243427	0.502245	–0.651505
H	–5.429660	0.124811	0.872948
C	–4.208583	–1.850609	–1.508235
H	–3.358740	–2.297603	–2.038477
H	–4.401243	–2.464119	–0.621954
H	–5.083208	–1.910290	–2.167025

Table S29 Coordinates of QM25.

Element	X	Y	Y
C	–1.183534	–2.394498	3.094764

C	–1.644273	–1.286930	2.393736
C	–1.541108	–1.180558	0.985402
C	–0.961580	–2.294468	0.335962
C	–0.510738	–3.414790	1.037941
C	–0.604258	–3.473669	2.424599
H	–1.297417	–2.423562	4.175885
H	–2.141588	–0.504586	2.959675
H	–0.903677	–2.284644	–0.751396
H	–0.087626	–4.253620	0.492937
H	–0.254761	–4.345406	2.969824
C	–2.079438	–0.037751	0.182686
H	–1.196919	0.126654	–0.897264
C	–2.268912	1.236005	1.0187
H	–3.336233	1.490314	1.20003
H	–1.820584	1.114708	2.008228
N	–1.623834	2.464989	0.481003
C	–2.303391	2.888448	–0.738767
H	–1.752695	3.697477	–1.229436
H	–2.363315	2.042635	–1.419537
H	–3.326731	3.241085	–0.521822
C	–1.728879	3.526987	1.482387
H	–1.260734	4.443653	1.111991
H	–2.783900	3.746089	1.723028
H	–1.218053	3.226711	2.40104
O	3.422406	–0.678412	–1.223353
C	–1.361233	0.615094	–3.275456
H	–1.725723	1.643473	–3.172465
H	–0.950924	0.542592	–4.303080
H	–2.236127	–0.048656	–3.240063
C	0.299042	–1.064481	–2.759979
H	0.721027	–0.925592	–3.779511
H	1.121484	–1.481976	–2.165878
H	–0.461963	–1.853416	–2.851471
C	4.156816	0.496987	–0.946239
H	4.013135	1.239514	–1.745253
H	3.764138	0.891995	–0.009329
H	5.228527	0.268502	–0.857019
C	3.754903	–1.211074	–2.487845
H	4.833848	–1.414910	–2.547253
H	3.19822	–2.140732	–2.614059
H	3.471967	–0.511164	–3.286954
O	1.561047	1.038717	1.04603
C	1.985941	0.867808	2.363483
C	0.877214	1.254834	3.351985
H	1.182346	1.111658	4.396376
H	0.621159	2.313041	3.215059
H	–0.013328	0.646453	3.165846
C	2.368587	–0.605362	2.592852
H	3.166438	–0.886922	1.890339
H	2.735734	–0.795751	3.608499
H	1.496872	–1.257544	2.431803
C	3.211248	1.751916	2.659959
H	3.550438	1.643864	3.697565
H	4.052103	1.492647	2.005146

H	2.955537	2.805337	2.492909
C	–0.307486	0.238036	–2.234242
Li	0.371906	2.102249	0.241639
O	1.193299	3.918741	–0.192814
C	2.576634	3.70406	–0.433053
H	2.857493	2.824841	0.145523
H	2.750325	3.52242	–1.503761
H	3.154278	4.580985	–0.111994
C	0.711582	5.008726	–0.952943
H	0.748682	4.777734	–2.027057
H	–0.319721	5.202584	–0.657773
H	1.314381	5.904666	–0.754812
C	3.360603	–3.485869	–0.051293
H	3.692121	–2.659145	0.576789
H	3.257111	–4.396354	0.55497
H	4.108205	–3.660086	–0.839465
O	2.117262	–3.123279	–0.614277
C	1.617908	–4.124028	–1.474668
H	1.52424	–5.083634	–0.947157
H	0.636057	–3.802624	–1.827529
H	2.285626	–4.261267	–2.338541
C	0.763219	1.327126	–2.254058
H	1.238829	1.434231	–3.255262
H	0.3286	2.315032	–2.026601
H	1.591443	1.160019	–1.546341
Si	–3.728190	–0.655439	–0.510261
C	–3.549010	–2.172847	–1.627788
H	–4.507384	–2.369201	–2.124662
H	–2.790582	–2.039531	–2.406338
H	–3.285871	–3.062895	–1.045991
C	–4.859780	–1.178340	0.920121
H	–4.420799	–2.012909	1.479656
H	–5.023781	–0.355423	1.627274
H	–5.841363	–1.498258	0.549393
C	–4.704281	0.656723	–1.470751
H	–4.185409	1.024246	–2.362313
H	–5.649667	0.20532	–1.797670
H	–4.956842	1.518757	–0.842948
Na	1.286567	–0.853601	–0.088155

Table S30 Coordinates of QM26.

Element	X	Y	Z
C	0.454882	–0.390095	–2.680965
C	–0.654400	0.131076	–2.020303
C	–1.514664	–0.661949	–1.175730
C	–1.062323	–2.029430	–1.043840
C	0.044728	–2.529890	–1.703044
C	0.840362	–1.722583	–2.539653
H	1.036678	0.270422	–3.321291
H	–0.891489	1.177296	–2.201603
H	–1.645515	–2.714777	–0.438521
H	0.286341	–3.584373	–1.579050
H	1.694222	–2.123923	–3.076631

C	–2.968894	1.311863	–0.794381
H	–2.888013	1.553511	–1.866319
H	–4.004344	1.575687	–0.508171
N	–2.054565	2.275867	–0.096190
C	–2.197024	3.610869	–0.668994
H	–3.231939	3.983098	–0.571139
H	–1.526158	4.311137	–0.160107
H	–1.937135	3.589273	–1.731886
C	–2.372444	2.326331	1.329889
H	–1.639904	2.953227	1.852304
H	–3.379584	2.743695	1.505785
H	–2.342449	1.311905	1.737046
C	–2.658014	–0.139466	–0.525640
C	2.206753	2.639451	–0.692994
O	1.548687	1.598273	–0.051301
C	1.372779	3.181642	–1.866815
H	1.856067	4.032918	–2.362596
H	1.211381	2.39574	–2.612502
H	0.39328	3.512323	–1.500959
C	3.558302	2.153878	–1.245495
H	4.104457	2.944637	–1.774646
H	4.19455	1.790396	–0.428435
H	3.387793	1.325036	–1.945661
C	2.446628	3.79268	0.298669
H	2.955197	4.647615	–0.164246
H	1.482365	4.136096	0.695059
H	3.055595	3.451305	1.144971
O	3.253425	–2.381551	–0.409539
C	3.175176	–3.700928	–0.903644
H	4.110157	–4.241087	–0.698927
H	2.350169	–4.195953	–0.388865
H	2.984532	–3.707983	–1.985468
C	4.331229	–1.662926	–0.972650
H	4.195342	–1.540344	–2.056772
H	4.353213	–0.678424	–0.496954
H	5.283268	–2.178194	–0.782497
Li	–0.244882	1.302001	–0.008145
O	–0.000134	0.102935	1.679106
C	0.533058	0.925185	2.710393
H	1.118345	1.702183	2.216134
H	–0.286949	1.363505	3.295651
H	1.172062	0.326838	3.372352
C	–0.775062	–0.953941	2.220839
H	–1.614545	–0.555648	2.80679
H	–1.160821	–1.538095	1.387809
H	–0.145132	–1.577113	2.871407
O	3.016888	–0.696223	1.850861
C	3.750621	0.469045	2.173018
H	4.803541	0.356121	1.873079
H	3.285709	1.286683	1.619154
H	3.709909	0.666066	3.254357
C	3.516034	–1.845625	2.496847
H	3.481428	–1.718055	3.588384
H	2.89056	–2.689328	2.202304

H	4.55293	–2.046993	2.190635
Na	1.648858	–0.628899	–0.043025
Si	–4.069623	–1.070787	0.213409
C	–3.790413	–2.874156	0.744536
H	–4.708866	–3.214207	1.239988
H	–3.616696	–3.540856	–0.107057
H	–2.969326	–3.007787	1.458888
C	–4.749954	–0.208699	1.768094
H	–5.019582	0.837036	1.58001
H	–5.658544	–0.724283	2.104485
H	–4.034595	–0.222684	2.599898
C	–5.540157	–1.139548	–0.987907
H	–5.268746	–1.689954	–1.896233
H	–6.409894	–1.632989	–0.535622
H	–5.849110	–0.133029	–1.295422

Table S31 Coordinates of QM27.

Element	X	Y	Z
C	–0.583566	–2.958935	1.67445
C	–1.398371	–1.858147	1.406091
C	–1.728938	–1.500884	0.090379
C	–1.239994	–2.311448	–0.944794
C	–0.433972	–3.417731	–0.683511
C	–0.093889	–3.744370	0.630235
H	–0.337146	–3.203743	2.704531
H	–1.783831	–1.270647	2.234636
H	–1.486596	–2.056825	–1.973441
H	–0.063445	–4.017257	–1.511076
H	0.540339	–4.602110	0.838373
C	–2.657306	–0.356232	–0.229675
H	–2.474473	–0.087693	–1.276842
C	–2.477355	0.905705	0.644998
H	–3.417481	1.164526	1.175923
H	–1.715133	0.725699	1.408366
N	–2.025343	2.09681	–0.094472
C	–2.992097	2.488261	–1.115823
H	–2.645358	3.397305	–1.614366
H	–3.098951	1.714502	–1.879262
H	–3.984041	2.695598	–0.675195
C	–1.889004	3.19705	0.86426
H	–1.661437	4.12278	0.330163
H	–2.822440	3.342254	1.439292
H	–1.061917	2.976148	1.543215
O	4.018158	–0.138926	–0.090198
C	–0.291119	1.094122	–3.079904
H	–0.692839	2.086284	–2.821216
H	–0.108330	1.106134	–4.181618
H	–1.105716	0.372475	–2.908296

C	1.418085	–0.636176	–2.775687
H	1.560775	–0.655189	–3.880962
H	2.391785	–0.935789	–2.348519
H	0.700415	–1.441698	–2.551998
C	4.090354	1.223661	0.283912
H	4.357241	1.850416	–0.577412
H	3.094549	1.50637	0.639643
H	4.834737	1.36792	1.081628
C	5.149962	–0.569881	–0.808722
H	6.060279	–0.487471	–0.196173
H	4.978492	–1.613323	–1.081208
H	5.27748	0.026816	–1.723124
O	0.744354	1.286222	1.374041
C	1.05746	1.265601	2.721471
C	–0.199954	1.14934	3.610092
H	0.056131	1.135143	4.677228
H	–0.880734	1.990191	3.436699
H	–0.738013	0.222338	3.382322
C	1.958338	0.052393	3.061976
H	2.864769	0.057087	2.441725
H	2.270705	0.047745	4.113682
H	1.411153	–0.884920	2.880572
C	1.804124	2.546383	3.146848
H	2.011578	2.565022	4.224225
H	2.76304	2.62951	2.620459
H	1.197289	3.425284	2.896789
C	0.964847	0.742778	–2.285101
Li	0.226805	1.754187	–0.354111
O	0.615194	3.734892	–0.691321
C	1.803125	4.167447	–0.061370
H	2.034675	3.425608	0.70199
H	2.622597	4.235083	–0.791866
H	1.652186	5.149873	0.409084
C	0.182789	4.632048	–1.687944
H	0.960643	4.777261	–2.450220
H	–0.698893	4.200331	–2.163248
H	–0.072582	5.6052	–1.243022
K	1.456049	–0.905768	0.117013
C	3.729039	–3.410115	0.753462
H	3.750865	–2.550838	1.429229
H	3.210849	–4.250249	1.238809
H	4.761728	–3.712241	0.525488
O	3.053225	–3.005903	–0.414680
C	2.995121	–4.013801	–1.396463
H	2.445169	–4.892831	–1.026955
H	2.476434	–3.591703	–2.260054

H	4.006382	–4.326925	–1.694143
C	2.069918	1.694333	–2.745779
H	1.867245	2.743444	–2.497156
H	3.041084	1.436792	–2.297703
H	2.216951	1.656014	–3.852267
Si	–4.448647	–1.037384	–0.149471
C	–5.691266	0.288792	–0.651201
H	–6.697873	–0.146322	–0.667125
H	–5.706080	1.129415	0.050415
H	–5.485281	0.68591	–1.651368
C	–4.815969	–1.588712	1.615044
H	–4.624275	–0.788858	2.340296
H	–5.870336	–1.874082	1.709289
H	–4.208532	–2.455216	1.897426
C	–4.618210	–2.501772	–1.321273
H	–4.363768	–2.222114	–2.350506
H	–3.967625	–3.332649	–1.027884
H	–5.651693	–2.867593	–1.322843

Table S32 Coordinates of QM28.

Element	X	Y	Z
C	0.398835	–2.721031	2.366773
C	–0.576063	–1.785541	2.018562
C	–1.128307	–1.719722	0.720125
C	–0.677989	–2.718006	–0.180709
C	0.274906	–3.668025	0.16959
C	0.848203	–3.666025	1.444038
H	0.796802	–2.717395	3.379252
H	–0.924520	–1.107053	2.788868
H	–1.082874	–2.728876	–1.187237
H	0.57732	–4.411506	–0.564485
H	1.598561	–4.401690	1.721714
C	–2.094399	–0.687359	0.248014
H	–1.377223	–0.130077	–0.831706
C	–2.281308	0.457696	1.257328
H	–3.139003	0.310031	1.949346
H	–1.387690	0.539649	1.883893
N	–2.410684	1.808874	0.649659
C	–3.673777	1.951697	–0.061075
H	–3.739073	2.941881	–0.522091
H	–3.747065	1.203171	–0.847440
H	–4.529237	1.839128	0.626302
C	–2.353341	2.82363	1.701109
H	–2.500290	3.814674	1.2599
H	–3.131231	2.659625	2.468185
H	–1.370390	2.804369	2.177527
O	3.786262	0.444887	–1.152199
C	–1.868888	1.143989	–2.743566
H	–2.024704	2.114385	–2.252642
H	–1.739038	1.344067	–3.825947

H	–2.800517	0.57166	–2.635784
C	–0.513629	–0.883643	–2.994652
H	–0.391542	–0.645427	–4.071004
H	0.367978	–1.478458	–2.705955
H	–1.389067	–1.540019	–2.916452
C	3.66814	1.853523	–1.076464
H	3.490462	2.281735	–2.073895
H	2.810258	2.063884	–0.430328
H	4.580967	2.294538	–0.649471
C	4.804563	0.034948	–2.037995
H	5.782434	0.41338	–1.706965
H	4.808514	–1.056344	–2.040343
H	4.604673	0.404611	–3.053650
O	0.947779	1.589168	1.050717
C	1.666783	1.744134	2.229731
C	0.794468	1.509129	3.477083
H	1.387474	1.558592	4.399343
H	0.001249	2.260037	3.55115
H	0.33007	0.519398	3.423696
C	2.830357	0.728222	2.301374
H	3.46187	0.799409	1.40641
H	3.467982	0.896439	3.177764
H	2.436919	–0.296310	2.381974
C	2.262099	3.161204	2.337977
H	2.779549	3.315982	3.292954
H	2.984991	3.344735	1.532839
H	1.457963	3.902955	2.257542
C	–0.678892	0.388091	–2.163451
Li	–0.449623	1.949877	–0.028132
O	–0.392165	3.909036	–0.584180
C	0.914222	4.448446	–0.672947
H	1.552461	3.810956	–0.063302
H	1.261015	4.448131	–1.716688
H	0.927894	5.476566	–0.286677
C	–1.302771	4.606512	–1.408011
H	–1.029909	4.495661	–2.467003
H	–2.291571	4.177997	–1.245179
H	–1.316527	5.672554	–1.143839
K	1.525714	–0.686533	–0.150069
C	4.343494	–2.404702	0.45726
H	4.370348	–1.342416	0.713767
H	3.985885	–2.985919	1.319307
H	5.359095	–2.735635	0.195788
O	3.469116	–2.554610	–0.641934
C	3.446681	–3.877269	–1.129858
H	3.135307	–4.581059	–0.344670
H	2.729099	–3.912239	–1.951901
H	4.440299	–4.169468	–1.498800
C	0.567584	1.243475	–2.442570
H	0.608142	1.52557	–3.516385
H	0.608646	2.190764	–1.888720
H	1.515709	0.723279	–2.250151
Si	–3.685537	–1.672287	–0.150275
C	–3.803522	–2.374251	–1.907219

H	–4.778315	–2.867859	–2.013355
H	–3.741556	–1.592694	–2.672595
H	–3.034965	–3.123075	–2.126177
C	–3.823093	–3.143878	1.041531
H	–3.015983	–3.871751	0.909164
H	–3.782967	–2.797310	2.082561
H	–4.778335	–3.663792	0.898749
C	–5.334348	–0.761853	0.104264
H	–5.573443	–0.036853	–0.679547
H	–6.114864	–1.533148	0.08391
H	–5.403393	–0.257239	1.073741

Table S33 Coordinates of QM29.

Element	X	Y	Z
C	0.294843	–1.123502	–2.574799
C	–0.678691	–0.330277	–1.975635
C	–1.644827	–0.855183	–1.041173
C	–1.387712	–2.228753	–0.671861
C	–0.404659	–3.000557	–1.266912
C	0.458862	–2.471251	–2.243625
H	0.942952	–0.664910	–3.320209
H	–0.747261	0.705136	–2.303232
H	–2.039801	–2.703692	0.05873
H	–0.309597	–4.044426	–0.971778
H	1.209325	–3.089476	–2.727036
C	–2.893070	1.288725	–1.059445
H	–2.697586	1.34187	–2.142516
H	–3.915068	1.676375	–0.917198
N	–1.956271	2.290243	–0.441418
C	–1.970817	3.518754	–1.232317
H	–2.989741	3.938241	–1.301393
H	–1.316768	4.271146	–0.781454
H	–1.615164	3.311364	–2.246352
C	–2.395181	2.590335	0.921607
H	–1.665302	3.24291	1.415773
H	–3.376891	3.096403	0.923376
H	–2.485299	1.653923	1.480802
C	–2.743697	–0.114023	–0.540541
C	2.21183	2.590089	–0.752433
O	1.609893	1.491548	–0.154753
C	1.764085	2.709289	–2.220098
H	2.234566	3.553187	–2.740559
H	2.008635	1.785521	–2.759452
H	0.675539	2.842553	–2.266080
C	3.745583	2.463002	–0.706613
H	4.246098	3.295912	–1.215962
H	4.091829	2.44721	0.334071

H	4.062311	1.527527	–1.181523
C	1.820136	3.880444	–0.007127
H	2.229545	4.783636	–0.477192
H	0.726704	3.973065	0.023882
H	2.184722	3.838987	1.027205
O	3.808612	–1.839619	–1.046664
C	4.618924	–2.989039	–1.136266
H	5.656724	–2.718359	–1.374977
H	4.591026	–3.487598	–0.165292
H	4.240606	–3.673820	–1.908246
C	3.78522	–1.106042	–2.255414
H	3.432887	–1.734018	–3.085823
H	3.095549	–0.268888	–2.116645
H	4.785812	–0.717540	–2.491162
Li	–0.200099	1.273316	–0.083183
O	–0.128464	0.490807	1.801205
C	0.445986	1.418372	2.709001
H	1.087804	2.072969	2.117375
H	–0.347267	1.992355	3.207778
H	1.038482	0.884457	3.464032
C	–0.946889	–0.455943	2.46439
H	–1.724425	0.050363	3.051617
H	–1.424485	–1.067021	1.698873
H	–0.332169	–1.072279	3.136531
K	1.567393	–1.056763	0.215622
O	2.748406	–0.880213	2.632452
C	3.532856	0.274597	2.375972
H	4.55709	–0.009971	2.097108
H	3.057086	0.819466	1.552724
H	3.569221	0.918978	3.266487
C	3.226516	–1.621202	3.731561
H	3.199087	–1.017168	4.649501
H	2.576955	–2.490468	3.852568
H	4.258599	–1.957967	3.559382
Si	–4.222241	–0.947850	0.189855
C	–5.635820	0.297553	0.411222
H	–6.480109	–0.201109	0.902567
H	–5.344084	1.149323	1.037847
H	–5.996995	0.691271	–0.545635
C	–4.045311	–1.738391	1.918994
H	–3.859754	–0.977973	2.687176
H	–4.990817	–2.234530	2.173342
H	–3.255533	–2.495081	1.995326
C	–4.893992	–2.330953	–0.924630
H	–5.212122	–1.913558	–1.887222
H	–4.141066	–3.098054	–1.137180

H	−5.759368	−2.825965	−0.466332

4.1.3 Deprotonation of *N,N*-dimethyl-2-phenylethan-1-amine (12)

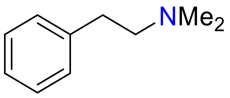

12

Figure S32 Lewis formula of *N,N*-dimethyl-2-phenylethan-1-amine (**12**).

In the following, the energies (Table S34) and the coordinates of the opitimized structures for the deprotonation reaction of (**12**) with various lithium alkyls (*t*-BuLi/MeLi/*i*-PrLi; Scheme S7) and the two Schlosser bases *t*-BuONa/*t*-BuLi and *t*-BuOK/*t*-BuLi (Scheme S8) are listed.

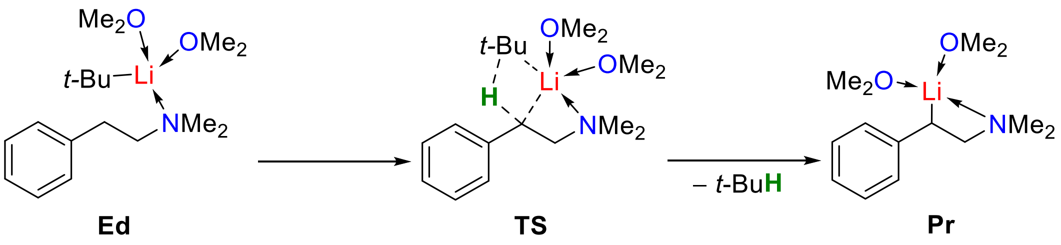

Scheme S7 Calculated reaction scheme for the deprotonation of the (**12**) with different lithium alkyls; exemplary for *t*-BuLi; with stationary points: Ed = educt; TS = transition state; Pr = product.

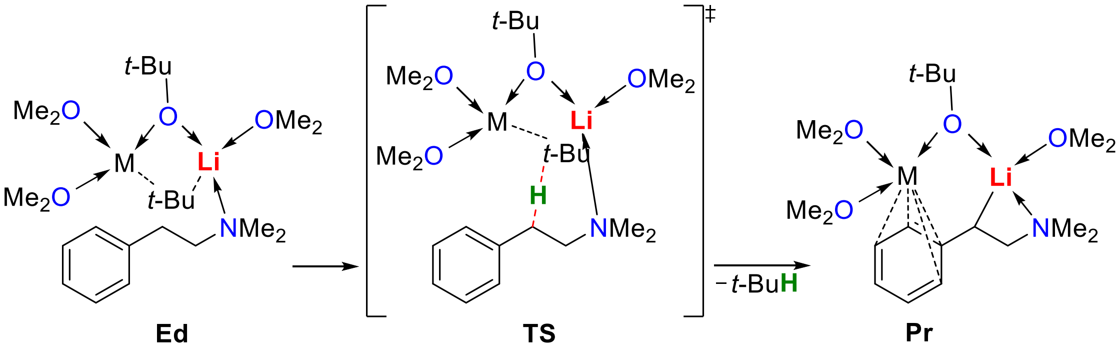

M= Na, K

Scheme S8 Calculated reaction scheme for the deprotonation of the **12** with the Schlosser-base mixture *t*-BuOM/*t*-BuLi; with stationary points: Ed = educt; TS = transition state; Pr = product.

Table S34 Total (SCF) and zero-point-corrected (ZPE) energies of the optimized structures and stationary points of the deprotonation reaction of **12**.

stationary point	Metalating reagent	calculation number	SCF [Hartree]	ZPE [Hartree]
Ed		QM30	–919.858312134	–919.33477
TS	*t*-BuLi	QM31	–919.833259772	–919.31405
Pr		QM32	–761.526355615	–761.137248
	t-BuH	QM4	–158.363419798	–158.230481
Ed		QM33	–801.985258937	–801.547762
TS	MeLi	QM34	-801.956069371	-801.523057
Pr		QM32	–761.526355615	–761.137248
	MeH	QM7	–40.4835867202	–40.438039
Ed		QM35	–880.563598083	–880.06878
TS	*i*-PrLi	QM36	–880.539650739	–880.048747
Pr		QM32	–761.526355615	–761.137248
	i-PrH	QM10	–119.067575926	–118.962723
Ed		QM37	–1470.14382443	–1469.409523
TS	*t*-BuONa/*t*-BuLi	QM38	–1470.11423727	–1469.385306
Pr		QM39	–1311.79579573	–1311.196753
Ed		QM40	–1907.75087857	–1907.017529
TS	*t*-BuOK/*t*-BuLi	QM41	–1907.73158159	–1907.002798
Pr		QM42	–1749.41775228	–1748.819491

Table S345 Coordinates of QM30.

Element	X	Y	Z
C	–4.424352	–1.068800	–0.309300
C	–3.315892	–1.624171	0.327326
C	–2.325738	–0.797721	0.859715
C	–2.430492	0.592462	0.769646
C	–3.558952	1.140041	0.148658
C	–4.544910	0.319349	–0.393663
C	–1.344145	1.465289	1.352122
C	–0.669146	2.453767	0.400788
N	0.147542	1.842069	–0.663889
C	1.781697	–0.352443	1.894815
C	1.009776	–0.937315	3.075692
C	2.237522	1.044919	2.319901
Li	1.097416	–0.002203	–0.087053
C	3.01447	–1.261297	–2.008758
C	3.807562	0.782815	–1.209012
C	1.120991	2.830139	–1.131580
C	–0.674686	1.433162	–1.804810
C	–0.548153	–1.949345	–2.134646
C	0.252676	–2.739679	–0.084319
O	0.126226	–1.612282	–0.939122
O	2.659605	–0.021627	–1.421888
C	3.042389	–1.204880	1.740795
H	–5.195905	–1.710925	–0.724674
H	–3.226063	–2.704481	0.416312

H	–1.454651	–1.223585	1.354717
H	–3.664911	2.221707	0.083626
H	–5.412960	0.761761	–0.874699
H	–0.560282	0.828556	1.775228
H	–1.764589	2.056102	2.177721
H	0.005768	3.064777	1.00909
H	–1.406471	3.142784	–0.052135
H	1.629004	–0.967154	4.001574
H	0.108858	–0.360175	3.334562
H	0.680499	–1.975606	2.904963
H	2.861336	1.540262	1.557091
H	1.389766	1.718161	2.525682
H	2.850154	1.029932	3.2518
H	3.598575	–1.862017	–1.298652
H	2.092396	–1.784804	–2.257983
H	3.603582	–1.094274	–2.920678
H	4.186168	1.160941	–2.168713
H	3.521186	1.616231	–0.568333
H	4.596655	0.208871	–0.708945
H	1.783684	3.112045	–0.308025
H	1.716783	2.399319	–1.941046
H	0.621586	3.74008	–1.508918
H	–1.379098	0.653368	–1.511441
H	–1.244007	2.286306	–2.215727
H	–0.015894	1.045444	–2.588818
H	–1.612443	–2.132528	–1.933051
H	–0.446946	–1.117025	–2.831505
H	–0.105132	–2.850019	–2.584006
H	0.681275	–2.378308	0.850226
H	–0.732110	–3.193713	0.091982
H	0.915994	–3.486669	–0.543657
H	2.81994	–2.249338	1.467001
H	3.726126	–0.814417	0.972612
H	3.63673	–1.255975	2.683077

Table S356 Coordinates of QM31.

Element	X	Y	Z
C	4.570966	–0.473683	–0.618725
C	4.212135	0.857845	–0.377503
C	3.057406	1.161352	0.328893
C	2.192259	0.158069	0.825796
C	2.59892	–1.173316	0.609555
C	3.759837	–1.481404	–0.102752
C	0.93236	0.56775	1.469513
C	0.167137	–0.402361	2.351641
N	–1.013161	–1.079861	1.715043
C	–1.003807	2.19791	0.101568
C	–0.055094	3.391048	0.144075
C	–1.958073	2.278105	1.28943
Li	–1.116455	–0.016578	–0.102203
C	–2.978700	–0.779851	–2.371775
C	–3.960545	–0.783190	–0.243920
C	–2.027469	–1.321568	2.741063

C	–0.638633	–2.362226	1.12028
C	0.169785	–1.879755	–2.177692
C	1.017053	0.307042	–2.132493
O	0.033385	–0.587396	–1.621602
O	–2.746352	–0.812687	–0.973082
C	–1.809175	2.297538	–1.197326
H	5.471874	–0.715099	–1.174672
H	4.842231	1.664295	–0.745349
H	2.78925	2.203726	0.495869
H	2.01843	–1.990981	1.026635
H	4.035268	–2.524566	–0.244084
H	0.028698	1.194378	0.598732
H	1.119828	1.481933	2.045226
H	–0.248994	0.171564	3.185789
H	0.804123	–1.188210	2.802912
H	–0.606257	4.34935	0.115851
H	0.55675	3.400749	1.055804
H	0.635086	3.393361	–0.712010
H	–2.722331	1.483493	1.26184
H	–1.417779	2.17089	2.242652
H	–2.503576	3.240009	1.334023
H	–3.644826	0.053214	–2.630429
H	–2.015682	–0.631110	–2.859600
H	–3.433469	–1.723835	–2.699860
H	–4.565854	–1.670746	–0.470560
H	–3.706007	–0.767512	0.815527
H	–4.532741	0.121207	–0.491052
H	–2.439678	–0.369026	3.088894
H	–2.838143	–1.935258	2.336434
H	–1.601299	–1.858493	3.605943
H	0.119574	–2.205768	0.351933
H	–0.232109	–3.060166	1.874024
H	–1.517985	–2.819132	0.651873
H	1.118284	–2.331953	–1.856959
H	–0.670566	–2.485072	–1.828856
H	0.148664	–1.827937	–3.275595
H	0.934099	1.235268	–1.567081
H	2.020049	–0.114438	–1.997087
H	0.823918	0.490938	–3.198738
H	–1.192005	2.143563	–2.094958
H	–2.636050	1.576761	–1.238522
H	–2.273716	3.298196	–1.303552

Table S367 Coordinates of QM32.

Element	X	Y	Z
C	–2.636327	–0.956334	1.004157
C	–1.424608	–1.304318	0.405238
C	–1.157110	–1.040329	–0.983756
C	–2.219955	–0.356641	–1.674095
C	–3.398261	0.001032	–1.053128
C	–3.636959	–0.289666	0.304354
H	–2.798093	–1.222153	2.048834
H	–0.690528	–1.851176	0.997753

H	–2.092837	–0.158485	–2.737777
H	–4.168091	0.502987	–1.636310
H	–4.575236	–0.022148	0.779075
C	1.102466	–2.148067	–0.888802
H	0.654726	–2.941204	–0.269288
H	1.793058	–2.645295	–1.590488
N	1.922282	–1.310311	0.064754
C	2.513331	–2.125923	1.116842
H	3.17371	–2.909884	0.706324
H	3.107198	–1.497844	1.790178
H	1.721859	–2.613875	1.695745
C	2.97444	–0.618108	–0.674665
H	3.489145	0.096593	–0.022072
H	3.719185	–1.327585	–1.077496
H	2.523746	–0.077642	–1.513892
Li	0.448116	0.151055	0.063641
C	0.068986	–1.347061	–1.602641
H	0.170235	–1.188395	–2.672692
O	0.853937	1.855531	–0.834537
O	0.280033	1.102471	1.790934
C	0.304713	0.283077	2.945108
H	0.473569	0.895347	3.840793
H	–0.640712	–0.264660	3.048209
H	1.125709	–0.426005	2.822462
C	–0.821751	2.002546	1.802306
H	–0.777793	2.636798	2.697331
H	–0.740862	2.617734	0.904916
H	–1.766677	1.445655	1.777573
C	1.891547	2.708766	–0.395855
H	2.057215	2.496216	0.662039
H	2.813002	2.521381	–0.964613
H	1.600596	3.760461	–0.521923
C	0.539113	2.028588	–2.205628
H	0.292458	3.080213	–2.402424
H	1.387486	1.72724	–2.834668
H	–0.321901	1.395339	–2.419568

Table S378 Coordinates of QM33.

Element	X	Y	Z
C	3.981249	1.212075	–0.395145
C	2.917415	1.642125	0.396125
C	2.049837	0.712915	0.971924
C	2.231927	–0.656457	0.762892
C	3.318758	–1.078809	–0.011195
C	4.183924	–0.155026	–0.592188
C	1.253094	–1.647425	1.34402
C	0.455113	–2.466795	0.324682
N	–0.440144	–1.698329	–0.560365
C	–1.621624	0.07754	2.456395
Li	–1.360685	–0.034228	0.396231
C	–3.674546	1.332118	–0.593254
C	–4.045006	–0.738305	0.418516
C	–1.465459	–2.600077	–1.092000

C	0.280989	–1.112643	–1.694156
C	–0.265883	2.270857	–1.575739
C	–0.679875	2.758634	0.674401
O	–0.699281	1.759211	–0.333744
O	–3.202943	0.004988	–0.452772
H	4.657854	1.934461	–0.843009
H	2.76881	2.705036	0.572393
H	1.210252	1.038218	1.583356
H	3.482711	–2.143678	–0.168935
H	5.020588	–0.498816	–1.194309
H	0.537854	–1.125434	1.988289
H	1.796944	–2.370120	1.967416
H	–0.176936	–3.150001	0.90154
H	1.128812	–3.091542	–0.291846
H	–3.591362	1.867267	0.363034
H	–3.058205	1.826867	–1.345039
H	–4.722637	1.328854	–0.921962
H	–5.038216	–0.855478	–0.036032
H	–3.587058	–1.716779	0.564478
H	–4.122193	–0.242234	1.392051
H	–2.008311	–3.068228	–0.267054
H	–2.174171	–2.031804	–1.701135
H	–1.018619	–3.398667	–1.710559
H	0.964328	–0.328264	–1.363807
H	0.860462	–1.878474	–2.240294
H	–0.449893	–0.680100	–2.384696
H	0.808592	2.499548	–1.542189
H	–0.452042	1.514805	–2.338947
H	–0.822715	3.184025	–1.830680
H	–0.974739	2.27296	1.605772
H	0.327943	3.187783	0.763631
H	–1.387091	3.561037	0.418763
H	–2.460652	0.735676	2.763922
H	–1.888449	–0.917093	2.867918
H	–0.770476	0.413216	3.082258

Table S389 Coordinates of QM34.

Element	X	Y	Z
C	–4.308962	–0.584210	–0.333585
C	–3.971180	–0.558502	1.024604
C	–2.845027	0.124804	1.460406
C	–1.988461	0.812955	0.56797
C	–2.374140	0.807387	–0.788200
C	–3.505723	0.119169	–1.228724
C	–0.756930	1.412784	1.099002
C	0.063176	2.382267	0.267436
N	1.22241	1.763238	–0.462154
C	1.037908	–0.404007	2.432572
Li	1.235878	–0.149685	0.312017
C	3.233093	–2.246213	–0.166632
C	3.996527	–0.256972	0.806553
C	2.367915	2.66943	–0.466790
C	0.882968	1.437849	–1.846354

C	0.129373	–1.895971	–1.937498
C	–0.821453	–2.212707	0.184339
O	0.196637	–1.557386	–0.568084
O	3.054464	–0.843677	–0.078232
H	–5.189236	–1.119291	–0.677177
H	–4.595699	–1.077623	1.748043
H	–2.593626	0.126055	2.520202
H	–1.802836	1.378889	–1.513799
H	–3.767311	0.147321	–2.284605
H	0.114141	0.439209	1.625485
H	–0.959767	1.851944	2.082815
H	0.503134	3.113772	0.952354
H	–0.530516	2.963846	–0.463738
H	3.063055	–2.716164	0.811216
H	2.505313	–2.628735	–0.881923
H	4.249666	–2.476171	–0.511489
H	5.011639	–0.366409	0.403541
H	3.745917	0.800747	0.896559
H	3.935626	–0.725432	1.796801
H	2.673913	2.886184	0.561041
H	3.206983	2.20298	–0.994193
H	2.131742	3.624475	–0.967740
H	0.05661	0.725759	–1.869727
H	0.585857	2.336349	–2.416403
H	1.751365	0.984175	–2.337467
H	–0.812449	–1.536739	–2.374548
H	0.975394	–1.424151	–2.442675
H	0.191949	–2.985995	–2.064506
H	–0.746545	–1.850780	1.209515
H	–1.809954	–1.965565	–0.221676
H	–0.657346	–3.298587	0.149321
H	1.535739	–1.358357	2.175843
H	1.79823	0.257275	2.877649
H	0.3212	–0.642478	3.230231

Table S40 Coordinates of QM35.

Element	X	Y	Z
C	4.282711	0.997631	0.038914
C	3.273295	1.15991	0.985794
C	2.253626	0.213065	1.095514
C	2.237706	–0.918016	0.274284
C	3.266778	–1.079246	–0.661269
C	4.275783	–0.127775	–0.787306
H	5.075333	1.735271	–0.049591
H	3.280132	2.025588	1.644213
H	1.459301	0.338093	1.830131
H	3.278145	–1.960853	–1.299952
H	5.064136	–0.268655	–1.521630
C	1.157132	–1.960034	0.450267
H	1.614373	–2.875663	0.850722
C	0.362133	–2.365119	–0.792645
H	–0.290718	–3.189723	–0.488828
H	1.020001	–2.757296	–1.589776

N	–0.504143	–1.305441	–1.340144
H	0.43629	–1.605030	1.194475
C	–1.796975	–0.657026	1.99882
C	–0.950068	–0.560811	3.275087
H	–1.463759	–0.941263	4.181713
H	–0.024610	–1.156691	3.198617
H	–0.632156	0.46721	3.509935
C	–2.253242	–2.118425	1.877463
H	–2.980472	–2.281238	1.065954
H	–1.402066	–2.788815	1.664435
H	–2.721425	–2.526373	2.797455
Li	–1.186054	0.055234	0.105064
C	–2.801078	2.149495	–1.391593
H	–2.894719	2.827021	–0.531068
H	–1.868917	2.36329	–1.917025
H	–3.649397	2.308047	–2.071338
C	–3.977629	0.39223	–0.375192
H	–4.790879	0.450842	–1.111388
H	–3.846243	–0.636393	–0.037230
H	–4.215424	1.019066	0.49334
C	–1.672790	–1.911446	–1.982121
H	–2.260486	–2.452512	–1.234474
H	–2.295192	–1.126189	–2.417785
H	–1.373900	–2.615827	–2.778295
C	0.20265	–0.481515	–2.319133
H	1.095516	–0.042937	–1.869281
H	0.510405	–1.072111	–3.201077
H	–0.462787	0.322315	–2.655700
C	0.577277	2.682156	–0.712913
H	1.551803	2.845664	–0.236041
H	0.7295	2.165143	–1.661158
H	0.09511	3.653584	–0.898442
C	–0.514480	2.492662	1.349008
H	–1.181225	1.826401	1.898844
H	0.419979	2.631816	1.911522
H	–0.987581	3.472818	1.19212
O	–0.246542	1.874867	0.100215
O	–2.756296	0.804579	–0.967439
H	–2.715167	–0.064207	2.202327

Table S391 Coordinates of QM37.

Element	X	Y	Z
C	3.775851	–0.704741	1.136403
C	2.521417	–1.024430	0.625463
C	2.383259	–1.930562	–0.433905
C	3.539036	–2.484512	–0.988841
C	4.80148	–2.153861	–0.491868
C	4.924279	–1.270415	0.578945
H	3.856633	–0.012976	1.972459
H	1.621273	–0.577933	1.04537
H	3.451429	–3.186689	–1.815375
H	5.688734	–2.597669	–0.935196

H	5.905467	−1.026006	0.976569
C	1.00121	−2.281432	−0.934707
H	0.492686	−1.373716	−1.293780
H	1.084072	−2.963134	−1.789132
C	0.13524	−2.888206	0.177106
H	0.464705	−3.921590	0.399174
H	0.261172	−2.292205	1.084209
N	−1.300812	−2.865397	−0.137716
C	−1.609946	−3.802795	−1.213395
H	−2.675303	−3.752165	−1.457690
H	−1.050476	−3.550230	−2.115023
H	−1.366556	−4.840454	−0.920880
C	−2.062981	−3.224466	1.05809
H	−3.128285	−3.275851	0.813993
H	−1.747906	−4.203054	1.462368
H	−1.921540	−2.452748	1.819052
O	−0.304978	3.543574	−0.082468
C	−1.820403	−0.816636	−2.916323
H	−2.683985	−1.288716	−2.422416
H	−2.161233	−0.528409	−3.937775
H	−1.062464	−1.600299	−3.056657
C	−0.077344	0.880176	−2.967891
H	−0.374259	1.134914	−4.011361
H	0.391011	1.799342	−2.570933
H	0.718362	0.123719	−3.054416
C	−1.498217	3.960297	0.551435
H	−2.317039	4.04476	−0.175674
H	−1.751671	3.201087	1.29503
H	−1.350481	4.928559	1.04876
C	0.081122	4.413905	−1.128511
H	0.24592	5.428702	−0.740699
H	1.009905	4.021906	−1.547730
H	−0.688695	4.441452	−1.911884
O	−0.720634	0.011817	1.334602
C	−0.495530	0.282897	2.677762
C	0.075308	−0.937856	3.429793
H	0.257534	−0.708158	4.487102
H	−0.627367	−1.777376	3.386019
H	1.024294	−1.267199	2.991934
C	0.517057	1.440311	2.820368
H	0.143088	2.337189	2.307385
H	0.709933	1.705304	3.867574
H	1.476642	1.163078	2.361465
C	−1.799901	0.690215	3.387032
H	−1.654800	0.837718	4.464656
H	−2.193861	1.625712	2.970699

H	–2.555132	–0.091593	3.240236
C	–1.268765	0.378475	–2.144281
Li	–1.604648	–0.714989	–0.134498
O	–3.591444	–0.599862	0.11263
C	–4.010874	0.602631	0.727072
H	–3.103682	1.134379	1.016273
H	–4.597284	1.211742	0.026058
H	–4.616032	0.386204	1.618328
C	–4.650100	–1.280865	–0.520619
H	–5.071528	–0.671511	–1.332473
H	–4.245258	–2.202944	–0.941921
H	–5.441472	–1.523091	0.203022
C	2.896977	2.910501	0.275354
H	2.120365	3.313647	0.929202
H	3.668496	2.409856	0.876818
H	3.36199	3.729915	–0.292203
O	2.27828	1.990241	–0.598996
C	3.192648	1.436205	–1.530584
H	4.039953	0.970782	–1.011452
H	2.65942	0.673624	–2.099034
H	3.556642	2.218172	–2.211707
C	–2.332239	1.47502	–2.215728
H	–3.285400	1.153628	–1.774752
H	–2.030112	2.396532	–1.692952
H	–2.557976	1.770461	–3.267610
Na	0.124401	1.285526	–0.253697

Table S402 Coordinates of QM38.

Element	X	Y	Z
C	3.030542	–1.536353	1.961707
C	1.715477	–1.602565	1.502419
C	1.38146	–2.209723	0.269409
C	2.466031	–2.742807	–0.468453
C	3.772708	–2.685218	–0.006038
C	4.075854	–2.076434	1.217019
H	3.231259	–1.065151	2.922513
H	0.927704	–1.181946	2.119208
H	2.255794	–3.236364	–1.416320
H	4.568179	–3.129079	–0.601587
H	5.098011	–2.043481	1.584002
C	0.010333	–2.262166	–0.264086
H	–0.360600	–1.138231	–1.062692
H	–0.034082	–3.047677	–1.029094
C	–1.076751	–2.463065	0.782123
H	–1.059583	–3.483571	1.221862
H	–0.937751	–1.757818	1.605412
N	–2.434272	–2.199242	0.249635
C	–2.811318	–3.253293	–0.688982
H	–3.815031	–3.068203	–1.085904

H	–2.114838	–3.287888	–1.525830
H	–2.816325	–4.239225	–0.189758
C	–3.401286	–2.171843	1.343903
H	–4.409557	–2.017963	0.945267
H	–3.393985	–3.114957	1.918878
H	–3.169412	–1.344709	2.018887
O	1.170963	2.837884	–0.719434
C	–1.418399	–1.222837	–3.134002
H	–2.461319	–1.313529	–2.796262
H	–1.444734	–0.895651	–4.191067
H	–0.977771	–2.229455	–3.125390
C	0.803626	–0.206222	–2.812078
H	0.814449	–0.035602	–3.905481
H	1.403158	0.610769	–2.380780
H	1.339373	–1.148682	–2.624242
C	0.126286	3.736784	–0.396727
H	–0.392876	4.070651	–1.306399
H	–0.570945	3.188797	0.239417
H	0.523528	4.613577	0.133944
C	1.989429	3.333387	–1.757758
H	2.43948	4.294587	–1.471256
H	2.773592	2.5967	–1.934597
H	1.402007	3.468396	–2.677143
O	–0.934031	0.802306	1.13474
C	–0.869523	1.316975	2.42783
C	–1.168911	0.247306	3.495663
H	–1.088465	0.660193	4.508986
H	–2.186592	–0.136955	3.370398
H	–0.474186	–0.595454	3.423951
C	0.540669	1.877297	2.704626
H	0.8066	2.633477	1.955248
H	0.619528	2.338559	3.696742
H	1.282015	1.067499	2.65527
C	–1.900844	2.443051	2.637245
H	–1.865939	2.838984	3.659682
H	–1.726688	3.279673	1.950461
H	–2.909513	2.05249	2.45637
C	–0.624628	–0.252958	–2.266706
Li	–1.907332	–0.151283	–0.072312
O	–3.687173	0.624377	–0.387224
C	–3.809678	2.033112	–0.313952
H	–2.896083	2.403171	0.152062
H	–3.914103	2.462755	–1.319654
H	–4.682442	2.308714	0.292615
C	–4.700610	0.031639	–1.170571
H	–4.683961	0.435419	–2.192885
H	–4.498536	–1.039545	–1.205425
H	–5.688273	0.21152	–0.724857
C	3.838097	1.481909	0.486408
H	3.073737	1.78783	1.204522
H	4.609216	0.89398	1.001816
H	4.294428	2.380287	0.043447
O	3.207242	0.703106	–0.508253
C	4.120669	0.245844	–1.483549

H	4.931043	–0.323381	–1.011696
H	3.573504	–0.405908	–2.166257
H	4.548321	1.096091	–2.037679
C	–1.245737	1.13706	–2.425685
H	–1.055762	1.546214	–3.439506
H	–2.334814	1.108537	–2.307020
H	–0.857930	1.868079	–1.706446
Na	0.904737	0.543396	–0.106677

Table S43 Coordinates of QM39.

Element	X	Y	Z
C	–0.006359	–1.587796	–2.299082
C	–1.159115	–1.570844	–1.525947
C	–1.279717	–2.312944	–0.291072
C	–0.081563	–3.045672	0.066683
C	1.052302	–3.049645	–0.722508
C	1.132082	–2.308035	–1.919978
H	0.006916	–1.015001	–3.225189
H	–2.010596	–0.995634	–1.885498
H	–0.110959	–3.676667	0.953946
H	1.89615	–3.664584	–0.413711
H	2.01726	–2.334418	–2.547677
C	–3.657665	–1.582119	0.069571
H	–3.850029	–1.716819	–1.005882
H	–4.549626	–1.959992	0.598859
N	–3.581848	–0.094241	0.27681
C	–4.580392	0.598278	–0.529510
H	–5.605954	0.287685	–0.262320
H	–4.494638	1.680167	–0.382503
H	–4.416885	0.378328	–1.589024
C	–3.789947	0.217845	1.68854
H	–3.615314	1.284908	1.868259
H	–4.818494	–0.028816	2.008083
H	–3.089969	–0.368715	2.291758
C	–2.425909	–2.301753	0.498578
H	–2.462269	–2.919827	1.391961
C	–0.462765	2.31369	–1.481971
O	–0.314125	1.394932	–0.451788
C	–1.538892	1.863357	–2.486752
H	–1.721889	2.619103	–3.261129
H	–1.238403	0.933049	–2.980516
H	–2.482050	1.681531	–1.956531
C	0.866102	2.477549	–2.241008
H	0.786829	3.173877	–3.085273
H	1.646432	2.851172	–1.565414
H	1.183055	1.499792	–2.628637
C	–0.892410	3.676021	–0.905701
H	–1.028059	4.438828	–1.682720
H	–1.841422	3.556836	–0.367092
H	–0.144980	4.045565	–0.192740
O	3.511827	–0.747466	–0.226519
C	4.230743	–1.955092	–0.343979
H	5.309963	–1.768408	–0.249528

H	3.903708	–2.610459	0.465162
H	4.031801	–2.442232	–1.308418
C	3.892548	0.196439	–1.205431
H	3.659604	–0.171124	–2.215090
H	3.327398	1.112791	–1.013836
H	4.968159	0.413749	–1.138103
Li	–1.530306	0.154996	0.121568
O	–0.509557	0.045788	1.925805
C	–0.529392	1.326406	2.540374
H	–0.580068	2.055078	1.72915
H	–1.405587	1.407682	3.199044
H	0.384048	1.473692	3.131612
C	–0.411984	–0.989375	2.885735
H	–1.255662	–0.947647	3.588453
H	–0.432030	–1.936105	2.348566
H	0.527658	–0.885242	3.446938
O	2.348391	1.150535	1.492046
C	2.259255	2.550332	1.318909
H	3.159788	2.934895	0.816031
H	1.376034	2.726509	0.70237
H	2.154261	3.055755	2.290322
C	3.448586	0.773571	2.288192
H	3.37649	1.234496	3.284182
H	3.433721	–0.313266	2.38076
H	4.395203	1.077831	1.818325
Na	1.144448	–0.224113	0.000224

Table S414 Coordinates of QM40.

Element	X	Y	Z
C	2.751103	–2.710930	1.488453
C	1.40592	–2.778121	1.113744
C	1.033767	–2.782012	–0.234127
C	2.054093	–2.751821	–1.197793
C	3.39471	–2.691096	–0.829994
C	3.749785	–2.657993	0.520375
H	3.011833	–2.699576	2.543418
H	0.645124	–2.819812	1.886988
H	1.783118	–2.763815	–2.251850
H	4.1639	–2.665299	–1.597681
H	4.795022	–2.603574	0.812558
C	–0.406014	–2.746245	–0.704925
H	–0.515484	–1.888773	–1.386963
H	–0.601520	–3.646295	–1.302339
C	–1.454715	–2.581794	0.395268
H	–1.660823	–3.552472	0.889914
H	–1.091872	–1.878473	1.150357
N	–2.697265	–1.993592	–0.118212
C	–3.308774	–2.847775	–1.129431
H	–4.254663	–2.409178	–1.461025
H	–2.659893	–2.934980	–2.002855
H	–3.516786	–3.859406	–0.732562
C	–3.627806	–1.786138	0.988125
H	–4.572243	–1.389835	0.60372

H	–3.836514	–2.727833	1.528994
H	–3.203570	–1.052071	1.677017
O	1.396358	2.940258	–0.142387
C	–1.838031	–0.304233	–3.133686
H	–2.874971	–0.293200	–2.765543
H	–1.878203	0.033405	–4.196291
H	–1.524556	–1.358981	–3.164344
C	0.476767	0.448099	–2.951084
H	0.436418	0.661516	–4.043578
H	1.207326	1.169487	–2.544142
H	0.920523	–0.559646	–2.859772
C	0.24081	3.559882	0.391701
H	–0.250748	4.184939	–0.365976
H	–0.435409	2.75442	0.695701
H	0.502991	4.182275	1.26046
C	2.253651	3.847305	–0.794222
H	2.636676	4.602194	–0.091193
H	3.084955	3.267912	–1.201655
H	1.728026	4.354961	–1.614788
O	–1.002385	0.414184	1.36468
C	–0.760339	0.628175	2.710005
C	–1.060366	–0.623060	3.563005
H	–0.845967	–0.450851	4.625448
H	–2.113391	–0.910661	3.473749
H	–0.446883	–1.468599	3.230372
C	0.727276	0.986625	2.942984
H	1.011348	1.856579	2.335895
H	0.943263	1.221485	3.992623
H	1.365131	0.135162	2.660195
C	–1.628214	1.777013	3.260081
H	–1.471411	1.93656	4.334466
H	–1.400061	2.717306	2.743317
H	–2.687198	1.543289	3.096161
C	–0.901881	0.56445	–2.293641
Li	–1.852716	0.150608	–0.263657
O	–3.630300	1.050177	–0.251854
C	–3.600406	2.35224	0.297637
H	–2.681827	2.422091	0.88049
H	–3.601262	3.109514	–0.498743
H	–4.469133	2.510212	0.95244
C	–4.731256	0.851458	–1.108485
H	–4.674418	1.519508	–1.979423
H	–4.696811	–0.184223	–1.450983
H	–5.673253	1.031756	–0.570798
K	1.142348	0.288859	–0.115860
C	4.25091	1.522627	0.753043
H	3.402525	1.846487	1.361242
H	4.867424	0.812389	1.322003
H	4.860972	2.400198	0.493583
O	3.727925	0.914555	–0.407376
C	4.730079	0.517949	–1.314378
H	5.408461	–0.215594	–0.854830
H	4.230315	0.062988	–2.172003
H	5.315677	1.386341	–1.649504

C	–1.337424	2.013177	–2.517549
H	–2.357900	2.207133	–2.157224
H	–0.671473	2.727094	–2.011259
H	–1.333048	2.29088	–3.599139

Table S425 Coordinates of QM41.

Element	X	Y	Z
C	2.607803	–2.128413	2.006716
C	1.263242	–2.113635	1.632948
C	0.834836	–2.558959	0.359286
C	1.853973	–3.053093	–0.493353
C	3.190163	–3.074659	–0.116589
C	3.589205	–2.603169	1.139205
H	2.883299	–1.774124	2.998162
H	0.536948	–1.739775	2.347758
H	1.571361	–3.427419	–1.475053
H	3.930462	–3.470931	–0.808632
H	4.633417	–2.628386	1.439057
C	–0.541928	–2.420354	–0.139634
H	–0.677661	–1.328157	–1.007516
H	–0.750187	–3.228004	–0.853541
C	–1.617494	–2.317320	0.929427
H	–1.781476	–3.274524	1.469897
H	–1.320691	–1.573761	1.674197
N	–2.912618	–1.840501	0.394875
C	–3.533415	–2.878823	–0.421792
H	–4.469588	–2.511194	–0.854848
H	–2.873271	–3.169955	–1.237696
H	–3.760173	–3.773870	0.185552
C	–3.809664	–1.503173	1.49699
H	–4.784367	–1.197765	1.1024
H	–3.962538	–2.361222	2.175944
H	–3.391372	–0.669509	2.065665
O	1.785136	2.823138	–0.795336
C	–2.112223	–1.026714	–2.918365
H	–3.021910	–0.687820	–2.404279
H	–2.212318	–0.731667	–3.981814
H	–2.112543	–2.124183	–2.896582
C	0.334212	–1.040263	–3.082609
H	0.256716	–0.805898	–4.162378
H	1.315467	–0.656709	–2.758646
H	0.372063	–2.134208	–2.995105
C	0.629993	3.626146	–0.643771
H	0.300037	4.017272	–1.616918
H	–0.150510	2.984922	–0.221572
H	0.829393	4.466119	0.037896
C	2.860492	3.520111	–1.381664
H	3.179966	4.355057	–0.741321
H	3.67858	2.80706	–1.498068
H	2.576031	3.915241	–2.367055
O	–1.007161	0.953861	1.052924
C	–0.771469	1.527258	2.294266
C	–1.257408	0.630158	3.448568

H	–1.023541	1.066218	4.428208
H	–2.342943	0.495895	3.392912
H	–0.785407	–0.357288	3.397169
C	0.741233	1.768761	2.502516
H	1.144793	2.40227	1.701115
H	0.954501	2.260736	3.459398
H	1.279738	0.809635	2.50052
C	–1.498870	2.879295	2.436294
H	–1.322059	3.339317	3.416692
H	–1.164030	3.585945	1.667383
H	–2.578869	2.729875	2.319585
C	–0.841449	–0.460037	–2.296357
Li	–2.039581	0.047128	–0.113150
O	–3.668605	1.110093	–0.522151
C	–3.531672	2.51752	–0.460189
H	–2.548403	2.713997	–0.031420
H	–3.606153	2.954639	–1.465885
H	–4.311208	2.948334	0.182919
C	–4.881476	0.713714	–1.123069
H	–4.937845	1.076262	–2.158895
H	–4.908194	–0.376920	–1.122231
H	–5.736585	1.103684	–0.553786
K	1.186826	0.267087	–0.237034
C	4.364678	1.094771	0.661393
H	3.546303	1.642915	1.136846
H	4.74813	0.3333	1.354504
H	5.172736	1.799592	0.414201
O	3.850372	0.491381	–0.505646
C	4.836828	–0.216783	–1.223473
H	5.323365	–0.964232	–0.582826
H	4.337709	–0.725054	–2.050458
H	5.596654	0.474095	–1.617420
C	–0.845492	1.056819	–2.507259
H	–0.989044	1.304964	–3.579990
H	–1.648330	1.56964	–1.961791
H	0.098397	1.540341	–2.218528

Table S436 Coordinates of QM42.

Element	X	Y	Z
C	–0.032846	–2.014035	–2.043453
C	–1.210318	–1.758848	–1.355481
C	–1.487668	–2.325250	–0.053080
C	–0.396392	–3.110665	0.486912
C	0.769586	–3.347887	–0.217174
C	0.991943	–2.799080	–1.496924
H	0.091482	–1.582999	–3.036457
H	–1.971833	–1.153690	–1.844207
H	–0.545129	–3.601002	1.448237
H	1.525356	–3.993228	0.22972
H	1.888057	–3.028627	–2.064706
C	–3.848853	–1.448416	–0.015160
H	–3.918404	–1.700445	–1.084147
H	–4.801894	–1.763458	0.445199

N	–3.774359	0.050171	0.038753
C	–4.652022	0.658308	–0.953755
H	–5.707019	0.373242	–0.793204
H	–4.574021	1.749066	–0.900566
H	–4.352571	0.338223	–1.956522
C	–4.140241	0.515729	1.373199
H	–3.967778	1.594896	1.45663
H	–5.202681	0.309651	1.59608
H	–3.525035	–0.005632	2.113422
C	–2.688048	–2.129622	0.625742
H	–2.840879	–2.633352	1.577122
C	–0.396967	2.212633	–1.623503
O	–0.544830	1.509539	–0.439015
C	–1.435749	1.764349	–2.665796
H	–1.359581	2.327267	–3.604997
H	–1.300700	0.699739	–2.889784
H	–2.444118	1.905825	–2.258402
C	1.010203	1.965016	–2.206310
H	1.192943	2.524871	–3.132267
H	1.776093	2.255058	–1.474038
H	1.131223	0.895211	–2.431156
C	–0.580729	3.720596	–1.372794
H	–0.491090	4.312952	–2.292501
H	–1.573870	3.895384	–0.941289
H	0.167015	4.084167	–0.657779
O	3.809705	–1.088445	0.008431
C	4.290576	–2.415268	0.047732
H	5.386	–2.432778	–0.041533
H	3.997151	–2.840041	1.009508
H	3.852397	–3.014920	–0.761088
C	4.096475	–0.452938	–1.221042
H	3.591584	–0.966907	–2.051788
H	3.728725	0.574564	–1.149578
H	5.179302	–0.437952	–1.410208
Li	–1.738204	0.284862	0.112371
O	–0.898627	0.347008	2.047667
C	–0.995243	1.673079	2.546979
H	–1.002524	2.327638	1.673341
H	–1.918930	1.789015	3.13133
H	–0.134261	1.899648	3.191638
C	–0.937213	–0.619153	3.08148
H	–1.896386	–0.581750	3.615702
H	–0.823354	–1.598155	2.615376
H	–0.122764	–0.436999	3.797024
K	1.19202	–0.305801	0.273043
O	2.865572	1.642589	1.079782
C	2.163281	2.868782	1.168271
H	2.626289	3.627122	0.519676
H	1.140869	2.675676	0.827857
H	2.162654	3.23875	2.204051
C	4.221337	1.764115	1.448628
H	4.312595	2.114529	2.486542
H	4.670951	0.774286	1.350953
H	4.739661	2.475396	0.788855

4.2 Calculated Aminometalation Reaction to Different Styrene Derivatives

4.2.1 Calculated Aminometalation Reaction of Dimethylamide to 1,1-Diphenylethene

In the following, the energies (Table S47) and the coordinates of the opitimized structures for the aminometalation to different metal dimethylamides to 1,1-diphenylethene (Scheme S9) are listed.

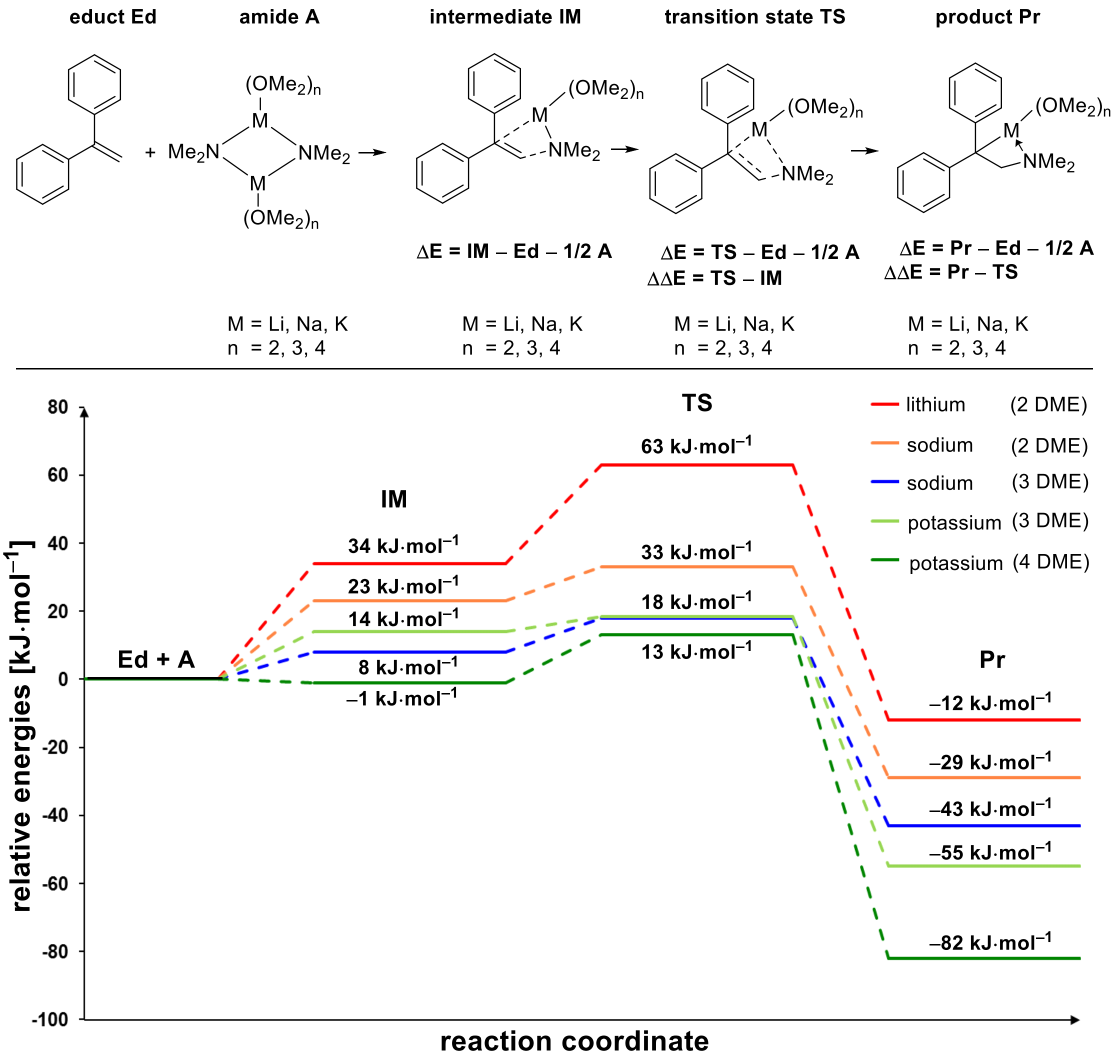

Scheme S9 Calculated reaction scheme for the aminometalation of different metal dimethylamides to 1,1-diphenylethene.

Table S47 Total (SCF) and zero-point-corrected (ZPE) energies of the optimized structures of the elimination reaction of a metal dimethylamide and 1,1-diphenylethene.

Optimized structure		SCF [Hartree]	ZPE [Hartree]
1,1-diphenylethene	QM43	–540.477056599	–540.260202
A Li + 2 DME	QM44	–904.007590038	–903.508184
A Na + 2 DME	QM45	–1213.41663972	–1212.924143
A Na + 3 DME	QM46	–1523.34423200	–1522.763223
A K + 3 DME	QM47	–2398.56867462	–2397.912793
A K + 4 DME	QM47b	-2708.49244230	-2707.668902
IM Li + 2 DME	QM48	–992.468097158	–992.001377
TS Li + 2 DME	QM49	–992.456764972	–991.990110X
Pr Li +2 DME	QM50	–992.491165328	–992.020597
IM Na + 2 DME	QM51	–1147.17848060	–1146.713553
TS Na + 2 DME	QM52	–1147.17479387	–1146.709614
Pr Na + 2 DME	QM53	–1147.20704560	–1146.738779
IM Na + 3 DME	QM54	–1302.14368438	–1301.595461
TS Na + 3 DME	QM55	–1302.14306649	–1301.594997
Pr Na + 3 DME	QM56	–1302.17564137	–1301.623462
IM K + 3 DME	QM57	–1739.75677836	–1739.211265
TS K + 3 DME	QM58	–1739.75603162	–1739.209664
Pr K + 3 DME	QM59	–1739.79883184	–1739.248034
IM K + 4 DME	QM60	–1894.72378865	–1894.094992
TS K + 4 DME	QM61	–1894.71821965	–1894.089787
Pr K + 4 DME	QM62	–1894.75970107	–1894.125793

Table S448 Coordinates of QM43

Element	X	Y	Z
C	3.596.661	0.119773	0.612128
C	2.395.367	0.82179	0.685438
C	1.275.452	0.401343	–0.042080
C	1.382.383	–0.752464	–0.830140
C	2.583.656	–1.451273	–0.908690
C	3.695.846	–1.017099	–0.187791
H	4.453.210	0.455154	1.190.119
H	2.314.305	1.693.617	1.329.416
H	0.51697	–1.094950	–1.392053
H	2.650.489	–2.337652	–1.533185
H	4.631.208	–1.566538	–0.242511
C	0.000006	2.510.928	0.000028
H	–0.924338	3.075.811	0.079389
H	0.924372	3.075.775	–0.079360
C	0.000007	1.169.617	0.000024
C	–1.275398	0.401311	0.042132
C	–1.382446	–0.752404	0.830238
C	–2.395270	0.821722	–0.685544
C	–2.583779	–1.451171	0.908753
H	–0.517136	–1.094924	1.392.293
C	–3.596586	0.119793	–0.612252
H	–2.314131	1.693.444	–1.329650

C	–3.695892	–1.017030	0.187774
H	–2.650622	–2.337515	1.533.293
H	–4.453086	0.455176	–1.190315
H	–4.631282	–1.566420	0.242464

Table S459 Coordinates of QM44.

Element	X	Y	Z
C	0.061238	1.596773	2.248992
C	–0.005051	–0.718833	2.568534
C	–0.043751	0.728788	–2.537000
C	0.057602	–1.591165	–2.257189
H	0.985101	1.722761	2.864449
H	–0.776070	1.771891	2.965946
H	0.042105	2.425402	1.524148
H	0.911958	–0.726814	3.201705
H	–0.844323	–0.644773	3.297946
H	–0.077904	–1.706505	2.092868
H	–0.140853	1.706726	–2.044110
H	0.876669	0.77089	–3.163827
H	–0.876503	0.648346	–3.272906
H	0.060299	–2.430722	–1.545014
H	0.978272	–1.691437	–2.882047
H	–0.783321	–1.770191	–2.968624
Li	1.131311	0.000615	–0.000558
Li	–1.175024	–0.033700	0.019989
N	–0.013741	0.328861	1.57899
N	–0.034029	–0.335569	–1.565887
O	2.439072	1.378839	–0.472127
O	–2.503782	1.325987	–0.376472
O	2.451902	–1.369851	0.452857
O	–2.427899	–1.465769	0.46967
C	–3.050929	–2.339741	–0.444531
H	–3.952599	–1.878207	–0.873352
H	–2.330978	–2.539237	–1.239840
H	–3.332862	–3.279156	0.050563
C	–3.286073	–1.030259	1.50233
H	–2.719841	–0.315278	2.104492
H	–4.176082	–0.538978	1.080485
H	–3.600669	–1.877389	2.126776
C	–3.253951	1.095407	–1.548522
H	–4.289684	0.822327	–1.295434
H	–3.261687	1.989217	–2.187057
H	–2.766037	0.273935	–2.078917
C	–3.023806	2.350268	0.439642
H	–2.393684	2.401571	1.329051
H	–3.002726	3.313591	–0.088246
H	–4.059866	2.124363	0.732184
C	3.232253	0.90825	–1.540874
H	4.199651	0.537418	–1.169064
H	2.678314	0.093166	–2.014483
H	3.409368	1.707707	–2.272826
C	3.062062	2.393094	0.282496
H	3.252717	3.276923	–0.341499
H	2.383655	2.653752	1.096392

H	4.015524	2.036637	0.69936
C	3.070047	–2.388219	–0.299959
H	4.02641	–2.037730	–0.715370
H	3.254173	–3.272844	0.324833
H	2.391468	–2.645422	–1.114809
C	3.244845	–0.904211	1.523984
H	2.69238	–0.088384	1.998059
H	3.417549	–1.705568	2.254873
H	4.214336	–0.536347	1.154448

Table S50 Coordinates of QM45.

Element	X	Y	Z
C	0.442628	–1.380869	–2.515954
C	0.555819	0.939796	–2.676373
C	–0.605779	–1.165576	2.65234
C	–0.575823	1.16326	2.619711
H	1.313819	–1.535724	–3.198839
H	–0.447655	–1.444326	–3.187954
H	0.406308	–2.258740	–1.850810
H	1.451381	0.928278	–3.345375
H	–0.312739	0.9783	–3.378044
H	0.575382	1.900366	–2.137697
H	–0.592591	–2.095795	2.062453
H	0.256467	–1.219018	3.359657
H	–1.508066	–1.216449	3.309375
H	–0.538664	2.076178	2.00422
H	0.287407	1.213636	3.327613
H	–1.476907	1.256155	3.27446
N	0.506359	–0.162585	–1.755880
N	–0.577372	–0.012447	1.793625
O	2.894144	–1.702620	0.368118
O	–2.744481	–1.894621	–0.281428
O	2.465927	1.925847	0.323021
O	–2.588252	1.874753	–0.459883
C	–3.217954	2.247583	0.749473
H	–4.297875	2.381656	0.595274
H	–3.031183	1.44718	1.469197
H	–2.788604	3.181354	1.137342
C	–2.663933	2.874767	–1.451488
H	–2.142920	2.500193	–2.334412
H	–3.710980	3.092973	–1.703863
H	–2.178315	3.79804	–1.106382
C	–3.399134	–2.029003	0.965431
H	–4.488622	–2.062190	0.825078
H	–3.070102	–2.943437	1.477376
H	–3.114923	–1.164832	1.570806
C	–2.942463	–3.005019	–1.127833
H	–2.396385	–2.813328	–2.053087
H	–2.556274	–3.920789	–0.659441
H	–4.010411	–3.138796	–1.350141
C	3.295288	–2.657768	1.324548
H	4.390919	–2.697307	1.399409
H	2.874363	–2.357354	2.285718
H	2.917682	–3.654345	1.057673

C	3.375834	–1.989721	–0.931524
H	3.05227	–2.990153	–1.249887
H	2.940235	–1.245528	–1.602586
H	4.473517	–1.939041	–0.952511
C	2.617583	3.001314	1.222392
H	3.669327	3.113004	1.521624
H	2.274103	3.939004	0.764006
H	2.008356	2.781029	2.100622
C	3.217068	2.088599	–0.863047
H	2.983244	1.238921	–1.508825
H	2.931765	3.016697	–1.377307
H	4.292523	2.114198	–0.637744
Na	–1.388822	–0.062907	–0.367589
Na	1.320432	–0.044896	0.406144

Table S51 Coordinates of QM46.

Element	X	Y	Z
C	0.306533	–1.641792	2.375265
C	–0.241355	–2.907443	0.505194
C	0.245625	2.907599	–0.513820
C	–0.309502	1.630654	–2.373906
H	1.219917	–2.225212	2.651175
H	–0.514026	–2.131597	2.953477
H	0.419631	–0.635127	2.802615
H	0.565468	–3.659066	0.694498
H	–1.136339	–3.358685	1.003839
H	–0.438381	–2.919368	–0.577986
H	0.443821	2.926836	0.568992
H	1.141085	3.353426	–1.016472
H	–0.559929	3.659364	–0.707696
H	–0.423506	0.621324	–2.794793
H	0.509333	2.116999	–2.957475
H	–1.223914	2.21215	–2.650758
N	0.058096	–1.580449	0.960178
N	–0.057061	1.57812	–0.959146
O	3.566624	–1.086969	0.494717
O	–2.394518	0.35695	2.230169
O	2.395032	–0.366857	–2.227083
O	–3.565632	1.092899	–0.486875
C	–3.347938	2.478494	–0.667107
H	–3.955109	3.062219	0.041485
H	–2.283863	2.650712	–0.496150
H	–3.606739	2.779899	–1.691999
C	–4.897877	0.718951	–0.737989
H	–4.965875	–0.360991	–0.592190
H	–5.586174	1.231966	–0.048511
H	–5.185911	0.968721	–1.770359
C	–3.219935	1.41794	2.643936
H	–4.124576	1.475366	2.020746
H	–3.514769	1.294327	3.696264
H	–2.646364	2.34107	2.533863
C	–3.057070	–0.888996	2.277982
H	–2.349917	–1.635634	1.9045
H	–3.348623	–1.136466	3.308493

H	–3.954977	–0.871127	1.639716
C	4.897144	–0.706626	0.745622
H	5.588031	–1.218589	0.057964
H	4.960323	0.373226	0.596757
H	5.185532	–0.952337	1.778855
C	3.354785	–2.473015	0.678177
H	3.612383	–2.770482	1.704504
H	2.291955	–2.650765	0.505046
H	3.966289	–3.055938	–0.027328
C	3.056155	0.879665	–2.279872
H	3.954552	0.86496	–1.642214
H	3.346762	1.123721	–3.311464
H	2.348514	1.626883	–1.908450
C	3.222057	–1.428590	–2.635693
H	2.649921	–2.352077	–2.521233
H	3.516877	–1.309606	–3.688557
H	4.126681	–1.481598	–2.012081
Na	–1.472112	0.014742	0.094755
Na	1.472475	–0.016795	–0.093216
O	–2.911150	–1.612648	–1.019737
O	2.906885	1.618549	1.011163
C	–3.360663	–2.932108	–0.812780
H	–3.400734	–3.102642	0.264195
H	–4.363706	–3.066671	–1.245556
H	–2.673149	–3.660742	–1.263452
C	–2.846109	–1.282438	–2.391519
H	–2.568596	–0.228186	–2.463050
H	–2.094407	–1.898650	–2.904744
H	–3.824162	–1.433325	–2.872975
C	2.832755	1.296695	2.384419
H	2.082094	1.920409	2.890048
H	2.548809	0.244433	2.460563
H	3.809189	1.444406	2.870136
C	3.366273	2.93352	0.798383
H	4.368711	3.06379	1.233958
H	3.411247	3.09772	–0.279407
H	2.682193	3.669404	1.242481

Table S52 Coordinates of QM47.

Element	X	Y	Z
C	0.008027	0.54923	2.11907
C	–0.252418	–1.738959	2.35336
C	0.180412	2.55554	–1.853352
C	–1.002944	0.952217	–3.039192
H	0.54492	0.693589	3.092046
H	–1.067639	0.737788	2.382462
H	0.302856	1.393778	1.470527
H	0.241407	–1.772896	3.358454
H	–1.344579	–1.640001	2.592408
H	–0.121452	–2.736918	1.905143
H	0.571254	2.880788	–0.872704
H	1.049279	2.568834	–2.565728
H	–0.474776	3.389962	–2.214895
H	–1.556348	–0.002195	–3.001739

H	–0.225042	0.83202	–3.835188
H	–1.712952	1.710496	–3.457598
N	0.242474	–0.713799	1.486966
N	–0.480949	1.292739	–1.748992
O	3.670346	–0.105002	1.392162
O	–3.943233	1.645583	–0.348597
O	2.756927	–2.212433	–1.036554
O	–1.568381	–2.411759	–1.703766
C	–1.979320	–2.877748	–2.967564
H	–1.641333	–3.911796	–3.129214
H	–3.070482	–2.846476	–2.993317
H	–1.578355	–2.241741	–3.769804
C	–0.157121	–2.368618	–1.586183
H	0.075794	–2.016079	–0.574513
H	0.275758	–3.369035	–1.738069
H	0.267663	–1.673372	–2.326084
C	–3.437526	2.742293	–1.094095
H	–3.982669	2.841464	–2.043385
H	–3.543420	3.675177	–0.521338
H	–2.380839	2.5242	–1.289299
C	–5.317656	1.758688	–0.074266
H	–5.614831	0.862582	0.475521
H	–5.525358	2.651555	0.535178
H	–5.898414	1.826854	–1.005639
C	5.020313	0.274797	1.293056
H	5.683696	–0.570848	1.529108
H	5.194904	0.606777	0.267982
H	5.247536	1.103952	1.981135
C	3.321324	–0.556423	2.6868
H	3.507137	0.231772	3.432636
H	2.255182	–0.800405	2.645553
H	3.91249	–1.443964	2.958564
C	3.478806	–2.984696	–1.964051
H	4.505235	–3.164224	–1.612229
H	2.988401	–3.955034	–2.132023
H	3.51038	–2.430414	–2.904447
C	2.643122	–2.846321	0.225836
H	2.014522	–2.207302	0.855966
H	2.172932	–3.834897	0.113001
H	3.635142	–2.968027	0.68448
K	–1.988294	–0.210096	–0.106021
K	1.74311	0.259285	–0.517774
O	–3.967552	–0.717899	1.737801
C	–4.317621	–1.980412	2.260354
H	–3.649093	–2.261804	3.085678
H	–5.356007	–1.975803	2.62244
H	-4.217957	-2.709194	1.453614
C	-4.014463	0.294392	2.720787
H	-3.320777	0.070643	3.543268
H	-3.716271	1.228218	2.238114
H	-5.033483	0.397648	3.123257
O	3.217698	2.549792	-0.065605
C	3.443504	3.634881	-0.938976
H	2.557395	4.28168	-0.999148

H	4.304546	4.230032	-0.601814
H	3.649415	3.222907	-1.928534
C	2.901836	2.981521	1.241617
H	3.730232	3.567344	1.667121
H	1.991592	3.598566	1.23852
H	2.734142	2.08809	1.848082

Table S53 Coordinates of QM47b.

Element	X	Y	Z
C	0.297711	0.807252	–2.313494
C	0.633935	–1.46282	–2.60607
C	–0.601816	2.230828	1.982291
C	0.181252	0.348754	3.080829
H	–0.112729	1.021398	–3.335561
H	1.398431	0.993496	–2.429277
H	–0.074985	1.610964	–1.652888
H	0.291297	–1.481091	–3.673718
H	1.745811	–1.325438	–2.678409
H	0.479492	–2.480932	–2.210721
H	–0.655325	2.791285	1.032423
H	–1.664249	2.092802	2.327368
H	–0.165246	2.93454	2.738548
H	0.771656	–0.580832	3.011867
H	–0.820643	0.056982	3.491692
H	0.646144	0.955481	3.902238
N	–0.016128	–0.485335	–1.789714
N	0.128255	1.015781	1.817812
O	–3.50751	0.218642	–2.165364
O	3.757815	1.824216	1.353675
O	–3.146068	–2.223335	–0.314235
O	1.320424	–2.507237	0.90826
C	1.81719	–3.255871	1.987908
H	1.587769	–4.325527	1.864046
H	2.901347	–3.115543	2.00755
H	1.38926	–2.910568	2.940831
C	–0.09016	–2.541642	0.813373
H	–0.345079	–1.953	–0.072763
H	–0.451721	–3.576542	0.704951
H	–0.544282	–2.089748	1.707846
C	3.076574	2.370666	2.473847
H	3.549794	2.038291	3.409206
H	3.102043	3.469755	2.433376
H	2.04146	2.012859	2.409894
C	5.087992	2.26791	1.264766
H	5.516888	1.842181	0.353804
H	5.13357	3.3665	1.206068
H	5.673596	1.94178	2.137915
C	–4.781589	0.798227	–2.293456
H	–5.449143	0.151444	–2.883624
H	–5.185873	0.930547	–1.287889
H	–4.720504	1.782071	–2.784539
C	–2.869227	0.013403	–3.412202
H	–2.769089	0.966417	–3.955144
H	–1.878874	–0.394876	–3.182272

H	–3.45227	–0.682363	–4.034848
C	–4.536436	–2.361793	–0.143326
H	–5.073464	–1.863637	–0.964091
H	–4.828121	–3.422859	–0.120099
H	–4.800394	–1.88409	0.802292
C	–2.710414	–2.761396	–1.548738
H	–1.71387	–2.359534	–1.748691
H	–2.68898	–3.86022	–1.502513
H	–3.383323	–2.447371	–2.358291
O	3.952068	1.035834	–1.565802
C	4.320763	0.326375	–2.726882
H	3.588288	0.484559	–3.53121
H	5.316578	0.639875	–3.075927
H	4.333854	–0.733764	–2.465173
C	3.823994	2.419946	–1.808305
H	3.100885	2.609793	–2.614198
H	3.46589	2.87562	–0.882438
H	4.794269	2.858934	–2.087703
O	–3.183721	2.677045	–0.30084
C	–3.510494	3.694818	0.618462
H	–2.607771	4.21551	0.967314
H	–4.195888	4.423925	0.161726
H	–4.001032	3.224572	1.47214
C	–2.54932	3.199113	–1.448803
H	–3.210457	3.908681	–1.969082
H	–1.615132	3.713574	–1.179526
H	–2.322038	2.359031	–2.109026
K	–1.905096	0.209311	0.092912
C	–3.821006	0.297805	3.32158
H	–3.990302	1.257169	2.829357
H	–4.761989	–0.056615	3.768746
H	–3.069175	0.432175	4.110692
C	–3.102198	–1.881351	2.890516
H	–4.004897	–2.290715	3.369975
H	–2.798575	–2.532	2.068011
H	–2.296679	–1.821595	3.636335
O	–3.367386	–0.608261	2.340342
C	3.923731	–3.033004	–0.78613
H	4.505273	–3.044855	–1.720884
H	4.314445	–3.817542	–0.119956
H	2.871099	–3.224072	–1.000113
C	5.308597	–1.390995	0.165598
H	5.938304	–1.307515	–0.733794
H	5.245496	–0.41644	0.65299
H	5.765044	–2.120887	0.851231
O	3.995489	–1.769682	–0.167114
K	1.980668	0.040675	0.118419

Table S53 Coordinates of QM48.

Element	X	Y	Z
C	2.982765	–2.352798	–0.265509
C	2.034018	–1.407880	–0.651161
C	0.676962	–1.759467	–0.733427

C	0.299215	–3.072295	–0.418103
C	1.251475	–4.014118	–0.036533
C	2.597093	–3.656814	0.043831
H	4.027131	–2.059825	–0.198862
H	2.332932	–0.372826	–0.831198
H	–0.746243	–3.362488	–0.486037
H	0.941661	–5.029210	0.195786
H	3.339421	–4.390473	0.34584
C	–0.000549	0.238719	–2.016000
H	–0.727346	0.993334	–2.308851
H	0.997863	0.33096	–2.432006
C	3.688224	1.644409	0.232967
H	4.35222	2.536159	0.243662
H	4.260219	0.868319	–0.335707
H	3.621996	1.290025	1.271032
C	2.555905	2.423204	–1.644006
H	3.037738	1.696254	–2.345386
H	3.192083	3.335329	–1.693995
H	1.5855	2.68921	–2.094661
C	–0.334601	–0.761169	–1.180161
N	2.390123	1.914052	–0.312557
Li	0.701818	1.413683	0.218698
O	0.312306	0.505924	1.878463
O	–0.648643	2.795672	0.055792
C	–0.641030	1.063308	2.762458
H	–1.470697	1.441304	2.163616
H	–0.197154	1.88676	3.337081
H	–1.012169	0.293974	3.453559
C	1.409889	–0.074608	2.566331
H	1.923716	0.683005	3.171852
H	2.093032	–0.471637	1.815473
H	1.056043	–0.888609	3.213096
C	–2.026786	2.706671	–0.243210
H	–2.593001	3.447637	0.337637
H	–2.364774	1.699315	0.014109
H	–2.202480	2.881002	–1.313995
C	–0.103483	4.079687	–0.222927
H	–0.274968	4.339853	–1.275860
H	0.968861	4.006851	–0.027110
H	–0.576075	4.832913	0.421091
C	–1.738023	–0.873170	–0.686453
C	–2.011739	–1.180087	0.654286
C	–2.815287	–0.622902	–1.546609
C	–3.321167	–1.189708	1.128709
H	–1.184322	–1.389045	1.326797
C	–4.126221	–0.636229	–1.074032
H	–2.619675	–0.426118	–2.597532
C	–4.383669	–0.910390	0.268503
H	–3.513419	–1.416842	2.174032
H	–4.947104	–0.441921	–1.758643
H	–5.404552	–0.921575	0.639222

Table S464 Coordinates of QM49.

Element	X	Y	Z
C	–1.864321	2.697639	–1.268195
C	–1.128315	1.522219	–1.406372
C	0.230769	1.450239	–1.029427
C	0.803253	2.628657	–0.505169
C	0.064746	3.796953	–0.359592
C	–1.280710	3.844296	–0.732807
H	–2.904221	2.713325	–1.586575
H	–1.625911	0.657571	–1.844448
H	1.851978	2.625668	–0.223562
H	0.546977	4.684442	0.04203
H	–1.855063	4.759229	–0.623267
C	0.56736	–0.832114	–1.935323
H	–0.270031	–0.733432	–2.621740
N	–0.654801	–2.418535	–0.687882
C	–1.217846	–3.220420	–1.735058
H	–0.481714	–3.449923	–2.539106
H	–1.590936	–4.207248	–1.386807
H	–2.062164	–2.703509	–2.215281
C	0.461602	–3.105175	–0.093955
H	0.161184	–4.006072	0.480174
H	1.201641	–3.461837	–0.844150
H	1.008964	–2.440142	0.590102
Li	–1.243833	–0.758098	–0.074611
C	1.01987	0.215155	–1.185452
O	–0.950758	–0.046469	1.693815
O	–3.230737	–0.749555	–0.023603
C	–3.761897	–2.019723	0.324064
H	–3.658015	–2.188713	1.405361
H	–4.823612	–2.069532	0.049438
H	–3.185470	–2.771242	–0.212847
C	–3.868532	0.293489	0.685148
H	–3.758787	0.145172	1.769204
H	–3.389491	1.2316	0.397898
H	–4.936454	0.330951	0.432564
C	–0.699467	–1.039756	2.674024
H	–0.726472	–2.006129	2.165126
H	0.289396	–0.887544	3.126375
H	–1.470923	–1.004243	3.455662
C	–1.020254	1.261708	2.228952
H	–1.843955	1.331758	2.953194
H	–0.079454	1.523371	2.732179
H	–1.189244	1.951961	1.399765
H	1.193633	–1.704537	–2.093702
C	2.294075	0.045341	–0.433293
C	3.439112	–0.447194	–1.074344
C	2.38406	0.318076	0.940346
C	4.619092	–0.677532	–0.370388
H	3.398212	–0.645201	–2.142635
C	3.56258	0.09696	1.647169
H	1.505557	0.696777	1.45644
C	4.688185	–0.406930	0.995198
H	5.491087	–1.060898	–0.893528

H	3.601313	0.311725	2.712267
H	5.608876	–0.582441	1.544025

Table S475 Coordinates of QM50.

Element	X	Y	Z
C	–2.287783	2.084541	–1.552985
C	–1.374324	1.026805	–1.570434
C	–0.009352	1.177178	–1.140242
C	0.328427	2.515792	–0.736141
C	–0.590631	3.543085	–0.717922
C	–1.932297	3.348378	–1.100023
H	–3.298088	1.904125	–1.917962
H	–1.719079	0.076908	–1.980225
H	1.361382	2.736847	–0.487821
H	–0.257374	4.535075	–0.419292
H	–2.645413	4.166074	–1.087659
C	0.527394	–1.148912	–1.881382
H	–0.086166	–0.913143	–2.759622
N	–0.290859	–2.087110	–1.030555
C	–1.067668	–3.000874	–1.861248
H	–0.421158	–3.607497	–2.518304
H	–1.650316	–3.682149	–1.231968
H	–1.763063	–2.430367	–2.484463
C	0.606397	–2.840809	–0.154135
H	0.02799	–3.480723	0.523002
H	1.289814	–3.483637	–0.736008
H	1.21503	–2.147793	0.434709
Li	–1.080162	–0.529397	0.049231
C	0.916238	0.093816	–1.138498
O	–0.853655	0.095029	1.87544
O	–2.945899	–1.162448	0.383466
C	–2.914441	–2.278171	1.250176
H	–2.665858	–1.963380	2.27368
H	–3.884948	–2.791630	1.253174
H	–2.145434	–2.958721	0.879677
C	–3.906525	–0.207555	0.797751
H	–3.709196	0.109188	1.831435
H	–3.822137	0.650964	0.129943
H	–4.915880	–0.634402	0.735782
C	–0.081219	–0.496136	2.903535
H	0.128048	–1.526176	2.606482
H	0.868049	0.037377	3.031723
H	–0.642118	–0.491641	3.848308
C	–1.191235	1.448664	2.1437
H	–1.818177	1.504971	3.044607
H	–0.284379	2.048657	2.288151
H	–1.734156	1.83648	1.277004
H	1.395551	–1.712810	–2.254582
C	2.213068	0.139562	–0.461512
C	3.347578	–0.550853	–0.949891
C	2.400819	0.819583	0.766454
C	4.56053	–0.562230	–0.268082
H	3.287412	–1.072069	–1.901318

C	3.614396	0.816789	1.443866
H	1.555241	1.345926	1.201645
C	4.714356	0.122634	0.937133
H	5.402312	–1.101893	–0.696046
H	3.699323	1.355674	2.385606
H	5.663342	0.119365	1.464548

Table S486 Coordinates of QM51.

Element	X	Y	Z
C	3.330439	–1.439938	–0.859214
C	2.185053	–0.673093	–1.069779
C	0.925254	–1.290008	–1.142317
C	0.843264	–2.681271	–0.979257
C	1.98947	–3.441394	–0.763321
C	3.23874	–2.821979	–0.703169
H	4.296254	–0.945340	–0.801364
H	2.254785	0.415101	–1.112051
H	–0.124455	–3.173217	–1.039815
H	1.907706	–4.518924	–0.650091
H	4.134124	–3.414095	–0.534938
C	–0.224835	0.667998	–2.110304
H	–1.104149	1.293084	–2.247867
H	0.706629	1.020326	–2.542230
C	3.192112	2.374062	0.562584
H	3.764555	3.327424	0.612546
H	3.89119	1.650158	0.063997
H	3.072972	2.029123	1.602549
C	2.143592	3.042162	–1.397149
H	2.71954	2.362507	–2.083800
H	2.728009	3.989684	–1.391641
H	1.192746	3.258403	–1.910290
C	–0.295649	–0.479328	–1.414371
N	1.92233	2.504407	–0.091279
O	0.690507	–0.177750	2.437279
O	–1.651634	2.403943	0.403069
C	–0.139949	–0.723608	3.440039
H	–1.099549	–0.203281	3.39433
H	0.309612	–0.580376	4.430859
H	–0.300834	–1.798750	3.27334
C	1.936578	–0.853806	2.331374
H	2.411602	–0.927613	3.318064
H	2.569243	–0.268345	1.662543
H	1.792709	–1.858215	1.907915
C	–3.023027	2.105471	0.262107
H	–3.636387	2.86674	0.763824
H	–3.199928	1.127146	0.715472
H	–3.301899	2.06188	–0.800226
C	–1.287370	3.636762	–0.204615
H	–1.542588	3.619576	–1.273681
H	–0.203868	3.747359	–0.082547
H	–1.817875	4.467593	0.279287
C	–1.605254	–0.928192	–0.860215
C	–1.695670	–1.432885	0.444803

C	–2.781694	–0.816151	–1.612281
C	–2.927427	–1.780464	0.9945
H	–0.784721	–1.548382	1.026885
C	–4.013994	–1.170608	–1.067605
H	–2.722259	–0.461896	–2.638224
C	–4.093492	–1.648104	0.240125
H	–2.976769	–2.161981	2.011237
H	–4.914172	–1.084023	–1.669768
H	–5.053854	–1.928012	0.663313
Na	0.28068	1.311108	0.765176

Table S497 Coordinates of QM52.

Element	X	Y	Z
C	–2.574343	3.037748	–0.994857
C	–1.642354	2.138918	–1.503591
C	–0.555984	1.687054	–0.729044
C	–0.463929	2.180505	0.587792
C	–1.392383	3.087833	1.096899
C	–2.454243	3.526153	0.307929
H	–3.391220	3.374822	–1.627537
H	–1.731869	1.801065	–2.532373
H	0.366911	1.863049	1.214635
H	–1.281874	3.453205	2.114565
H	–3.173693	4.239851	0.698199
C	–0.008812	–0.303010	–2.093485
H	–1.026084	–0.304086	–2.470702
N	–0.618343	–2.364307	–1.109320
C	–1.856751	–2.526864	–1.824302
H	–1.744706	–2.398844	–2.926152
H	–2.308228	–3.531484	–1.687127
H	–2.599462	–1.785370	–1.491791
C	0.326776	–3.312271	–1.628960
H	–0.052757	–4.354051	–1.600846
H	0.595152	–3.126213	–2.696327
H	1.265317	–3.302261	–1.057338
C	0.420089	0.706229	–1.269060
O	1.011668	–1.155594	2.057157
O	–2.802835	–0.960041	1.056107
C	–3.358639	–2.259956	1.128531
H	–3.842283	–2.413720	2.102412
H	–4.095000	–2.409600	0.328303
H	–2.539490	–2.970247	0.990774
C	–3.782580	0.056998	1.009108
H	–4.417301	0.023959	1.905201
H	–3.260672	1.014764	0.962201
H	–4.410437	–0.058793	0.114858
C	1.562101	–2.442302	1.827033
H	0.836166	–3.007091	1.234472
H	2.503584	–2.362157	1.268366
H	1.745947	–2.951245	2.78265
C	1.935611	–0.281169	2.678866
H	2.307946	–0.724004	3.612601
H	2.777523	–0.066602	2.007528

H	1.406346	0.644835	2.911402
H	0.695871	–0.919518	–2.643815
C	1.830901	0.741784	–0.826277
C	2.467188	1.934511	–0.432679
C	2.608221	–0.431775	–0.827838
C	3.809929	1.952856	–0.068509
H	1.905127	2.864647	–0.438263
C	3.956737	–0.410336	–0.478904
H	2.140502	–1.373732	–1.101865
C	4.568385	0.780585	–0.090427
H	4.272182	2.893417	0.219864
H	4.529817	–1.334367	–0.501446
H	5.618351	0.797748	0.186914
Na	–0.619041	–0.843972	0.524582

Table S508 Coordinates of QM53.

Element	X	Y	Z
C	2.122691	–1.507751	–2.256419
C	1.17507	–0.531062	–1.948764
C	–0.141890	–0.851259	–1.472496
C	–0.399058	–2.259357	–1.375353
C	0.550622	–3.214265	–1.683304
C	1.843747	–2.863558	–2.108699
H	3.097034	–1.190268	–2.625240
H	1.453534	0.511527	–2.106465
H	–1.397004	–2.586487	–1.100557
H	0.276727	–4.265143	–1.610252
H	2.582	–3.621444	–2.350293
C	–0.892651	1.524233	–1.740197
H	–0.322435	1.454159	–2.673926
N	–0.118727	2.457587	–0.849792
C	0.43866	3.561474	–1.620604
H	–0.347072	4.142863	–2.135605
H	0.989981	4.238381	–0.959225
H	1.131606	3.174037	–2.374041
C	–0.993495	2.978411	0.199656
H	–0.421839	3.618633	0.882107
H	–1.823134	3.575116	–0.221182
H	–1.438682	2.152546	0.764562
C	–1.110722	0.156747	–1.158923
O	1.059706	–0.828711	1.997015
O	3.123646	1.177028	0.754304
C	3.422251	2.052199	1.821984
H	3.270613	1.551559	2.788627
H	4.460708	2.402177	1.756506
H	2.750485	2.909976	1.743459
C	3.95856	0.032119	0.756314
H	3.832949	–0.530384	1.691618
H	3.660156	–0.589928	–0.091091
H	5.009835	0.32821	0.643677
C	0.388298	–0.824836	3.242104
H	0.157919	0.213084	3.491219
H	–0.548603	–1.392827	3.179588

H	1.028457	–1.256589	4.023325
C	1.399821	–2.141489	1.571948
H	2.125245	–2.584731	2.268315
H	0.50512	–2.774282	1.51964
H	1.83236	–2.072604	0.569964
H	–1.838200	2.028222	–2.001846
C	–2.300658	–0.105634	–0.354143
C	–3.511420	0.613105	–0.514827
C	–2.300376	–1.048984	0.707089
C	–4.616216	0.403309	0.304253
H	–3.599500	1.343877	–1.313415
C	–3.406382	–1.262749	1.519835
H	–1.389901	–1.610765	0.903204
C	–4.585722	–0.539108	1.332075
H	–5.521744	0.978505	0.123924
H	–3.343099	–1.999735	2.31859
H	–5.451541	–0.706273	1.965356
Na	0.945047	0.685425	0.321661

Table S519 Coordinates of QM54.

Element	X	Y	Z
C	–0.026792	3.872516	–0.602719
C	0.107852	2.552821	–1.030349
C	1.28056	1.831569	–0.749308
C	2.297773	2.459648	–0.015571
C	2.156056	3.776936	0.415087
C	0.99351	4.489496	0.1214
H	–0.942997	4.411508	–0.827488
H	–0.720996	2.052822	–1.531464
H	3.214411	1.919101	0.205917
H	2.959666	4.249928	0.972942
H	0.883964	5.517505	0.455683
C	0.769015	0.007085	–2.335324
H	0.858667	–1.025299	–2.663653
H	0.109356	0.651729	–2.907649
C	–3.173647	1.77656	–0.894948
H	–4.280212	1.780956	–1.037852
H	–2.825891	2.720041	–1.388444
H	–2.990521	1.91634	0.183812
C	–2.695962	0.579451	–2.813994
H	–2.222354	1.456953	–3.330302
H	–3.761290	0.606621	–3.139423
H	–2.248767	–0.318730	–3.267354
C	1.444318	0.439892	–1.256899
N	–2.526156	0.596336	–1.392740
O	–0.491113	0.512451	2.070212
O	–0.574836	–2.464983	–0.696824
C	–0.328276	–0.394126	3.142587
H	–0.401613	–1.403557	2.734635
H	–1.119261	–0.252631	3.891822
H	0.651391	–0.249931	3.621598
C	–0.353401	1.862505	2.476088
H	–1.084894	2.099794	3.261371

H	–0.531203	2.490078	1.601802
H	0.660865	2.047601	2.856974
C	0.472632	–3.409569	–0.666124
H	0.071867	–4.422565	–0.516891
H	1.136706	–3.145875	0.160303
H	1.045015	–3.385671	–1.604361
C	–1.462935	–2.666237	–1.783825
H	–0.921290	–2.609214	–2.738528
H	–2.212990	–1.869138	–1.740051
H	–1.946058	–3.651094	–1.705916
C	2.355275	–0.499334	–0.540127
C	2.340439	–0.592905	0.859009
C	3.213995	–1.345437	–1.253267
C	3.130001	–1.529971	1.520973
H	1.690425	0.069793	1.423913
C	4.009291	–2.279761	–0.592559
H	3.262903	–1.258056	–2.335785
C	3.965896	–2.381129	0.79724
H	3.093936	–1.594944	2.605436
H	4.671092	–2.923508	–1.165330
H	4.586446	–3.108150	1.313135
Na	–1.118031	–0.366956	0.040086
O	–2.685009	–1.437852	1.488717
C	–3.315811	–2.581152	0.950437
H	–3.826973	–2.328937	0.010932
H	–2.538424	–3.323575	0.761115
H	–4.047117	–2.987016	1.663752
C	–3.630551	–0.418405	1.763953
H	–4.372684	–0.775029	2.492316
H	–3.081187	0.426701	2.186614
H	–4.126320	–0.102029	0.837247

Table S60 Coordinates of QM55.

Element	X	Y	Z
C	–2.592988	–0.271852	–2.759501
H	–2.135480	–0.036356	–3.751312
H	–2.871933	0.688917	–2.299517
H	–3.546702	–0.791986	–3.002917
C	–4.180683	–0.468741	0.203122
H	–3.750063	–1.094187	–0.582791
H	–4.728065	0.369862	–0.249055
H	–4.868691	–1.045599	0.838392
O	–3.090044	0.010347	0.973383
C	–3.506290	0.866119	2.013293
H	–3.991887	1.764595	1.60667
H	–2.616267	1.153201	2.574746
H	–4.212909	0.351833	2.680338
C	0.488453	–0.129776	–2.401640
H	–0.120658	0.472275	–3.068009
H	0.672029	–1.149947	–2.718823
C	1.231719	0.440441	–1.409300
N	–1.735415	–1.033168	–1.899949
C	–1.506270	–2.311923	–2.505849

H	–1.089369	–2.246544	–3.539835
H	–2.431090	–2.923463	–2.605930
H	–0.788483	–2.899603	–1.913892
C	0.966373	1.848726	–1.033927
C	–0.311605	2.406475	–1.234060
C	1.965202	2.693198	–0.514673
C	–0.572799	3.745224	–0.946258
H	–1.109618	1.779597	–1.626565
C	1.701763	4.026768	–0.218952
H	2.967794	2.300614	–0.366810
C	0.430135	4.564655	–0.431187
H	–1.568559	4.145262	–1.119656
H	2.499015	4.656167	0.168063
H	0.228227	5.60738	–0.203989
C	0.09107	–0.122111	3.143243
H	1.151273	0.010359	3.402469
H	–0.083932	–1.153084	2.831094
H	–0.523031	0.116048	4.023721
C	–1.798366	–3.204683	0.908078
H	–2.603163	–2.499676	1.125322
H	–1.789716	–4.001227	1.665779
H	–1.956266	–3.642057	–0.085692
O	–0.261730	0.713353	2.05739
O	–0.583772	–2.478303	0.949254
C	–0.027639	2.080325	2.338562
H	–0.341383	2.659632	1.468951
H	1.040662	2.260666	2.525383
H	–0.600041	2.391469	3.224777
C	2.249729	–0.357335	–0.677206
C	2.948927	–1.396946	–1.318665
C	2.5466	–0.130443	0.678936
C	3.87142	–2.183337	–0.635735
H	2.778325	–1.576505	–2.376302
C	3.478074	–0.909373	1.362512
H	2.032588	0.669037	1.203191
C	4.144169	–1.947550	0.71272
H	4.394072	–2.975609	–1.165037
H	3.682539	–0.704206	2.41062
H	4.872505	–2.553862	1.243176
C	0.546982	–3.299736	0.726858
H	0.493635	–3.773034	–0.262576
H	1.433145	–2.664572	0.776838
H	0.607327	–4.081166	1.497727
Na	–0.952492	–0.358544	0.102604

Table S61 Coordinates of QM56.

Element	X	Y	Z
C	0.194617	–2.478703	1.740426
H	0.959551	–2.977526	2.364633
H	0.614254	–2.341981	0.737255
H	–0.673603	–3.140500	1.661832
C	–3.116164	–2.510771	0.503431
H	–3.215918	–1.642935	1.160019
H	–2.955110	–3.412422	1.112333
H	–4.037924	–2.635419	–0.082669

O	–2.015279	–2.272705	–0.345041
C	–1.728897	–3.356094	–1.199737
H	–1.408203	–4.235125	–0.622703
H	–0.924223	–3.036101	–1.864748
H	–2.612629	–3.624127	–1.796763
C	0.988953	–0.287943	2.426665
H	1.854402	–0.890558	2.743272
H	0.774576	0.370381	3.277211
C	1.300409	0.514642	1.194542
N	–0.205479	–1.192506	2.298641
C	–0.836872	–1.408458	3.594748
H	–0.146892	–1.869395	4.324738
H	–1.703649	–2.069276	3.480714
H	–1.182735	–0.453560	4.001741
C	2.501144	0.202109	0.437432
C	3.120342	–1.078384	0.4903
C	3.193245	1.152425	–0.366292
C	4.250288	–1.400158	–0.252858
H	2.700566	–1.853703	1.122755
C	4.318572	0.825551	–1.112733
H	2.866648	2.186192	–0.361925
C	4.854296	–0.464649	–1.093706
H	4.663078	–2.403783	–0.172135
H	4.800107	1.603639	–1.701694
H	5.731453	–0.719208	–1.680462
C	–0.225489	0.446263	–3.042457
H	0.68166	1.040726	–3.215646
H	–1.018919	1.104987	–2.681588
H	–0.526985	–0.031680	–3.986218
C	–3.398903	–0.170694	–2.109206
H	–2.807415	–1.059483	–2.336268
H	–3.378657	0.514198	–2.969032
H	–4.442319	–0.455010	–1.909792
O	0.003039	–0.530055	–2.046145
O	–2.825269	0.437987	–0.973824
C	1.18599	–1.273517	–2.299139
H	1.297583	–2.004786	–1.496253
H	2.068617	–0.620862	–2.292186
H	1.108036	–1.790343	–3.267106
C	0.470834	1.666911	0.952631
C	–0.473297	2.15988	1.913621
C	0.411588	2.358973	–0.303158
C	–1.212950	3.32157	1.711431
H	–0.587117	1.661234	2.87021
C	–0.351209	3.499865	–0.503639
H	0.999595	1.981892	–1.132332
C	–1.150643	4.033225	0.513316
H	–1.858526	3.670749	2.515345
H	–0.318855	3.983504	–1.479036
H	–1.723369	4.943314	0.364682
C	–3.420103	1.686747	–0.666927
H	–4.497686	1.562556	–0.489780
H	–2.930286	2.071125	0.228748
H	–3.264541	2.399462	–1.488251

Na	–0.910968	–0.290290	0.065323

Table S62 Coordinates of QM57.

Element	X	Y	Z
C	–2.546800	3.181703	–1.598286
H	–2.217440	3.575898	–2.590554
H	–2.145057	3.86564	–0.834376
H	–3.658106	3.322722	–1.594644
C	–4.583299	0.681186	0.52503
H	–3.948397	1.343926	–0.077514
H	–5.162288	1.248857	1.266751
H	–5.277730	0.126819	–0.123338
O	–3.702216	–0.224315	1.180132
C	–4.382673	–1.180642	1.953948
H	–4.950168	–0.701168	2.764263
H	–3.636278	–1.854903	2.381582
H	–5.080203	–1.764285	1.334158
C	0.548527	0.05265	–2.355509
H	0.063803	0.946283	–2.737882
H	0.208715	–0.901961	–2.751922
C	1.49154	0.10421	–1.398449
N	–2.134027	1.834568	–1.345610
C	–2.660084	1.000942	–2.381787
H	–2.333817	1.288508	–3.412896
H	–3.780616	0.985223	–2.438833
H	–2.343233	–0.050190	–2.247228
C	1.911307	1.402154	–0.793847
C	1.021418	2.489906	–0.761181
C	3.207963	1.571388	–0.285651
C	1.438313	3.717371	–0.247378
H	–0.010347	2.364489	–1.119933
C	3.617528	2.80152	0.223922
H	3.907183	0.739102	–0.307316
C	2.734435	3.881003	0.243141
H	0.739098	4.549235	–0.230851
H	4.630218	2.91808	0.600309
H	3.055445	4.841522	0.636941
C	0.544712	–0.552605	3.559002
H	1.552086	–0.992792	3.60415
H	–0.170958	–1.325455	3.267153
H	0.281252	–0.166447	4.553202
C	–2.822594	–2.544321	–0.960858
H	–3.456421	–1.692181	–0.708603
H	–3.299558	–3.477843	–0.628132
H	–2.678974	–2.581493	–2.049619
O	0.486967	0.470136	2.590761
O	–1.585959	–2.360156	–0.299200
C	1.418499	1.506336	2.842898
H	1.313964	2.243697	2.045247
H	2.44552	1.113042	2.834748
H	1.218465	1.975376	3.815839
C	2.116435	–1.157395	–0.905624
C	2.487597	–2.168617	–1.800321

C	2.30069	–1.381803	0.466966
C	2.999874	–3.380009	–1.338796
H	2.380877	–1.994391	–2.867864
C	2.813268	–2.591670	0.929921
H	2.018271	–0.602976	1.171755
C	3.161365	–3.598279	0.028763
H	3.284356	–4.150554	–2.050040
H	2.94055	–2.751539	1.997575
H	3.564477	–4.540081	0.389565
C	–0.659589	–3.376180	–0.622799
H	–0.429948	–3.366195	–1.697893
H	0.256838	–3.185536	–0.060641
H	–1.059301	–4.364178	–0.352050
K	–1.148685	0.228176	0.417158

Table 523 Coordinates of QM58.

Element	X	Y	Z
C	1.599583	–1.859243	–3.310851
H	1.224617	–1.427167	–4.270142
H	1.006902	–2.764460	–3.112035
H	2.637114	–2.196427	–3.531174
C	3.930202	–2.004903	–0.133669
H	3.280637	–1.832444	–0.999883
H	4.15893	–3.075133	–0.033093
H	4.872716	–1.447583	–0.241642
O	3.216373	–1.545947	1.004964
C	3.926013	–1.743241	2.203762
H	4.116342	–2.811833	2.376537
H	3.315774	–1.347382	3.01884
H	4.89093	–1.214132	2.182086
C	–0.733619	–0.116802	–2.566909
H	–0.815260	–1.064712	–3.087679
H	–0.212883	0.672235	–3.100355
C	–1.521821	0.146639	–1.482232
N	1.522466	–0.946384	–2.210851
C	2.280896	0.228031	–2.531941
H	1.977613	0.711276	–3.491316
H	3.375217	0.039242	–2.639124
H	2.168293	0.996384	–1.747314
C	–2.223219	–0.988418	–0.836178
C	–1.640935	–2.270555	–0.840543
C	–3.496708	–0.855948	–0.251133
C	–2.309817	–3.371034	–0.304702
H	–0.653971	–2.399016	–1.285154
C	–4.161468	–1.953162	0.285636
H	–3.979772	0.118194	–0.247568
C	–3.573389	–3.220158	0.263888
H	–1.837759	–4.349860	–0.332495
H	–5.151985	–1.822223	0.713926
H	–4.096210	–4.076698	0.679746
C	–0.338444	0.552528	3.423029
H	–1.208266	1.225073	3.416418
H	0.539583	1.099415	3.06834
H	–0.165386	0.203089	4.450684

C	3.708387	1.745586	0.591588
H	4.06366	0.767929	0.921619
H	4.191491	2.53911	1.179414
H	3.958307	1.881101	–0.470182
O	–0.542846	–0.537149	2.551147
O	2.309458	1.767697	0.783821
C	–1.705954	–1.276934	2.865149
H	–1.813646	–2.057695	2.109648
H	–2.596304	–0.631990	2.838047
H	–1.618267	–1.728353	3.863232
C	–1.611131	1.506484	–0.899497
C	–1.421244	2.656728	–1.690925
C	–1.882784	1.715252	0.467339
C	–1.498297	3.935607	–1.150068
H	–1.237593	2.543019	–2.755379
C	–1.978076	2.997467	1.006787
H	–2.025998	0.854623	1.115817
C	–1.785677	4.11983	0.204243
H	–1.354214	4.797243	–1.796847
H	–2.202201	3.119223	2.064028
H	–1.865299	5.11904	0.622079
C	1.72696	2.975739	0.333169
H	1.916756	3.124111	–0.739343
H	0.650271	2.906167	0.500762
H	2.130362	3.832065	0.892261
K	0.847454	–0.456343	0.2499

Table S534 Coordinates of QM59.

Element	X	Y	Z
C	0.335157	–2.022694	1.726158
H	1.041933	–2.710571	2.229641
H	0.72813	–1.818236	0.722008
H	–0.631554	–2.534676	1.632398
C	–3.443100	–2.800256	0.378332
H	–3.950713	–1.926180	0.793155
H	–3.044156	–3.420203	1.194287
H	–4.158901	–3.398797	–0.203333
O	–2.401834	–2.325067	–0.447495
C	–1.665079	–3.370667	–1.050552
H	–1.186169	–4.000701	–0.287354
H	–0.903472	–2.902655	–1.676899
H	–2.321555	–3.997739	–1.669821
C	1.44192	0.004283	2.462868
H	2.260969	–0.652339	2.803387
H	1.321971	0.751676	3.260255
C	1.781362	0.656258	1.15184
N	0.16808	–0.772655	2.447391
C	–0.294856	–1.038257	3.799276
H	0.435777	–1.626519	4.386436
H	–1.237648	–1.596463	3.771066
H	–0.470453	–0.091859	4.320539
C	2.967101	0.225415	0.433613
C	3.494395	–1.089429	0.541183
C	3.708368	1.09985	–0.406395

C	4.602417	–1.510067	–0.186477
H	3.010769	–1.810321	1.194719
C	4.809156	0.67468	–1.138865
H	3.426886	2.147739	–0.448046
C	5.263701	–0.644454	–1.059001
H	4.950518	–2.534733	–0.072700
H	5.335824	1.392205	–1.764758
H	6.124315	–0.975625	–1.632238
C	–0.180858	0.111003	–3.283223
H	0.554375	0.92396	–3.348408
H	–1.174023	0.543344	–3.131186
H	–0.172374	–0.459838	–4.222766
C	–4.557697	–0.044102	–1.433385
H	–4.018869	–0.942335	–1.742489
H	–4.548851	0.693671	–2.248072
H	–5.600391	–0.300193	–1.197275
O	0.095149	–0.731571	–2.185840
O	–3.897157	0.463208	–0.293055
C	1.414005	–1.254597	–2.241547
H	1.545905	–1.915864	–1.383172
H	2.160831	–0.452701	–2.175138
H	1.557739	–1.822249	–3.172129
C	0.936712	1.721946	0.721615
C	–0.065048	2.288398	1.584948
C	0.855727	2.190386	–0.635916
C	–1.052138	3.152619	1.129391
H	–0.081960	2.012569	2.634146
C	–0.148490	3.035921	–1.079392
H	1.56393	1.812875	–1.365901
C	–1.140542	3.527065	–0.216484
H	–1.772169	3.543942	1.847023
H	–0.163844	3.318978	–2.131214
H	–1.910971	4.205972	–0.568208
C	–4.488966	1.654755	0.187224
H	–5.532032	1.475255	0.48244
H	–3.911297	1.980759	1.054019
H	–4.456760	2.440531	–0.579110
K	–1.250369	0.073422	0.034885

Table S545 Coordinates of QM60.

Element	X	Y	Z
C	–2.385241	2.151444	2.18358
C	–1.967666	0.103474	3.240442
C	1.747028	–1.046246	2.836874
C	0.731007	3.435527	1.13746
C	0.741639	4.583761	0.343514
C	–3.408744	0.853213	–2.065984
C	0.639837	–2.818024	1.758983
C	1.082042	2.203687	0.590814
C	–4.426061	–0.941756	0.736139
C	1.103601	4.488545	–0.999942
C	4.832551	–0.322648	0.55547
C	–3.406329	–2.887553	–0.123211
C	3.833954	0.477654	0.005148

C	1.432009	2.09434	–0.762402
C	4.752795	–1.711323	0.447977
C	1.445268	3.253191	–1.548094
C	–2.04599	2.682622	–1.520383
C	2.749398	–0.09862	–0.672278
C	1.743355	0.761739	–1.354257
C	3.658157	–2.290811	–0.194863
C	2.654555	–1.496882	–0.747402
C	0.18021	–3.954924	–1.639901
C	1.103021	0.357737	–2.46348
C	–1.091759	–2.287999	–2.613831
N	–0.371915	–2.651358	–1.431513
O	–1.679313	0.934298	2.135738
O	0.67527	–1.419542	2.000595
O	–2.404603	1.354995	–1.207745
O	–3.240624	–1.533644	0.272441
K	–0.80262	–0.495144	–0.031556
C	–3.471233	1.976153	2.204785
C	–2.105661	2.731621	3.075739
C	–2.128725	2.717587	1.28645
C	–3.041261	–0.13166	3.279777
C	–1.675474	0.593367	4.180982
C	–1.39099	–0.813923	3.110948
C	2.709511	–1.240668	2.343906
C	1.707213	–1.594406	3.790129
C	1.652256	0.024065	3.038605
C	0.44997	3.496641	2.1856
C	0.477103	5.54659	0.771467
C	–3.082188	0.897443	–3.114549
C	–3.581541	–0.188166	–1.786745
C	–4.337907	1.431342	–1.953571
C	0.347512	–3.352044	2.67517
C	–0.080291	–2.997832	0.953098
C	1.62444	–3.173925	1.430475
C	1.075917	1.313053	1.216898
C	–4.781859	–1.430942	1.655532
C	–5.222159	–1.003096	–0.021873
C	–4.205331	0.110243	0.93989
C	1.125316	5.379333	–1.621513
C	5.676722	0.13944	1.059858
C	–4.162261	–2.959811	–0.918717
C	–3.728642	–3.497147	0.733366
C	–2.4278	–3.214493	–0.496388
C	3.908279	1.560216	0.079377
C	5.534679	–2.337379	0.869479
C	1.736913	3.178243	–2.592625
C	–1.257122	2.985971	–0.828107
C	–2.910265	3.355637	–1.415407
C	–1.662304	2.754781	–2.548731
C	3.577996	–3.372952	–0.267185
C	1.763603	–1.95093	–1.196324
C	0.754273	–4.289905	–0.760043
C	–0.585038	–4.746504	–1.837912
C	0.878862	–4.014583	–2.512307

C	0.339491	0.981501	–2.924867
C	1.297097	–0.608407	–2.919951
C	–1.955519	–2.961366	–2.854711
C	–1.515375	–1.26844	–2.534341
C	–0.472678	–2.287139	–3.54601

Table S556 Coordinates of QM61.

Element	X	Y	Z
C	–2.160966	1.250584	2.934025
C	–1.574839	–0.950308	3.495208
C	1.596766	–1.804072	2.453222
C	0.509297	3.399515	1.380291
C	0.178844	4.496901	0.584647
C	–4.175521	0.760366	–1.126536
C	0.671913	–3.132564	0.735905
C	1.028019	2.241039	0.80437
C	–3.7138	–2.158859	1.005432
C	0.374484	4.413448	–0.795285
C	4.819168	0.033353	0.934666
C	–2.708228	–3.085289	–0.911625
C	3.77159	0.756016	0.371093
C	1.240853	2.13752	–0.585459
C	4.990136	–1.31997	0.637144
C	0.898177	3.257673	–1.367515
C	–2.622024	2.480383	–0.764986
C	2.854549	0.149385	–0.508261
C	1.796581	0.910169	–1.204002
C	4.079359	–1.942305	–0.216485
C	3.018858	–1.224851	–0.763672
C	0.321853	–2.256387	–3.272687
C	1.312643	0.446313	–2.40039
C	–1.334604	–0.632873	–3.139708
N	–0.284774	–1.28606	–2.410422
O	–1.532849	0.060734	2.514436
O	0.65997	–1.887407	1.402942
O	–3.044766	1.183858	–0.397046
O	–2.703119	–2.035489	0.036924
K	–0.740278	–0.230618	–0.060236
H	–3.229478	1.079767	3.13085
H	–1.686874	1.64387	3.845403
H	–2.057314	1.979259	2.12744
H	–2.611042	–1.15312	3.805297
H	–0.992644	–0.659808	4.382041
H	–1.141898	–1.846065	3.045405
H	2.621551	–1.886895	2.068591
H	1.418745	–2.591867	3.20177
H	1.465367	–0.825886	2.923191
H	0.365905	3.443665	2.45736
H	–0.216393	5.404364	1.031752
H	–3.99344	0.830646	–2.20815
H	–4.356539	–0.283478	–0.864622
H	–5.053008	1.370862	–0.869447
H	0.206952	–3.909503	1.361955
H	0.103876	–3.007887	–0.192733

H	1.698386	–3.434133	0.48972
H	1.286796	1.397555	1.443621
H	–3.536201	–3.030199	1.653893
H	–4.702462	–2.270051	0.534216
H	–3.699304	–1.244821	1.604862
H	0.134366	5.263494	–1.428859
H	5.520016	0.536246	1.596328
H	–3.661737	–3.101392	–1.459936
H	–2.566015	–4.056847	–0.415261
H	–1.880573	–2.879353	–1.597777
H	3.67938	1.817149	0.588693
H	5.816863	–1.878668	1.066717
H	1.079924	3.223148	–2.438491
H	–1.743929	2.727976	–0.162544
H	–3.411423	3.222036	–0.574853
H	–2.343864	2.515471	–1.828836
H	4.188222	–2.999359	–0.4498
H	2.289571	–1.726709	–1.394108
H	1.104712	–2.822946	–2.747683
H	–0.402954	–3.003746	–3.664587
H	0.796151	–1.812641	–4.181578
H	0.578649	1.023913	–2.956962
H	1.866477	–0.288831	–2.974978
H	–2.160535	–1.317997	–3.43895
H	–1.79616	0.168399	–2.53707
H	–0.99197	–0.156156	–4.088853

Table S567 Coordinates of QM62.

Element	X	Y	Z
C	2.090895	–1.263199	3.393169
C	2.090092	1.084058	3.271941
C	–1.336926	0.744921	2.408264
C	0.005753	–3.258005	1.277398
C	1.080323	–3.745262	0.522161
C	4.226458	–1.33379	–0.105441
C	–1.662305	2.825125	1.400059
C	–0.923507	–2.378255	0.744876
C	1.68136	3.826061	0.517201
C	1.17864	–3.298142	–0.797611
C	–4.980933	–1.025675	0.811079
C	3.131098	2.776607	–0.997602
C	–3.774479	–1.402455	0.235012
C	–0.861637	–1.898384	–0.609408
C	–5.518543	0.246715	0.598923
C	0.265972	–2.403283	–1.34248
C	3.371511	–0.907507	–2.256606
C	–3.002345	–0.520536	–0.570098
C	–1.72196	–0.893257	–1.150906
C	–4.822498	1.112949	–0.244075
C	–3.606775	0.743118	–0.810535
C	–0.499718	1.868373	–1.962797
C	–1.359512	–0.332694	–2.499261
C	0.319052	0.730831	–3.881621
N	–0.168457	0.566831	–2.52235

O	2.085031	–0.129534	2.550374
O	–0.762462	1.748802	1.587571
O	3.340139	–0.562642	–0.888626
O	2.212246	2.602115	0.061166
K	0.97032	0.169392	0.123447
H	2.975682	–1.254099	4.045379
H	1.185796	–1.288981	4.017411
H	2.109066	–2.144839	2.749709
H	2.97925	1.148168	3.914975
H	1.187935	1.173182	3.89446
H	2.108501	1.893636	2.539679
H	–2.243569	0.334066	1.942669
H	–1.583358	1.15506	3.398895
H	–0.59488	–0.051972	2.522983
H	–0.114674	–3.573042	2.313343
H	1.791206	–4.454681	0.935233
H	5.265086	–1.188559	–0.436098
H	4.117079	–0.994802	0.927115
H	3.968106	–2.399677	–0.168361
H	–1.828811	3.355269	2.349291
H	–1.215264	3.511041	0.676152
H	–2.621745	2.462482	1.01074
H	–1.713873	–2.025244	1.396749
H	1.116607	4.329686	–0.281956
H	2.483717	4.495275	0.859609
H	1.010082	3.599593	1.348516
H	1.988593	–3.664786	–1.427325
H	–5.524763	–1.74941	1.41466
H	3.98296	3.39127	–0.674306
H	2.647865	3.265677	–1.856677
H	3.483608	1.783623	–1.282324
H	–3.434535	–2.424419	0.366915
H	–6.462298	0.539848	1.048736
H	0.431198	–2.072752	–2.363322
H	2.559635	–0.362248	–2.744768
H	3.207132	–1.983895	–2.392086
H	4.336738	–0.62956	–2.705414
H	–5.22733	2.099846	–0.461476
H	–3.104469	1.463171	–1.449938
H	–0.924239	1.74279	–0.960392
H	0.40383	2.487207	–1.888431
H	–1.24209	2.411213	–2.579825
H	–1.126815	–1.144239	–3.204917
H	–2.216314	0.203241	–2.943501
H	1.195623	1.390035	–3.891145
H	0.61203	–0.242267	–4.2915
H	–0.444904	1.168042	–4.552248

4.2.2 Calculated Aminometalation Reaction of Dimethylamide to Phenylsilylethene (13)

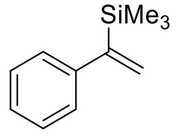

13

Figure S33 Lewis formula of tri-methyl(1-phenylvinyl)silane (**13**).

In the following, the energies (Table S68) and the coordinates of the opitimized structures for the aminometalation of different metal dimethylamides to **13** (Scheme S10) are listed.

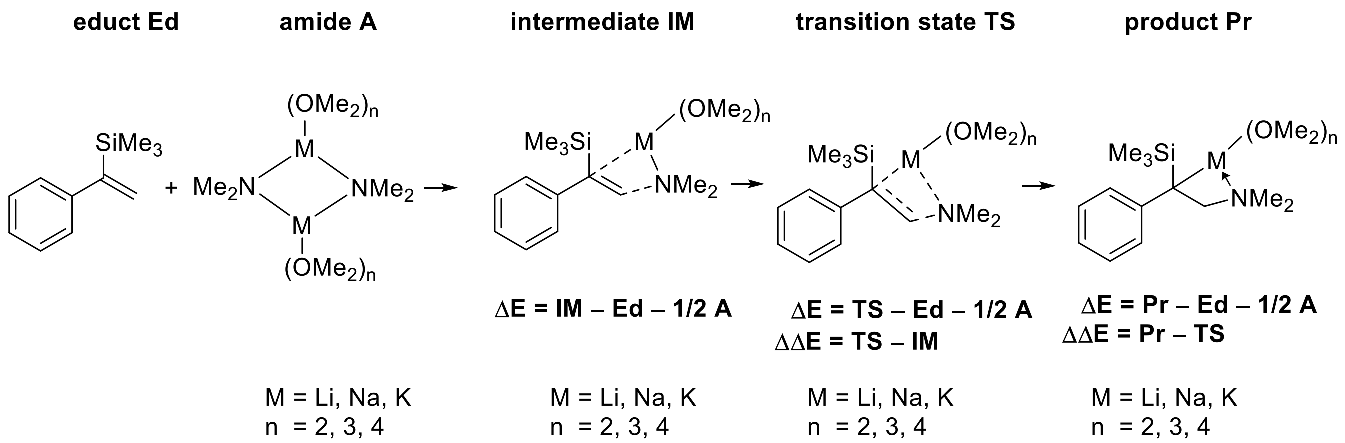

Scheme S10: Calculated reaction scheme for the aminometalation of different metal dimethylamides to **13**.

Table S68 Total (SCF) and zero-point-corrected (ZPE) energies of the optimized structures of the elimination reaction of a metal dimethylamide and **13**.

Optimized structure		SCF [Hartree]	ZPE [Hartree]
**13**	QM63	–718.082368426	–717.845233
A Li + 2 DME	QM44	–904.007590038	–903.508184
A Na + 2 DME	QM45	–1213.41663972	–1212.924143
A Na + 3 DME	QM46	–1523.34423200	–1522.763223
A K + 3 DME	QM47	–2398.56867462	–2397.912793
A K + 4 DME	QM47b	-2708.49244230	-2707.668902
IM Li + 2 DME	QM64	–1170.07006136	–1169.582438
TS Li + 2 DME	QM65	–1170.06590553	–1169.577693
Pr Li +2 DME	QM66	–1170.10072944	–1169.608803
IM Na + 2 DME	QM67	–1324.77845089	–1324.293957
TS Na + 2 DME	QM68	–1324.77948776	–1324.294644
Pr Na + 2 DME	QM69	–1324.81972988	–1324.329654
IM Na + 3 DME	QM70	–1479.75404604	–1479.185702
TS Na + 3 DME	QM71	–1479.74901679	–1479.179921
Pr Na + 3 DME	QM72	–1479.78455202	–1479.212125
IM K + 3 DME	QM73	–1917.36152441	–1916.796011
TS K + 3 DME	QM74	–1917.35323201	–1916.786445
Pr K + 3 DME	QM75	–1917.39820648	–1916.827494

Table S579 Coordinates of QM63.

Element	X	Y	Z
C	–2.682734	–1.363464	–0.415250
C	–1.372013	–0.918051	–0.567012
C	–1.004578	0.384439	–0.201443
C	–1.988942	1.218565	0.347476
C	–3.299522	0.774595	0.504235
C	–3.653139	–0.518277	0.120838
H	–2.946603	–2.373207	–0.717185
H	–0.627260	–1.582789	–0.997211
H	–1.712967	2.21905	0.670501
H	–4.044274	1.438078	0.935111
H	–4.674129	–0.867178	0.244899
C	0.391973	0.868721	–0.375643
C	0.61487	2.105437	–0.847376
H	–0.201235	2.75736	–1.160364
H	1.618253	2.511588	–0.953205
Si	1.85562	–0.225565	0.134759
C	3.38801	0.859601	0.288826
H	3.227193	1.689018	0.985952
H	4.227253	0.263262	0.665474
H	3.692103	1.279317	–0.676579
C	2.212492	–1.561637	–1.151874
H	1.475661	–2.371268	–1.137980
H	2.235516	–1.139927	–2.162689
H	3.19367	–2.009820	–0.954015
C	1.462631	–1.028281	1.79054
H	1.296412	–0.270473	2.564098
H	0.558812	–1.644496	1.729456
H	2.288621	–1.670495	2.117217

Table S70 Coordinates of QM64.

Element	X	Y	Z
C	2.503633	2.848349	–0.193939
C	1.593958	1.868585	–0.585231
C	1.96564	0.513114	–0.587574
C	3.26505	0.176089	–0.182711
C	4.177485	1.15851	0.194256
C	3.797827	2.499882	0.192955
H	2.191976	3.889359	–0.185148
H	0.56423	2.13673	–0.827071
H	3.578783	–0.865427	–0.180050
H	5.183423	0.875256	0.491223
H	4.504433	3.26754	0.496063
C	0.047448	–0.275519	–1.911417
H	–0.673795	–1.032132	–2.219706
H	–0.033225	0.690929	–2.408392
C	–1.357787	3.477475	0.241085
H	–2.240163	4.145827	0.355463
H	–0.635380	4.06189	–0.381649
H	–0.901748	3.377884	1.236491
C	–2.257655	2.400826	–1.614714
H	–1.567250	2.903173	–2.336500
H	–3.177224	3.03144	–1.608715

H	–2.538020	1.43967	–2.081258
C	1.000208	–0.543217	–0.997491
N	–1.684791	2.199296	–0.314571
Li	–1.456085	0.412883	0.015139
O	–0.951611	–0.234464	1.748645
O	–3.142827	–0.596022	–0.253561
C	–1.684131	–1.101066	2.591288
H	–2.273978	–1.760877	1.952348
H	–2.356874	–0.525620	3.241959
H	–0.999280	–1.693775	3.212356
C	–0.288476	0.798197	2.46674
H	–1.025458	1.45835	2.941613
H	0.300706	1.36865	1.745686
H	0.370104	0.365149	3.231372
C	–3.657968	–1.192742	–1.428511
H	–4.515386	–1.833046	–1.183460
H	–2.866815	–1.804253	–1.865264
H	–3.971296	–0.424332	–2.146695
C	–4.117265	0.21621	0.392883
H	–4.427167	1.035692	–0.266161
H	–3.647962	0.650531	1.27652
H	–4.981407	–0.400010	0.675701
Si	1.091431	–2.294296	–0.262918
C	1.6597	–2.254769	1.536918
H	1.223568	–3.093319	2.093006
H	2.748729	–2.331519	1.621515
H	1.352581	–1.324249	2.023076
C	–0.624351	–3.069241	–0.391684
H	–0.873700	–3.314334	–1.430920
H	–0.658825	–4.004967	0.1788
H	–1.402242	–2.403508	–0.000321
C	2.279731	–3.355478	–1.274645
H	1.97614	–3.376116	–2.327256
H	3.309292	–2.984225	–1.233843
H	2.282208	–4.387287	–0.903280

Table S71 Coordinates of QM65.

Element	X	Y	Z
C	–1.138047	2.724618	–1.428469
C	–0.510463	1.481839	–1.494229
C	0.767931	1.261813	–0.935180
C	1.374455	2.372803	–0.312291
C	0.75184	3.614238	–0.248419
C	–0.517571	3.80233	–0.799746
H	–2.123007	2.843721	–1.874197
H	–1.030187	0.675792	–2.010320
H	2.361795	2.262273	0.125008
H	1.261647	4.442767	0.236432
H	–1.005923	4.770863	–0.747887
C	0.900439	–1.059712	–1.726721
H	0.184028	–0.870974	–2.523737
N	–1.017095	–2.241340	–0.744840
C	–1.535599	–2.892428	–1.910965
H	–0.739200	–3.365470	–2.530256

H	–2.255705	–3.709415	–1.685233
H	–2.057234	–2.174654	–2.562889
C	–0.300602	–3.186151	0.062711
H	–0.954236	–3.947858	0.542034
H	0.455548	–3.767752	–0.511406
H	0.242842	–2.670184	0.871633
Li	–1.261265	–0.503816	–0.125029
C	1.416055	–0.067663	–0.946207
O	–1.047513	–0.096720	1.739796
O	–3.156905	0.056989	–0.285115
C	–4.094580	–1.006984	–0.322074
H	–4.303888	–1.365099	0.695757
H	–5.029938	–0.667978	–0.786455
H	–3.644946	–1.813720	–0.899363
C	–3.598369	1.130318	0.519302
H	–3.766600	0.792388	1.552593
H	–2.818909	1.895598	0.506816
H	–4.532761	1.549681	0.122864
C	–1.593002	–1.095140	2.583162
H	–1.811703	–1.962002	1.955141
H	–0.870474	–1.371886	3.362571
H	–2.515762	–0.731641	3.058079
C	–0.767708	1.113927	2.415908
H	–1.688648	1.529792	2.848434
H	–0.036900	0.943524	3.216559
H	–0.352842	1.809332	1.682403
H	1.340366	–2.053292	–1.750353
Si	2.869872	–0.482831	0.170474
C	2.447779	–0.198481	1.992881
H	1.573147	–0.794874	2.27967
H	3.287859	–0.489328	2.635034
H	2.220853	0.854405	2.197272
C	4.416585	0.534113	–0.243978
H	4.380052	1.567092	0.116746
H	5.294804	0.061613	0.213012
H	4.580194	0.564504	–1.327067
C	3.360528	–2.287308	–0.061186
H	4.216248	–2.510651	0.587259
H	2.554428	–2.981219	0.195559
H	3.66764	–2.489835	–1.093806

Table S72 Coordinates of QM66.

Element	X	Y	Z
C	–1.857599	2.432234	–1.350163
C	–0.973143	1.351964	–1.391766
C	0.190568	1.272307	–0.553965
C	0.302161	2.360201	0.378095
C	–0.594486	3.410576	0.422377
C	–1.693445	3.477831	–0.449658
H	–2.692594	2.449992	–2.050128
H	–1.158906	0.577589	–2.133897
H	1.143023	2.372671	1.063343
H	–0.432294	4.205704	1.147248
H	–2.383392	4.315035	–0.422571

C	0.84215	–0.873328	–1.646927
H	0.540381	–0.422269	–2.605436
H	1.735604	–1.482386	–1.875060
N	–0.264601	–1.822716	–1.272855
C	–0.828304	–2.454572	–2.459079
H	–0.059112	–2.998200	–3.034752
H	–1.605753	–3.170301	–2.169731
H	–1.272218	–1.695290	–3.111900
C	0.247201	–2.852049	–0.369230
H	–0.577249	–3.481355	–0.014408
H	0.990766	–3.496919	–0.870649
H	0.732017	–2.373369	0.487519
Li	–1.025143	–0.500412	0.117518
C	1.106411	0.168815	–0.586883
O	–0.910441	–0.897883	2.016143
O	–3.000664	–0.593275	0.21397
C	–3.720214	–0.756027	–0.993156
H	–4.757792	–1.045087	–0.780089
H	–3.712964	0.174765	–1.574919
H	–3.226883	–1.546131	–1.560957
C	–3.545292	0.443665	1.02101
H	–4.583019	0.204198	1.287712
H	–2.931651	0.502326	1.921099
H	–3.502599	1.401536	0.488534
C	–1.509348	–2.050018	2.57547
H	–2.324046	–2.342833	1.910311
H	–0.776658	–2.864670	2.655968
H	–1.908544	–1.825507	3.573626
C	0.178929	–0.411553	2.782048
H	–0.147125	–0.211474	3.811289
H	0.99955	–1.141626	2.790836
H	0.513808	0.511216	2.307941
Si	2.85835	0.182285	–0.007498
C	3.343129	1.327401	1.428885
H	4.381994	1.094847	1.696077
H	3.310924	2.385426	1.147301
H	2.738465	1.19549	2.333563
C	4.033047	0.662236	–1.422607
H	3.815468	1.679498	–1.769147
H	5.084766	0.625031	–1.111296
H	3.914937	–0.008892	–2.282320
C	3.424615	–1.548350	0.544123
H	2.916932	–1.871941	1.461211
H	3.253733	–2.316126	–0.219292
H	4.501737	–1.531430	0.753033

Table S73 Coordinates of QM67.

Element	X	Y	Z
C	–0.581491	2.389256	–1.586221
C	0.410786	1.420233	–1.732771
C	1.109827	0.92593	–0.617590
C	0.810564	1.470789	0.640818
C	–0.184044	2.438189	0.789729
C	–0.893842	2.892192	–0.321765

H	–1.126352	2.739748	–2.457811
H	0.652334	1.035577	–2.720010
H	1.336808	1.108821	1.521081
H	–0.425366	2.817332	1.77834
H	–1.697165	3.611636	–0.200385
C	1.88358	–1.178741	–1.595333
H	2.61894	–1.961756	–1.769954
H	0.929645	–1.285826	–2.115201
C	–3.679961	1.421449	1.42302
H	–4.444195	1.124105	2.183946
H	–3.865128	2.510194	1.244676
H	–2.701571	1.354341	1.93413
C	–4.997371	0.743262	–0.363712
H	–5.262826	1.790225	–0.645944
H	–5.827709	0.404843	0.301395
H	–5.070407	0.145354	–1.285127
C	2.13278	–0.144596	–0.774408
N	–3.703120	0.620635	0.234546
Na	–1.630272	0.09896	–0.220598
O	–1.586177	–1.750845	–1.575465
O	–1.105662	–1.175650	1.574326
C	–2.932590	–1.702759	–2.034686
H	–3.110945	–2.502761	–2.765360
H	–3.079493	–0.728972	–2.507000
H	–3.627747	–1.783624	–1.189168
C	–1.288526	–2.981533	–0.949045
H	–0.257902	–2.926710	–0.589678
H	–1.385267	–3.808137	–1.666548
H	–1.959546	–3.157385	–0.097296
C	–2.240759	–1.491460	2.370555
H	–2.328812	–2.582197	2.480787
H	–3.109334	–1.081591	1.842305
H	–2.144723	–1.035560	3.365121
C	0.09923	–1.678094	2.098332
H	0.327234	–1.220003	3.072033
H	0.893306	–1.437749	1.385729
H	0.043633	–2.769769	2.224514
Si	3.801693	–0.012672	0.124571
C	5.053015	–1.088227	–0.780486
H	6.045102	–0.960659	–0.332272
H	5.125165	–0.816478	–1.839094
H	4.800937	–2.152719	–0.718701
C	3.682583	–0.632384	1.904959
H	3.007444	–0.037191	2.529079
H	4.67545	–0.593632	2.369314
H	3.344131	–1.674496	1.936491
C	4.348748	1.786691	0.107559
H	4.427693	2.162268	–0.918663
H	5.328814	1.89995	0.584738
H	3.638529	2.428008	0.640679

Table S74 Coordinates of QM68.

Element	X	Y	Z
C	–0.718358	3.838673	–0.802669

C	–0.104086	2.684625	–1.276245
C	0.528957	1.77551	–0.404433
C	0.503544	2.089487	0.968726
C	–0.113671	3.245552	1.447052
C	–0.727158	4.130762	0.563663
H	–1.180598	4.525901	–1.506764
H	–0.076351	2.491031	–2.345251
H	0.989951	1.420015	1.676922
H	–0.106536	3.456194	2.513336
H	–1.196712	5.038777	0.930457
C	0.659649	–0.141612	–1.952216
H	–0.219237	0.221591	–2.485962
H	1.185457	–0.957270	–2.441703
N	–0.794605	–2.032486	–1.429664
C	–1.500564	–2.076626	–2.675556
H	–0.825792	–1.960951	–3.558991
H	–2.043884	–3.030760	–2.846680
H	–2.240870	–1.265428	–2.737557
C	0.175681	–3.088092	–1.408212
H	–0.272017	–4.106734	–1.401029
H	0.85713	–3.078801	–2.292489
H	0.818614	–3.016920	–0.515372
C	1.205599	0.553687	–0.909089
O	–0.835958	–1.807298	2.120906
O	–2.960108	0.274851	–0.066791
C	–3.900995	–0.701126	–0.474941
H	–4.693229	–0.806610	0.278894
H	–4.350985	–0.421172	–1.436942
H	–3.355079	–1.641565	–0.592222
C	–3.509703	1.57159	0.026495
H	–4.350046	1.584788	0.734696
H	–2.720116	2.238748	0.378002
H	–3.860800	1.911986	–0.957074
C	–0.991710	–3.188024	1.82686
H	–1.310238	–3.256631	0.783311
H	–0.035678	–3.714344	1.956141
H	–1.743965	–3.632749	2.491945
C	–0.412402	–1.582481	3.448054
H	–1.148834	–1.978958	4.159692
H	0.561335	–2.057151	3.63014
H	–0.319279	–0.503056	3.587235
Na	–0.893849	–0.552718	0.228065
Si	2.790221	–0.072331	–0.123322
C	3.658216	–1.240882	–1.317947
H	3.833888	–0.760517	–2.286875
H	4.630686	–1.537028	–0.907017
H	3.078653	–2.153808	–1.494162
C	2.483192	–1.035748	1.485739
H	3.400827	–1.553296	1.792881
H	2.183067	–0.388049	2.31825
H	1.701744	–1.793652	1.349471
C	3.93003	1.376567	0.278171
H	4.860816	1.021884	0.736405
H	4.189067	1.92929	–0.631731

H	3.461693	2.083586	0.971841

Table S75 Coordinates of QM69.

Element	X	Y	Z
C	1.811885	–1.019851	–2.383305
C	0.862312	–0.139685	–1.865277
C	–0.372681	–0.584204	–1.281494
C	–0.528813	–2.010822	–1.281392
C	0.416806	–2.872257	–1.806760
C	1.617049	–2.399638	–2.360368
H	2.719761	–0.611372	–2.826480
H	1.068742	0.92867	–1.920305
H	–1.441407	–2.438591	–0.876697
H	0.217803	–3.942295	–1.788576
H	2.352439	–3.081098	–2.775700
C	–1.180910	1.772618	–1.040076
H	–0.815051	1.905674	–2.069636
H	–2.143230	2.311997	–0.983203
N	–0.201889	2.531938	–0.176505
C	0.198339	3.766364	–0.837734
H	–0.669390	4.41315	–1.060559
H	0.889477	4.330928	–0.201559
H	0.700599	3.535601	–1.783616
C	–0.809802	2.8526	1.110544
H	–0.077586	3.339263	1.765544
H	–1.679856	3.525092	0.998579
H	–1.166511	1.932833	1.590617
C	–1.338723	0.312654	–0.714310
O	1.254779	–1.148348	2.117837
O	3.08377	0.992236	0.559401
C	3.422971	1.877844	–0.491273
H	3.504485	1.336175	–1.443373
H	2.623152	2.617433	–0.562928
H	4.375361	2.379812	–0.274254
C	4.02618	–0.055414	0.691179
H	5.032887	0.355408	0.845488
H	3.734745	–0.644410	1.561838
H	4.022444	–0.689423	–0.206489
C	0.829514	–1.394635	3.443537
H	0.621709	–0.428662	3.908109
H	–0.081862	–2.005452	3.450937
H	1.619357	–1.907813	4.008345
C	1.541012	–2.346808	1.411298
H	2.359704	–2.888796	1.905396
H	0.650335	–2.984915	1.36075
H	1.833305	–2.076846	0.392124
Na	0.853017	0.540412	0.664734
Si	–2.875015	–0.295440	0.092917
C	–2.481555	–1.299976	1.666602
H	–2.102178	–0.628510	2.449035
H	–3.361547	–1.818252	2.067345
H	–1.705180	–2.051374	1.472746
C	–3.982405	1.145892	0.641422
H	–3.502365	1.822461	1.35652

H	–4.318410	1.744716	–0.213448
H	–4.878680	0.738053	1.125241
C	–4.026411	–1.366829	–0.978324
H	–3.567604	–2.291785	–1.341619
H	–4.925880	–1.641085	–0.412233
H	–4.346336	–0.796749	–1.858820

Table S586 Coordinates of QM70.

Element	X	Y	Z
C	2.394688	2.316255	1.314999
C	1.897424	1.102588	0.845517
C	2.314126	0.594469	–0.393631
C	3.263441	1.31121	–1.134422
C	3.774701	2.51696	–0.653941
C	3.337453	3.026232	0.569035
H	2.050595	2.698574	2.272952
H	1.169388	0.536262	1.425966
H	3.611326	0.908776	–2.083067
H	4.51919	3.056585	–1.233071
H	3.736655	3.964856	0.943091
C	1.210652	–0.748405	–2.125075
H	0.803618	–1.676513	–2.523776
H	1.178694	0.114499	–2.793021
C	–0.857841	0.510271	2.834231
H	–0.029247	0.398525	3.578202
H	–1.767846	0.692504	3.459551
H	–0.664909	1.454872	2.297275
C	–1.296145	–1.755714	2.7134
H	–2.259966	–1.663722	3.276416
H	–0.538758	–1.988641	3.499649
H	–1.383898	–2.659222	2.092392
C	1.740152	–0.683981	–0.892176
N	–0.982821	–0.597533	1.929642
O	–2.111632	–1.389720	–1.510873
C	–2.992602	–2.063033	–0.627428
H	–2.832102	–3.148875	–0.682917
H	–4.036894	–1.832404	–0.883058
H	–2.768555	–1.708671	0.383692
C	–2.196248	–1.866730	–2.831633
H	–1.889313	–2.921245	–2.885354
H	–1.526274	–1.261478	–3.446030
H	–3.222167	–1.773909	–3.215956
O	–3.257799	0.910048	0.115344
C	–3.964779	0.598101	1.30205
H	–3.269467	0.029508	1.925005
H	–4.857007	–0.001805	1.069083
H	–4.278266	1.519382	1.814417
C	–4.037419	1.597822	–0.831823
H	–4.414707	2.545248	–0.418169
H	–4.894875	0.987287	–1.151352
H	–3.395752	1.808972	–1.690243
Na	–1.066658	0.163067	–0.179078
O	–0.810779	2.396148	–0.797372
C	0.184843	2.945216	–1.640061

H	0.455556	2.182278	–2.371629
H	1.075716	3.222148	–1.062188
H	–0.206318	3.8301	–2.161743
C	–1.196237	3.315843	0.208145
H	–1.605656	4.228226	–0.248659
H	–0.333058	3.57922	0.834718
H	–1.960666	2.830885	0.81709
Si	1.896494	–2.289336	0.134322
C	2.195258	–1.967291	1.955161
H	2.338349	–2.922732	2.474617
H	3.092493	–1.359055	2.117402
H	1.329119	–1.458270	2.391819
C	0.346843	–3.313427	–0.155362
H	–0.526062	–2.728259	0.149002
H	0.230303	–3.596673	–1.208500
H	0.368192	–4.232871	0.441082
C	3.39541	–3.179260	–0.596196
H	3.586465	–4.123921	–0.073729
H	3.239877	–3.403234	–1.657814
H	4.297652	–2.562090	–0.512849

Table 597 Coordinates of QM71.

Element	X	Y	Z
C	–1.251762	–1.462858	2.741264
H	–0.436004	–1.488116	3.504641
H	–0.993084	–2.210573	1.972708
H	–2.148306	–1.852500	3.271237
C	–3.927935	–1.678723	0.494348
H	–3.532317	–1.358991	1.46075
H	–4.194208	–2.744989	0.515068
H	–4.825587	–1.093331	0.24723
O	–2.906329	–1.442291	–0.454119
C	–3.329447	–1.637311	–1.783130
H	–3.701837	–2.661828	–1.930976
H	–2.464174	–1.466238	–2.427504
H	–4.128446	–0.927179	–2.044369
C	0.694628	0.821706	2.047633
H	0.870039	0.089848	2.836253
H	0.142418	1.702075	2.358665
C	1.443945	0.844849	0.896586
N	–1.458593	–0.166852	2.157111
C	–1.948398	0.718488	3.1736
H	–1.242062	0.841803	4.028991
H	–2.901550	0.371645	3.626104
H	–2.129130	1.724471	2.765521
C	2.323972	–0.298715	0.574882
C	2.094275	–1.584676	1.106477
C	3.445162	–0.154138	–0.265080
C	2.954001	–2.647914	0.840349
H	1.228662	–1.751529	1.742884
C	4.301331	–1.216866	–0.538795
H	3.670394	0.81948	–0.692423
C	4.065562	–2.475671	0.015129
H	2.750765	–3.621271	1.281317

H	5.163479	–1.057354	–1.181512
H	4.735435	–3.305198	–0.191514
C	–0.366592	–3.222753	–1.050381
H	–0.546826	–3.838216	–1.943709
H	–1.264410	–3.205998	–0.430045
H	0.474908	–3.644994	–0.484690
C	–2.388287	2.204713	–2.240964
H	–2.256581	3.258854	–1.957817
H	–3.381441	2.075469	–2.693957
H	–1.622303	1.927643	–2.966891
O	–0.085760	–1.886570	–1.424674
O	–2.246266	1.366596	–1.115888
C	–3.277662	1.577318	–0.163819
H	–3.046775	0.958482	0.709362
H	–4.249836	1.286603	–0.589059
H	–3.317598	2.636584	0.127816
C	1.079541	–1.809102	–2.229134
H	1.2815	–0.754824	–2.420705
H	0.913251	–2.333140	–3.180952
H	1.938465	–2.249583	–1.708602
Na	–0.930990	–0.251676	–0.029763
Si	1.39883	2.40812	–0.133724
C	2.943027	3.485725	0.049984
H	2.789203	4.448382	–0.453363
H	3.845586	3.032251	–0.372314
H	3.139205	3.688482	1.108818
C	1.190449	2.02318	–1.980252
H	0.995825	2.943271	–2.545579
H	0.348734	1.341946	–2.154427
H	2.087839	1.55823	–2.404615
C	–0.065455	3.464255	0.423528
H	0.10723	3.902106	1.413248
H	–0.990362	2.878924	0.469449
H	–0.217971	4.291109	–0.281198

Table S608 Coordinates of QM72.

Element	X	Y	Z
C	1.039375	3.588335	0.190264
C	1.563128	2.307318	0.209316
C	1.178042	1.291361	–0.726296
C	0.188162	1.722648	–1.670868
C	–0.315158	3.021197	–1.692103
C	0.086564	3.977379	–0.762142
H	1.392806	4.309851	0.92486
H	2.322152	2.074714	0.953109
H	–0.176865	1.020997	–2.418592
H	–1.037849	3.289141	–2.463294
H	–0.307726	4.988354	–0.784022
C	1.373343	–0.907488	–1.885186
H	2.137721	–1.683738	–2.062702
H	1.3331	–0.308998	–2.807775
C	0.192614	–2.824140	–1.006051
H	0.911821	–3.528528	–1.464137
H	–0.773103	–3.328467	–0.895459

H	0.567318	–2.559792	–0.010524
C	–0.421518	–1.974103	–3.136928
H	–1.374623	–2.512875	–3.071132
H	0.300377	–2.620491	–3.669134
H	–0.573169	–1.068510	–3.734957
C	1.71004	–0.042809	–0.700595
N	0.043378	–1.614296	–1.804941
O	–2.877685	0.619271	–0.662917
C	–3.125767	0.383778	–2.034021
H	–2.654298	1.161129	–2.650667
H	–4.206264	0.365649	–2.236015
H	–2.683602	–0.584080	–2.285177
C	–3.311650	1.906038	–0.258114
H	–2.886794	2.676721	–0.911302
H	–2.943253	2.068928	0.755494
H	–4.408988	1.961584	–0.279506
O	–2.040391	–1.959272	0.912956
C	–1.494751	–2.708701	1.976192
H	–0.649488	–2.140955	2.374348
H	–1.143769	–3.689378	1.624927
H	–2.241919	–2.856835	2.768933
C	–3.184499	–2.560453	0.351489
H	–3.968368	–2.685179	1.112545
H	–2.943815	–3.545594	–0.073966
H	–3.546746	–1.894619	–0.433625
Na	–0.820370	–0.180681	0.01736
O	–1.378561	0.623718	2.112608
C	–0.467178	1.302345	2.958861
H	0.421521	1.521396	2.366613
H	–0.201270	0.674541	3.820779
H	–0.906022	2.244548	3.314369
C	–2.600959	0.342394	2.758461
H	–3.049094	1.265702	3.152913
H	–2.447693	–0.358860	3.59195
H	–3.266241	–0.106399	2.01885
Si	3.099176	–0.521471	0.406131
C	3.661022	–2.303347	0.06682
H	2.873006	–3.045626	0.236145
H	4.028275	–2.432844	–0.957854
H	4.491241	–2.543342	0.742755
C	2.67571	–0.481820	2.268433
H	3.463462	–0.982314	2.846279
H	2.583248	0.536571	2.663652
H	1.729767	–1.002993	2.468067
C	4.673818	0.529034	0.224874
H	4.48367	1.600199	0.351957
H	5.433591	0.23186	0.959304
H	5.100921	0.393175	–0.775828

Table S619 Coordinates of QM73.

Element	X	Y	Z
C	–0.035873	1.012151	2.906089
C	–0.971147	0.564078	1.973485
C	–0.930916	–0.746461	1.472291

C	0.067897	–1.606617	1.964069
C	1.005344	–1.158949	2.889941
C	0.96224	0.15392	3.36364
H	–0.091609	2.031229	3.280926
H	–1.729377	1.255327	1.61308
H	0.117725	–2.629261	1.603747
H	1.775048	–1.841465	3.240908
H	1.692058	0.500531	4.090479
C	2.04725	–3.577586	–0.132931
H	1.187512	–4.300866	–0.152212
H	2.896528	–4.143885	–0.585143
H	2.301005	–3.428915	0.93026
C	1.528473	–2.586917	–2.162538
H	2.385797	–3.063708	–2.698096
H	0.66052	–3.268890	–2.363715
H	1.310514	–1.650275	–2.712643
N	1.771869	–2.331902	–0.774618
O	–0.470627	1.185626	–2.389949
C	–1.268254	0.310991	–3.164851
H	–2.291200	0.703839	–3.263027
H	–0.834744	0.176678	–4.164927
H	–1.294178	–0.650120	–2.646953
C	–0.371532	2.470487	–2.966069
H	–1.357671	2.957324	–3.000655
H	0.306849	3.055012	–2.341234
H	0.031012	2.409369	–3.986591
O	3.892504	0.400094	–0.225998
C	4.011275	–0.392512	0.943833
H	3.586053	0.184869	1.771168
H	3.458486	–1.334516	0.815861
H	5.068996	–0.601721	1.159257
C	4.417043	–0.285156	–1.355336
H	5.497358	–0.447202	–1.230932
H	3.895873	–1.244775	–1.479948
H	4.252831	0.355734	–2.225714
K	1.170575	0.158075	–0.495677
O	1.069279	2.785131	0.118754
C	–0.050608	3.598373	0.380812
H	–0.900995	3.169876	–0.156091
H	–0.277669	3.621401	1.456149
H	0.123665	4.627364	0.033389
C	2.218399	3.190102	0.837607
H	2.041807	3.110835	1.920212
H	3.034935	2.524318	0.546465
H	2.483663	4.227914	0.591886
C	–1.471923	–2.156714	–0.452657
H	–2.137373	–2.518392	–1.234933
H	–0.454317	–2.563296	–0.438980
C	–1.867008	–1.192343	0.403018
Si	–3.569644	–0.395984	0.18936
C	–4.532471	–1.367715	–1.105806
H	–5.546137	–0.959413	–1.192770
H	–4.064822	–1.301316	–2.095245
H	–4.618846	–2.427275	–0.842462

C	–3.402009	1.392396	–0.431306
H	–4.236853	1.642844	–1.097220
H	–3.413059	2.119296	0.389606
H	–2.467501	1.523952	–0.990890
C	–4.504280	–0.404864	1.824459
H	–4.666288	–1.429843	2.175451
H	–3.951473	0.129063	2.605453
H	–5.483775	0.075223	1.716265

Table S80 Coordinates of QM74.

Element	X	Y	Z
C	–0.663251	–2.635696	–2.805298
C	–1.264884	–1.439011	–2.425518
C	–1.553614	–1.150938	–1.075157
C	–1.226368	–2.145277	–0.132216
C	–0.638680	–3.351215	–0.512345
C	–0.342747	–3.603167	–1.850927
H	–0.460609	–2.822957	–3.856724
H	–1.538436	–0.712967	–3.186865
H	–1.423922	–1.962026	0.920689
H	–0.407517	–4.096198	0.245567
H	0.115984	–4.541480	–2.149067
C	–1.711791	1.289383	–1.320302
H	–1.110450	1.252185	–2.226835
H	–2.167211	2.258911	–1.125122
N	0.262207	2.578281	–0.704983
C	0.116869	3.552088	–1.741232
H	–0.930464	3.930504	–1.855842
H	0.73044	4.467906	–1.583337
H	0.411361	3.137472	–2.718135
C	–0.126674	3.16953	0.53973
H	0.527004	4.015953	0.855679
H	–1.160979	3.590204	0.531689
H	–0.095513	2.42749	1.355135
C	–2.112591	0.160044	–0.661133
O	3.570946	1.403863	–0.710116
O	3.087654	–1.580991	0.357491
O	1.023614	–0.282408	2.2037
C	1.969595	–0.530398	3.219087
H	1.668849	–1.394381	3.829831
H	2.92204	–0.744656	2.729524
H	2.078394	0.347219	3.87174
C	–0.259528	–0.001013	2.724428
H	–0.231404	0.875566	3.386568
H	–0.915207	0.214116	1.875451
H	–0.648288	–0.865079	3.28357
C	3.462318	1.86175	0.624315
H	3.456345	0.976611	1.267671
H	4.320737	2.495929	0.888111
H	2.529435	2.429087	0.757731
C	3.566469	2.487001	–1.625703
H	4.442742	3.129725	–1.457968
H	3.621987	2.061424	–2.630242
H	2.642093	3.066307	–1.515220

C	4.43148	–1.627753	–0.080297
H	4.661809	–0.648767	–0.503788
H	5.102705	–1.840457	0.764598
H	4.564759	–2.407569	–0.843143
C	2.659242	–2.803144	0.916789
H	3.307533	–3.097155	1.756002
H	1.639567	–2.652589	1.279324
H	2.665889	–3.602725	0.162563
K	1.130669	0.120899	–0.475538
Si	–3.571213	0.312272	0.513751
C	–3.504534	1.915894	1.505669
H	–3.507228	2.796592	0.85464
H	–4.385297	1.98047	2.156796
H	–2.610220	1.981176	2.135318
C	–3.769092	–1.125043	1.731985
H	–4.706797	–0.978947	2.282538
H	–3.836340	–2.089926	1.217118
H	–2.963798	–1.189346	2.472364
C	–5.146097	0.335487	–0.531289
H	–6.045462	0.410691	0.09177
H	–5.135599	1.187301	–1.220807
H	–5.224534	–0.577309	–1.133411

Table S81 Coordinates of QM75.

QM72.log			
C	–1.055265	0.048729	2.905298
C	–0.055287	0.642915	2.141695
C	0.953465	–0.112703	1.447178
C	0.829148	–1.532851	1.632786
C	–0.169450	–2.109789	2.400611
C	–1.144261	–1.337411	3.048619
H	–1.778169	0.6911	3.408429
H	–0.042881	1.727083	2.074577
H	1.546185	–2.187830	1.149007
H	–0.189936	–3.194881	2.495996
H	–1.916922	–1.796597	3.658555
C	1.798379	1.989843	0.406503
H	1.693867	2.504625	1.375816
H	2.71357	2.41768	–0.049943
N	0.635494	2.438968	–0.417663
C	0.386054	3.857555	–0.224678
H	1.258767	4.477447	–0.504788
H	–0.467805	4.174637	–0.834915
H	0.154085	4.055669	0.827977
C	0.885877	2.18058	–1.827607
H	–0.012918	2.400244	–2.418645
H	1.715033	2.798051	–2.222768
H	1.169616	1.130401	–1.965144
C	1.903298	0.491926	0.571143
O	–3.020627	1.93749	–0.638139
O	–3.068590	–1.503614	0.033112
O	–0.472698	–1.901067	–2.239533
C	–1.078613	–2.821898	–3.117286
H	–1.118012	–3.824469	–2.667130

H	–2.095402	–2.472701	–3.308546
H	–0.525325	–2.880533	–4.064565
C	0.840453	–2.283826	–1.867715
H	1.485056	–2.371590	–2.753376
H	1.236356	–1.503918	–1.207179
H	0.825627	–3.242850	–1.330352
C	–4.260988	2.148266	–1.274182
H	–4.333264	1.435998	–2.098168
H	–5.093653	1.979982	–0.575772
H	–4.329309	3.172041	–1.666688
C	–2.844635	2.770998	0.491487
H	–3.610306	2.559837	1.252011
H	–1.852886	2.559793	0.898639
H	–2.903358	3.830339	0.205614
C	–4.113115	–1.044607	0.863416
H	–4.186826	0.036291	0.721013
H	–5.066732	–1.518046	0.587342
H	–3.889989	–1.256352	1.917445
C	–2.832808	–2.887545	0.187397
H	–3.687644	–3.469388	–0.187525
H	–1.937457	–3.128771	–0.390100
H	–2.653943	–3.132950	1.241783
K	–1.050688	0.105812	–0.574320
Si	3.543978	–0.171498	0.064119
C	3.917818	0.200377	–1.766725
H	3.836001	1.271852	–1.986464
H	4.94033	–0.108823	–2.018292
H	3.23259	–0.327831	–2.441964
C	3.859402	–2.033177	0.278893
H	4.889423	–2.235134	–0.042077
H	3.77385	–2.347174	1.325029
H	3.201674	–2.672065	–0.322759
C	4.958327	0.664146	1.024447
H	5.940884	0.32987	0.667174
H	4.921372	1.755717	0.924696
H	4.885901	0.429361	2.092914

4.2.3 Calculated Aminometalation Reaction of Dimethylamide to Styrene

In the following, the energies (Table S82) and the coordinates of the opitimized structures for the aminometalation of different metal dimethylamides to styrene (Scheme S11) are listed.

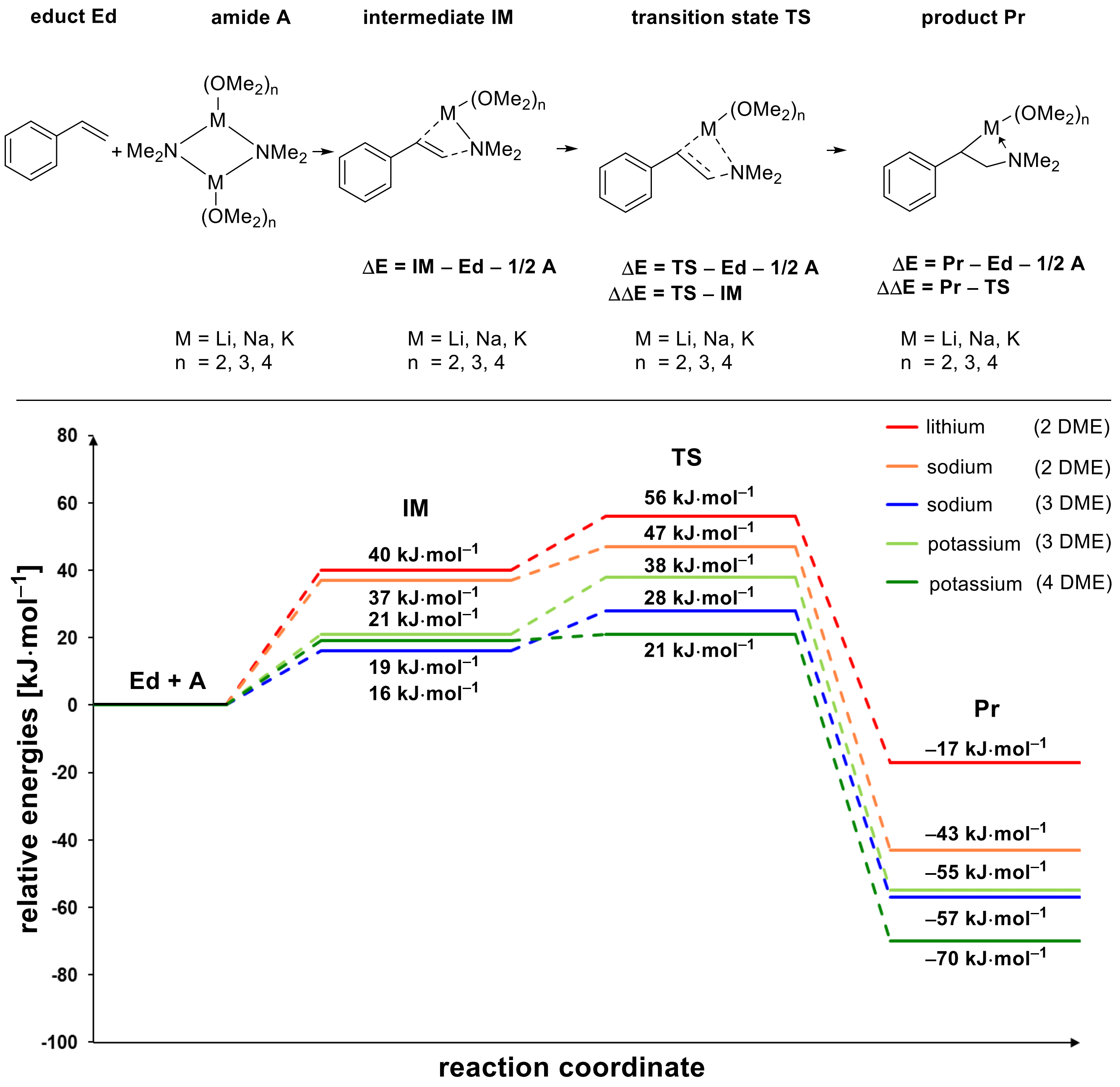

Scheme S11 Calculated reaction scheme for the aminometalation of different metal dimethylamides to styrene.

Table S82 Total (SCF) and zero-point-corrected (ZPE) energies of the optimized structures of the elimination reaction of a metal dimethylamide and styrene.

Optimized structure		SCF [Hartree]	ZPE [Hartree]
Styrene	QM76	–309.50976171	–309.375123
A Li + 2 DME	QM44	–904.007590038	–903.508184
A Na + 2 DME	QM45	–1213.41663972	–1212.924143
A Na + 3 DME	QM46	–1523.34423200	–1522.763223
A K + 3 DME	QM47	–2398.56867462	–2397.912793
A K + 4 DME	QM47b	-2708.49244230	-2707.668902
IM Li + 2 DME	QM77	–761.498624237	–761.113944
TS Li + 2 DME	QM78	–761.492455772	–761.107705
Pr Li +2 DME	QM79	–761.52200082	–761.133823
IM Na + 2 DME	QM80	–916.205435694	–915.823028
TS Na + 2 DME	QM81	–916.201667889	–915.819108
Pr Na + 2 DME	QM82	–916.234392995	–915.848344
IM Na + 3 DME	QM83	-1071.17549136	–1070.710740
TS Na + 3 DME	QM84	–1071.17289819	–1070.706159
Pr Na + 3 DME	QM85	–1071.20250169	–1070.733206
IM K + 3 DME	QM86	–1508.78631718	–1508.323424
TS K + 3 DME	QM87	–1508.78075067	–1508.316921
Pr K + 3 DME	QM88	–1508.81840158	–1508.351794
IM K + 4 DME	QM89	-1663.74806033	–1663.276543
TS K + 4 DME	QM90	-1663.74729996	–1663.201541
Pr K + 4 DME	QM91	-1663.78156969	–1663.230375

Table S83 Coordinates of QM76.

Element	X	Y	Z
C	0.407627	–1.280748	0.000007
C	1.780248	–1.041826	0.000145
C	2.259104	0.265753	0.000125
C	1.354226	1.328991	–0.000069
C	–0.014698	1.086967	–0.000213
C	–0.511539	–0.224377	–0.000137
H	0.038021	–2.303844	0.000037
H	2.473651	–1.877917	0.000279
H	3.327983	0.458052	0.000234
H	1.718911	2.352282	–0.000131
H	–0.702241	1.927832	–0.000418
C	–1.954104	–0.535112	–0.000247
H	–2.190389	–1.599088	–0.000801
C	–2.964447	0.338975	0.000327
H	–2.810471	1.41443	0.000983
H	–3.993968	–0.003490	0.000189

Table S84 Coordinates of QM77.

Element	X	Y	Z
C	–2.635921	–0.954429	1.006139
C	–1.424348	–1.302908	0.407206

C	–1.157612	–1.041256	–0.982332
C	–2.220961	–0.358982	–1.673332
C	–3.399140	–0.000800	–1.052440
C	–3.637147	–0.289422	0.305625
H	–2.797035	–1.218427	2.051374
H	–0.689480	–1.847994	1.000384
H	–2.094258	–0.162281	–2.737339
H	–4.169409	0.500006	–1.636029
H	–4.575365	–0.021536	0.78026
C	1.101949	–2.148951	–0.886819
H	0.654317	–2.941224	–0.266113
H	1.792148	–2.647195	–1.588186
N	1.92234	–1.310291	0.065412
C	2.514105	–2.124991	1.117795
H	3.174552	–2.909011	0.707501
H	3.108064	–1.496301	1.790473
H	1.723054	–2.612863	1.697347
C	2.974074	–0.618812	–0.675276
H	3.489355	0.096304	–0.023593
H	3.71845	–1.328703	–1.078058
H	2.522959	–0.078938	–1.514654
Li	0.448299	0.151722	0.063152
C	0.068281	–1.348753	–1.601316
H	0.168833	–1.192137	–2.671731
O	0.853209	1.854905	–0.836667
O	0.281189	1.103703	1.789946
C	0.306033	0.285301	2.944816
H	0.480519	0.897737	3.839298
H	–0.641435	–0.258147	3.051749
H	1.123442	–0.427543	2.820186
C	–0.819581	2.005031	1.80154
H	–0.775658	2.638178	2.697325
H	–0.737351	2.621191	0.904944
H	–1.765071	1.449118	1.775438
C	1.890969	2.708721	–0.399477
H	2.057356	2.497056	0.658473
H	2.812054	2.521001	–0.968712
H	1.59971	3.760243	–0.526267
C	0.537708	2.026152	–2.207854
H	0.291476	3.077608	–2.405991
H	1.385583	1.723481	–2.836912
H	–0.323710	1.392982	–2.420389

Table S85 Coordinates of QM78.

Element	X	Y	Z
C	–3.103097	–1.195374	0.736184
C	–1.912088	–1.418265	0.054053
C	–1.554052	–0.642116	–1.070079
C	–2.459180	0.367464	–1.460248
C	–3.646254	0.594439	–0.772001
C	–3.980253	–0.184894	0.336952
H	–3.347555	–1.815397	1.595333
H	–1.243672	–2.201360	0.40333
H	–2.224541	0.968155	–2.337337

H	–4.319056	1.379294	–1.108254
H	–4.908045	–0.013110	0.87418
C	0.664755	–1.752498	–1.530091
H	0.470979	–2.577820	–0.847658
H	1.463278	–1.928424	–2.245501
N	2.28641	–1.097500	–0.142428
C	2.666625	–2.343279	0.458303
H	2.805614	–3.156276	–0.290100
H	3.62134	–2.279013	1.015819
H	1.896694	–2.684484	1.16511
C	3.296343	–0.664099	–1.069746
H	4.239643	–0.367009	–0.570566
H	3.574375	–1.447494	–1.809029
H	2.941512	0.206527	–1.643453
Li	0.77461	–0.008830	–0.011837
C	–0.312488	–0.830410	–1.817917
H	–0.156236	–0.153692	–2.657338
O	1.13552	1.865278	–0.364523
O	0.144912	0.19126	1.786165
C	0.997598	–0.049493	2.886978
H	1.392097	0.897904	3.281341
H	0.454095	–0.571715	3.685207
H	1.820776	–0.665832	2.520218
C	–0.913419	1.084456	2.074032
H	–0.512337	2.051777	2.407507
H	–1.490549	1.219009	1.155829
H	–1.566520	0.66744	2.851397
C	2.332408	2.291755	0.259613
H	2.627656	1.500866	0.953603
H	3.127881	2.428308	–0.484660
H	2.165791	3.235548	0.796155
C	0.720315	2.709046	–1.416946
H	0.577916	3.734863	–1.051795
H	1.462194	2.709816	–2.227139
H	–0.228772	2.318415	–1.787346

Table S86 Coordinates of QM79.

Element	X	Y	Z
C	–2.635921	–0.954429	1.006139
C	–1.424348	–1.302908	0.407206
C	–1.157612	–1.041256	–0.982332
C	–2.220961	–0.358982	–1.673332
C	–3.399140	–0.000800	–1.052440
C	–3.637147	–0.289422	0.305625
H	–2.797035	–1.218427	2.051374
H	–0.689480	–1.847994	1.000384
H	–2.094258	–0.162281	–2.737339
H	–4.169409	0.500006	–1.636029
H	–4.575365	–0.021536	0.78026
C	1.101949	–2.148951	–0.886819
H	0.654317	–2.941224	–0.266113
H	1.792148	–2.647195	–1.588186
N	1.92234	–1.310291	0.065412
C	2.514105	–2.124991	1.117795

H	3.174552	–2.909011	0.707501
H	3.108064	–1.496301	1.790473
H	1.723054	–2.612863	1.697347
C	2.974074	–0.618812	–0.675276
H	3.489355	0.096304	–0.023593
H	3.71845	–1.328703	–1.078058
H	2.522959	–0.078938	–1.514654
Li	0.448299	0.151722	0.063152
C	0.068281	–1.348753	–1.601316
H	0.168833	–1.192137	–2.671731
O	0.853209	1.854905	–0.836667
O	0.281189	1.103703	1.789946
C	0.306033	0.285301	2.944816
H	0.480519	0.897737	3.839298
H	–0.641435	–0.258147	3.051749
H	1.123442	–0.427543	2.820186
C	–0.819581	2.005031	1.80154
H	–0.775658	2.638178	2.697325
H	–0.737351	2.621191	0.904944
H	–1.765071	1.449118	1.775438
C	1.890969	2.708721	–0.399477
H	2.057356	2.497056	0.658473
H	2.812054	2.521001	–0.968712
H	1.59971	3.760243	–0.526267
C	0.537708	2.026152	–2.207854
H	0.291476	3.077608	–2.405991
H	1.385583	1.723481	–2.836912
H	–0.323710	1.392982	–2.420389

Table S628 Coordinates of QM80.

Element	X	Y	Z
C	1.235726	–2.514622	0.020375
C	1.878683	–1.490177	0.711638
C	2.447638	–0.406995	0.022347
C	2.379665	–0.397133	–1.378024
C	1.731255	–1.419067	–2.072873
C	1.153537	–2.480061	–1.375158
H	0.789966	–3.340604	0.567486
H	1.93966	–1.520798	1.795765
H	2.82037	0.432592	–1.926596
H	1.670624	–1.382116	–3.156366
H	0.639239	–3.272429	–1.910021
C	2.749062	1.134449	1.957207
H	3.263424	1.968853	2.424365
H	1.931977	0.672232	2.506892
C	–3.802190	0.057116	–0.123449
H	–4.173176	–0.185578	0.907651
H	–4.654548	–0.198729	–0.794533
H	–3.693928	1.153436	–0.158374
C	–2.830432	–2.017108	–0.460709
H	–3.628192	–2.336434	–1.169942
H	–3.160416	–2.414978	0.535163
H	–1.925649	–2.586637	–0.735427
N	–2.581997	–0.604445	–0.467957

Na	–0.420756	–0.198575	–0.392070
O	–0.379385	0.19434	1.947757
O	–0.197299	1.881328	–1.246320
C	–1.239473	–0.798187	2.495067
H	–1.414974	–0.594567	3.560392
H	–0.730069	–1.760302	2.389128
H	–2.182051	–0.826330	1.936198
C	–0.993820	1.470331	1.966638
H	–0.268500	2.182679	1.563346
H	–1.253477	1.757079	2.995284
H	–1.901048	1.46328	1.345875
C	0.679786	2.973871	–1.104706
H	0.882588	3.437986	–2.079935
H	1.609698	2.597695	–0.672051
H	0.251313	3.732271	–0.433396
C	–1.475059	2.26098	–1.743933
H	–1.939430	2.991133	–1.066003
H	–2.082495	1.350592	–1.782841
H	–1.372299	2.708183	–2.741701
C	3.089518	0.717003	0.734107
H	3.873785	1.239189	0.185455

Table S639 Coordinates of QM81.

Element	X	Y	Z
C	3.159476	0.101396	1.073113
C	2.127575	0.898499	0.585274
C	1.920781	1.058588	–0.802449
C	2.775947	0.342861	–1.665405
C	3.796918	–0.463367	–1.174702
C	4.002107	–0.587753	0.201142
H	3.304236	0.016899	2.147691
H	1.476098	1.412021	1.289904
H	2.636097	0.442462	–2.740081
H	4.442934	–0.993309	–1.869949
H	4.804621	–1.210540	0.585004
C	–0.010592	2.633016	–0.595906
H	0.215225	2.888738	0.434807
H	–0.774985	3.240686	–1.070645
N	–1.849197	1.528689	0.481204
C	–1.928411	2.325146	1.669483
H	–1.865478	3.420513	1.465984
H	–2.875992	2.185743	2.23287
H	–1.105253	2.084156	2.35991
C	–2.930578	1.882267	–0.397655
H	–3.933330	1.642378	0.019481
H	–2.966700	2.96988	–0.637299
H	–2.846777	1.346389	–1.356464
C	0.837836	1.878681	–1.348625
H	0.674371	1.80389	–2.423145
O	–1.989942	–1.427813	–1.376585
O	–0.249861	–1.492378	1.425758
C	–0.916893	–1.044206	2.593143
H	–0.202868	–0.572204	3.282723
H	–1.655669	–0.305564	2.270603

H	–1.406048	–1.886911	3.100736
C	0.784161	–2.411490	1.70384
H	0.372875	–3.337136	2.12966
H	1.293887	–2.631483	0.763179
H	1.508968	–1.979753	2.406467
C	–3.084917	–1.563051	–0.484271
H	–3.022521	–0.731029	0.223871
H	–4.032558	–1.507940	–1.036274
H	–3.024530	–2.521923	0.04889
C	–1.967719	–2.433627	–2.364083
H	–1.868186	–3.427261	–1.905719
H	–2.886364	–2.405945	–2.965041
H	–1.108554	–2.243458	–3.010257
Na	–0.516804	–0.067305	–0.315101

Table S90 Coordinates of QM82.

Element	X	Y	Z
C	2.705014	–0.060478	1.906319
C	1.910908	0.742595	1.086678
C	2.013023	0.709498	–0.350127
C	2.999231	–0.211791	–0.851010
C	3.768911	–1.000965	–0.020170
C	3.636072	–0.952484	1.380153
H	2.591105	0.024999	2.986156
H	1.22054	1.444868	1.55656
H	3.152537	–0.256308	–1.928891
H	4.504383	–1.669385	–0.464726
H	4.248429	–1.574820	2.024102
C	0.29084	2.529177	–0.684697
H	0.743737	3.041044	0.179282
H	0.113132	3.304408	–1.452543
N	–1.058229	2.061595	–0.194329
C	–1.695734	3.099714	0.60206
H	–1.812369	4.040929	0.034181
H	–2.692325	2.775228	0.922774
H	–1.091436	3.309599	1.491013
C	–1.905933	1.72492	–1.331811
H	–2.839818	1.261949	–0.988740
H	–2.157645	2.617743	–1.934105
H	–1.370687	1.021404	–1.981004
C	1.17397	1.448792	–1.206514
H	1.385305	1.426592	–2.272734
O	–0.886252	–1.743799	–1.376275
O	–1.973717	–1.066189	1.400119
C	–2.539119	–0.259120	2.415061
H	–2.081373	–0.482531	3.387728
H	–2.338059	0.780517	2.1473
H	–3.623136	–0.422624	2.478332
C	–2.128098	–2.450548	1.648212
H	–3.193088	–2.717116	1.686385
H	–1.643197	–2.980738	0.826065
H	–1.651729	–2.729048	2.597299
C	–2.204596	–1.892739	–1.858976
H	–2.881145	–1.717046	–1.019091

H	–2.413707	–1.161352	–2.651843
H	–2.357913	–2.905334	–2.256416
C	0.087841	–1.861042	–2.403728
H	0.025297	–2.852921	–2.870528
H	–0.062524	–1.082324	–3.162570
H	1.068559	–1.725247	–1.941957
Na	–0.293590	–0.192447	0.162621

Table S91 Coordinates of QM83.

Element	X	Y	Z
C	–3.796428	–0.114678	–0.326111
C	–3.243775	0.437173	0.827775
C	–2.135956	1.291729	0.760913
C	–1.623814	1.622272	–0.504515
C	–2.168005	1.061748	–1.657088
C	–3.251407	0.184587	–1.573544
H	–4.655510	–0.776950	–0.251708
H	–3.668839	0.195057	1.800438
H	–0.794613	2.319363	–0.581683
H	–1.757623	1.334799	–2.626314
H	–3.681126	–0.242293	–2.475816
C	1.54229	3.013542	–1.156781
H	1.201348	3.838326	–0.476198
H	2.443478	3.425128	–1.666084
H	0.766774	2.915231	–1.934841
C	2.90443	1.930962	0.381556
H	3.816638	2.318044	–0.130908
H	2.73098	2.64924	1.225771
H	3.191428	0.971466	0.843208
N	1.783945	1.765877	–0.497864
O	1.492802	–1.129976	1.683133
C	1.95972	–0.484809	2.851574
H	1.499488	–0.932188	3.744028
H	3.052521	–0.563163	2.926945
H	1.676554	0.566419	2.774881
C	1.836076	–2.496381	1.635862
H	1.394518	–3.038759	2.484759
H	1.440859	–2.897231	0.69852
H	2.927419	–2.623870	1.661825
O	1.554659	–1.205812	–1.934690
C	1.175966	–0.373894	–3.020371
H	0.09596	–0.486845	–3.157698
H	1.420129	0.67127	–2.786879
H	1.688155	–0.690243	–3.940053
C	2.951145	–1.099772	–1.692332
H	3.513015	–1.486710	–2.554298
H	3.211266	–0.051767	–1.496586
H	3.176375	–1.710377	–0.814212
O	–0.905627	–1.831089	0.043712
C	–1.432200	–2.276720	1.273773
H	–1.117392	–1.563997	2.040023
H	–2.528976	–2.316182	1.233252
H	–1.045159	–3.277144	1.519205
C	–1.253918	–2.677479	–1.030529

H	–2.345566	–2.723667	–1.144652
H	–0.803363	–2.262090	–1.932810
H	–0.860112	–3.691061	–0.865694
C	–0.310415	2.356758	2.095376
H	0.05237	2.715215	3.055171
H	0.36597	2.42953	1.238307
C	–1.530451	1.811017	2.002805
H	–2.144174	1.711534	2.899005
Na	0.57958	–0.078959	–0.124662

Table S92 Coordinates of QM84.

Element	X	Y	Z
C	–0.940976	2.454724	1.606194
H	–0.240341	3.322727	1.633019
H	–0.417055	1.605858	2.074894
H	–1.770326	2.728346	2.290658
C	–3.105987	–0.149955	1.974806
H	–3.014622	0.816733	1.472779
H	–3.036884	–0.024848	3.064154
H	–4.069930	–0.620712	1.729995
O	–2.036434	–0.949903	1.499063
C	–2.077409	–2.263106	2.008572
H	–1.963610	–2.262027	3.101783
H	–1.256052	–2.816310	1.549308
H	–3.031710	–2.748273	1.753401
C	0.368784	2.420742	–1.043891
H	0.68798	3.113285	–0.264966
H	–0.318532	2.836679	–1.774963
C	1.167516	1.367355	–1.417899
H	0.989874	0.882232	–2.378301
N	–1.396808	2.125096	0.279808
C	–2.201596	3.212784	–0.199912
H	–1.642679	4.175764	–0.256026
H	–3.078870	3.413407	0.447764
H	–2.584551	3.007501	–1.209109
C	2.281145	0.856604	–0.629531
C	2.453775	1.170735	0.736083
C	3.255475	0.021841	–1.218973
C	3.547497	0.697063	1.45644
H	1.724533	1.805756	1.233367
C	4.344895	–0.452201	–0.497549
H	3.160002	–0.224744	–2.275136
C	4.502839	–0.119887	0.851039
H	3.651704	0.968061	2.504351
H	5.083543	–1.078963	–0.991774
H	5.355669	–0.485900	1.41492
C	0.788898	–2.577575	–1.293664
H	1.801598	–2.266646	–1.579313
H	0.073659	–2.245177	–2.049243
H	0.756877	–3.672768	–1.202265
C	–2.795182	–1.914114	–1.358359
H	–2.156655	–2.690971	–0.929727
H	–3.336182	–2.313930	–2.227376
H	–3.517405	–1.584102	–0.598858

O	0.413461	–1.972435	–0.070734
O	–1.955277	–0.848783	–1.745835
C	–2.683973	0.261179	–2.239529
H	–1.955453	1.026582	–2.516348
H	–3.341976	0.667644	–1.459418
H	–3.274882	–0.028165	–3.119148
C	1.340796	–2.235648	0.968706
H	0.992808	–1.703675	1.858206
H	2.339653	–1.871814	0.69962
H	1.381412	–3.314314	1.1792
Na	–0.642448	0.033892	–0.073568

Table S93 Coordinates of QM85.

Element	X	Y	Z
C	3.829076	–0.564170	–0.808497
C	2.790783	0.044642	–1.487152
C	1.887356	0.966532	–0.856710
C	2.125276	1.159409	0.547683
C	3.200166	0.565209	1.204505
C	4.067622	–0.311206	0.553897
H	4.491821	–1.233604	–1.354599
H	2.676792	–0.134160	–2.555732
H	1.482604	1.835867	1.110993
H	3.362641	0.796373	2.257573
H	4.902417	–0.770540	1.073575
C	0.118392	2.754816	–0.943120
H	–0.214637	3.478759	–1.708637
H	0.7711	3.31191	–0.252498
C	–2.224531	2.156152	–1.008867
H	–2.502557	3.082331	–1.546519
H	–3.091927	1.810589	–0.436088
H	–1.966267	1.39673	–1.757226
C	–1.411861	3.405783	0.845301
H	–2.289997	3.109307	1.429927
H	–1.630996	4.376652	0.363284
H	–0.569438	3.547044	1.530373
C	0.843223	1.607056	–1.563075
H	0.857624	1.543104	–2.648935
N	–1.087285	2.370727	–0.124752
O	–0.010596	–1.122527	1.931839
C	0.166611	–0.411241	3.140687
H	1.163324	0.045981	3.175092
H	0.040351	–1.080351	4.003616
H	–0.589358	0.378083	3.179218
C	1.009489	–2.096023	1.746784
H	1.999949	–1.636679	1.835318
H	0.901326	–2.493838	0.736647
H	0.896504	–2.903392	2.483332
O	–2.657110	–0.717127	0.261844
C	–3.534881	–1.000951	–0.804539
H	–2.935323	–1.033899	–1.716646
H	–4.305054	–0.222391	–0.898999
H	–4.026541	–1.972758	–0.650933
C	–3.318964	–0.627533	1.503053

H	–3.874723	–1.551755	1.715354
H	–4.018129	0.221328	1.513625
H	–2.553213	–0.487924	2.267867
Na	–0.437218	0.021031	0.000151
O	–0.520449	–1.884648	–1.289101
C	0.112023	–1.903426	–2.556081
H	0.419017	–0.876739	–2.767422
H	–0.590504	–2.254067	–3.325939
H	0.992816	–2.558394	–2.538790
C	–1.045713	–3.139244	–0.919270
H	–0.252796	–3.900372	–0.886046
H	–1.814778	–3.465118	–1.635354
H	–1.490806	–3.024904	0.071919

Table S94 Coordinates of QM86.

Element	X	Y	Z
C	–0.678616	2.955195	–0.922520
C	–0.110735	2.90986	0.350589
C	1.16733	2.366018	0.548516
C	1.885168	1.910657	–0.568683
C	1.320591	1.962282	–1.840509
C	0.032871	2.472243	–2.022246
H	–1.671631	3.375929	–1.058475
H	–0.672614	3.274157	1.20877
H	2.875962	1.490543	–0.430326
H	1.887916	1.594727	–2.691451
H	–0.406039	2.510239	–3.015891
C	3.527661	–1.203769	–0.604291
H	4.318441	–0.869219	0.117719
H	3.892369	–2.190923	–0.979713
H	3.576436	–0.509973	–1.460661
C	2.197789	–2.200452	1.013379
H	2.493019	–3.235703	0.70642
H	2.883136	–1.957259	1.867114
H	1.186325	–2.293252	1.456548
N	2.211168	–1.244621	–0.051328
O	–1.328159	–0.892123	2.337395
C	–0.512365	–0.974405	3.49065
H	–1.091328	–0.723095	4.390283
H	–0.091989	–1.983043	3.599065
H	0.302987	–0.259052	3.363079
C	–2.381423	–1.831645	2.351311
H	–3.054436	–1.650641	3.201879
H	–2.932176	–1.712795	1.414904
H	–1.988451	–2.855888	2.421016
O	–0.529550	–1.948164	–2.212633
C	0.452097	–1.399050	–3.075300
H	0.054945	–0.453295	–3.456353
H	1.38544	–1.225878	–2.519988
H	0.63982	–2.074668	–3.922289
C	–0.092947	–3.189815	–1.678305
H	0.001896	–3.936070	–2.479932
H	0.869757	–3.056869	–1.164928
H	–0.857984	–3.523716	–0.971699

K	–0.179956	–0.318626	–0.035958
O	–2.812123	0.115965	–0.359383
C	–3.521488	0.996195	0.482575
H	–3.096105	0.894149	1.484035
H	–3.411623	2.036545	0.144038
H	–4.590439	0.739869	0.503971
C	–3.209824	0.20002	–1.712275
H	–3.034659	1.212817	–2.104511
H	–2.606576	–0.522487	–2.267631
H	–4.275454	–0.046336	–1.820906
C	2.504289	1.180307	2.258865
H	2.868644	1.08475	3.278367
H	2.761814	0.401257	1.535159
C	1.703694	2.196645	1.913927
H	1.377701	2.92429	2.657743

Table S95 Coordinates of QM87.

Element	X	Y	Z
C	–3.482326	0.851837	–1.207514
C	–2.300812	1.353103	–0.667126
C	–2.022822	1.244449	0.711731
C	–2.969849	0.564916	1.504094
C	–4.143952	0.053713	0.961409
C	–4.414256	0.196874	–0.401457
H	–3.677675	0.979371	–2.269702
H	–1.593276	1.863828	–1.318668
H	–2.789510	0.476711	2.573612
H	–4.860395	–0.447175	1.60771
H	–5.336349	–0.190328	–0.824784
C	–0.016845	2.736821	0.726125
H	–0.298672	3.225579	–0.201680
H	0.797563	3.199435	1.273508
N	1.927954	2.136492	–0.685433
C	1.918934	3.327639	–1.480191
H	1.724139	4.254219	–0.882952
H	2.88151	3.518205	–2.001998
H	1.139371	3.277601	–2.254954
C	2.970176	2.252022	0.290505
H	3.988907	2.341716	–0.151750
H	2.872443	3.146602	0.954412
H	2.992542	1.370215	0.953411
C	–0.823744	1.821222	1.320878
H	–0.592056	1.484357	2.331187
O	2.985503	–1.366948	–0.517945
O	–0.949983	–1.918442	–1.779742
O	0.580122	–1.357894	1.937717
C	1.763206	–0.906099	2.569173
H	2.09641	–1.627373	3.329022
H	2.527609	–0.807900	1.795752
H	1.599224	0.071594	3.043835
C	–0.465166	–1.552924	2.86535
H	–0.654754	–0.634291	3.438255
H	–1.365820	–1.810672	2.304369

H	–0.216196	–2.364138	3.564121
C	3.423245	–2.632112	–0.082000
H	2.571382	–3.127605	0.390543
H	3.776816	–3.237954	–0.927903
H	4.238843	–2.534053	0.649759
C	4.013686	–0.603487	–1.128137
H	4.410429	–1.131721	–2.006448
H	3.565044	0.351029	–1.421077
H	4.829926	–0.424037	–0.414435
C	–1.786560	–1.896034	–2.916244
H	–1.226703	–1.424372	–3.725882
H	–2.061490	–2.918213	–3.211860
H	–2.699655	–1.320349	–2.715723
C	–1.613718	–2.435988	–0.643136
H	–2.017377	–3.437159	–0.850888
H	–0.871873	–2.514063	0.156996
H	–2.431473	–1.770223	–0.331267
K	0.629743	–0.100196	–0.511447

Table S96 Coordinates of QM88.

Element	X	Y	Z
C	–2.524691	0.322274	–2.209428
C	–1.689470	1.132675	–1.443299
C	–1.971276	1.438165	–0.063957
C	–3.132611	0.765461	0.460261
C	–3.936659	–0.049190	–0.312963
C	–3.655970	–0.291459	–1.670842
H	–2.281107	0.169921	–3.260829
H	–0.832350	1.602938	–1.926988
H	–3.411119	0.961396	1.495438
H	–4.816758	–0.501350	0.141712
H	–4.301191	–0.918791	–2.277181
C	–0.149389	3.188321	0.142254
H	–0.411570	3.463431	–0.891109
H	–0.098873	4.134894	0.715769
N	1.25162	2.650892	0.056976
C	2.075255	3.549733	–0.736022
H	2.090669	4.574203	–0.317707
H	3.109462	3.188454	–0.773262
H	1.686762	3.604528	–1.758245
C	1.815493	2.524005	1.393056
H	2.805821	2.053025	1.345745
H	1.924221	3.506645	1.89062
H	1.15189	1.911789	2.015116
C	–1.173205	2.277313	0.731738
H	–1.547989	2.527319	1.722697
O	2.816129	–1.018452	–0.946679
O	0.007709	–2.635583	–0.706746
O	0.191081	–1.206417	2.489069
C	0.613786	–2.367471	3.165
H	–0.154787	–3.152808	3.115599
H	1.522313	–2.722613	2.674259
H	0.828331	–2.148660	4.220193
C	–1.012089	–0.675313	3.018494

H	–0.882103	–0.412932	4.077432
H	–1.249641	0.223157	2.4416
H	–1.830511	–1.402827	2.921944
C	3.306572	–2.341312	–0.886358
H	2.472284	–2.976974	–0.582238
H	3.678636	–2.662450	–1.869673
H	4.123149	–2.419880	–0.154802
C	3.79729	–0.084211	–1.342505
H	4.176638	–0.317600	–2.346859
H	3.320234	0.899157	–1.353949
H	4.637974	–0.075502	–0.634544
C	–0.061486	–2.569109	–2.118586
H	0.959823	–2.457972	–2.491336
H	–0.502764	–3.488415	–2.528021
H	–0.667788	–1.710381	–2.438806
C	–1.286032	–2.717347	–0.127043
H	–1.821335	–3.599932	–0.503551
H	–1.147171	–2.809554	0.952073
H	–1.881853	–1.820366	–0.352371
K	0.546152	–0.079457	0.086764

Table S97 Coordinates of QM89.

Element	X	Y	Z
C	2.762063	–1.116618	1.705871
C	2.955583	–1.013054	0.329292
C	2.83764	0.221884	–0.324466
C	2.56904	1.36456	0.444186
C	2.378462	1.262385	1.819832
C	2.462397	0.022113	2.455273
H	2.855631	–2.083114	2.195363
H	3.193116	–1.900447	–0.253757
H	2.489481	2.328573	–0.046272
H	2.162103	2.158542	2.395358
H	2.315645	–0.052927	3.529831
C	0.371119	3.875806	–0.511516
H	1.043478	4.217647	–1.342583
H	–0.401817	4.678878	–0.430327
H	0.970291	3.931853	0.41321
C	–1.016427	2.597154	–1.850431
H	–1.842471	3.351874	–1.798436
H	–0.488325	2.828832	–2.813094
H	–1.515381	1.621056	–2.000423
N	–0.174143	2.568337	–0.696273
O	–2.696754	–0.766663	–1.013863
C	–3.520413	–0.501431	–2.126984
H	–3.829687	–1.437334	–2.61514
H	–4.417258	0.056629	–1.822071
H	–2.94756	0.105971	–2.829778
C	–3.356334	–1.560043	–0.048223
H	–3.570809	–2.5607	–0.451845
H	–2.695176	–1.641649	0.817145
H	–4.303657	–1.092831	0.258452
O	–1.957567	1.125393	1.938896
C	–1.051556	2.017867	2.563706

H	–0.344079	1.415454	3.1428
H	–0.515707	2.597059	1.798193
H	–1.587057	2.694735	3.246004
C	–2.900732	1.846749	1.159252
H	–3.547067	2.451846	1.812002
H	–2.372733	2.48768	0.439893
H	–3.509365	1.112803	0.625714
K	–0.274635	0.070247	0.018933
O	–0.610593	–1.958306	1.768932
C	–0.24299	–3.242893	1.325659
H	–0.294969	–3.229341	0.234176
H	0.780657	–3.491707	1.64234
H	–0.933293	–4.00439	1.717226
C	–0.64202	–1.840883	3.173424
H	0.361141	–1.986274	3.600342
H	–1.004252	–0.835955	3.398821
H	–1.328383	–2.581104	3.609155
C	2.287426	1.218067	–2.524933
H	2.385912	1.23161	–3.607423
H	1.605601	1.931765	–2.052084
C	2.936223	0.302555	–1.79477
H	3.549056	–0.459086	–2.279755
O	0.402004	–1.847786	–1.84729
C	–0.108234	–1.247236	–3.020963
H	–0.565454	–0.294021	–2.744185
H	0.699662	–1.047783	–3.737364
H	–0.865454	–1.894875	–3.486075
C	1.204071	–2.965996	–2.140694
H	2.08242	–2.671328	–2.73339
H	1.538648	–3.393898	–1.192841
H	0.635552	–3.724107	–2.69948

Table S98 Coordinates of QM90.

Element	X	Y	Z
C	3.442932	0.752185	–1.437107
C	2.485127	–0.256364	–1.466148
C	2.529221	–1.333601	–0.558379
C	3.562125	–1.324026	0.398609
C	4.516561	–0.311832	0.432266
C	4.467377	0.734978	–0.489361
H	3.385548	1.559508	–2.163138
H	1.698602	–0.213565	–2.216989
H	3.625341	–2.147699	1.107704
H	5.309119	–0.345828	1.175513
C	0.648889	–2.671491	–1.548409
H	0.61019	–2.096978	–2.469914
H	0.02563	–3.558115	–1.521772
N	–1.75771	–1.822543	–1.308088
C	–2.293313	–2.397666	–2.501244
H	–1.902393	–3.425066	–2.715167
H	–3.401899	–2.51632	–2.484798
H	–2.053377	–1.78245	–3.381731
C	–2.089876	–2.670774	–0.20413
H	–3.182518	–2.722544	0.022591

H	–1.781744	–3.737342	–0.340698
H	–1.596893	–2.328953	0.724283
C	1.564743	–2.438565	–0.581173
H	1.615218	–3.123196	0.265354
O	–2.699191	0.870959	0.884293
O	0.711106	2.352654	0.739183
O	–0.386541	–0.841607	2.59482
C	–1.580587	–1.253455	3.228416
H	–1.545548	–1.02412	4.302985
H	–2.398102	–0.708186	2.753425
H	–1.740897	–2.331905	3.093917
C	0.733904	–1.583075	3.026135
H	0.60551	–2.650079	2.795666
H	1.606935	–1.201708	2.488494
H	0.890548	–1.463246	4.107983
C	–3.004488	1.896517	1.795473
H	–2.05765	2.287761	2.175546
H	–3.562145	2.707335	1.302341
H	–3.608887	1.516448	2.633366
C	–3.848332	0.267276	0.315585
H	–4.483523	1.028159	–0.163546
H	–3.481679	–0.451157	–0.426973
H	–4.437189	–0.24447	1.091375
C	1.38624	3.315894	–0.042596
H	0.841654	3.412446	–0.984153
H	1.413811	4.283782	0.479
H	2.414139	2.993032	–0.253285
C	1.466018	1.963339	1.869278
H	1.7294	2.83539	2.485203
H	0.844358	1.281492	2.456224
H	2.389983	1.451609	1.558417
K	–0.356923	–0.047254	–0.019719
C	–1.484282	1.06523	–2.954836
H	–2.418043	0.566897	–2.663032
H	–1.649822	1.707275	–3.832108
H	–0.752973	0.290153	–3.196275
C	–1.857543	2.817871	–1.445017
H	–1.972294	3.597693	–2.212476
H	–2.842861	2.386287	–1.220015
H	–1.446057	3.25294	–0.53022
O	–0.96135	1.819664	–1.876452
H	5.21552	1.522353	–0.470728

Table S99 Coordinates of QM91.

Element	X	Y	Z
C	–3.391402	–0.190455	–1.639075
C	–2.423436	0.743992	–1.280204
C	–2.411048	1.375806	0.014083
C	–3.41063	0.872949	0.922113
C	–4.347978	–0.071213	0.551491
C	–4.367683	–0.625492	–0.742006
H	–3.380139	–0.587604	–2.654133
H	–1.702675	1.063521	–2.034163
H	–3.464191	1.313129	1.917651

H	–5.097773	–0.380019	1.278485
H	–5.119134	–1.352436	–1.032852
C	–0.744483	3.15776	–0.646133
H	–1.258441	3.127461	–1.619239
H	–0.680274	4.226842	–0.359482
N	0.65184	2.690937	–0.922668
C	1.17699	3.343556	–2.110452
H	1.196892	4.445754	–2.007248
H	2.199465	3.001356	–2.307781
H	0.553838	3.094324	–2.975614
C	1.499646	2.997581	0.217944
H	2.51593	2.628563	0.042228
H	1.548665	4.087599	0.409763
H	1.093292	2.516321	1.11615
C	–1.50707	2.387538	0.380679
H	–1.677186	2.877126	1.338449
O	1.844571	–1.583425	–1.752084
O	–0.55335	–2.653337	–0.150484
O	0.385488	–0.522299	2.531395
C	0.99515	–1.523054	3.311281
H	0.286734	–2.331728	3.542873
H	1.828937	–1.927455	2.731835
H	1.375092	–1.107658	4.25647
C	–0.718679	0.086778	3.180622
H	–0.398025	0.551382	4.123832
H	–1.105474	0.849877	2.499694
H	–1.502993	–0.654303	3.389576
C	2.392819	–2.624652	–0.969859
H	2.201025	–2.38006	0.076018
H	1.915724	–3.585122	–1.208488
H	3.476975	–2.705304	–1.13912
C	2.104838	–1.747457	–3.127469
H	1.706	–2.704696	–3.493296
H	1.615267	–0.926676	–3.654651
H	3.186224	–1.718265	–3.327166
C	–1.040427	–3.025447	–1.42401
H	–0.184764	–3.076017	–2.101038
H	–1.52749	–4.010217	–1.376591
H	–1.762359	–2.287302	–1.796426
C	–1.615532	–2.513874	0.781064
H	–2.208997	–3.437775	0.830022
H	–1.160831	–2.323754	1.754507
H	–2.275593	–1.679401	0.50806
K	0.306876	–0.005396	–0.127569
O	2.954872	0.126168	0.741544
C	4.022412	0.183851	–0.179645
H	3.638674	–0.161348	–1.140936
H	4.393723	1.214104	–0.281987
H	4.85423	–0.459075	0.14443
C	3.325353	0.639667	2.004372
H	3.718872	1.661788	1.908554
H	2.426836	0.653161	2.622447
H	4.094725	0.006026	2.469926

4.2.4 Calculated Aminometalation Reaction of Dimethylamide to 4-Methoxystyrene

In the following, the energies (Table S100) and the coordinates of the opitimized structures for the aminometalation of different metal dimethylamides to 4-methoxystyrene (Scheme S12) are listed.

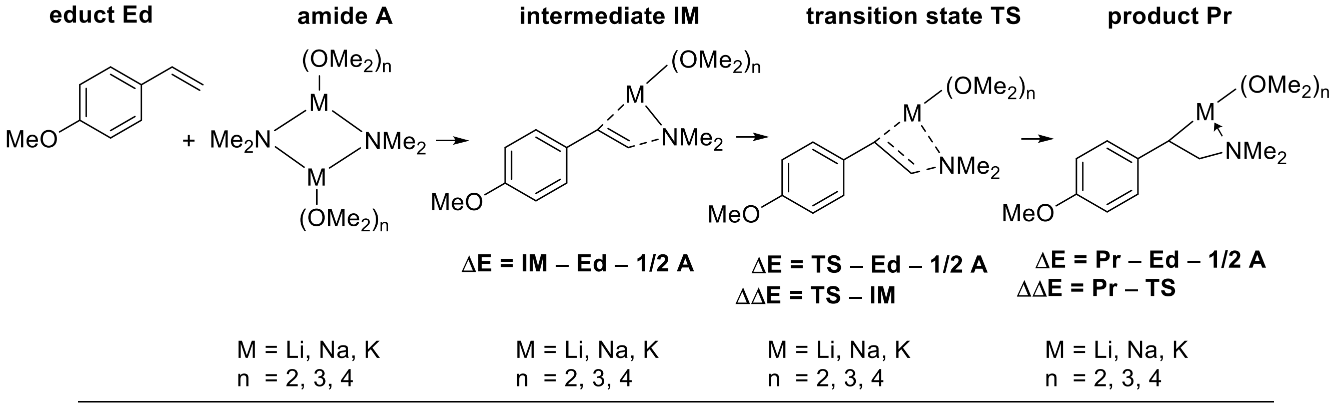

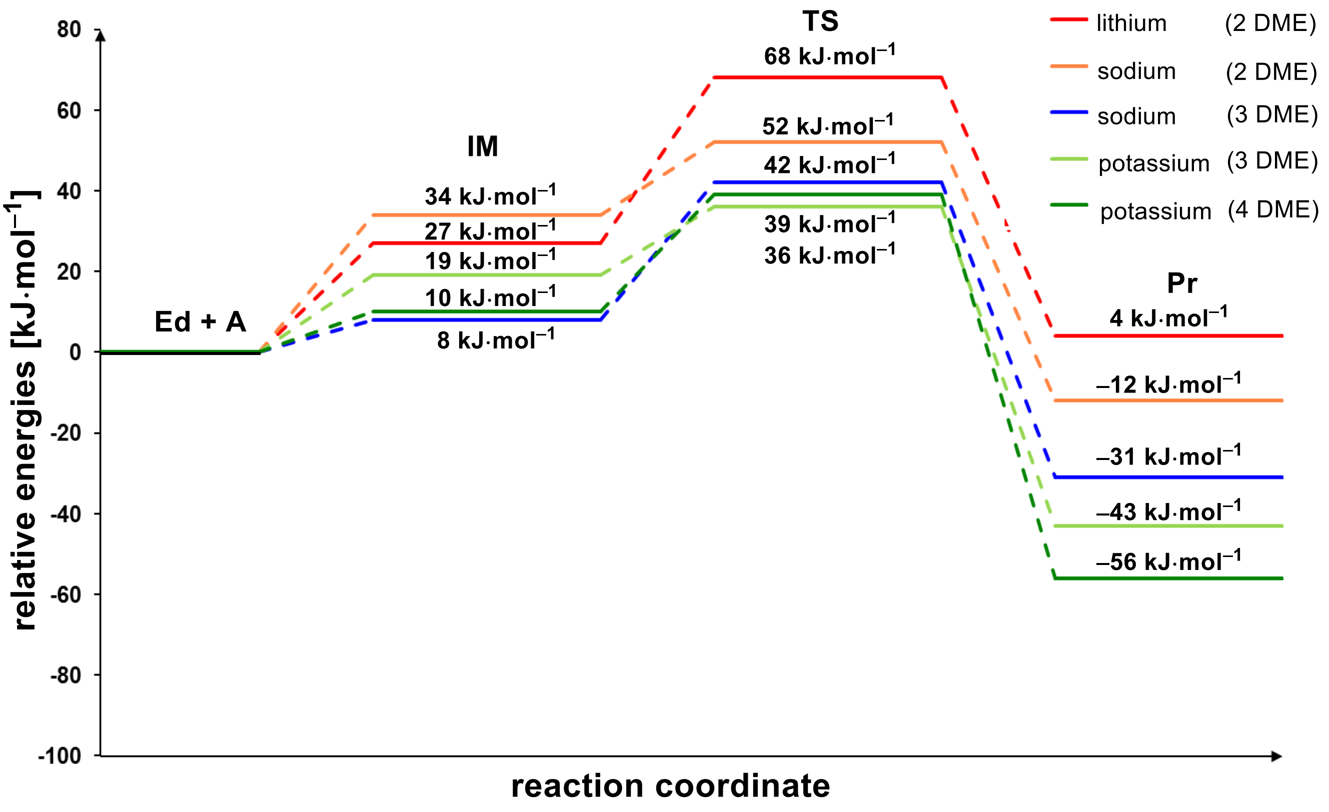

Scheme S12 Calculated reaction scheme for the aminometalation of different metal dimethylamides to 4-methoxystyrene.

Table S100 Total (SCF) and zero-point-corrected (ZPE) energies of the optimized structures of the elimination reaction of a metal amide and 4-methoxystyrene.

Optimized structure		SCF [Hartree]	ZPE [Hartree]
4-methoxystyrene	QM92	–423.991103234	–423.823263
A Li + 2 DME	QM44	–904.007590038	–903.508184
A Na + 2 DME	QM45	–1213.41663972	–1212.924143
A Na + 3 DME	QM46	–1523.34423200	–1522.763223
A K + 3 DME	QM47	–2398.56867462	–2397.912793
A K + 4 DME	QM47b	-2708.49244230	-2707.668902
IM Li +2 DME	QM93	-875.984609506	-875.567102
TS Li + 2 DME	QM94	-875.968906218	-875.551527
Pr Li + 2 DME	QM95	-875.996648132	-875.575696
IM Na +2 DME	QM96	-1030.68823145	-1030.27246
TS Na + 2 DME	QM97	-1030.68138972	-1030.265673
Pr Na + 2 DME	QM98	-1030.70867168	-1030.289845
IM Na + 3 DME	QM99	-1185.66046227	-1185.161927
TS Na + 3 DME	QM100	-1185.64813262	-1185.148858
Pr Na + 3 DME	QM101	-1185.67972557	-1185.176694
IM K + 3 DME	QM102	-1623.26926992	-1622.772262
TS K + 3 DME	QM103	-1623.26177157	-1622.766040
Pr K + 3 DME	QM104	-1623.29639317	-1622.796096
IM K + 4 DME	QM105	-1778.23405018	-1777.654022
TS K + 4 DME	QM106	-1778.22292848	-1777.642733
Pr K + 4 DME	QM107	-1778.25918525	-1777.675196

Table S101 Coordinates of QM92.

Element	X	Y	Z
C	–0.502189	–1.276756	–0.000068
C	0.872626	–1.030901	0.000027
C	1.328767	0.286508	0.000002
C	0.403224	1.340168	–0.000148
C	–0.953777	1.075591	–0.000242
C	–1.440236	–0.243937	–0.000166
H	–0.849243	–2.307767	–0.000045
H	1.562675	–1.866876	0.000124
H	0.780954	2.357955	–0.000209
H	–1.648757	1.910359	–0.000411
C	–2.876400	–0.570906	–0.000178
H	–3.099646	–1.637928	–0.000678
C	–3.899472	0.289277	0.000421
H	–3.760473	1.366871	0.001012
H	–4.924157	–0.067162	0.000349
O	2.638286	0.64962	0.000067
C	3.605084	–0.379016	0.000152
H	4.574528	0.119184	0.000168
H	3.515997	–1.005833	0.896
H	3.51608	–1.005914	–0.895650

Table S102 Coordinates of QM93.

Element	X	Y	Z
C	–0.260189	–1.210866	–1.322568
C	–1.317421	–0.304791	–1.416577
C	–2.475526	–0.440747	–0.642664
C	–2.579286	–1.574768	0.17628
C	–1.537282	–2.482581	0.290368
C	–0.351706	–2.287689	–0.429392
H	0.627025	–1.072630	–1.931387
H	–1.249673	0.513448	–2.129595
H	–3.487772	–1.730629	0.755159
H	–1.608133	–3.347972	0.943024
C	–3.382789	1.868625	–0.882469
H	–4.226295	2.551877	–0.889073
H	–2.393927	2.297695	–1.033435
C	3.405946	0.534651	0.781005
H	4.118664	1.381459	0.646631
H	4.050112	–0.370528	0.88908
H	2.914134	0.691487	1.754182
C	3.130006	0.290783	–1.521964
H	3.792605	–0.607489	–1.576028
H	3.79985	1.150104	–1.755733
H	2.424685	0.210521	–2.365696
N	2.438686	0.42723	–0.274535
O	0.080154	2.528601	–0.158136
O	–0.068663	0.16002	1.737756
C	0.544239	2.953588	–1.432617
H	0.417412	4.039249	–1.536431
H	–0.061401	2.449834	–2.189970
H	1.598514	2.675649	–1.556539
C	0.832178	3.152609	0.876115
H	0.454538	2.775041	1.828256
H	0.696726	4.241079	0.831532
H	1.893497	2.893176	0.771406
C	–1.214851	0.684323	2.375721
H	–1.942404	–0.113796	2.572307
H	–1.658448	1.416918	1.696384
H	–0.938056	1.16611	3.324293
C	0.569002	–0.874951	2.467058
H	0.942241	–0.490325	3.425381
H	1.404771	–1.224598	1.855937
H	–0.134495	–1.698399	2.643963
C	–3.556422	0.560784	–0.664689
H	–4.558384	0.185444	–0.454726
O	0.635771	–3.182382	–0.210897
C	1.908297	–2.916158	–0.799798
H	2.578745	–3.677465	–0.401393
H	1.854974	–3.017710	–1.890856
H	2.248227	–1.907440	–0.526634
Li	0.636063	0.62083	0.049694

Table S103 Coordinates of QM94.

Element	X	Y	Z
C	2.661718	1.219137	0.207052

C	1.344528	1.353393	–0.222666
C	0.843789	0.612067	–1.313354
C	1.7362	–0.284024	–1.936740
C	3.04942	–0.431133	–1.505169
C	3.517615	0.322478	–0.428974
H	3.038861	1.810246	1.03866
H	0.690107	2.047116	0.300315
H	1.386435	–0.866138	–2.787262
H	3.72996	–1.116951	–2.003557
C	–1.412248	1.713873	–1.358214
H	–1.041322	2.620413	–0.883306
H	–2.358817	1.828047	–1.880915
N	–2.493856	1.357089	0.507898
C	–2.177108	2.507479	1.308773
H	–2.328842	3.467047	0.766401
H	–2.800800	2.571329	2.220341
H	–1.127695	2.484246	1.633411
C	–3.868235	1.430399	0.087846
H	–4.576664	1.368498	0.935879
H	–4.107248	2.375464	–0.447469
H	–4.105955	0.605513	–0.599499
C	–0.532911	0.738994	–1.781991
H	–0.857710	0.022745	–2.535267
O	–2.574100	–1.699880	–0.396392
O	–0.395653	–0.978985	1.38337
C	–0.117991	–0.264537	2.573232
H	0.793507	0.336751	2.454253
H	–0.974177	0.387821	2.757426
H	0.011048	–0.962656	3.411293
C	0.651253	–1.856300	1.005945
H	0.799241	–2.619915	1.78121
H	0.347884	–2.333227	0.071583
H	1.582698	–1.297865	0.844987
C	–3.410776	–1.996673	0.709782
H	–2.870556	–1.697362	1.609034
H	–4.350975	–1.434472	0.642477
H	–3.624890	–3.073030	0.741404
C	–3.171092	–2.033411	–1.632888
H	–3.383717	–3.109721	–1.674553
H	–4.105442	–1.474643	–1.778160
H	–2.462827	–1.770286	–2.419852
Li	–1.481588	–0.143843	0.034196
O	4.826689	0.192843	–0.010872
C	4.988587	–0.756385	1.027775
H	4.671386	–1.754887	0.697521
H	6.050393	–0.774707	1.280117
H	4.405895	–0.471440	1.914348

Table S104 Coordinates of QM95.

Element	X	Y	Z
C	–2.182125	–0.803536	0.681258
C	–0.942453	–1.224144	0.191778
C	–0.520297	–0.954305	–1.154787
C	–1.458259	–0.184699	–1.928954

C	–2.667380	0.245537	–1.421946
C	–3.047587	–0.058150	–0.103980
H	–2.481492	–1.050481	1.699369
H	–0.305958	–1.818937	0.846848
H	–1.207284	0.038112	–2.965154
H	–3.346009	0.828509	–2.041815
C	1.649899	–2.193978	–0.850348
H	1.095646	–2.972510	–0.301386
H	2.386443	–2.716706	–1.483197
N	2.403046	–1.419340	0.20719
C	2.823797	–2.282045	1.302509
H	3.480519	–3.098281	0.953425
H	3.372622	–1.699358	2.050732
H	1.945524	–2.729395	1.780295
C	3.569107	–0.776558	–0.392984
H	4.040531	–0.097682	0.327224
H	4.317644	–1.521164	–0.718574
H	3.248703	–0.201898	–1.268385
Li	1.02767	0.129311	0.055354
C	0.747026	–1.324118	–1.652979
H	0.96129	–1.169576	–2.706559
O	1.629935	1.818582	–0.740044
O	0.703648	1.059847	1.778715
C	0.576236	0.234246	2.92092
H	0.694787	0.83037	3.835446
H	–0.403633	–0.260094	2.931149
H	1.365018	–0.518628	2.866025
C	–0.330286	2.034314	1.70887
H	–0.296866	2.679856	2.596225
H	–0.150921	2.625979	0.80967
H	–1.310059	1.546984	1.634079
C	2.594553	2.654124	–0.132754
H	2.609123	2.408547	0.930463
H	3.586204	2.479003	–0.572605
H	2.324837	3.710575	–0.266781
C	1.516296	2.034981	–2.135925
H	1.241578	3.079602	–2.332110
H	2.466915	1.806579	–2.637128
H	0.73927	1.36425	–2.501490
O	–4.247654	0.409822	0.410762
C	–5.340368	–0.451318	0.147448
H	–6.227167	0.012871	0.585535
H	–5.489285	–0.579053	–0.932969
H	–5.178707	–1.438148	0.601057

Table S105 Coordinates of QM96.

Element	X	Y	Z
C	–2.135390	–0.918166	0.802763
C	–1.030796	–1.704626	1.131661
C	–0.284809	–2.372088	0.153674
C	–0.702705	–2.246017	–1.183098
C	–1.797021	–1.465228	–1.530316
C	–2.528888	–0.798168	–0.536313
H	–2.682724	–0.413287	1.590885

H	–0.739693	–1.794429	2.174512
H	–0.144691	–2.753766	–1.966975
H	–2.111183	–1.356896	–2.563540
C	1.717172	–2.929327	1.532874
H	2.582218	–3.557819	1.72227
H	1.540208	–2.096238	2.209703
C	0.827815	3.724002	–0.065426
H	0.693896	4.072948	0.993025
H	0.536185	4.600797	–0.687692
H	1.913001	3.588207	–0.205695
C	–1.295485	2.813151	–0.272953
H	–1.630069	3.681814	–0.885196
H	–1.635469	3.055017	0.772243
H	–1.900867	1.954209	–0.609541
N	0.105015	2.533059	–0.385726
Na	0.326109	0.338654	–0.325738
O	1.135553	0.288477	1.915873
O	2.159439	–0.057234	–1.577703
C	0.333164	1.223477	2.625986
H	0.780665	1.430915	3.607805
H	–0.647889	0.762293	2.768602
H	0.222418	2.144708	2.041193
C	2.427894	0.812434	1.664292
H	2.988627	0.043707	1.12452
H	2.940231	1.041746	2.609122
H	2.355275	1.723534	1.054004
C	3.104663	–1.091776	–1.720192
H	3.390118	–1.213293	–2.774272
H	2.645915	–2.011463	–1.351434
H	4.007728	–0.878175	–1.129805
C	2.655022	1.206914	–2.004112
H	3.53961	1.48258	–1.411889
H	1.848484	1.930491	–1.841801
H	2.931969	1.162162	–3.065922
C	0.917085	–3.160437	0.486864
H	1.156484	–3.970983	–0.202557
O	–3.589722	–0.078391	–0.960625
C	–4.352556	0.619545	0.009702
H	–5.137550	1.132716	–0.544273
H	–4.802618	–0.078876	0.725194
H	–3.735737	1.35583	0.537923

Table S106 Coordinates of QM97.

Element	X	Y	Z
C	–2.550310	–0.678780	0.680258
C	–1.353351	–1.303394	0.317844
C	–0.978663	–1.481209	–1.026457
C	–1.854834	–0.944538	–1.997355
C	–3.034522	–0.307570	–1.650850
C	–3.398032	–0.175126	–0.304953
H	–2.798143	–0.589503	1.73348
H	–0.700148	–1.665676	1.109369
H	–1.601397	–1.055231	–3.049993
H	–3.704159	0.089714	–2.408243

C	1.110659	–2.746892	–0.524008
H	0.747484	–3.080286	0.444749
H	1.999019	–3.258593	–0.884186
N	2.437762	–1.400941	0.741541
C	2.334153	–2.012906	2.034912
H	2.476382	–3.117741	2.004463
H	3.085633	–1.635432	2.758408
H	1.342373	–1.833385	2.477578
C	3.739276	–1.677835	0.192404
H	4.567744	–1.234192	0.784409
H	3.967938	–2.765662	0.133818
H	3.825785	–1.276029	–0.829431
C	0.269089	–2.137099	–1.416291
H	0.558889	–2.057169	–2.463572
O	2.705036	1.676207	–1.087123
O	0.297734	1.663601	0.767427
C	0.487537	1.294247	2.122849
H	–0.399696	0.768805	2.50341
H	1.351342	0.622791	2.15547
H	0.674831	2.184574	2.73915
C	–0.849723	2.465599	0.576329
H	–0.738525	3.425234	1.100349
H	–0.953342	2.642743	–0.496351
H	–1.748135	1.949089	0.937665
C	3.444517	1.913061	0.10059
H	3.610381	0.94255	0.575056
H	4.402774	2.392735	–0.138561
H	2.869152	2.558131	0.77978
C	2.304412	2.87803	–1.706729
H	1.663835	3.464346	–1.032320
H	3.178801	3.48056	–1.986936
H	1.74619	2.616562	–2.608273
Na	1.234697	0.019231	–0.490885
O	–4.578162	0.471667	–0.061283
C	–5.011418	0.548028	1.277298
H	–4.314815	1.134188	1.892018
H	–5.979286	1.049604	1.254555
H	–5.126508	–0.451167	1.715874

Table S107 Coordinates of QM98.

Element	X	Y	Z
C	–2.519897	0.276316	–1.131364
C	–1.419499	1.00915	–0.679410
C	–1.151981	1.211364	0.719591
C	–2.109353	0.595708	1.599503
C	–3.187716	–0.132885	1.136867
C	–3.405069	–0.310133	–0.238336
H	–2.691607	0.151453	–2.199246
H	–0.764838	1.468304	–1.421191
H	–1.980130	0.734783	2.672202
H	–3.888087	–0.581779	1.839054
C	0.856731	2.682133	0.29379
H	0.273877	3.14563	–0.518885
H	1.352693	3.50745	0.83673

N	1.935806	1.892973	–0.409418
C	2.484973	2.660887	–1.516962
H	2.887598	3.634651	–1.182885
H	3.300735	2.108537	–1.997235
H	1.703923	2.85	–2.260858
C	2.996754	1.560068	0.53372
H	3.715606	0.870272	0.07332
H	3.542037	2.46076	0.872463
H	2.552554	1.083075	1.415861
C	–0.008186	1.882318	1.204532
H	0.055492	2.072724	2.272825
O	1.502399	–1.562192	1.561374
O	1.971972	–1.600974	–1.430730
C	2.454647	–1.115146	–2.668694
H	1.784193	–1.406354	–3.487901
H	2.48981	–0.026078	–2.593946
H	3.461336	–1.505487	–2.868761
C	1.848215	–3.010412	–1.403815
H	2.82652	–3.483909	–1.563460
H	1.463348	–3.280936	–0.418512
H	1.15019	–3.352812	–2.178709
C	2.85707	–1.895472	1.777284
H	3.328229	–1.995998	0.796661
H	3.366572	–1.105368	2.346047
H	2.937815	–2.842687	2.328084
C	0.797665	–1.334500	2.773162
H	0.816706	–2.238387	3.39619
H	1.243724	–0.494283	3.320858
H	–0.232647	–1.083990	2.510835
Na	0.786291	–0.200085	–0.107996
O	–4.472328	–1.073230	–0.691170
C	–5.659200	–0.320479	–0.856651
H	–6.430605	–1.010784	–1.207004
H	–5.975545	0.131001	0.093286
H	–5.516613	0.477994	–1.597035

Table S108 Coordinates of QM99.

Element	X	Y	Z
C	–0.349098	2.839816	0.040276
H	0.64777	3.137785	–0.376201
H	–0.142868	2.310488	0.987904
H	–0.832176	3.808239	0.317628
C	–2.814963	1.755166	1.94891
H	–2.651959	2.216862	0.969726
H	–2.349496	2.358848	2.740276
H	–3.889647	1.6536	2.163521
O	–2.207603	0.472834	1.887639
C	–2.383286	–0.269150	3.07071
H	–1.914234	0.23989	3.925164
H	–1.912538	–1.243051	2.918215
H	–3.453224	–0.408546	3.288092
C	–0.345244	–0.764508	–2.838439
H	–0.420391	0.319232	–2.758637
H	–1.113422	–1.279145	–3.408763

C	0.649426	–1.458093	–2.267811
H	0.674671	–2.540606	–2.405736
N	–1.133758	2.031653	–0.846182
C	–1.323987	2.762832	–2.062185
H	–0.371029	2.986682	–2.606795
H	–1.806224	3.757101	–1.914716
H	–1.962192	2.209565	–2.768607
C	1.755267	–0.906837	–1.464320
C	1.773398	0.423724	–1.025805
C	2.830415	–1.735759	–1.103305
C	2.820721	0.909089	–0.243505
H	0.936312	1.086304	–1.258293
C	3.883928	–1.262683	–0.335379
H	2.843389	–2.770654	–1.441726
C	3.882074	0.066844	0.103159
H	2.789126	1.942601	0.083557
H	4.720494	–1.898856	–0.063098
C	–0.107970	–3.088949	0.715833
H	0.875225	–3.225173	0.246432
H	–0.892826	–3.372522	0.011316
H	–0.169676	–3.721929	1.612334
C	–4.254609	–1.194301	0.208194
H	–3.776994	–1.924556	0.865999
H	–5.181210	–1.619827	–0.201691
H	–4.493927	–0.291556	0.786964
O	–0.314140	–1.733978	1.05329
O	–3.344767	–0.900420	–0.828401
C	–3.835687	0.103298	–1.702761
H	–3.096783	0.230368	–2.495748
H	–3.944091	1.058944	–1.173475
H	–4.796235	–0.207323	–2.136229
C	0.696391	–1.226284	1.908303
H	0.461449	–0.175108	2.0967
H	1.681709	–1.303441	1.431179
H	0.702701	–1.777425	2.85986
Na	–1.295353	–0.081507	–0.166105
O	4.947454	0.444634	0.85847
C	5.002439	1.789067	1.290702
H	4.148434	2.034231	1.933641
H	5.92688	1.882431	1.860578
H	5.025332	2.47736	0.437617

Table S109 Coordinates of QM100.

Element	X	Y	Z
C	–1.515050	2.155162	1.929696
H	–0.941719	3.109312	2.001169
H	–0.822285	1.343334	2.205479
H	–2.273146	2.208633	2.737899
C	–3.280430	–0.739899	2.180553
H	–3.379915	0.275826	1.788907
H	–3.103526	–0.717947	3.264676
H	–4.193642	–1.319939	1.979835
O	–2.174984	–1.321213	1.509085
C	–1.982943	–2.671919	1.861937

H	–1.743027	–2.766647	2.930427
H	–1.156324	–3.052061	1.258745
H	–2.888716	–3.259943	1.650342
C	–0.591165	2.629592	–0.826437
H	–0.249169	3.263747	–0.008195
H	–1.414311	3.03599	–1.407624
C	0.268741	1.749205	–1.438774
H	0.01709	1.363489	–2.427225
N	–2.097380	1.929871	0.631273
C	–3.101704	2.931671	0.408787
H	–2.693150	3.968438	0.427347
H	–3.902394	2.910407	1.175384
H	–3.585059	2.796383	–0.568649
C	1.537994	1.307288	–0.874204
C	1.867567	1.478035	0.48752
C	2.516785	0.702806	–1.692905
C	3.104482	1.085285	0.993617
H	1.146136	1.938924	1.157954
C	3.750976	0.305168	–1.192591
H	2.306983	0.580952	–2.754127
C	4.048498	0.494079	0.158102
H	3.350798	1.237571	2.041579
H	4.502593	–0.134368	–1.844242
C	0.42618	–2.207603	–1.778030
H	1.316115	–1.685092	–2.151118
H	–0.442731	–1.925853	–2.376153
H	0.588937	–3.293092	–1.839040
C	–3.195451	–2.071112	–1.318094
H	–2.410432	–2.786155	–1.058012
H	–3.774784	–2.447803	–2.172622
H	–3.862713	–1.942551	–0.454463
O	0.152866	–1.826335	–0.442259
O	–2.561938	–0.854794	–1.647945
C	–3.486838	0.186069	–1.908575
H	–2.906404	1.077367	–2.157044
H	–4.089280	0.397616	–1.014767
H	–4.139611	–0.082802	–2.750096
C	1.259619	–2.041443	0.415831
H	0.977757	–1.678774	1.408058
H	2.137482	–1.484916	0.064941
H	1.497313	–3.114012	0.470942
Na	–1.137744	0.021156	–0.078445
O	5.278076	0.117651	0.661305
C	5.326721	–1.242211	1.051878
H	5.129164	–1.904558	0.197943
H	6.332422	–1.429019	1.433195
H	4.59034	–1.447781	1.841188

Table S110 Coordinates of QM101.

Element	X	Y	Z
C	–3.327501	0.194021	1.13493
C	–2.190438	0.81072	1.626283
C	–1.206707	1.417789	0.772727
C	–1.473137	1.273672	–0.632082

C	–2.630289	0.664288	–1.113012
C	–3.566122	0.117175	–0.243947
H	–4.055272	–0.240528	1.818178
H	–2.051680	0.88612	2.70395
H	–0.770956	1.686127	–1.356318
H	–2.801740	0.588145	–2.185894
C	0.753955	2.949409	0.394958
H	1.184456	3.796774	0.960893
H	0.137228	3.388945	–0.405795
C	2.979499	2.034657	0.618788
H	3.3729	2.986042	1.024142
H	3.799549	1.503569	0.124324
H	2.619792	1.431172	1.461035
C	2.358732	3.08643	–1.423060
H	3.198274	2.597997	–1.929853
H	2.69832	4.082273	–1.082231
H	1.551231	3.230785	–2.148270
C	–0.058661	2.06893	1.28113
H	–0.031480	2.269067	2.350077
N	1.888009	2.263366	–0.318519
O	0.072064	–1.872035	–1.064903
C	0.522063	–2.695232	–2.113090
H	–0.142452	–2.620825	–2.984995
H	0.564972	–3.746341	–1.790125
H	1.526051	–2.362860	–2.385103
C	–1.210275	–2.250549	–0.589333
H	–1.971405	–2.121994	–1.367976
H	–1.457321	–1.598332	0.253395
H	–1.194616	–3.300680	–0.262734
O	2.9514	–0.839104	–0.685970
C	3.98646	–1.337784	0.134585
H	3.560037	–1.501884	1.125683
H	4.807802	–0.611304	0.209175
H	4.379845	–2.281772	–0.268309
C	3.413402	–0.427011	–1.953292
H	3.828857	–1.276794	–2.514408
H	4.188367	0.346192	–1.853618
H	2.560989	–0.008051	–2.494494
Na	0.889791	0.009216	–0.012680
O	1.093021	–1.444609	1.74627
C	0.473623	–1.093518	2.972126
H	0.376285	–0.005980	2.972599
H	1.089713	–1.424102	3.819848
H	–0.522438	–1.551182	3.041114
C	1.246071	–2.838537	1.599697
H	0.274778	–3.346335	1.677386
H	1.918298	–3.237423	2.373667
H	1.668802	–3.018591	0.60866
O	–4.684254	–0.548120	–0.731205
C	–5.810490	0.296388	–0.879536
H	–6.628965	–0.322158	–1.256386
H	–6.098798	0.739449	0.083101
H	–5.602151	1.105392	–1.592371

Table S111 Coordinates of QM102.

Element	X	Y	Z
C	1.593434	–1.685028	–1.689957
C	0.536398	–1.098550	–2.374528
C	0.394272	0.297125	–2.440172
C	1.380287	1.086371	–1.835888
C	2.452314	0.512293	–1.155161
C	2.551413	–0.878348	–1.064042
H	1.709224	–2.763752	–1.635669
H	–0.208083	–1.734045	–2.851449
H	1.298487	2.167496	–1.871138
H	3.186227	1.16238	–0.691252
C	1.004467	3.624798	0.421845
H	0.875194	4.418624	–0.357810
H	1.148788	4.19058	1.376868
H	1.970174	3.134518	0.207581
C	–1.271863	3.376297	0.756009
H	–1.267291	3.91938	1.735685
H	–1.539743	4.161222	0.003881
H	–2.138973	2.688402	0.794864
N	–0.058919	2.672905	0.466139
O	–3.282519	–0.697866	–0.037754
C	–4.154412	0.091308	–0.822279
H	–4.797648	–0.545014	–1.446049
H	–4.785354	0.725605	–0.185001
H	–3.534329	0.722568	–1.462318
C	–3.977336	–1.547480	0.850257
H	–4.611835	–2.253430	0.294625
H	–3.226935	–2.096649	1.422348
H	–4.610475	–0.963019	1.532242
O	0.483987	0.132313	2.820914
C	1.781683	0.386597	2.317515
H	2.087637	–0.499172	1.748662
H	1.763619	1.268983	1.661083
H	2.492806	0.545345	3.142214
C	–0.010345	1.256577	3.536489
H	0.605995	1.438076	4.428751
H	–0.014270	2.13859	2.881585
H	–1.028833	1.014746	3.850376
K	–0.751151	0.196176	0.356753
O	–0.843826	–2.475807	0.804381
C	–1.263921	–3.385701	–0.186144
H	–2.039228	–2.887618	–0.773569
H	–0.428898	–3.664388	–0.843779
H	–1.674211	–4.297934	0.271581
C	0.23462	–2.968591	1.576776
H	1.12965	–3.100895	0.951054
H	0.431765	–2.229426	2.356247
H	–0.028091	–3.930962	2.038618
C	–1.314393	2.083843	–2.652427
H	–2.207735	2.484797	–3.124230
H	–0.862480	2.648193	–1.832184
C	–0.810459	0.903506	–3.038476
H	–1.315764	0.317029	–3.807137

O	3.535545	–1.534252	–0.388129
C	4.615698	–0.765398	0.10563
H	5.311439	–1.476782	0.550272
H	5.112024	–0.221678	–0.706779
H	4.284821	–0.052277	0.870566

Table S112 Coordinates of QM103.

Element	X	Y	Z
C	–2.881173	1.079549	–1.362358
C	–1.732324	1.749808	–0.977368
C	–1.378219	1.895877	0.382584
C	–2.250080	1.324786	1.319687
C	–3.412459	0.644611	0.948134
C	–3.730024	0.515828	–0.402442
H	–3.148112	0.979315	–2.411003
H	–1.099012	2.183666	–1.747734
H	–2.028156	1.435834	2.379636
H	–4.055004	0.232825	1.71946
C	0.763198	3.155253	0.013457
H	0.614307	3.281348	–1.054508
H	1.610085	3.689244	0.431942
N	2.683171	1.776512	–0.800742
C	3.319771	2.731404	–1.652937
H	3.349245	3.761835	–1.216615
H	4.383713	2.490992	–1.883291
H	2.796063	2.805951	–2.616665
C	3.377219	1.726018	0.447622
H	4.397057	1.271669	0.392948
H	3.537872	2.727817	0.916799
H	2.810201	1.131455	1.187965
C	–0.160414	2.585606	0.822474
H	0.013096	2.589788	1.898478
O	2.806351	–1.872148	–1.052968
O	–1.158577	–1.851795	–0.512233
O	1.487043	–0.993146	2.048086
C	2.807131	–1.427706	2.309614
H	2.817977	–2.139681	3.14689
H	3.169904	–1.910524	1.40056
H	3.457753	–0.575840	2.548462
C	0.952987	–0.236401	3.113331
H	1.537911	0.680525	3.272382
H	–0.070477	0.033379	2.839197
H	0.939392	–0.824813	4.041894
C	2.921933	–3.273867	–1.055072
H	1.963355	–3.685602	–0.729958
H	3.157509	–3.647663	–2.061632
H	3.712852	–3.605666	–0.364999
C	4.012706	–1.220925	–1.427132
H	4.329541	–1.553225	–2.425675
H	3.796857	–0.145272	–1.428440
H	4.80854	–1.453443	–0.704030
C	–2.109356	–2.252669	–1.475459
H	–1.860657	–1.748353	–2.411357
H	–2.069846	–3.341451	–1.624952

H	–3.124689	–1.960825	–1.175215
C	–1.420824	–2.405470	0.75871
H	–1.427722	–3.504469	0.709935
H	–0.620347	–2.080579	1.428333
H	–2.389168	–2.053394	1.14152
K	0.893497	–0.061386	–0.448980
O	–4.827941	–0.141118	–0.886367
C	–5.699292	–0.726777	0.054751
H	–6.119904	0.027059	0.731572
H	–6.502819	–1.187604	–0.520189
H	–5.185662	–1.497431	0.646373

Table S113 Coordinates of QM104.

Element	X	Y	Z
C	–1.577261	–1.683153	–0.911394
C	–0.328551	–1.352300	–1.430486
C	–0.080614	–0.106197	–2.109962
C	–1.241561	0.736162	–2.238743
C	–2.472644	0.396837	–1.709439
C	–2.656562	–0.812449	–1.025053
H	–1.720172	–2.633437	–0.396872
H	0.486793	–2.063938	–1.316305
H	–1.138174	1.675279	–2.780504
H	–3.319227	1.074611	–1.815848
C	2.364897	–0.615460	–2.451268
H	2.086144	–1.654187	–2.695376
H	3.144613	–0.323311	–3.180391
N	3.008208	–0.697937	–1.096874
C	3.919248	–1.828930	–1.032070
H	4.731752	–1.753097	–1.779375
H	4.37535	–1.892236	–0.036993
H	3.371693	–2.758987	–1.222814
C	3.739107	0.528437	–0.817561
H	4.11803	0.523408	0.212308
H	4.600615	0.66031	–1.500139
H	3.074138	1.388875	–0.959934
C	1.195456	0.302573	–2.527917
H	1.276382	1.1893	–3.152810
O	1.440551	–1.406334	2.408978
O	–1.436969	0.481698	1.858131
O	0.54558	3.11254	0.60034
C	0.207165	4.114632	1.530912
H	–0.824074	4.462601	1.374749
H	0.292297	3.679541	2.528746
H	0.888083	4.972907	1.444956
C	0.399327	3.545081	–0.741358
H	1.045555	4.411819	–0.938278
H	0.693049	2.711258	–1.387036
H	–0.645795	3.815298	–0.946093
C	1.266757	–1.641192	3.788064
H	1.034968	–0.683344	4.257836
H	0.437724	–2.341093	3.966158
H	2.182962	–2.056185	4.230267
C	1.658622	–2.598472	1.678601

H	0.780206	–3.256762	1.73961
H	1.832779	–2.311328	0.638497
H	2.539528	–3.132595	2.061845
C	–1.990642	–0.715922	2.367236
H	–1.180080	–1.447998	2.41442
H	–2.397679	–0.553333	3.37681
H	–2.781900	–1.088945	1.704991
C	–2.431851	1.448109	1.5843
H	–2.868155	1.827542	2.520572
H	–1.947056	2.265708	1.044685
H	–3.222037	1.018346	0.95648
K	0.875245	0.449592	0.548046
O	–3.879439	–1.109325	–0.426218
C	–4.787666	–1.756870	–1.299499
H	–5.699362	–1.953643	–0.729363
H	–5.024437	–1.119419	–2.161290
H	–4.368777	–2.703601	–1.664311

Table S114 Coordinates of QM105.

Element	X	Y	Z
C	–2.773433	–1.763851	0.613147
C	–1.972916	–1.600803	1.736127
C	–1.699749	–0.327362	2.259695
C	–2.295549	0.778569	1.640243
C	–3.109741	0.629137	0.519959
C	–3.334706	–0.644255	–0.009475
H	–2.99171	–2.746703	0.205089
H	–1.534625	–2.476747	2.211424
H	–2.10292	1.775682	2.021692
H	–3.544867	1.513042	0.066006
C	–0.779309	3.721541	0.56484
H	–0.869715	4.327797	1.500713
H	–0.458703	4.45919	–0.216634
H	–1.804204	3.41504	0.291854
C	1.380391	3.052096	1.05989
H	1.871667	3.725518	0.306027
H	1.40377	3.63903	2.012433
H	2.078874	2.205634	1.212655
N	0.081554	2.589089	0.684316
O	2.403198	–1.659737	1.266433
C	3.034598	–0.78133	2.176408
H	3.653956	–1.34008	2.892579
H	3.66575	–0.061085	1.635099
H	2.250355	–0.247182	2.718047
C	3.347953	–2.409487	0.537195
H	3.862307	–3.127931	1.192039
H	2.810081	–2.945552	–0.248468
H	4.092771	–1.746176	0.075472
O	0.201293	0.997665	–2.461732
C	–1.184352	1.216209	–2.276261
H	–1.662251	0.238089	–2.150953
H	–1.352698	1.83032	–1.380043
H	–1.617113	1.715587	–3.15691
C	0.886127	2.243188	–2.525784

H	0.483228	2.851702	–3.348566
H	0.781492	2.766967	–1.565258
H	1.936576	2.026714	–2.726691
K	0.565686	0.099283	0.166536
O	0.310506	–2.348278	–1.011922
C	0.166075	–3.537112	–0.266244
H	0.636287	–3.367051	0.705373
H	–0.894366	–3.783799	–0.122188
H	0.658352	–4.377145	–0.7784
C	–0.383278	–2.389301	–2.243289
H	–1.468496	–2.444863	–2.072781
H	–0.141147	–1.467947	–2.780277
H	–0.06643	–3.256746	–2.840056
C	–0.051464	0.943694	3.622841
H	0.623491	0.993377	4.473589
H	–0.103917	1.801952	2.947739
C	–0.759229	–0.168908	3.384903
H	–0.632903	–1.042582	4.026189
O	–4.077083	–0.891751	–1.125634
C	–4.762443	0.197286	–1.711625
H	–5.322625	–0.217485	–2.549777
H	–5.455047	0.656649	–0.996504
H	–4.064084	0.958326	–2.081894
C	3.032662	–0.501309	–2.124253
H	2.242876	–1.241674	–1.975446
H	2.832067	0.062797	–3.045722
H	4.002151	–1.013542	–2.22138
C	4.077194	1.292629	–1.030897
H	5.051695	0.787748	–0.947926
H	4.059417	1.873768	–1.96386
H	3.936184	1.97123	–0.187688
O	3.027408	0.351394	–0.999082

Table S115 Coordinates of QM106.

Element	X	Y	Z
C	3.181725	–0.016971	–1.584111
C	2.033671	–0.80454	–1.527172
C	1.85984	–1.781743	–0.523527
C	2.886687	–1.893876	0.437046
C	4.025318	–1.097824	0.396891
C	4.177564	–0.155266	–0.620061
H	3.319857	0.709099	–2.381967
H	1.271153	–0.678791	–2.294121
H	2.796287	–2.654962	1.208945
H	4.817082	–1.214926	1.132668
C	–0.20053	–2.848316	–1.468641
H	–0.023341	–2.447493	–2.463012
H	–0.942385	–3.637945	–1.405083
N	–2.212283	–1.573397	–1.634938
C	–2.480574	–1.865262	–3.010738
H	–2.313529	–2.938899	–3.276015
H	–3.529903	–1.653035	–3.312113
H	–1.829813	–1.273347	–3.67261
C	–3.077148	–2.378309	–0.823373

H	–4.159215	–2.165046	–0.97506
H	–2.974702	–3.476215	–1.004878
H	–2.87379	–2.22022	0.249992
C	0.7026	–2.672858	–0.465823
H	0.590144	–3.245675	0.455021
O	–2.968274	0.763572	1.336978
O	0.567951	2.268552	0.521779
O	–0.338288	–0.929172	2.587811
C	–1.468462	–1.732042	2.870846
H	–1.640497	–1.788949	3.955443
H	–2.328171	–1.264402	2.387471
H	–1.33093	–2.747332	2.472617
C	0.820483	–1.434944	3.213974
H	1.001085	–2.477219	2.915075
H	1.668784	–0.82516	2.895834
H	0.72001	–1.390183	4.308031
C	–3.292872	1.331393	2.581992
H	–2.361234	1.438792	3.142641
H	–3.758513	2.319192	2.452059
H	–3.984674	0.685647	3.143804
C	–4.098612	0.543749	0.514596
H	–4.628884	1.489601	0.328072
H	–3.733361	0.121509	–0.427117
H	–4.791185	–0.164547	0.992392
C	1.165926	2.951958	–0.558803
H	0.361399	3.274342	–1.222251
H	1.72715	3.827543	–0.200292
H	1.846663	2.2896	–1.109975
C	1.524175	1.737254	1.416667
H	2.186604	2.528528	1.798005
H	0.97004	1.295654	2.248751
H	2.13665	0.963425	0.927412
K	–0.798939	–0.063722	–0.016485
O	5.31786	0.618183	–0.685685
C	5.211104	1.832424	0.035224
H	5.013857	1.64111	1.098905
H	6.165514	2.350732	–0.072479
H	4.404581	2.460336	–0.369187
C	–2.733639	1.834332	–2.416385
H	–3.80643	1.947036	–2.199797
H	–2.454153	2.549441	–3.203833
H	–2.542631	0.807344	–2.735292
C	–2.270022	3.263538	–0.606021
H	–2.065652	4.123122	–1.263389
H	–3.330195	3.294974	–0.313004
H	–1.642352	3.320632	0.285775
O	–1.965363	2.050742	–1.250277

Table S116 Coordinates of QM107.

Element	X	Y	Z
C	–2.640646	0.694815	–1.299958
C	–1.562541	1.544643	–1.070323
C	–1.303894	2.13077	0.219534
C	–2.271997	1.787147	1.228364

C	–3.334853	0.937955	0.985806
C	–3.526958	0.366617	–0.279398
H	–2.792436	0.261006	–2.288257
H	–0.888365	1.762801	–1.897763
H	–2.161898	2.230205	2.217387
H	–4.035915	0.695439	1.784093
C	0.740537	3.394305	–0.565482
H	0.197929	3.580911	–1.506532
H	1.208972	4.356828	–0.279597
N	1.843987	2.447219	–0.933186
C	2.499491	2.879528	–2.155493
H	2.950258	3.886094	–2.055638
H	3.295027	2.175826	–2.427629
H	1.771179	2.913536	–2.972644
C	2.815792	2.381854	0.145601
H	3.602941	1.659533	–0.098754
H	3.288951	3.365877	0.33372
H	2.316465	2.06877	1.071228
C	–0.174533	2.915022	0.506843
H	–0.13756	3.421268	1.469544
O	1.200335	–1.87977	–1.927001
O	–1.178054	–1.999017	0.212428
O	0.823522	–0.556632	2.619784
C	0.996148	–1.719534	3.393645
H	0.045038	–2.040836	3.841606
H	1.362548	–2.504277	2.726958
H	1.726532	–1.551012	4.199052
C	0.301015	0.529142	3.365967
H	0.986265	0.798427	4.182609
H	0.192914	1.371105	2.67498
H	–0.680909	0.270758	3.786653
C	1.472104	–3.142505	–1.359054
H	1.406866	–3.03142	–0.275324
H	0.735346	–3.886585	–1.69354
H	2.480058	–3.48524	–1.636857
C	1.202107	–1.905141	–3.336189
H	0.436709	–2.59667	–3.716758
H	0.980921	–0.893382	–3.681286
H	2.183766	–2.218272	–3.721976
C	–1.832352	–2.602173	–0.88632
H	–1.251222	–2.359959	–1.779149
H	–1.874858	–3.695394	–0.757955
H	–2.85004	–2.206667	–0.994043
C	–1.935726	–2.121132	1.399603
H	–1.887967	–3.152326	1.783352
H	–1.507416	–1.429074	2.128184
H	–2.981395	–1.843425	1.219296
K	0.674574	–0.025889	–0.026158
O	–4.549892	–0.557047	–0.490877
C	–5.772841	0.042986	–0.877359
H	–6.502616	–0.761226	–1.003485
H	–6.126354	0.742011	–0.108238
H	–5.657354	0.587393	–1.82382
C	3.959795	–0.75502	1.498398

H	4.535356	–1.658758	1.745825
H	4.649282	0.099205	1.434354
H	3.215614	–0.56118	2.272366
C	4.128915	–1.079109	–0.813785
H	4.725781	–1.997862	–0.71428
H	3.509623	–1.134772	–1.710953
H	4.81278	–0.221171	–0.894059
O	3.258714	–0.924675	0.284944

5 References

[1] G. M. Sheldrick, *Acta Cryst*. **2008**, *A64*, 112-122.

[2] O. V. Dolomanov, L. J. Bourhis, R. J Gildea, J. A. K. Howard, H. Puschmann, *J. Appl. Cryst.* **2009**, *42*, 339-341.

[3] a) T. Kottke, D. Stalke, *J. Appl. Crystallogr.* **1993**, *26*, 615; b) D. Leusser, *Dissertation* **2002**, Universität Würzburg; c) G. M. Sheldrick, *SHELXL97*, *A Programm for the Solu-tion of Crystal Structures*, **1990**, Universität Göttingen; d) G. M. Sheldrick, *SHELXL97*, *A Program for Crystal Structure Refinement*, **1990**, Universität Göttingen.

[4] Ortep–3 V 2.02 for Windows, L. J. Farrugia, *J. Appl. Cryst.* **1997**, *30*, 565.

[5] Gaussian 09, Revision E.01, M. J. Frisch, G. W. Trucks, H. B. Schlegel, G. E. Scuseria, M. A. Robb, J. R. Cheeseman, G. Scalmani, V. Barone, B. Mennucci, G. A. Petersson, H. Nakatsuji, M. Caricato, X. Li, H. P. Hratchian, A. F. Izmaylov, J. Bloino, G. Zheng, J. L. Sonnenberg, M. Hada, M. Ehara, K. Toyota, R. Fukuda, J. Hasegawa, M. Ishida, T. Nakajima, Y. Honda, O. Kitao, H. Nakai, T. Vreven, J. A. Montgomery, Jr., J. E. Peralta, F. Ogliaro, M. Bearpark, J. J. Heyd, E. Brothers, K. N. Kudin, V. N. Staroverov, T. Keith, R. Kobayashi, J. Normand, K. Raghavachari, A. Rendell, J. C. Burant, S. S. Iyengar, J. Tomasi, M. Cossi, N. Rega, J. M. Millam, M. Klene, J. E. Knox, J. B. Cross, V. Bakken, C. Adamo, J. Jaramillo, R. Gomperts, R. E. Stratmann, O. Yazyev, A. J. Austin, R. Cammi, C. Pomelli, J. W. Ochterski, R. L. Martin, K. Morokuma, V. G. Zakrzewski, G. A. Voth, P. Salvador, J. J. Dannenberg, S. Dapprich, A. D. Daniels, O. Farkas, J. B. Foresman, J. V. Ortiz, J. Cioslowski, D. J. Fox, Gaussian, Inc., Wallingford CT, **2013**.

[6] Y. Zhao, D.G. Truhlar*, Theor Chem Acc.* **2006**, *120,* 215–241.
